# Selective C-H trifluoromethoxylation of (hetero)arenes as limiting reagent

**DOI:** 10.1038/s41467-020-16451-x

**Published:** 2020-05-22

**Authors:** Zhijie Deng, Mingxin Zhao, Feng Wang, Pingping Tang

**Affiliations:** State Key Laboratory and Institute of Elemento-Organic Chemistry, College of Chemistry, Nankai University, 300071 Tianjin, China

**Keywords:** Drug discovery and development, Diversity-oriented synthesis, Synthetic chemistry methodology

## Abstract

Methods for direct C-H trifluoromethoxylation of arenes and heteroarenes are rare, despite the importance of trifluoromethoxylated compounds for pharmaceuticals, agrochemicals, and material sciences. Especially selective C-H trifluoromethoxylation of pyridines remains a formidable challenge. Here we show a general late-stage C-H trifluoromethoxylation of arenes and heteroarenes as limiting reagent with trifluoromethoxide anion. The reaction is mediated by silver salts under mild reaction conditions, exhibiting broad substrate scope and wide functional-group compatibility. In addition, *ortho*-position selective C-H trifluoromethoxylation of pyridines is observed. The method is not only applicable to the gram-scale synthesis of trifluoromethoxylated products but also allows efficient late-stage C-H trifluoromethoxylation of marketed small-molecule drugs, common pharmacophores and natural products.

## Abstract (web summary)

Selective C-H trifluoromethoxylation of pyridines remains a formidable synthetic challenge. Here, the authors report a silver-mediated late-stage C-H trifluoromethoxylation of arenes and heteroarenes as limiting reagents with trifluoromethoxide anion.

## Introduction

**Fig. 1:**
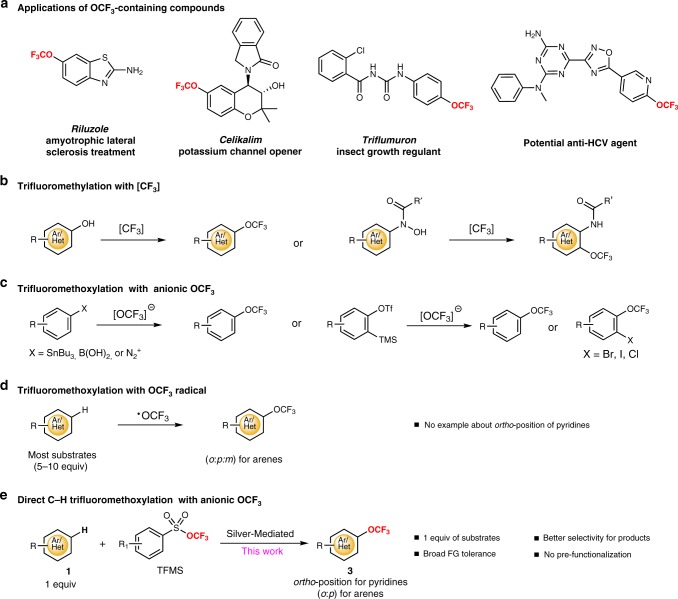
Arene and heteroarene C-H trifluoromethoxylation. **a** Applications of OCF_3_-containing compounds. **b** Trifluoromethylation with [CF_3_]. **c** Trifluoromethoxylation with anionic OCF_3_. **d** Trifluoromethoxylation with OCF_3_ radical. **e** Direct C-H trifluoromethoxylation with anionic OCF_3_.

Trifluoromethoxy (OCF_3_) groups are becoming increasingly important in pharmaceuticals, agrochemicals, and material sciences. In particular, the introduction of trifluoromethoxy groups into arenes and heteroarenes is of paramount importance in organic molecules because of their outstanding electronegativity and high lipophilicity (Fig. 1a)^1–3^. For example, Riluzole is the first approved drug for the treatment of patients with neurological diseases such as amyotrophic lateral sclerosis^4^; Celikalim is a potent potassium channel opener in human airway smooth muscles^5^. Many pesticides also contain the OCF_3_ group, such as Triflumuron, Indoxacarb, and Thifluzamide^6^. In addition, 2-(trifluoromethoxy)pyridine and 2-(trifluoromethoxy)quinoline derivatives are especially useful for drugs and agrochemicals^7^. Despite the development of methods for the synthesis of trifluoromethoxylated compounds that has been an area of great interest, only a few examples have been reported for the synthesis of trifluoromethoxylated arenes and heteroarenes^8–13^. Especially the most atom economical C-H trifluoromethoxylation of arenes and heteroarenes remains a formidable challenge due to the reversible decomposition of trifluoromethoxide anion and the limited number of available trifluoromethoxylation reagents^14–32^. Traditional methods for the synthesis of aryl trifluoromethyl ethers employed classical approaches such as reactions of phenols via fluoroformate or chlorothionoformate intermediates, followed by nucleophilic fluorination with antimony trifluoride, hydrofluoric acid, or sulfur tetrafluoride at 100–160 °C^33–37^. The harsh reaction conditions can only be tolerated by structurally simple phenol derivatives. In this context, Togni reported the successful trifluoromethylation of 2,4,6-trimethylphenol with the Togni’s reagent and up to 15% yield of the desired aryl trifluoromethyl ether^38^, while Wu reported the synthesis of *N*-heteroaromatic trifluoromethoxy compounds by direct O-CF_3_ bond formation with the Togni’s reagent^39^. Umemoto reported the trifluoromethylation of phenols using Umemoto’s reagent under photoirradiation at −100 °C^40^. In 2014, Ngai reported a two-step sequence of *O*-trifluoromethylation of *N*-aryl-*N*-hydroxylamine and intramolecular OCF_3_ migration to prepare trifluoromethoxylated aniline derivatives^41,42^. Qing reported a silver-mediated oxidative trifluoromethylation of phenols with Ruppert–Prakash reagent^43^. Both of these reactions were realized with CF_3_ reagents (Fig. 1b). Recently, Ritter reported a silver-mediated cross-coupling of aryl stannanes and aryl boronic acids to produce trifluoromethoxylated arenes with tris(dimethylamino)sulfonium trifluoromethoxide^44^. The trifluoromethoxylation of arenediazonium tetrafluoroborates with stoichiometric silver or stoichiometric copper was described by Tang and our group^45,46^. The synthesis of phenyl and naphthyl trifluoromethyl ether was reported by the groups of Kolomeitsev and Hu, via the addition of trifluoromethoxide to benzyne and α-naphthyne generated in situ^47,48^. Hartwig reported the trifluoromethoxylation of quinoline and phenanthridine *N*-oxide to synthesize heteroaromatic trifluoromethyl ethers^49^. These reactions utilized pre-functionalized arenes and heterocyclic *N*-oxide as substrates, which might hamper the applicability for late-stage modifications of complex small molecules (Fig. 1c).

Direct functionalization of a C-H bond with a trifluoromethoxy group would be advantageous for the step-economic synthesis of trifluoromethoxy ethers. However, only a few examples of direct C-H trifluoromethoxylation of (hetero)arenes were reported to date. In 2018, the groups of Ngai and Togni independently reported the intermolecular C-H trifluoromethoxylation of (hetero)arenes employing a photoredox catalyst with the *N*-OCF_3_ reagents^50–52^. Mechanism involving a OCF_3_ radical was proposed. However, excess of (hetero)arenes was required to achieve good yields of the desired products as mixture of isomers (*o*:*m*:*p*). In addition, no example of *ortho*-position selective C-H trifluoromethoxylation of pyridines was reported (Fig. 1d). It is certainly highly desirable to develop an efficient and general approach for the selective (hetero)aromatic C-H trifluoromethoxylation, preferably with 1 equivalent of (hetero)arenes, which would be a powerful tool for late-stage trifluoromethoxylation of drug candidates, agrochemicals, and natural products. Herein we report a silver-mediated direct C-H trifluoromethoxylation with arenes and heteroarenes as the limiting reagent (Fig. 1e). Compared to previous reports, exclusive *ortho*-selective C-H trifluoromethoxylation of pyridines and selective C-H trifluoromethoxylation of *ortho*- and *para*-position of arenes are achieved. This method not only exhibits high efficiency and wide functional-group compatibility but also allows the late-stage trifluoromethoxylation of bioactive molecules and natural product derivatives.

## Results

### Investigations of reaction conditions and scope

In order to improve the regioselectivity of the direct (hetero)aromatic C-H trifluoromethoxylation reactions, we envisioned to in situ generate the proposed Ag(II)OCF_3_ intermediate in the presence of silver salts, oxidant, and trifluoromethyl arylsulfonate (TFMS), which was triggered by fluoride ions to deliver OCF_3_ anion as reported in our previous results^53–55^. The Ag(II)OCF_3_ intermediate could coordinate with pyridine substrates and give the desired products through the trifluoromethoxylation at the *ortho*-position of pyridines. In order to test our hypothesis, we chose 4-(*tert*-butyl)pyridine **1a** as the model substrate for the direct trifluoromethoxylation of C(sp^2^)-H bonds (Table 1). No desired product **3a** was observed in the absence of silver salts (Table 1, entry 1). We were delighted to find that 62% yield of the desired product **3a** was observed when AgF_2_ was used as the silver salts (Table 1, entries 2–6). The oxidant was important for this transformation, and Selectfluor could give the best yield. Switching to other oxidants, Selectfluor II, *N*-fluorobenzenesulfonimide, PhI(OAc)_2_, and K_2_S_2_O_8_ could generate **3a** in lower yields (Table 1, entries 7–10). In addition, CsF was the best fluoride source, which reacted with TFMS to generate CsOCF_3_. The poorly soluble CsOCF_3_ ensured that the intermediate Ag(II)OCF_3_ was generated gradually, which might slow down the decomposition of OCF_3_ anion. Less than 10% yields of the desired products were detected in control experiments with no CsF or oxidant. It was worth mentioning that exclusive selective for C-H trifluoromethoxylation adjacent to nitrogen was observed. The main by-product was 4-(*tert*-butyl)−2-fluoropyridine, which was generated from fluorination of pyridines with AgF_2_. After extensive screening of various solvents, different substitutions on TFMS, and temperatures (see more details in Supplementary Tables 1–8), the ideal conditions under 1.0 equiv of AgF_2_, 2.0 equiv of Selectfluor, 3.0 equiv of CsF, 3.0 equiv of TFMS (**2**) in dimethyl carbonate (DMC) under N_2_ atmosphere at 35 °C were found to produce the highest yields of the desired product **3a** (method A).

**Table 1:** Optimization of reaction conditions^a^.


Entry	Silver salt	Fluoride	Oxidant	Yield (%)^b^
1	None	CsF	Selectfluor	0
2	Ag_2_O	CsF	Selectfluor	48
3	Ag_2_CO_3_	CsF	Selectfluor	50
4	AgF	CsF	Selectfluor	53
5	AgO	CsF	Selectfluor	46
6	AgF_2_	CsF	Selectfluor	62 (58)^c^
7	AgF_2_	CsF	Selectfluor II	47
8	AgF_2_	CsF	K_2_S_2_O_8_	44
9	AgF_2_	CsF	NFSI	0
10	AgF_2_	CsF	PhI(OAc)_2_	0
11	AgF_2_	CsF	None	9
12	AgF_2_	KF	Selectfluor	57
13	AgF_2_	NaF	Selectfluor	11
14	AgF_2_	Et_3_N•HF	Selectfluor	0
15	AgF_2_	None	Selectfluor	10

DMC: dimethyl carbonate.

^a^Reaction conditions: 4-(*tert*-butyl)-pyridine (**1a**) (1.0 equiv), silver salt (1.0 equiv), oxidant (2.0 equiv), fluoride (3.0 equiv), TFMS (2) (3.0 equiv), DMC, N_2_, 35 °C.

^b^Yields were determined by ^19^F NMR with benzotrifluoride as an internal standard.

^c^Isolated yield.

**Fig. 2:**
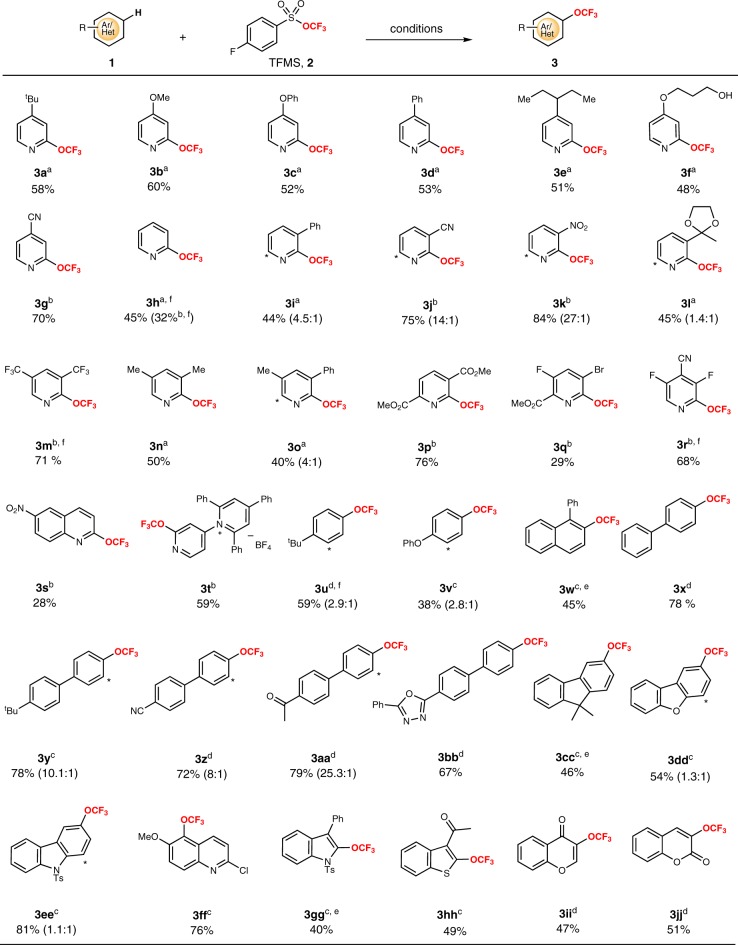
Substrate scope of simple arenes and heteroarenes. ^a^Method A: 1 (1.0 equiv), AgF_2_ (1.0 equiv), TFMS (**2**) (3.0 equiv), Selectfluor (2.0 equiv), CsF (3.0 equiv), DMC, N_2_, 35 °C. ^b^Method B: 1 (1.0 equiv), AgF_2_ (4.0 equiv), TFMS (**2**) (4.0 equiv), MeCN, N_2_, 10 °C. ^c^Method C: 1 (1.0 equiv), AgF_2_ (1.0 equiv), TFMS (**2**) (4.0 equiv), Selectfluor (2.0 equiv), CsF (4.0 equiv), 4-*tert*-butyl-2,6-bis(4-*tert*-butylpyridin-2-yl)pyridine (0.1 equiv). DMC, N_2_, 35 °C. ^d^Method D: 1 (1.0 equiv), AgF_2_ (4.0 equiv), TFMS (**2**) (4.0 equiv), di(pyridin-2-yl)methanone (0.1 equiv), MeCN, N_2_, 10 °C. ^e^K_2_S_2_O_8_ was used instead of Selectfluor. ^f^Yields were determined by the ^19^F NMR spectrum using benzotrifluoride as internal standards.

With the optimized reaction conditions in hand, this method was further applied to a number of pyridines with representative electron-rich substitutions, providing the corresponding *ortho*-selective C-H trifluoromethoxylation products in good yields (Fig. 2). However, the extension of the method to the trifluoromethoxylation of pyridines with electron-deficient substitution resulted in relatively low efficiency, and there were a lot of starting material recovered. This might be attributed to that Selectfluor (*E*_1/2_^red^ = +0.33 V vs saturated calomel electrode (SCE) in CH_3_CN) is not strong enough to oxidize electron-deficient substrates well^56^. We then switched Selectfluor to AgF_2_ (the redox potential of the Ag(I)/Ag(II) couple is +1.98 V vs SCE)^57^, a stronger oxidant and powerful reagent in C-H fluorination of pyridines with either electron-donating or electron-withdrawing substituents^58–62^. Gratifyingly, the expected trifluoromethoxylation products could be achieved in high efficiency when the reaction was carried out with AgF_2_ (4.0 equiv) as the oxidant and fluoride source in MeCN solution at 10 °C (method B). The generality of the method was then clearly demonstrated by the synthesis of trifluoromethoxylated products **(3g, 3j, 3k, 3m, 3p** to **3t)** in satisfactory yields from the corresponding electron-deficient pyridines (Fig. 2). In contrast, the extension of method B to the trifluoromethoxylation of electron-rich pyridines resulted in low yields with the generation of large amounts of fluorinated by-products. Perhaps, before the Ag(II)OCF_3_ intermediate was formed, AgF_2_ reacted with electron-rich pyridines under this condition to give the fluorinated by-products.

Having established the reaction protocol for the C(*sp*^2^)-H trifluoromethoxylation of pyridines, we next focused on the C(*sp*^2^)-H trifluoromethoxylation of arenes and other heterocyclic compounds, which are certainly of high value. Direct application of the method A to the trifluoromethoxylation of model substrate 2-chloro-6-methoxyquinoline (**1ff**) resulted in a low conversion (5% yield). This phenomenon might be ascribed to the relatively low concentration and stability of Ag(II)–OCF_3_ species responsible for trifluoromethoxylation. A possible solution was adding a ligand to coordinate with Ag(II)–OCF_3_ species like pyridine, which could increase the solubility and stability of intermediate. We then screened a number of ligands based on method A. With a catalytic amount of 4,4′,4″-tri-*tert*-butyl-2,2′:6′,2″-terpyridine as the ligand, the yield of the trifluoromethoxylation reaction was increased from 5% to 76% (see Supplementary Table 9). The optimal conditions were thus determined as to run the reaction in DMC at 35 °C with AgF_2_ (1 equiv) as the reaction medium, Selectfluor (2 equiv) as the oxidant, TFMS (4 equiv) as the OCF_3_ source, CsF (4 equiv) as the fluorine source, and 4,4′,4″-tri-*tert*-butyl-2,2′:6′,2″-terpyridine (0.1 equiv) as the ligand (method C). Method C was then applied to the trifluoromethoxylation of a variety of (hetero)arenes, and the results are summarized in Fig. 2. Substrates with electron-donating substituents on the (hetero)aromatic ring all underwent C-H trifluoromethoxylation smoothly, furnishing the corresponding products in satisfactory yields. However, the same problem appeared in the relatively electron-deficient substrates, which need AgF_2_ (4 equiv) as the oxidant and di(pyridin-2-yl)methanone (0.1 equiv) as the ligand (method D). In addition, <5% yield of trifluoromethoxylated products with more electron-deficient substrates, such as (trifluoromethyl)benzene and nitrobenzene, were observed even with method D, and 11% yield was obtained when benzene was used as the substrate. Although this trifluoromethoxylation method was not suitable for electron-deficient arenes, mono-, di-, and tri-substituted pyridines with both electron-donating and electron-withdrawing groups are tolerated, and other heteroaromatic substrates, such as quinoline, indole, thiophene, chromone, and coumarin, were also successfully employed to provide the corresponding trifluoromethoxylated products (**3ee**–**3jj**). A good range of functional groups including ester, ether, ketone, acetal, nitrile, nitro, hydroxyl, and halogens were well tolerated under these mild reaction conditions. Compared to the previous reports, only 1 equiv of (hetero)arenes was used in the reaction, and exclusive *ortho*-position selective C-H trifluoromethoxylation of pyridines was achieved. In addition, compounds **3p** and **3ff** were prepared in a gram scale in 75% and 73% isolated yield, respectively, which demonstrates the scalability of this method.

**Fig. 3:**
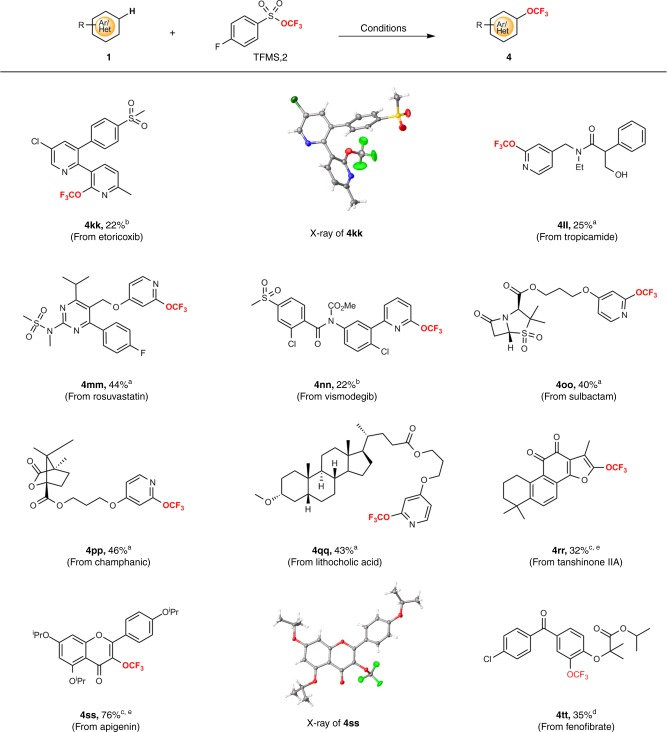
Late-stage C-H trifluoromethoxylation of natural products and drug derivatives. ^a^Method A: 1 (1.0 equiv), AgF_2_ (1.0 equiv), TFMS (**2**) (3.0 equiv), Selectfluor (2.0 equiv), CsF (3.0 equiv), DMC, N_2_, 35 °C. ^b^Method B: 1 (1.0 equiv), AgF_2_ (4.0 equiv), TFMS (**2**) (4.0 equiv), MeCN, N_2_, 10 °C. ^c^Method C: 1 (1.0 equiv), AgF_2_ (1.0 equiv), TFMS (**2**) (4.0 equiv), Selectfluor (2.0 equiv), CsF (4.0 equiv), 4-*tert*-butyl-2,6-bis(4-*tert*-butylpyridin-2-yl)pyridine (0.1 equiv). DMC, N_2_, 35 °C. ^d^Method D: 1 (1.0 equiv), AgF_2_ (4.0 equiv), TFMS (**2**) (4.0 equiv), di(pyridin-2-yl)methanone (0.1 equiv), MeCN, N_2_, 10 °C. ^e^K_2_S_2_O_8_ was used instead of Selectfluor.

The direct trifluoromethoxylation of aromatic C-H bonds could facilitate access to trifluoromethoxylated derivatives of complex small molecules that would otherwise be difficult to produce. Given that a trifluoromethoxy group is present in a range of small-molecule drugs and preclinical candidates, we examined a number of well-known natural products or drug derivatives (Fig. 3). Each of these architecturally complex molecules underwent (hetero)aryl C-H trifluoromethoxylation to achieve the corresponding trifluoromethoxylated products in moderate yields (**4kk**–**4tt**, 22–76% yield). For example, etoricoxib, a selective cyclooxygenase-2 inhibitor and a non-steroidal anti-inflammatory drug, was used as the substrate to give the corresponding trifluoromethoxylated product **4kk** in 22% yield via method B. The trifluoromethoxylation of tropicamide, an anticholinergic drug containing a unprotected hydroxyl group and an acidic α-phenyl amide, proceeded with method A to form the product **2ll**. 3-(Pyridin-4-yloxy)propyl ester of β-lactamase inhibitor sulbactam underwent *ortho*-position selective C-H trifluoromethoxylation of the pyridine ring to produce **4oo** in 40% isolated yield. In another case, Tanshinone IIA with significant effect in treating coronary heart disease was nicely converted to its trifluoromethoxylated product **4rr** in 32% yield via method C. The trifluoromethoxylation reaction of apigenin derivative, which is a natural product found in many plants, proceeded smoothly to provide the corresponding product (**4ss**) in 76% yield. Finally, a trifluoromethoxylated derivative of hypolipidemic drug fenofibrate was readily prepared in 35% yield via method D. The structure of the products **4kk** and **4ss** were confirmed by X-ray crystallographic analysis. These results illustrated the ability to conduct the late-stage (hetero)aromatic C-H trifluoromethoxylation of complex structures.

**Fig. 4:**
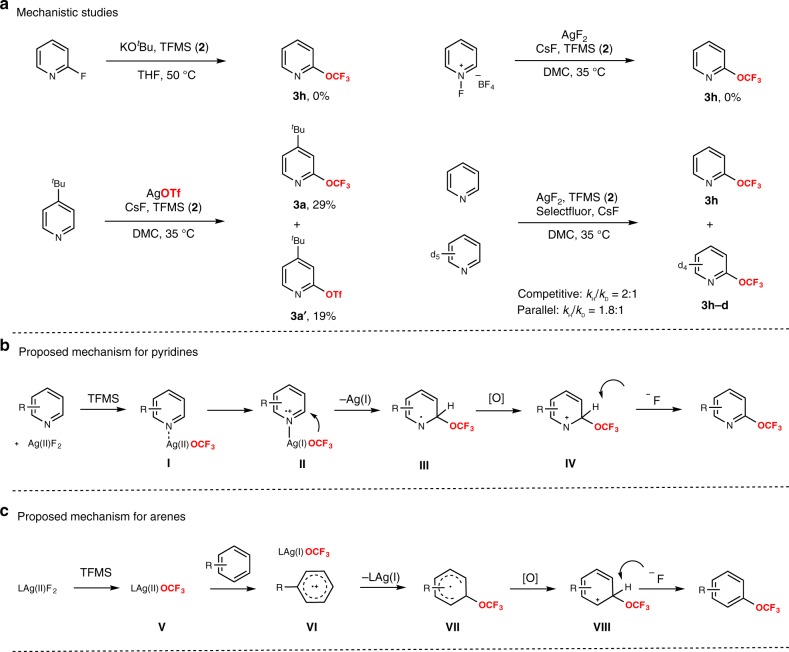
Mechanistic studies and proposed mechanism. **a** Mechanistic studies. **b** Proposed mechanism for pyridines. **c** Proposed mechanism for arenes.

Although the mechanistic details of this transformation are not clear, a series of experiments were performed to gain insight into the mechanism of the reactions (Fig. 4a). No trifluoromethoxylated product **3h** was observed when 2-fluoropyridine reacted with TFMS in the presence of the base. In all, 29% yield of trifluoromethoxylated product **3a** and 19% yield of by-product **3a’** were observed when AgF_2_ was replaced by AgOTf. These results indicated that the trifluoromethoxylated products were not generated from nucleophilic aromatic substitutions (S_N_Ar) of the 2-fluoropyridine^63^. When *N*-fluoropyridinium salt was used as the substrate, no desired product **3h** was observed. Furthermore, kinetic isotopic effect experiments were conducted by treatment of pyridine and deuterated pyridine under the reaction conditions. A *k*_H_/*k*_D_ value of 2.1 was obtained in a competitive reaction and 1.8 in parallel reactions on the basis of ^19^F nuclear magnetic resonance analysis, which was indicative of a primary kinetic isotope effect. In addition, no trifluoromethoxylated product was formed when the radical inhibitor 2,2,6,6-tetramethyl-1-piperidinyloxy (1 equiv) or 2,6-di-*tert*-butyl-4-methylphenol (4 equiv) was added, and electron paramagnetic resonance experiments were performed with the addition of free radical spin trapping agent α-(4-pyridyl *N*-oxide)-*N*-*tert*-butylnitrone (POBN). The signals of unknown organic radicals were observed, which did not match the previously reported POBN-OCF_3_• adducts^51^ (see more details in Supplementary Fig. 2). Furthermore, several pieces of data indicated that a free OCF_3_ radical was not involved in the reaction. First, the exclusive selectivity for trifluoromethoxylation at the 2-position of pyridines was observed, which contrasts with the mixture of isomeric products formed in varying amounts for the addition of alkyl, aryl, and fluoroalkyl radicals to pyridines depending on the steric and electronic properties of the reactants^64–67^. Second, 2,6-dimethylpyridine did not undergo trifluoromethoxylation, while an OCF_3_ radical should add to the 3- or 4-position. Third, C-H trifluoromethoxylation of *tert*-butylbenzene (**3u**) could get (*o*,*p*)-products, which was different from the previous report with (*o*:*m*:*p*) isomers via an OCF_3_ radical by Ngai^52,68^. Finally, compared to the previous reports with moderate yields, <5% yield of trifluoromethoxylated products were observed using more electron-deficient arenes as the substrates in our reaction.

On the basis of these mechanistic investigations, we proposed the mechanism depicted in Fig. 4. When pyridines were used as the substrates (Fig. 4b), the mechanism would involve initial coordination of AgF_2_ to pyridines, then reacted with TFMS to produce intermediate **I**, followed by single-electron transfer between Ag(II)OCF_3_ and pyridines leads to the generation of intermediated **II**, followed by addition OCF_3_ anion to get intermediate **III**, which is oxidized to form cation intermediate **IV**, then generated the desired products by proton abstraction. When arenes were the substrates (Fig. 4c), the mechanism was similar to pyridines with the production of intermediate **V** first. The subsequent single-electron transfer between Ag(II)OCF_3_ and arenes leads to the generation of Ag(I)OCF_3_ species and intermediated **VI**, followed by addition OCF_3_ anion to get intermediate **VII**, which is oxidized to form carbocation intermediate **VIII**, then generated the desired products by proton abstraction.

## Discussion

In conclusion, we have presented a silver-mediated late-stage C-H trifluoromethoxylation reaction with arenes and heteroarenes as limiting reagent, which allows efficient late-stage trifluoromethoxylation of a variety of organic molecules and known drugs. Although the trifluoromethoxylation method reported herein needs stoichiometric amount silver salts, it affords exclusive *ortho*-position selective C-H trifluoromethoxylation of pyridines. The reaction is operationally simple under mild conditions, tolerates a large range of functional groups, and is applicable to the gram-scale synthesis of trifluoromethoxylated products. It is anticipated to serve for the late-stage modification to quickly obtain structural diversity in drug development.

## Methods

### General procedure for the synthesis of trifluoromethoxylated products

Method A: In a glove box, to a 15.0-mL sealed tube were added in sequence Selectfluor (354 mg, 1.00 mmol, 2.00 equiv), AgF_2_ (72 mg, 0.50 mmol, 1.00 equiv), CsF (228 mg, 1.50 mmol, 3.00 equiv), 10.0 mL DMC, pyridines (0.50 mmol, 1.00 equiv), and TFMS (240 μL, 1.50 mmol, 3.00 equiv). The mixture was stirred at 35 °C for 24 h. After cooling to 23 °C, the reaction mixture was filtered through a short plug of silica gel eluting with approximately 25 mL of CH_2_Cl_2_ or EtOAc. The filtrate was concentrated, and the residue was purified by chromatography on silica gel.

Method B: In a glove box, to a 2.0-mL sealed tube were added in sequence pyridines (0.50 mmol, 1.00 equiv), 0.5 mL MeCN (the solvent was pre-cooled to −30 °C), and TFMS (320 μL, 2.00 mmol, 4.00 equiv), then AgF_2_ (290 mg, 2.0 mmol, 4.00 equiv) was added at once in one portion. The mixture was stirred at 10 °C for 36 h. After warming up to 23 °C, the reaction mixture was filtered through a short plug of silica gel eluting with approximately 25 mL of CH_2_Cl_2_ or EtOAc. The filtrate was concentrated, and the residue was purified by chromatography on silica gel.

Method C: In a glove box, to a 15.0-mL sealed tube were added in sequence Selectfluor (354 mg, 1.0 mmol, 2.00 equiv) or K_2_S_2_O_8_ (270 mg, 1.0 mmol, 2.00 equiv), AgF_2_ (72 mg, 0.50 mmol, 1.00 equiv), CsF (304 mg, 2.00 mmol, 4.00 equiv), 4-*tert*-butyl-2,6-bis(4-*tert*-butylpyridin-2-yl)pyridine (20.0 mg, 0.05 mmol, 0.10 equiv), 10.0 mL DMC, arenes or heteroarenes (0.50 mmol, 1.00 equiv), and TFMS (320 μL, 2.00 mmol, 4.00 equiv). The mixture was stirred at 35 °C for 24 h. After cooling to 23 °C, the reaction mixture was filtered through a short plug of silica gel eluting with approximately 25 mL of CH_2_Cl_2_ or EtOAc. The filtrate was concentrated, and the residue was purified by chromatography on silica gel.

Method D: In a glove box, to a 2.0-mL sealed tube were added in sequence di(pyridin-2-yl)methanone (9.2 mg, 0.05 mmol, 0.10 equiv), AgF_2_ (290 mg, 1.0 mmol, 4.00 equiv), 1.0 mL MeCN, arenes or heteroarenes (0.50 mmol, 1.00 equiv), and TFMS (320 μL, 2.00 mmol, 4.00 equiv). The mixture was stirred at 10 °C for 36 h. After warming up to 23 °C, the reaction mixture was filtered through a short plug of silica gel eluting with approximately 25 mL of CH_2_Cl_2_ or EtOAc. The filtrate was concentrated, and the residue was purified by chromatography on silica gel.

## Supplementary information

Supplementary Information

## Data Availability

The authors declare that the data supporting this study are available within the article and Supplementary Information files. The X-ray crystallographic coordinates for compounds (**4kk, 4ss**) reported in this study have been deposited at the Cambridge Crystallographic Data Centre (CCDC), under deposition numbers 1954409 and 1954410. These data can be obtained free of charge from The Cambridge Crystallographic Data Centre via www.ccdc.cam.ac.uk/data_request/cif.
