# Supplementary material for: Selective C-H trifluoromethoxylation of (hetero)arenes as limiting reagent
Source: Nat Commun. 2020 May 22;11:2569. doi: 10.1038/s41467-020-16451-x (PMC7244481; doi:10.1038/s41467-020-16451-x)
Supplement: Supplementary file 1 — Supplementary Information [file 41467_2020_16451_MOESM1_ESM.pdf]

Supplementary information

Selective C-H Trifluoromethoxylation of (Hetero)Arenes as Limiting Reagent

Deng *et al.*

Table of contents

Supplementary methods 1
Experimental Data 2
Optimization of the reaction condition for the trifluoromethoxylation 2
Optimization of solvents on the reaction 2
Optimization of oxidants on the reaction 2
Optimization of silver salts on the reaction 3
Effect of fluorine sources on the reaction 3
Effect of "OCF_3" sources on the reaction 4
Effect of temperature on the reaction 5
Effect of atmospheres on the reaction 6
Effect of sizes of sealed tube on the reaction 6
Effect of ligands on the reaction about aromatic compound 7
Procedure for trifluoromethoxylation of arenes and heteroarenes 8
General procedure A : 8
General procedure B : 8
General procedure C : 8
General procedure D : 8
Trifluoromethoxylation of arenes and heteroarenes (related to Experimental Procedures)-4-(tert-butyl)-2-(trifluoromethoxy)pyridine (**3a**) 9
4-Methoxy-2-(trifluoromethoxy)pyridine (**3b**) 9
4-Phenoxy-2-(trifluoromethoxy)pyridine (**3c**) 10
4-Phenyl-2-(trifluoromethoxy)pyridine (**3d**) 10
4-(Pentan-3-yl)-2-(trifluoromethoxy)pyridine (**1e**) 11
3-((2-(Trifluoromethoxy)pyridin-4-yl)oxy)propan-1-ol (**3f**) 11
2-(Trifluoromethoxy)isonicotinonitrile (**3g**) 12
2-(Trifluoromethoxy)pyridine (**3h**) 12
3-Phenyl-2-(trifluoromethoxy)pyridine (**3i**) and 5-phenyl-2-(trifluoromethoxy) Pyridine (**iso-3i**) 13
2-(Trifluoromethoxy)nicotinonitrile (**3j**) and 6-(trifluoromethoxy)nicotinonitrile (**iso-3j**) 13
3-Nitro-2-(trifluoromethoxy)pyridine (**3k**) and 5-nitro-2-(trifluoromethoxy)pyri -dine (**iso-3k**) 14
3-(2-Methyl-1,3-dioxolan-2-yl)-2-(trifluoromethoxy)pyridine (**3l**) and 5-(2-methyl-1,3-dioxolan-2-yl)-2-(trifluoromethoxy)pyridine (**iso-3l**) 14
2-(Trifluoromethoxy)-3,5-bis(trifluoromethyl)pyridine (**3m**) 15
3,5-Dimethyl-2-(trifluoromethoxy)pyridine (**3n**) 16
5-Methyl-3-phenyl-2-(trifluoromethoxy)pyridine (**3o**) and 3-methyl-5-phenyl-2-(trifluoro-methoxy)pyridine (**iso-3o**) 16
Dimethyl 6-(trifluoromethoxy)pyridine-2,5-dicarboxylate (**3p**) 17
Methyl 5-bromo-3-fluoro-6-(trifluoromethoxy)picolinate (**3q**) 17
3,5-Difluoro-2-(trifluoromethoxy)isonicotinonitrile (**3r**) 18

6-Nitro-2-(trifluoromethoxy)quinoline (**3s**) 18
2,4,6-Triphenyl-2'-(trifluoromethoxy)-[1,4'-bipyridin]-1-ium tetrafluoroborate (**3t**)...19
(Trifluoromethyl)benzene....................19
1-(*tert*-Butyl)-4-(trifluoromethoxy)benzene (**3u**) and 1-(*tert*-butyl)-2-(trifluoromethoxy)benzene (**iso-3u**)....................21
1-Phenoxy-4-(trifluoromethoxy)benzene (**3v**) and 1-phenoxy-2-(trifluoromethoxy) benzene (**iso-3v**) 22
1-Phenyl-2-(trifluoromethoxy)naphthalene (**3w**) 23
4-(Trifluoromethoxy)-1,1'-biphenyl (**3x**) and 4,4'-bis(trifluoromethoxy)- 1, 1' - biphenyl (**3x'**) 23
4-(*tert*-Butyl)-4'-(trifluoromethoxy)-1,1'-biphenyl (**3y**) and 4'-(*tert*-butyl)-3-(trifluoromethoxy)-1,1'-biphenyl (**iso-3y**) 24
4'-(Trifluoromethoxy)-[1,1'-biphenyl]-4-carbonitrile (**3z**) and 3'-(trifluorometh oxy)-25 [1,1'-biphenyl]-4-carbonitrile (**iso-3z**) 25
1-(4'-(Trifluoromethoxy)-[1,1'-biphenyl]-4-yl)ethanone (**3aa**) and 1-(3'-(trifluoro methoxy)-[1,1'-biphenyl]-4-yl)ethanone (**iso-3aa**)....................25
2-Phenyl-5-(4'-(trifluoromethoxy)-[1,1'-biphenyl]-4-yl)-1,3,4-oxadiazole (**3bb**) and 2-(4'-(trifluoromethoxy)-[1,1'-biphenyl]-4-yl)-5-(4-(trifluoromethoxy)phenyl)-1,3,4-oxadiazole (**3bb'**) 26
9,9-Dimethyl-3-(trifluoromethoxy)-9H-fluorene (**3cc**) and 9,9-dimethyl-3,6-bis(trifluoromethoxy)-9H-fluorene (**3cc'**) 27
2-(Trifluoromethoxy)dibenzo[b,d]furan (**3dd**) and 4-(trifluoromethoxy)dibenzo [b,d]furan (**iso-3dd**)....................27
9-Tosyl-3-(trifluoromethoxy)-9H-carbazole (**3ee**) and 9-tosyl-1-(trifluoromethoxy)-9H-carbazole (**iso-3ee**) 2-Chloro-6-methoxy-5-(trifluoromethoxy)quinoline (**3ff**) 29
3-Phenyl-1-tosyl-2-(trifluoromethoxy)-1H-indole (**3gg**)....................29
1-(2-(Trifluoromethoxy)benzo[b]thiophen-3-yl)ethanone (**3hh**) 30
3-(Trifluoromethoxy)-4H-chromen-4-one (**3ii**)....................30
3-(Trifluoromethoxy)-2H-chromen-2-one (**3jj**) 30
5-Chloro-6'-methyl-3-(4-(methylsulfonyl)phenyl)-2'-(trifluoromethoxy)-2,3'-bipyridine (**4kk**) 31
N-ethyl-3-hydroxy-2-phenyl-*N*-((2-(trifluoromethoxy)pyridin-4-yl)methyl)propanamide (**4ll**) 31
N-(4-(4-fluorophenyl)-6-isopropyl-5-((pyridin-4-yloxy)methyl)pyrimidin-2-yl)-*N*-methylmethanesulfonamide (**1mm**) 32
N-(4-(4-fluorophenyl)-6-isopropyl-5-(((2-(trifluoromethoxy)pyridin-4-yl)oxy)methyl) pyrimidin-2-yl)-*N*-methylmethanesulfonamide (**4mm**) 33
Methyl(4-chloro-3-(6-(trifluoromethoxy)pyridin-2-yl)phenyl)(2-chloro-4-(methylsulfonyl)benzoyl)carbamate (**4nn**) 33
(2S,5R)-3-(Pyridin-4-yloxy)propyl 3,3-dimethyl-7-oxo-4-thia-1-azabicyclo[3.2.0] heptane-2-carboxylate 4,4-dioxide (**1oo**) 34
(2S,5R)-3-((2-(Trifluoromethoxy)pyridin-4-yl)oxy)propyl 3,3-dimethyl-7-oxo-4-thia-1-azabicyclo[3.2.0]heptane-2-carboxylate 4,4-dioxide (**4oo**) 34

(1S,4R)-3-(Pyridin-4-yloxy)propyl 4,7,7-trimethyl-3-oxo-2-oxabicyclo[2.2.1]hep -tane-1-carboxylate (**1pp**)....35
(1R,4R)-3-((2-(Trifluoromethoxy)pyridin-4-yl)oxy)propyl 4,7,7-trimethyl-3-oxo-2-oxabicyclo[2.2.1]heptane-1-carboxylate (**4pp**)....36
(R)-3-(Pyridin-4-yloxy)propyl 4-((3R,5R,8R,9S,10S,13R,14S,17R)-3-methoxy-10, 13-dimethylhexadecahydro-1H-cyclopenta[a]phenanthren-17-yl)pentanoate (**1qq**)....36
(R)-3-((2-(Trifluoromethoxy)pyridin-4-yl)oxy)propyl 4-((3R,5R,8R,9S,10S,13R, 14S,17R)-3-methoxy-10,13-dimethylhexadecahydro-1H-cyclopenta[a]phenanthren-17 -yl)pentanoate (**4qq**)....37
1,6,6-Trimethyl-2-(trifluoromethoxy)-6,7,8,9-tetrahydrophenanthro[1,2-b]furan-10,11-dione (**4rr**)....38
5,7-Diisopropoxy-2-(4-isopropoxyphenyl)-3-(trifluoromethoxy)-4H-chromen-4-one (**4ss**)....38
Isopropyl 2-(4-(4-chlorobenzoyl)-2-(trifluoromethoxy)phenoxy)-2-methylpro panoate (**4tt**)....38
Gram scale synthesis of 4-((Trifluoromethoxy)methyl)phenyl benzoate (**3p**)....39
Gram scale synthesis of 2-chloro-6-methoxy-5-(trifluoromethoxy)quinoline (**3ff**)....39
Mechanism Studies....40
H/D isotope scrambling....40
X-ray Crystal Structure Data for 5-chloro-6'-methyl-3-(4-(methyl sulfonyl)phenyl)-2'-(trifluoromethoxy)-2,3'-bipyridine (**4kk**) (CCDC: 1954409)....46
X-ray Crystal Structure Data for 5,7-diisopropoxy-2-(4-isopro poxyphenyl)-3-(trifluoromethoxy)-4H-chromen-4-one (**4ss**) (CCDC: 1954410)....55
Spectrum Data....60
Supplementary References....150

Supplementary methods

All reactions were conducted in oven- or flame-dried glassware fitted with a Teflon-lined screw cap under an atmosphere of nitrogen unless otherwise noted. MeCN, EA, DCE, DMC (Dimethyl carbonate), 1,4-dioxane, Et_2O, HMPA, DMSO and DCM were dried by distillation over CaH_2. THF and toluene were dried by distillation over sodium/benzophenone. Cesium fluoride was purchased from TCI and dried at 120 °C overnight under reduced pressure. $K_2S_2O_8$ was purchased from Aladdin and dried at 50 °C overnight under reduced pressure. Silver difluoride (AgF_2) was purchased from Strem or Aldrich and stored in the glovebox (black microcrystalline solid). "OCF_3" reagents was prepared according to the reported literatures[1]. Unless otherwise noted, all other reagents and starting materials were purchased from commercial sources and used without further purification. Signal positions were recorded in ppm and the following abbreviations are used singularly or in combination to indicate the multiplicity of signals: s singlet, d doublet, t triplet, q quartet, m multiplet, Hz Hertz. For ^{1}H NMR: $CDCl_3$ = δ 7.26 ppm, CH_3CN = δ 2.10 ppm. For ^{13}C NMR: $CDCl_3$ = δ 77.16 ppm, CH_3CN = δ 116.43, 1.89 ppm. Mass spectra were obtained on Agilent 6520 Q-TOF LC/MS and Aligent 7890/5975C-GS/MSD. HRMS were obtained on VG ZAB-HS(ESI), Thermo Fisher Q-Exactive Orbitrap(ESI) and GCT Premier(EI). Preparative HPLC was performed on an UltiMate 3000 liquid chromatography with an Innoval ODS-2, 21.2 mm × 25 cm column, large column (Innoval ODS-2, 50.0 mm × 25 cm column). Single crystal X-ray diffraction data were collected on Rigaku Saturn70 diffractometer at 113(2) K (for compound 3t) with Mo-Kα radiation (λ = 0.71073 Å) and SCX-Mini diffractometer at 293(2) K (for compound 4kk and compound 4ss) with Mo-Kα radiation (λ = 0.71075 Å).

Notice: Reactions with AgF_2 supplied from Strem or Aldrich gave the best yields and a Teflon-lined screw cap was necessary when the reaction solvent was MeCN.

Experimental Data

Optimization of the reaction condition for the trifluoromethoxylation

Optimization of solvents on the reaction

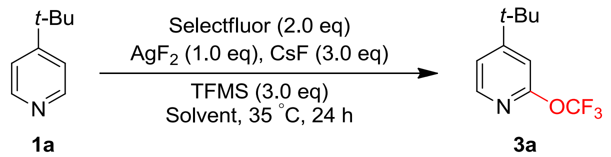

In a glove box, to a 2.00 mL sealed tube equipped with magnetic stir bar were added in sequence Selectfluor (35.4 mg, 0.10 mmol, 2.00 equiv), AgF_2 (7.2 mg, 0.050 mmol, 1.00 equiv), CsF (22.8 mg, 0.15 mmol, 3.00 equiv), solvent (1.0 mL), 4-(*tert*-butyl)pyridine (0.050 mmol, 1.00 equiv) and TFMS (25.0 μL, 0.15 mmol, 3.00 equiv). The mixture was stirred at 35 ºC for 24 hr. Yields based on **1a** were determined by ^{19}F NMR using trifluorotoluene as an internal standard.

Supplementary Table 1: Optimization of solvents on the reaction

Solvent (1.0 mL)	Yield [%] (^{19}F NMR)	Solvent (1.0 mL)	Yield [%] (^{19}F NMR)
DMC	62	1,2-Dichlorobenzene	23
DEC	55	THF	0
EA	49	DMF	0
DCM	42	Acetone	0
DCE	32	DMC (0.4mL)	56
MeCN	10	DMC (0.6mL)	58
1,4-dioxane	10	DMC (1.4mL)	60

Optimization of oxidants on the reaction

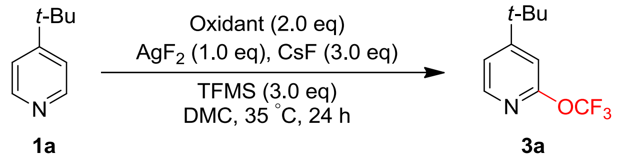

In a glove box, to a 2.00 mL sealed tube equipped with magnetic stir bar were added in sequence Oxidant (0.10 mmol, 2.00 equiv), AgF_2 (7.2 mg, 0.050 mmol, 1.00 equiv), CsF (22.8 mg, 0.15 mmol, 3.00 equiv), DMC (1.0 mL), 4-(*tert*-butyl)pyridine (0.050 mmol, 1.00 equiv) and TFMS (25.0 μL, 0.15 mmol, 3.00 equiv). The mixture was stirred at 35 ºC for 24 hr. Yields based on **1a** were determined by ^{19}F NMR using trifluorotoluene as an internal standard.

Supplementary Table 2: Optimization of oxidants on the reaction

Oxidant (2.0 eq)	Yield [%] (^{19}F NMR)	Oxidant (3.0 eq)	Yield [%] (^{19}F NMR)
Selectfluor	62	No	9

Selectfluor II	47	Oxone	1
Selectfluor·2OTf[2]	15	NSFI	0
$K_2S_2O_8$	44	$PhI(OAc)_2$	0
$Na_2S_2O_8$	2	Selectfluor (1.0 equiv)	48
TCCA	39	Selectfluor (1.5 equiv)	57
KIO_4	10	Selectfluor (2.5 equiv)	60

Optimization of silver salts on the reaction

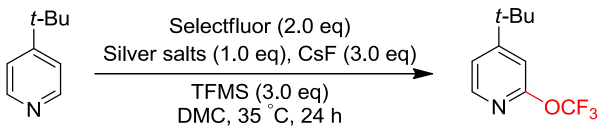

1a **3a**

In a glove box, to a 2.00 mL sealed tube equipped with magnetic stir bar were added in sequence Selectfluor (35.4 mg, 0.10 mmol, 2.00 equiv), Silver salts (0.050 mmol, 1.00 equiv), CsF (22.8 mg, 0.15 mmol, 3.00 equiv), DMC (1.0 mL), 4-(*tert*-butyl)pyridine (0.050 mmol, 1.00 equiv) and TFMS (25.0 µL, 0.15 mmol, 3.00 equiv). The mixture was stirred at 35 ºC for 24 hr. Yields based on **1a** were determined by ^{19}F NMR using trifluorotoluene as an internal standard.

Supplementary Table 3: Optimization of silver salts on the reaction

Silver salts (1.0 eq)	Yield [%] (^{19}F NMR)	Silver salts (1.0 eq)	Yield [%] (^{19}F NMR)
AgF_2	62	AgOTf	29
AgO	46	CF_3COOAg	20
AgF	53	Ag_2SO_4	21
Ag_2O	48	$AgNO_3$	6
Ag_2CO_3	50	AgCl	0
Ag_3PO_4	33	No	0
$AgBF_4$	42	AgF_2 (0.1 equiv)	9
PhCOOAg	48	AgF_2 (0.2 equiv)	18
$AgPF_6$	30	AgF_2 (0.3 equiv)	25
$AgClO_4$	46	AgF_2 (2.0 equiv)	46

Effect of fluorine sources on the reaction

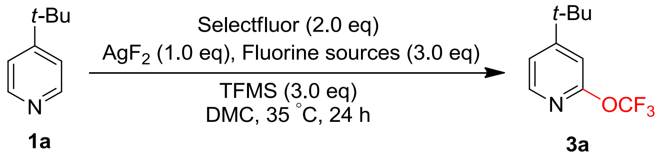

In a glove box, to a 2.00 mL sealed tube equipped with magnetic stir bar were added in sequence Selectfluor (35.4 mg, 0.10 mmol, 2.00 equiv), AgF_2 (7.2 mg, 0.050 mmol, 1.00 equiv), Fluorine source (0.15 mmol, 3.00 equiv), DMC (1.0 mL), 4-(*tert*-butyl)pyridine (0.050 mmol, 1.00 equiv) and TFMS (25.0 µL, 0.15 mmol, 3.00 equiv). The mixture was stirred at 35 ºC for 24 hr. Yields based on **1a** were determined by ^{19}F NMR using trifluorotoluene as an internal standard.

Supplementary Table 4: Effect of fluorine sources on the reaction

Fluorine sources (3.0 eq)	Yield [%] (^{19}F NMR)	Fluorine sources (3.0 eq)	Yield [%] (^{19}F NMR)
CsF	62	CuF_2	0
No CsF	10	CeF_3	8
KF	57	ZnF_2	8
NaF	11	$Et_3N\cdot HF$	0
RbF	25	TBAF	0
AgF	4	CsF (1.0 equiv)	29
CoF_3	9	CsF (2.0 equiv)	52
$NaHF_2$	10	CsF (4.0 equiv)	61

Effect of "OCF_3" sources on the reaction

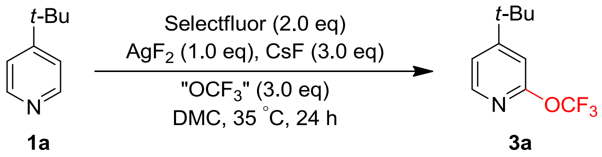

In a glove box, to a 2.00 mL sealed tube equipped with magnetic stir bar were added in sequence Selectfluor (35.4 mg, 0.10 mmol, 2.00 equiv), AgF_2 (7.2 mg, 0.050 mmol, 1.00 equiv), CsF (22.8 mg, 0.15 mmol, 3.00 equiv), DMC (1.0 mL), 4-(*tert*-butyl)pyridine (0.050 mmol, 1.00 equiv) and "OCF_3" source (0.15 mmol, 3.00 equiv). The mixture was stirred at 35 ºC for 24 hr. Yields based on **1a** were determined by ^{19}F NMR using trifluorotoluene as an internal standard.

Supplementary Table 5: Effect of "OCF_3" sources on the reaction

"OCF3" sources (3.0 eq)	Yield [%] (^{19}F NMR)	"OCF3" sources (3.0 eq)	Yield [%] (^{19}F NMR)
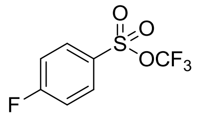	62 %	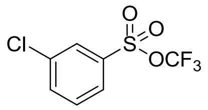	48 %
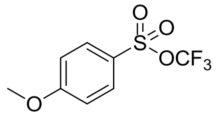	48 %	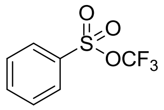	57 %
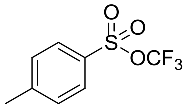	52 %	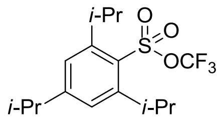	50 %
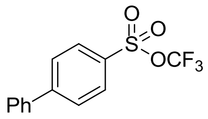	50 %	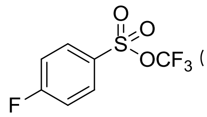 (1.0 eq)	39 %
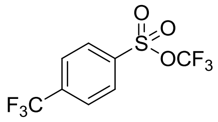	48 %	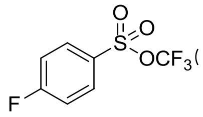 (2.0 eq)	48 %
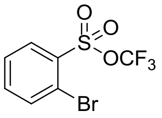	53 %	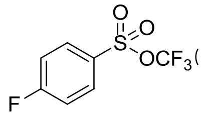 (4.0 eq)	62 %

Effect of temperature on the reaction

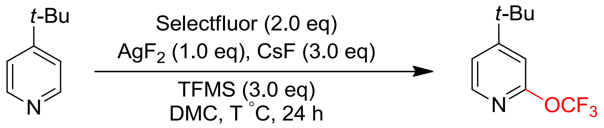

1a **3a**

In a glove box, to a 2.00 mL sealed tube equipped with magnetic stir bar were added in sequence Selectfluor (35.4 mg, 0.10 mmol, 2.00 equiv), AgF_2 (7.2 mg, 0.050 mmol, 1.00 equiv), CsF (22.8 mg, 0.15 mmol, 3.00 equiv), DMC (1.0 mL), 4-(*tert*-butyl)pyridine (0.050 mmol, 1.00 equiv) and TFMS (25.0 µL, 0.15 mmol, 3.00 equiv). The mixture was stirred at different temperatures for 24 hr. Yields based on **1a** were determined by ^{19}F NMR using trifluorotoluene as an internal standard.

Supplementary Table 6: Effect of different temperatures on the reaction

Temperature	Yield [%] (^{19}F NMR)
15 ºC	50
25 ºC	53

35 °C	62
45 °C	41
60 °C	32

Effect of atmospheres on the reaction

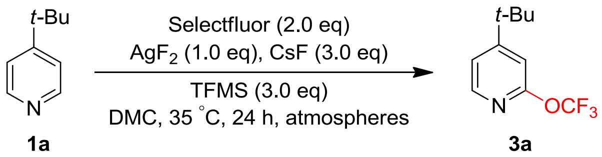

To a 2.00 mL sealed tube equipped with magnetic stir bar were added in sequence Selectfluor (35.4 mg, 0.10 mmol, 2.00 equiv), AgF_2 (7.2 mg, 0.050 mmol, 1.00 equiv), CsF (22.8 mg, 0.15 mmol, 3.00 equiv), DMC (1.0 mL), 4-(*tert*-butyl)pyridine (0.050 mmol, 1.00 equiv) and TFMS (25.0 µL, 0.15 mmol, 3.00 equiv). The mixture was stirred at 35 °C for 24 hr. Yields based on **1a** were determined by ^{19}F NMR using trifluorotoluene as an internal standard.

Supplementary Table 7: Effect of atmospheres on the reaction

Atmospheres	Yield [%] (^{19}F NMR)
N_2 (glovebox)	62
O_2	60
air	55
N_2 (with 2.0 eq H_2O)	0

Effect of sizes of sealed tube on the reaction

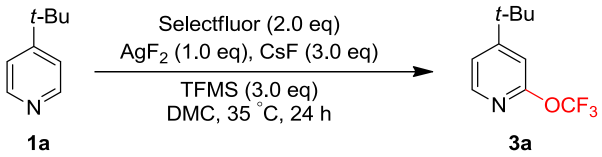

In a glove box, to different sizes of sealed tubes equipped with magnetic stir bar were added in sequence Selectfluor (35.4 mg, 0.10 mmol, 2.00 equiv), AgF_2 (7.2 mg, 0.050 mmol, 1.00 equiv), CsF (22.8 mg, 0.15 mmol, 3.00 equiv), DMC (1.0 mL), 4-(*tert*-butyl)pyridine (0.050 mmol, 1.00 equiv) and TFMS (25.0 µL, 0.15 mmol, 3.00 equiv). The mixture was stirred at 35 °C for 24 hr. Yields based on **1a** were determined by ^{19}F NMR using trifluorotoluene as an internal standard.

Supplementary Table 8: Effect of sizes of sealed tube on the reaction

Sizes of sealed tube	Yield [%] (^{19}F NMR)
2 mL	62
4 mL	52

8 mL	52

Effect of ligands on the reaction about aromatic compound

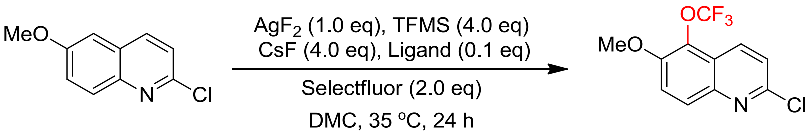

1jj **3jj**

In a glove box, to a 2.00 mL sealed tube equipped with magnetic stir bar were added in sequence Selectfluor (35.4 mg, 0.10 mmol, 2.00 equiv), AgF_2 (7.2 mg, 0.050 mmol, 1.00 equiv), CsF (22.8 mg, 0.15 mmol, 3.00 equiv), Ligand (0.005 mmol, 0.10 equiv), DMC (1.0 mL), 2-chloro-6-methoxyquinoline (9.7mg, 0.050 mmol, 1.00 equiv) and TFMS (25.0 µL, 0.15 mmol, 3.00 equiv). The mixture was stirred at 35 ºC for 24 hr. Yields based on **1jj** were determined by ^{19}F NMR using trifluorotoluene as an internal standard.

Supplementary Table 9: Effect of ligands on the reaction about aromatic compound

Ligand (0.1 eq)	Yield [%] (^{19}F NMR)	Ligand (0.1 eq)	Yield [%] (^{19}F NMR)
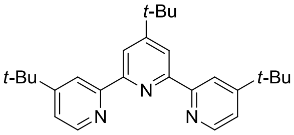	76	No	5
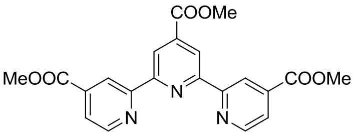	28	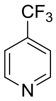	4
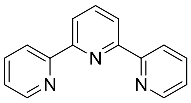	22	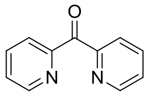	6
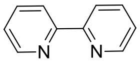	7	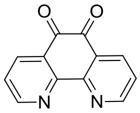	27
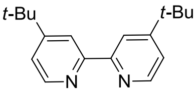	25	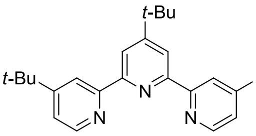 (0.05 eq)	59
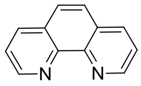	50	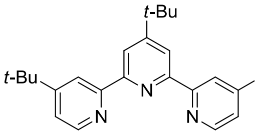 (0.3 eq)	58
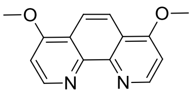	46	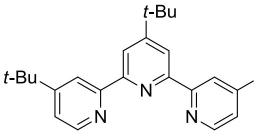 (1.0 eq)	47

Procedure for trifluoromethoxylation of arenes and heteroarenes

General procedure A :

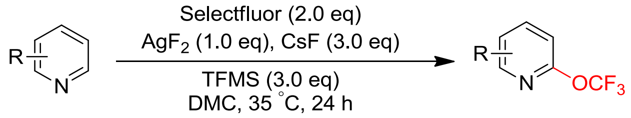

In a glove box, to a 15.0 mL sealed tube were added in sequence Selectfluor (354 mg, 1.00 mmol, 2.00 equiv), AgF_2 (72 mg, 0.50 mmol, 1.00 equiv), CsF (228 mg, 1.50 mmol, 3.00 equiv), 10.0 mL DMC, pyridines (0.50 mmol, 1.00 equiv) and TFMS (240 μL, 1.50 mmol, 3.00 equiv). The mixture was stirred at 35 ºC for 24 hr. After cooling to 23 °C, the reaction mixture was filtered through a short plug of silica gel eluting with approximately 25 mL of CH_2Cl_2 or EtOAc. The filtrate was concentrated, and the residue was purified by chromatography on silica gel.

General procedure B :

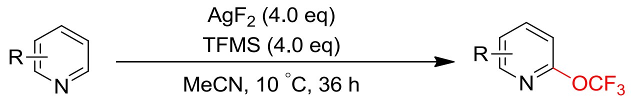

In a glove box, to a 2.0 mL sealed tube were added in sequence pyridines (0.50 mmol, 1.00 equiv), 0.5 mL MeCN (the solvent was pre-cooled to -30 °C) and TFMS (320 μL, 2.00 mmol, 4.00 equiv), then AgF_2 (290 mg, 2.0 mmol, 4.00 equiv) was added at once in one portion. The mixture was stirred at 10 ºC for 36 hr. After warming up to 23 °C, the reaction mixture was filtered through a short plug of silica gel eluting with approximately 25 mL of CH_2Cl_2 or EtOAc. The filtrate was concentrated, and the residue was purified by chromatography on silica gel.

General procedure C :

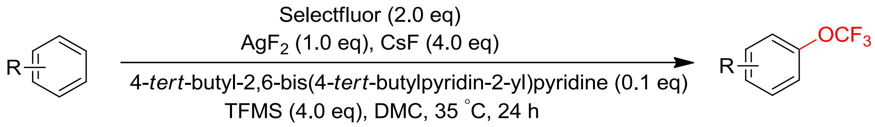

In a glove box, to a 15.0 mL sealed tube were added in sequence Selectfluor (354 mg, 1.0 mmol, 2.00 equiv) or $K_2S_2O_8$ (270 mg, 1.0 mmol, 2.00 equiv), AgF_2 (72 mg, 0.50 mmol, 1.00 equiv), CsF (304 mg, 2.00 mmol, 4.00 equiv), 4-*tert*-butyl-2,6-bis(4-*tert*-butylpyridin-2-yl)pyridine (20.0 mg, 0.05 mmol, 0.10 equiv), 10.0 mL DMC, Arenes or heteroarenes (0.50 mmol, 1.00 equiv) and TFMS (320 μL, 2.00 mmol, 4.00 equiv). The mixture was stirred at 35 ºC for 24 hr. After cooling to 23 °C, the reaction mixture was filtered through a short plug of silica gel eluting with approximately 25 mL of CH_2Cl_2 or EtOAc. The filtrate was concentrated, and the residue was purified by chromatography on silica gel.

General procedure D :

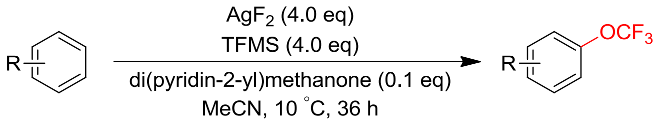

In a glove box, to a 2.0 mL sealed tube were added in sequence di(pyridin-2-yl)methanone (9.2 mg, 0.05 mmol, 0.10 equiv), AgF_2 (290 mg, 1.0 mmol, 4.00 equiv), 1.0 mL MeCN, arenes or heteroarenes (0.50 mmol, 1.00 equiv) and TFMS (320 µL, 2.00 mmol, 4.00 equiv). The mixture was stirred at 10 °C for 36 hr. After warming up to 23 °C, the reaction mixture was filtered through a short plug of silica gel eluting with approximately 25 mL of CH_2Cl_2 or EtOAc. The filtrate was concentrated, and the residue was purified by chromatography on silica gel.

Trifluoromethoxylation of arenes and heteroarenes (related to Experimental Procedures)-4-(tert-butyl)-2-(trifluoromethoxy)pyridine (3a)

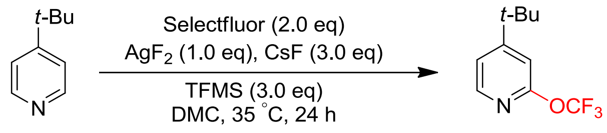

The reaction was performed according to the general procedure A using 4-(*tert*-butyl)pyridine (**1a**) (67.6 mg, 0.50 mmol) as the substrate. After 24 hr, the reaction mixture was filtered through a short plug of silica gel eluting with approximately 25 mL of CH_2Cl_2 and the filtrate was concentrated *in vacuo*. In order to remove 4-fluorobenzene-1-sulfonyl fluoride, the residue was sequentially added H_2O (20.0 mL), acetone (6.0 mL), $NaHCO_3$ (1.64 g, 19.5 mmol, 39.0 equiv) and Na_2SO_3 (1.91 g, 12.0 mmol, 24.0 equiv). Then the reaction mixture was stirred at 50°C for 4 hr, cooled to room temperature, extracted with CH_2Cl_2 (20.0 mL × 3), and washed with brine (20.0 mL). The combined organic layer was dried over $MgSO_4$ and concentrated *in vacuo*. The residue was purified by flash column chromatography on silica eluting with hexanes/ EtOAc 50:1 (v/v) to afford 63 mg 4-(*tert*-butyl)-2-(trifluoromethoxy)pyridine (**3a**) as a colourless liquid (58 % yield). R_f = 0.4 (*n*-hexane/EtOAc 50:1 (v/v)). ^{1}H NMR (400 MHz, $CDCl_3$) δ 8.23 (d, J = 5.4 Hz, 1H), 7.21 (d, J = 6.6 Hz, 1H), 6.97 (s, 1H), 1.32 (s, 9H). ^{13}C NMR (101 MHz, $CDCl_3$) δ 165.4, 157.4, 147.6, 120.3 (q, J = 272.7 Hz), 119.4, 110.0, 35.2, 30.5. ^{19}F NMR (376 MHz, $CDCl_3$) δ -56.32 (s, 3F). Mass Spectrometry: HRMS-ESI (m/z): Calcd for $C_{10}H_{13}F_3NO$ $[M + H]^+$, 220.0944. Found, 220.0945.

4-Methoxy-2-(trifluoromethoxy)pyridine (3b)

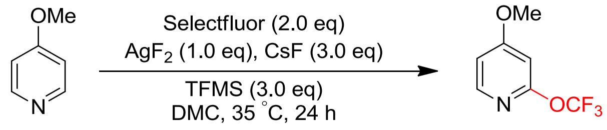

The reaction was performed according to the general procedure A using 4-methoxypyridine (**1b**) (54.5 mg, 0.50 mmol) as the substrate. After 24 hr, the reaction mixture was filtered through a short plug of silica gel eluting with approximately 25 mL of CH_2Cl_2 and the filtrate was concentrated *in vacuo*. In order to remove 4-fluorobenzene-1-sulfonyl fluoride, the residue was sequentially added H_2O (20.0 mL), acetone (6.0 mL), $NaHCO_3$ (1.64 g, 19.5 mmol, 39.0 equiv) and Na_2SO_3 (1.91 g, 12.0 mmol, 24.0 equiv). Then the reaction mixture was stirred at 50°C for 4 hr, cooled to room temperature, extracted with CH_2Cl_2 (20.0 mL × 3), and washed with brine (20.0 mL). The combined organic layer was dried over $MgSO_4$ and concentrated *in vacuo*. The residue

was purified by flash column chromatography on silica eluting with hexanes/ CH_2Cl_2 50:1 (v/v) to afford 57.9 mg 4-methoxy-2-(trifluoromethoxy)pyridine (**3b**) as a colourless liquid (60 % yield). R_f= 0.5 (*n*-hexane/CH_2Cl_2 50:1 (v/v)). ^{1}H NMR (400 MHz, $CDCl_3$) δ 8.13 (d, *J* = 5.8 Hz, 1H), 6.76 (dd, *J* = 5.8, 2.1 Hz, 1H), 6.48 (d, *J* = 2.0 Hz, 1H), 3.87 (s, 3H). ^{13}C NMR (101 MHz, $CDCl_3$) δ 169.0, 158.4, 148.6, 120.0 (q, *J* = 262.6 Hz), 109.6, 98.1, 55.9. ^{19}F NMR (376 MHz, $CDCl_3$) δ -56.26 (s, 3F). Mass Spectrometry: HRMS-ESI (m/z): Calcd for $C_7H_7F_3NO_2$ $[M + H]^+$, 194.0423. Found, 194.0428.

4-Phenoxy-2-(trifluoromethoxy)pyridine (3c)

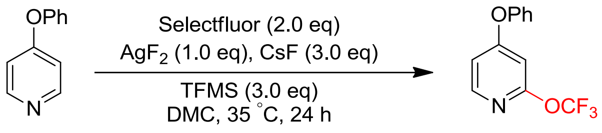

The reaction was performed according to the general procedure A using 4-phenoxypyridine (**1c**) (85.5 mg, 0.50 mmol) as the substrate. After 24 hr, the reaction mixture was filtered through a short plug of silica gel eluting with approximately 25 mL of CH_2Cl_2 and the filtrate was concentrated *in vacuo*. In order to remove 4-fluorobenzene-1-sulfonyl fluoride, the residue was sequentially added H_2O (20.0 mL), acetone (6.0 mL), $NaHCO_3$ (1.64 g, 19.5 mmol, 39.0 equiv) and Na_2SO_3 (1.91 g, 12.0 mmol, 24.0 equiv). Then the reaction mixture was stirred at 50°C for 4 hr, cooled to room temperature, extracted with CH_2Cl_2 (20.0 mL × 3), and washed with brine (20.0 mL). The combined organic layer was dried over $MgSO_4$ and concentrated *in vacuo*. The residue was purified by flash column chromatography on silica eluting with hexanes/ EtOAc 50:1 (v/v) to afford 66.3 mg 4-phenoxy-2-(trifluoromethoxy)pyridine (**3c**) as a white solid (52 % yield). R_f= 0.5 (*n*-hexane/EtOAc 50:1 (v/v)). ^{1}H NMR (400 MHz, $CDCl_3$) δ 8.16 (d, *J* = 5.8 Hz, 1H), 7.45 (t, *J* = 7.9 Hz, 2H), 7.29 (t, *J* = 7.4 Hz, 1H), 7.10 (d, *J* = 7.7 Hz, 2H), 6.77 (dd, *J* = 5.8, 2.1 Hz, 1H), 6.46 (d, *J* = 2.0 Hz, 1H). ^{13}C NMR (101 MHz, $CDCl_3$) δ 168.1, 158.5, 153.6, 149.0, 130.5, 126.2, 121.0, 120.2 (q, *J* = 262.6 Hz), 111.0, 100.5. ^{19}F NMR (376 MHz, DMSO) δ -56.71 (s, 3F). Mass Spectrometry: HRMS-ESI (m/z): Calcd for $C_{12}H_9F_3NO_2$ $[M + H]^+$, 256.0580. Found, 256.0583.

4-Phenyl-2-(trifluoromethoxy)pyridine (3d)

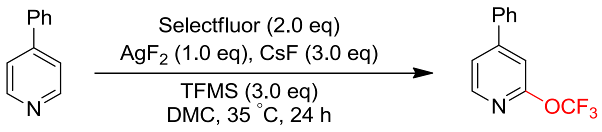

The reaction was performed according to the general procedure A using 4-phenylpyridine (**1d**) (77.6 mg, 0.50 mmol) as the substrate. After 24 hr, the reaction mixture was filtered through a short plug of silica gel eluting with approximately 25 mL of CH_2Cl_2 and the filtrate was concentrated *in vacuo*. In order to remove 4-fluorobenzene-1-sulfonyl fluoride, the residue was sequentially added H_2O (20.0 mL), acetone (6.0 mL), $NaHCO_3$ (1.64 g, 19.5 mmol, 39.0 equiv) and Na_2SO_3 (1.91 g, 12.0 mmol, 24.0 equiv). Then the reaction mixture was stirred at 50°C for 4 hr, cooled to room temperature, extracted with CH_2Cl_2 (20.0 mL × 3), and washed with brine (20.0 mL). The combined organic layer was dried over $MgSO_4$ and concentrated *in vacuo*. The residue

was purified by flash column chromatography on silica eluting with hexanes/ EtOAc 100:1 (v/v) to afford 63.3 mg 4-phenyl-2-(trifluoromethoxy)pyridine (**3d**) as a colourless liquid (53 % yield). R_f= 0.5 (*n*-hexane/EtOAc 100:1 (v/v)). ^{1}H NMR (400 MHz, $CDCl_3$) δ 8.37 (d, *J* = 5.2 Hz, 1H), 7.62 (dd, *J* = 7.8, 1.7 Hz, 2H), 7.55 – 7.47 (m, 3H), 7.44 (dd, *J* = 5.2, 1.4 Hz, 1H), 7.21 (s, 1H). ^{13}C NMR (101 MHz, $CDCl_3$) δ 157.7, 153.3, 148.3, 137.1, 129.9, 129.4, 127.2, 120.3 (q, *J* = 262.6 Hz), 120.2, 110.8. ^{19}F NMR (376 MHz, DMSO) δ -56.78 (s, 3F). Mass Spectrometry: HRMS-ESI (m/z): Calcd for $C_{12}H_9F_3NO$ $[M + H]^+$, 240.0631. Found, 240.0632.

4-(Pentan-3-yl)-2-(trifluoromethoxy)pyridine (1e)

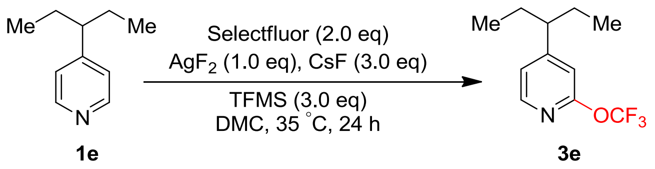

The reaction was performed according to the general procedure A using 4-(pentan-3-yl)pyridine (**1e**) (75 mg, 0.50 mmol) as the substrate. After 24 hr, the reaction mixture was filtered through a short plug of silica gel eluting with approximately 25 mL of CH_2Cl_2 and the filtrate was concentrated *in vacuo*. In order to remove 4-fluorobenzene-1-sulfonyl fluoride, the residue were sequentially added H_2O (20.0 mL), acetone (6.0 mL), $NaHCO_3$ (1.64 g, 19.5 mmol, 39.0 equiv) and Na_2SO_3 (1.91 g, 12.0 mmol, 24.0 equiv). Then the reaction mixture was stirred at 50°C for 4 hr, cooled to room temperature, extracted with CH_2Cl_2 (20.0 mL × 3), and washed with brine (20.0 mL). The combined organic layer was dried over $MgSO_4$ and concentrated *in vacuo*. The combined organic layer was dried over $MgSO_4$ and concentrated *in vacuo*. The residue was purified by flash column chromatography on silica eluting with hexanes/ EtOAc 10:1 (v/v) to afford 59.4 mg 4-(pentan-3-yl)-2-(trifluoromethoxy)pyridine (**3e**) as a colourless liquid (51 % yield).

R_f= 0.8 (*n*-hexane/EtOAc 10:1 (v/v)). ^{1}H NMR (400 MHz, $CDCl_3$) δ 8.20 (d, *J* = 5.2 Hz, 1H), 7.00 (d, *J* = 5.1 Hz, 1H), 6.77 (s, 1H), 2.47 – 2.23 (m, 1H), 1.71 (tt, *J* = 14.8, 7.4 Hz, 2H), 1.54 (tt, *J* = 14.6, 7.4 Hz, 2H), 0.77 (t, *J* = 7.4 Hz, 6H). ^{13}C NMR (101 MHz, $CDCl_3$) δ 160.5, 157.3, 147.6, 121.8, 120.3 (q, *J* = 262.6 Hz), 112.3, 49.3, 28.6, 12.0. ^{19}F NMR (376 MHz, $CDCl_3$) δ -56.77 (s, 3F). Mass Spectrometry: HRMS-ESI (m/z): Calcd for $C_{11}H_{15}F_3NO$ $[M + H]^+$, 234.1100. Found, 234.1102.

3-((2-(Trifluoromethoxy)pyridin-4-yl)oxy)propan-1-ol (3f)

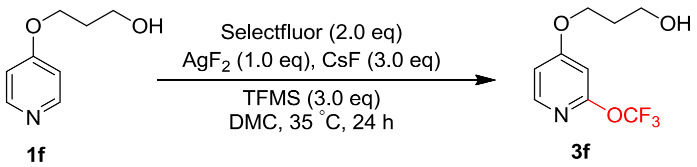

The reaction was performed according to the general procedure A using 3-(pyridin-4-yloxy)propan-1-ol[4] (**1f**) (76.6 mg, 0.50 mmol) in 10.0 mL DMC. After 24 hr, the reaction mixture was filtered through a short plug of silica gel eluting with approximately 25 mL

of EtOAc and the filtrate was concentrated *in vacuo*. In order to remove 4-fluorobenzene-1-sulfonyl fluoride, the residue was sequentially added H_2O (20.0 mL), acetone (6.0 mL), $NaHCO_3$ (1.64 g, 19.5 mmol, 39.0 equiv) and Na_2SO_3 (1.91 g, 12.0 mmol, 24.0 equiv). Then the reaction mixture was stirred at 50°C for 4 hr, cooled to room temperature, extracted with CH_2Cl_2 (20.0 mL × 3), and washed with brine (20.0 mL). The combined organic layer was dried over $MgSO_4$ and concentrated *in vacuo*. The residue was purified by flash column chromatography on silica eluting with hexanes/ EtOAc 10:1 (v/v) to afford 56.9 mg 3-((2-(trifluoromethoxy)pyridin-4-yl)oxy)propan-1-ol (**3f**) as a light yellow liquid (48 % yield).
R_f = 0.5 (*n*-hexane/EtOAc 1:1 (v/v)). ^{1}H NMR (400 MHz, $CDCl_3$) δ 8.11 (d, *J* = 5.8 Hz, 1H), 6.75 (dd, *J* = 5.9, 2.2 Hz, 1H), 6.49 (d, *J* = 2.1 Hz, 1H), 4.17 (t, *J* = 6.1 Hz, 2H), 3.84 (t, *J* = 6.0 Hz, 2H), 2.06 (p, *J* = 6.0 Hz, 2H), 1.82 (s, 1H). ^{13}C NMR (101 MHz, $CDCl_3$) δ 168.2, 158.4, 148.6, 120.2 (d, *J* = 262.6 Hz), 109.9, 98.6, 65.7, 59.3, 31.6. ^{19}F NMR (376 MHz, $CDCl_3$) δ -56.31(s, 3F). Mass Spectrometry: HRMS-EI (m/z): Calcd for $C_9H_{10}F_3NO_3$ $[M]^+$, 237.0613. Found, 237.0610.

2-(Trifluoromethoxy)isonicotinonitrile (3g)

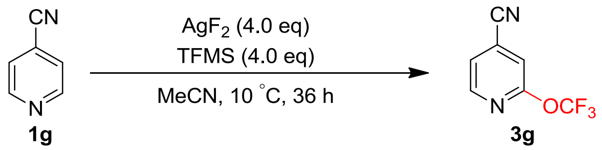

The reaction was performed according to the general procedure B using isonicotinonitrile (**1g**) (52 mg, 0.50 mmol) as the substrate. After 36 hr, the reaction mixture was filtered through a short plug of silica gel eluting with approximately 25 mL of CH_2Cl_2 and the filtrate was concentrated *in vacuo*. The residue was purified by flash column chromatography on silica eluting with hexanes/ EtOAc 40:1 (v/v) to afford 65.8 mg 2-(trifluoromethoxy)isonicotinonitrile (**3g**) as a colourless liquid (70 % yield).
R_f = 0.3 (*n*-hexane/EtOAc 40:1 (v/v)). ^{1}H NMR (400 MHz, $CDCl_3$) δ 8.52 (d, *J* = 5.7 Hz, 1H), 7.47 (dd, *J* = 5.1, 1.2 Hz, 1H), 7.26 (s, 1H). ^{13}C NMR (101 MHz, $CDCl_3$) δ 157.3 – 157.1 (m), 149.4, 124.4, 123.2, 120.0 (q, *J* = 262.6 Hz), 115.5 – 115.3 (m), 115.2. ^{19}F NMR (376 MHz, $CDCl_3$) δ -56.90 (s, 3F). Mass Spectrometry: HRMS-ESI (m/z): Calcd for $C_7H_4F_3N_2O$ $[M + H]^+$, 189.0270. Found, 189.0273.

2-(Trifluoromethoxy)pyridine (3h)

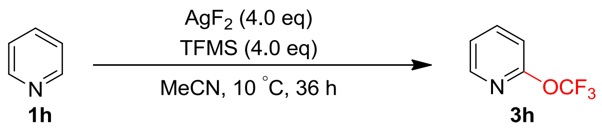

The reaction was performed according to the general procedure B using pyridine (**1h**) (39.6 mg, 0.50 mmol) as the substrate, 0.25 mL MeCN. After stirred at 10 °C for 36 hr, an internal standard $PhCF_3$ (60 µL, 0.5 mmol, 1.0 equiv) was added to the reaction vial. The reaction mixture (32 % yield by ^{19}F NMR) was purified by preparative HPLC (8 mL/min, detector UV λmax 210 nm, $MeCN/H_2O$ = 30/70 (0 min), $MeCN/H_2O$ = 70:30 (25 min), $MeCN/H_2O$ = 90:10 (30 min), $MeCN/H_2O$ = 100:0 (40 min)) to afford 2-(trifluoromethoxy)pyridine (**3h**) (retention time 30.4

min). As a volatile compound, the desired product was extracted with 1 mL $CDCl_3$ and then directly characterized.
^1H NMR (400 MHz, $CDCl_3$) δ 8.01 (d, J = 5.6 Hz, 1H), 7.60 (t, J = 6.8 Hz, 1H), 7.10 – 6.96 (m, 1H), 6.80 (d, J = 7.9 Hz, 1H). ^{19}F NMR (376 MHz, $CDCl_3$) δ -56.78 (s, 3F). Mass Spectrometry: HRMS-EI (m/z): Calcd for C6H4F3NO $[M]^+$, 163.0245. Found, 163.0239.

3-Phenyl-2-(trifluoromethoxy)pyridine (3i) and 5-phenyl-2-(trifluoromethoxy) Pyridine (iso-3i)

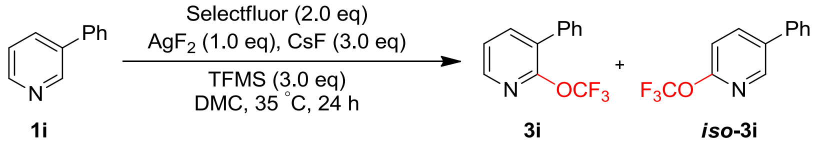

The reaction was performed according to the general procedure A using 3-phenylpyridine (**1i**) (77.6 mg, 0.50 mmol) as the substrate. After 24 hr, the reaction mixture was filtered through a short plug of silica gel eluting with approximately 25 mL of CH_2Cl_2 and the filtrate was concentrated *in vacuo*. In order to remove 4-fluorobenzene-1-sulfonyl fluoride, the residue was sequentially added H_2O (20.0 mL), acetone (6.0 mL), $NaHCO_3$ (1.64 g, 19.5 mmol, 39.0 equiv) and Na_2SO_3 (1.91 g, 12.0 mmol, 24.0 equiv). Then the reaction mixture was stirred at 50°C for 4 hr, cooled to room temperature, extracted with CH_2Cl_2 (20.0 mL × 3), and washed with brine (20.0 mL). The combined organic layer was dried over $MgSO_4$ and concentrated *in vacuo*. The residue was purified by preparative HPLC (8 mL/min, detector UV λmax 210 nm, MeCN/H_2O = 50/50 (0 min), MeCN/H_2O = 70:30 (25 min), MeCN/H_2O = 80:20 (30 min), MeCN/H_2O = 100:0 (40 min)) to afford 43 mg 3-phenyl-2-(trifluoromethoxy)pyridine (**3i**) (retention time 31.5 min) as a colourless liquid (36 % yield) and 9.5 mg 5-phenyl-2-(trifluoromethoxy)pyridine (***iso*-3i**) (retention time 34.6 min) as a colourless liquid (8 % yield).
Spectral data for **3i**:
R_f = 0.3 (*n*-hexane/CH_2Cl_2 5:1 (v/v)). ^1H NMR (400 MHz, $CDCl_3$) δ 8.31 (dd, J = 4.8, 1.9 Hz, 1H), 7.81 (dd, J = 7.5, 1.9 Hz, 1H), 7.52 – 7.39 (m, 5H), 7.31 (dd, J = 7.5, 4.8 Hz, 1H). ^{13}C NMR (101 MHz, $CDCl_3$) δ 153.7, 146.3, 140.8, 134.8, 129.2, 128.7, 128.5, 127.1, 122.2, 120.4 (q, J = 262.6 Hz). ^{19}F NMR (376 MHz, $CDCl_3$) δ -55.79 (s, 3F). Mass Spectrometry: HRMS-ESI (m/z): Calcd for $C_{12}H_9F_3NO$ $[M + H]^+$, 240.0631. Found, 240.0633.
Spectral data for ***iso*-3i**:
R_f = 0.3 (*n*-hexane/CH_2Cl_2 5:1 (v/v)). ^1H NMR (400 MHz, $CDCl_3$) δ 8.54 (d, J = 2.5 Hz, 1H), 7.98 (dd, J = 8.4, 2.6 Hz, 1H), 7.55 (d, J = 8.1 Hz, 2H), 7.49 (t, J = 7.9 Hz, 2H), 7.43 (t, J = 7.2 Hz, 1H), 7.10 (d, J = 8.4 Hz, 1H). ^{13}C NMR (101 MHz, $CDCl_3$) δ 156.1, 146.2, 138.9, 136.7, 135.5, 129.4, 128.5, 127.1, 120.4 (q, J = 262.6 Hz), 113.1. ^{19}F NMR (376 MHz, $CDCl_3$) δ -56.46 (s, 3F). Mass Spectrometry: HRMS-EI (m/z): Calcd for $C_{12}H_8F_3NO$ $[M]^+$, 239.0558. Found, 239.0547.

2-(Trifluoromethoxy)nicotinonitrile (3j) and 6-(trifluoromethoxy)nicotinonitrile (iso-3j)

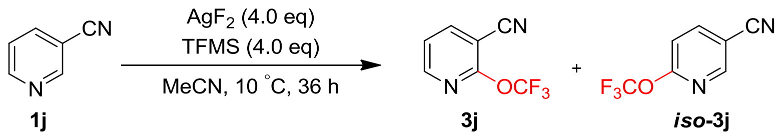

The reaction was performed according to the general procedure B using nicotinonitrile (**1j**) (52 mg, 0.50 mmol) as the substrate. After 36 hr, the reaction mixture was filtered through a short plug of silica gel eluting with approximately 25 mL of CH_2Cl_2 and the filtrate was concentrated *in vacuo*. The residue was purified by flash column chromatography on silica eluting with hexanes/ EtOAc 8:1 (v/v) to afford 66 mg 2-(trifluoromethoxy)nicotinonitrile (**3j**) as a colourless liquid (71 % yield) and 3.8 mg 6-(trifluoromethoxy)nicotinonitrile (***iso*-3j**) as a colourless liquid (4 % yield).

Spectral data for **3j**:

R_f = 0.5 (n-hexane/EtOAc 8:1 (v/v)).^{1}H NMR (400 MHz, CDC13) δ 8.51 (dd, J = 4.9, 1.8 Hz, 1H), 8.09 (dd, J = 7.7, 1.9 Hz, 1H), 7.37 (dd, J = 7.7, 5.0 Hz, 1H). ^{13}C NMR (101 MHz, CDC13) δ 156.6 – 156.8 (m), 151.4, 144.0, 121.5, 119.8 (q, J = 262.6 Hz), 113.0, 99.4. ^{19}F NMR (376 MHz, CDC13) δ -56.80 (s, 3F). The spectroscopic data is in agreement with the literature[3]. Spectral data for ***iso*-3j**:

R_f = 0.6 (*n*-hexane/EtOAc 8:1 (v/v)). ^{1}H NMR (400 MHz, $CDCl_3$) δ 8.62 (d, *J* = 2.2 Hz, 1H), 8.06 (dd, *J* = 8.6, 2.3 Hz, 1H), 7.11 (d, *J* = 8.6 Hz, 1H). ^{13}C NMR (101 MHz, $CDCl_3$) δ 158.8, 151.8, 143.4, 119.8 (q, *J* = 262.6 Hz), 115.8, 113.2, 108.0. ^{19}F NMR (376 MHz, $CDCl_3$) δ -56.92 (s, 3F). Mass Spectrometry: HRMS-ESI (m/z): Calcd for $C_7H_4F_3N_2O$ $[M + H]^+$, 189.0270. Found, 189.0270.

3-Nitro-2-(trifluoromethoxy)pyridine (3k) and 5-nitro-2-(trifluoromethoxy)pyri -dine (iso-3k)

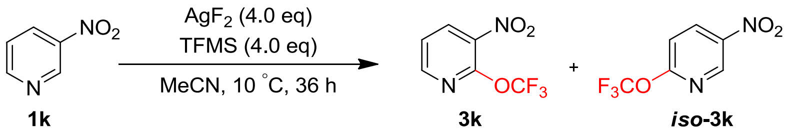

The reaction was performed according to the general procedure B using 3-nitropyridine (**1k**) (62 mg, 0.50 mmol) as the substrate. After 36 hr, an internal standard $PhCF_3$ (60 μL, 0.5 mmol, 1.0 equiv) was added to the reaction vial. The reaction mixture (84 % yield with isomers 2-OCF_3 : 6-OCF_3 = 27:1 by ^{19}F NMR) was filtered through a short plug of silica gel eluting with approximately 25 mL of CH_2Cl_2 and the filtrate was concentrated *in vacuo*. The residue was purified by flash column chromatography on silica eluting with hexanes/ EtOAc 8:1 (v/v) to afford 84.2 mg major isomer 3-nitro-2-(trifluoromethoxy)pyridine (**3k**) as a colourless liquid (81 % yield).

Spectral data for **3k**:

R_f = 0.3 (*n*-hexane/EtOAc 8:1 (v/v)). ^{1}H NMR (400 MHz, $CDCl_3$) δ 8.55 (d, *J* = 4.1 Hz, 1H), 8.41 (d, *J* = 7.9 Hz, 1H), 7.45 (dd, *J* = 7.9, 4.8 Hz, 1H). ^{13}C NMR (101 MHz, $CDCl_3$) δ 151.5, 148.6 – 148.4 (m), 136.0, 135.1, 122.2, 120.0 (q, *J* = 262.6 Hz). ^{19}F NMR (376 MHz, $CDCl_3$) δ -56.12 (s, 3F). Mass Spectrometry: HRMS-EI (m/z): Calcd for $C_6H_3F_3N_2O_3$ $[M]^+$, 208.0096. Found, 208.0090.

3-(2-Methyl-1,3-dioxolan-2-yl)-2-(trifluoromethoxy)pyridine (3l) and 5-(2-methyl-1,3-dioxolan-2-yl)-2-(trifluoromethoxy)pyridine (iso-3l)

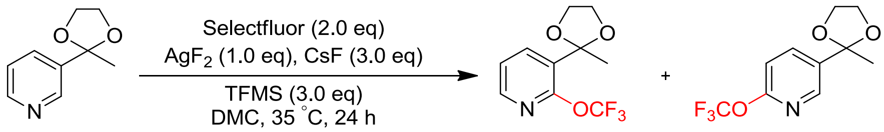

1l **3l** ***iso*-3l**

The reaction was performed according to the general procedure A using 3-(2-methyl-1,3-dioxolan-2-yl)pyridine (**1l**) (82.6 mg, 0.50 mmol) as the substrate. After 24 hr, the reaction mixture was filtered through a short plug of silica gel eluting with approximately 25 mL of CH_2Cl_2 and the filtrate was concentrated *in vacuo*. In order to remove 4-fluorobenzene-1-sulfonyl fluoride, the residue was sequentially added H_2O (20.0 mL), acetone (6.0 mL), $NaHCO_3$ (1.64 g, 19.5 mmol, 39.0 equiv) and Na_2SO_3 (1.91 g, 12.0 mmol, 24.0 equiv). Then the reaction mixture was stirred at 50°C for 4 hr, cooled to room temperature, extracted with CH_2Cl_2 (20.0 mL × 3), and washed with brine (20.0 mL). The combined organic layer was dried over $MgSO_4$ and concentrated *in vacuo*. The residue was purified by preparative HPLC (7.5 mL/min, detector UV λmax 210 nm, MeCN/H_2O = 50/50 (0 min), MeCN/H_2O = 70:30 (25 min), MeCN/H_2O = 75:25 (30 min)) to afford 32.4 mg 3-(2-methyl-1,3-dioxolan-2-yl)-2-(trifluoromethoxy)pyridine (**3l**) (retention time 27.6 min) as a colourless liquid (26 % yield) and 23.7 mg 5-(2-methyl-1,3-dioxolan-2-yl)-2-(trifluorometh-oxy)pyridine (***iso*-3l**) (retention time 28.8 min) as a colourless liquid (19 % yield).

Spectral data for **3l**:

R_f= 0.3 (*n*-hexane/CH_2Cl_2 30:1 (v/v)). ^{1}H NMR (400 MHz, $CDCl_3$) δ 8.25 (d, J = 3.4 Hz, 1H), 7.96 (dd, J = 7.5, 1.4 Hz, 1H), 7.20 (dd, J = 7.5, 4.9 Hz, 1H), 4.08 (t, J = 7.0 Hz, 2H), 3.79 (t, J = 7.0 Hz, 2H), 1.74 (s, 3H). ^{13}C NMR (101 MHz, $CDCl_3$) δ 154.1, 147.2, 137.7, 127.6, 121. 7, 120.4 (q, J = 262.6 Hz), 106.8, 64.9, 25.5. ^{19}F NMR (376 MHz, $CDCl_3$) δ -55.27 (s, 3F). Mass Spectrometry: HRMS-ESI (m/z): Calcd for $C_{10}H_{11}F_3NO_3$ $[M + H]^+$, 250.0686. Found, 250.0686.

Spectral data for ***iso*-3l:**

R_f= 0.3 (*n*-hexane/CH_2Cl_2 30:1 (v/v)). ^{1}H NMR (400 MHz, $CDCl_3$) δ 8.43 (d, J = 2.4 Hz, 1H), 7.87 (dd, J = 8.4, 2.5 Hz, 1H), 6.98 (d, J = 8.4 Hz, 1H), 4.07 (td, J = 6.2, 4.3 Hz, 2H), 3.78 (td, J = 6.1, 4.2 Hz, 2H), 1.65 (s, 3H). ^{13}C NMR (101 MHz, $CDCl_3$) δ 156.5, 145.5, 137.9, 137.5, 120.3 (q, J = 272.7 Hz), 112.6, 107.5, 64.9, 27. 8. ^{19}F NMR (376 MHz, $CDCl_3$) δ -56.54 (s, 3F). Mass Spectrometry: HRMS-ESI (m/z): Calcd for $C_{10}H_{11}F_3NO_3$ $[M + H]^+$, 250.0686. Found, 250.0690.

2-(Trifluoromethoxy)-3,5-bis(trifluoromethyl)pyridine (3m)

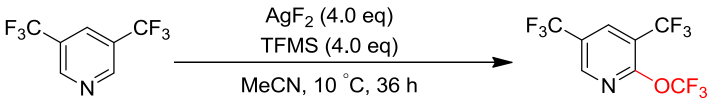

1m **3m**

The reaction was performed according to the general procedure B using 3,5-bis(trifluoromethyl)pyridine (**1m**) (107.6 mg, 0.50 mmol) as the substrate and 2.0 mL MeCN in 4.0 ml sealed tube. After 36 hr, an internal standard $PhCF_3$ (60 μL, 0.5 mmol, 1.0 equiv) was added to the reaction vial. The reaction mixture (71 % yield by ^{19}F NMR) was purified by preparative HPLC (8 mL/min, detector UV λmax 210 nm, MeCN/H_2O = 70/30 (0 min), MeCN/H_2O = 80:20 (25 min), MeCN/H_2O = 90:10 (30 min)) to afford 2-(trifluoromethoxy)-3,5-bis(trifluoromethyl)pyridine (**3m**) (retention time 22.5 min). As a

volatile compound, the desired product was extracted with 1 mL $CDCl_3$ and then directly characterized.

^1H NMR (400 MHz, $CDCl_3/CH_3CN$) δ 8.56 (s, 1H), 8.18 (s, 1H). ^{13}C NMR (101 MHz, $CDCl_3/CH_3CN$) δ 154.3 – 154.5 (m), 148.0 – 148.2 (m), 135.6 – 135.8 (m), 124.0 (q, J = 40.4 Hz), 121.9 (q, J = 272.7 Hz), 120.6 (q, J = 272.7 Hz), 119.0 (q, J = 272.7 Hz), 114.6 (q, J = 40.4 Hz). ^{19}F NMR (376 MHz, $CDCl_3/CH_3CN$) δ -56.61 (s, 3F), -62.26 (s, 3F), -63.65 (s, 3F) . Mass Spectrometry: HRMS-ESI (m/z): Calcd for $C_8H_3F_9NO$ $[M + H]^+$, 300.0065. Found, 300.0065.

3,5-Dimethyl-2-(trifluoromethoxy)pyridine (3n)

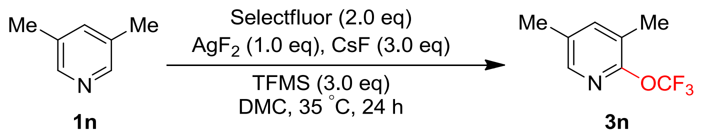

1n **3n**

The reaction was performed according to the general procedure A using 3,5-dimethylpyridine (**1n**) (53.6 mg, 0.50 mmol) as the substrate. After 24 hr, the reaction mixture was filtered through a short plug of silica gel eluting with approximately 25 mL of CH_2Cl_2 and the filtrate was concentrated *in vacuo*. In order to remove 4-fluorobenzene-1-sulfonyl fluoride, the residue was sequentially added H_2O (20.0 mL), acetone (6.0 mL), $NaHCO_3$ (1.64 g, 19.5 mmol, 39.0 equiv) and Na_2SO_3 (1.91 g, 12.0 mmol, 24.0 equiv). Then the reaction mixture was stirred at 50°C for 4 hr, cooled to room temperature, extracted with CH_2Cl_2 (20.0 mL × 3), and washed with brine (20.0 mL). The combined organic layer was dried over $MgSO_4$ and concentrated *in vacuo*. The residue was purified by flash column chromatography on silica eluting with hexanes/ EtOAc 100:1 (v/v) to afford 47.8 mg 3,5-dimethyl-2-(trifluoromethoxy)pyridine (**3n**) as a colourless liquid (50 % yield).

R_f = 0.4 (*n*-hexane/EtOAc 100:1 (v/v)). ^1H NMR (400 MHz, $CDCl_3$) δ 7.94 (s, 1H), 7.40 (s, 1H), 2.29 (s, 3H), 2.25 (s, 3H). ^{13}C NMR (101 MHz, $CDCl_3$) δ 153.6 – 153.8 (m), 144.8, 141.8, 131. 7, 122.6, 120.5 (q, J = 262.6 Hz), 17.59, 15.56. ^{19}F NMR (376 MHz, $CDCl_3$) δ -56.01 (s, 3F). Mass Spectrometry: HRMS-EI (m/z): Calcd for $C_8H_8F_3NO$ $[M]^+$, 191.0558. Found, 191.0551.

5-Methyl-3-phenyl-2-(trifluoromethoxy)pyridine (3o) and 3-methyl-5-phenyl-2- (trifluoro-methoxy)pyridine (iso-3o)

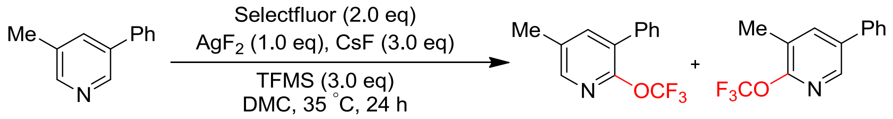

1o **3o** ***iso*-3o**

The reaction was performed according to the general procedure A using 3-methyl-5-phenylpyridine (**1o**) (84.6 mg, 0.50 mmol) as the substrate. After 24 hr, the reaction mixture was filtered through a short plug of silica gel eluting with approximately 25 mL of CH_2Cl_2 and the filtrate was concentrated *in vacuo*. In order to remove 4-fluorobenzene-1-sulfonyl fluoride, the residue was sequentially added H_2O (20.0 mL), acetone (6.0 mL), $NaHCO_3$ (1.64 g, 19.5 mmol, 39.0 equiv) and Na_2SO_3 (1.91 g, 12.0 mmol, 24.0 equiv). Then the reaction mixture was stirred at 50°C for 4 hr, cooled to room temperature, extracted with CH_2Cl_2 (20.0 mL × 3), and

washed with brine (20.0 mL). The combined organic layer was dried over $MgSO_4$ and concentrated *in vacuo*. The residue was purified by preparative HPLC (8 mL/min, detector UV λmax 210 nm, MeCN/H_2O = 50/50 (0 min), MeCN/H_2O = 70:30 (20 min), MeCN/H_2O = 80:20 (30 min)) to afford 40.5 mg 5-methyl-3-phenyl-2-(trifluoromethoxy)pyridine (**3o**) (retention time 23.5 min) as a colourless liquid (32 % yield) and 10.1 mg 3-methyl-5-phenyl-2-(trifluoromethoxy)pyridine (***iso*-3o**) (retention time 25.2 min) as a colourless liquid (8 % yield).

Spectral data for **3o**:

R_f = 0.2 (*n*-hexane/EtOAc 200:1 (v/v)). ^{1}H NMR (400 MHz, $CDCl_3$) δ 8.11 (d, J = 1.8 Hz, 1H), 7.61 (d, J = 2.0 Hz, 1H), 7.51 – 7.38 (m, 5H), 2.38 (s, 3H). ^{13}C NMR (101 MHz, $CDCl_3$) δ 151.9, 146.2, 141.5, 134.9, 132.0, 129.2, 128.6, 128.4, 126.6, 120.4 (q, J = 262.6 Hz), 17.7. ^{19}F NMR (376 MHz, DMSO) δ -55.76 (s, 3F). Mass Spectrometry: HRMS-EI (m/z): Calcd for $C_{13}H_{10}F_3NO$ $[M]^+$, 253.0714. Found, 253.0711.

Spectral data for ***iso*-3o:**

R_f = 0.2 (*n*-hexane/EtOAc 200:1 (v/v)). ^{1}H NMR (400 MHz, $CDCl_3$) δ 8.34 (s, 1H), 7.78 (s, 1H), 7.54 (d, J = 7.3 Hz, 2H), 7.47 (t, J = 7.4 Hz, 2H), 7.41 (t, J = 7.1 Hz, 1H), 2.36 (s, 3H). ^{13}C NMR (101 MHz, $CDCl_3$) δ 154.9, 143.1, 139.6, 136.9, 135.5, 129.3, 128.3, 127.2, 122.9, 120.5 (q, J = 262.6 Hz), 15.92. ^{19}F NMR (376 MHz, $CDCl_3$) δ -55.98 (s, 3F). Mass Spectrometry: HRMS-EI (m/z): Calcd for $C_{13}H_{10}F_3NO$ $[M]^+$, 253.0714. Found, 253.0710.

Dimethyl 6-(trifluoromethoxy)pyridine-2,5-dicarboxylate (3p)

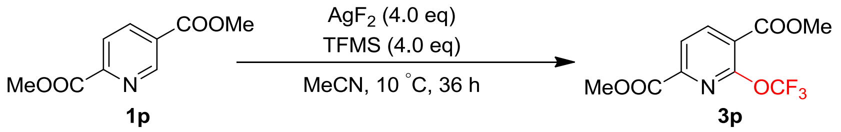

The reaction was performed according to the general procedure B using dimethyl pyridine-2,5-dicarboxylate (**1p**) (97.6 mg, 0.50 mmol) as the substrate, 0.25 mL MeCN. After 36 hr, the reaction mixture was filtered through a short plug of silica gel eluting with approximately 25 mL of EtOAc and the filtrate was concentrated *in vacuo*. The residue was purified by flash column chromatography on silica eluting with hexanes/ EtOAc 2:1 (v/v) to afford 106 mg dimethyl 6-(trifluoromethoxy)pyridine-2,5-dicarboxylate (**3p**) as a white solid (76 % yield).

R_f = 0.3 (*n*-hexane/EtOAc 2:1 (v/v)). ^{1}H NMR (400 MHz, $CDCl_3$) δ 8.39 (d, J = 7.8 Hz, 1H), 8.03 (d, J = 7.8 Hz, 1H), 3.94 (s, 3H), 3.91 (s, 3H). ^{13}C NMR (101 MHz, $CDCl_3$) δ 163.7, 163.2, 153.8 – 154.0 (m), 148.5, 143.3, 122.8, 122.0 (q, J = 262.6 Hz), 119.9, 53.2, 53.1. ^{19}F NMR (376 MHz, $CDCl_3$) δ -56.17 (s, 3F). Mass Spectrometry: HRMS-ESI (m/z): Calcd for $C_{10}H_9F_3NO_5$ $[M + H]^+$, 280.0427. Found, 280.0432.

Methyl 5-bromo-3-fluoro-6-(trifluoromethoxy)picolinate (3q)

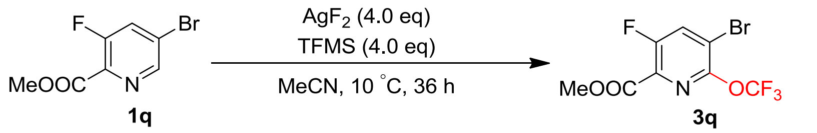

The reaction was performed according to the general procedure B using methyl 5-bromo-3-fluoropicolinate (**1s**) (117 mg, 0.50 mmol) as the substrate. After 36 hr, the reaction mixture was filtered through a short plug of silica gel eluting with approximately 25 mL of EtOAc and the filtrate was concentrated *in vacuo*. The residue was purified by flash column chromatography on silica eluting with hexanes/ EtOAc 10:1 (v/v) to afford 46.1 mg methyl 5-bromo-3-fluoro-6-(trifluoromethoxy)picolinate (**3q**) as a white solid (29 % yield).
R_f = 0.5 (*n*-hexane/EtOAc 10:1 (v/v)). ^{1}H NMR (400 MHz, $CDCl_3$) δ 7.92 (d, *J* = 8.1 Hz, 1H), 3.97 (s, 3H). ^{13}C NMR (101 MHz, $CDCl_3$) δ 161.8 (d, *J* = 6.4 Hz), 156.5 (d, *J* = 273.9 Hz), 148.2, 133.5 (d, *J* = 24.6 Hz), 132.2 (d, *J* = 12.0 Hz), 120.0 (q, *J* = 262.6 Hz), 113.5 – 113.7 (m), 53.3. ^{19}F NMR (376 MHz, $CDCl_3$) δ -57.11 (s, 3F), -118.13 – 118.18 (m, 1F). Mass Spectrometry: HRMS-EI (m/z): Calcd for $C_8H_4BrF_4NO_3$ $[M]^+$, 316.9311. Found, 316.9305.

3,5-Difluoro-2-(trifluoromethoxy)isonicotinonitrile (3r)

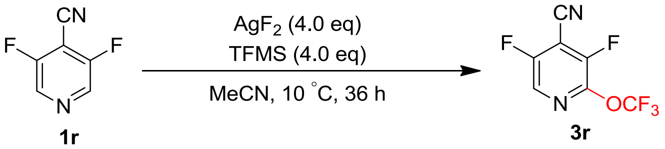

The reaction was performed according to the general procedure B using 3,5-difluoroisonicotinonitrile (**1r**) (70 mg, 0.50 mmol) as the substrate, 1.0 mL MeCN. After 36 hr, an internal standard $PhCF_3$ (60 μL, 0.5 mmol, 1.0 equiv) was added to the reaction vial. The reaction mixture (68 % yield by ^{19}F NMR) was purified by preparative HPLC (8 mL/min, detector UV λmax 210 nm, $MeCN/H_2O$ = 50/50 (0 min), $MeCN/H_2O$ = 70:30 (25 min), $MeCN/H_2O$ = 90:10 (30 min), $MeCN/H_2O$ = 100:0 (40 min)) to afford 3,5-difluoro-2-(trifluoromethoxy)isonicotin-
onitrile (**3t**) (retention time 28.5 min). As a volatile compound, the desired product was extracted with 1 mL $CDCl_3$ and then directly characterized.
^{1}H NMR (400 MHz, $CDCl_3/CH_3CN$) δ 8.18 (s, 1H). ^{13}C NMR (101 MHz, $CDCl_3/CH_3CN$) δ 155.7 (dd, *J* = 271.2, 2.9 Hz), 146.9 (d, *J* = 281.8 Hz), 140.4 (d, *J* = 10.9 Hz), 130.6 (dd, *J* = 24.4, 7.0 Hz), 119.2 (q, *J* = 262.6 Hz), 106.1 (s), 102.3 (dd, *J* = 18.4, 14.1 Hz). ^{19}F NMR (376 MHz, $CDCl_3/CH_3CN$) δ -57.05 (s), -121.51 (s), -124.17 (s). Mass Spectrometry: HRMS-EI (m/z): Calcd for $C_7HF_5N_2O$ $[M]^+$, 224.0009. Found, 224.0000.

6-Nitro-2-(trifluoromethoxy)quinoline (3s)

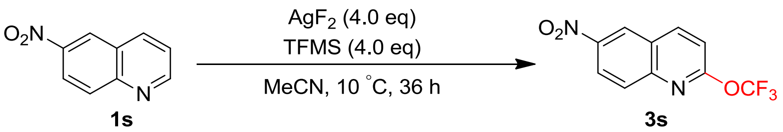

The reaction was performed according to the general procedure B using 6-nitroquinoline (**1s**) (87.1 mg, 0.50 mmol) as the substrate. After 36 hr, the reaction mixture was filtered through a short plug of silica gel eluting with approximately 25 mL of EtOAc and the filtrate was concentrated *in vacuo*. The residue was purified by flash column chromatography on silica eluting with hexanes/ EtOAc 10:1 (v/v) to afford 36.1 mg 6-nitro-2-(trifluoromethoxy)quinol-

one (**3s**) as a white solid (28 % yield).
R_f = 0.5 (*n*-hexane/EtOAc 10:1 (v/v)). ^{1}H NMR (400 MHz, $CDCl_3$) δ 8.78 (d, *J* = 2.5 Hz, 1H), 8.48 (dd, *J* = 9.2, 2.5 Hz, 1H), 8.42 (d, *J* = 8.8 Hz, 1H), 8.09 (d, *J* = 9.2 Hz, 1H), 7.24 (d, *J* = 8.8 Hz, 1H). ^{13}C NMR (101 MHz, $CDCl_3$) δ 157.3,148.5,145.6, 142.4, 130.3, 125.4, 124.2, 120.0 (q, *J* = 262.6 Hz),114.4, 114.3. ^{19}F NMR (376 MHz, $CDCl_3$) δ -56.83 (s, 3F). Mass Spectrometry: HRMS-EI (m/z): Calcd for $C_{10}H_5F_3N_2O_3$ $[M]^+$, 258.0252. Found, 258.0242.

2,4,6-Triphenyl-2'-(trifluoromethoxy)-[1,4'-bipyridin]-1-ium tetrafluoroborate (3t)

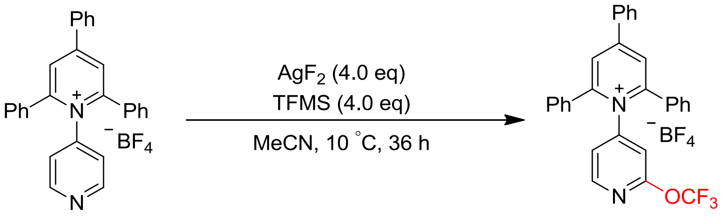

The reaction was performed according to the general procedure B using 2,4,6-triphenyl-[1,4'-bipyridin]-1-ium tetrafluoroborate[5] (**1t**) (118.0 mg, 0.25 mmol, 1.0 eq) as the substrate and 3.0 mL MeCN in 8.0 ml sealed tube. After stirred at rt for 15 hr, the reaction mixture was purified by preparative HPLC (8 mL/min, detector UV λmax 210 nm, MeCN/H_2O = 40/60 (0 min), MeCN/H_2O = 70:30 (30 min), MeCN/H_2O = 90:10 (35 min)) to afford 82 mg 2,4,6-triphenyl-2'-(trifluoromethoxy)-[1,4'-bipyridin]-1-ium tetrafluoroborate (**3t**) (retention time 23.5 min) as a light yellow solid (59 % yield).
^{1}H NMR (400 MHz, $CDCl_3$) δ 7.98 (d, *J* = 5.4 Hz, 1H), 7.74 (s, 2H), 7.60 (d, *J* = 7.7 Hz, 2H), 7.53 – 7.42 (m, 5H), 7.35 (d, *J* = 5.4 Hz, 2H), 7.31 (m, 3H), 7.28 – 7.20 (m, 4H), 7.15 (s, 1H). ^{13}C NMR (101 MHz, $CDCl_3$) δ 158.5, 156.8, 155.7, 149.6, 148.8, 134.7, 132.3, 132.2, 130.7, 129.8, 129.6, 128.7, 128.6, 126.4, 122.1, 119.7 (q, *J* = 262.6 Hz), 113.8. ^{19}F NMR (376 MHz, $CDCl_3$) δ -57.26 (s, 3F), -152.37 (s, 4F). Mass Spectrometry: HRMS-ESI (m/z): Calcd for $C_{29}H_{20}F_3N_2O^+$ $[M – BF_4]^+$, 469.1522. Found, 469.1526.

(Trifluoromethyl)benzene

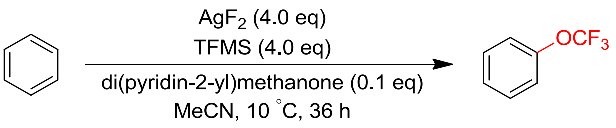

The reaction was performed according to the general procedure D using benzene (39 mg, 0.50 mmol) as the substrate. After 36 hr, an internal standard $PhCF_3$ (60 μL, 0.5 mmol, 1.0 equiv) was added to the reaction vial. The ^{19}FNMR yield of the volatile (trifluoromethoxy)benzene (**3x**) was determined by comparing the integration of the ^{19}F NMR resonance of (trifluoromethoxy)benzene (-58.20 ppm) with that of (trifluoromethyl)benzene (-62.80 ppm). (11% ^{19}F NMR Yield). The identity of the product was further confirmed by GCMS analysis, where the product peak was observed at 2.638 min, which was matched with with the authentic sample.

NMR Spectroscopy: ^{19}F NMR (376 MHz, MeCN) δ -58.20.

3x was a known compound and spectral data match the reported literature values. [6]

TIC and Mass spectrum for **3x** was listed as follows:

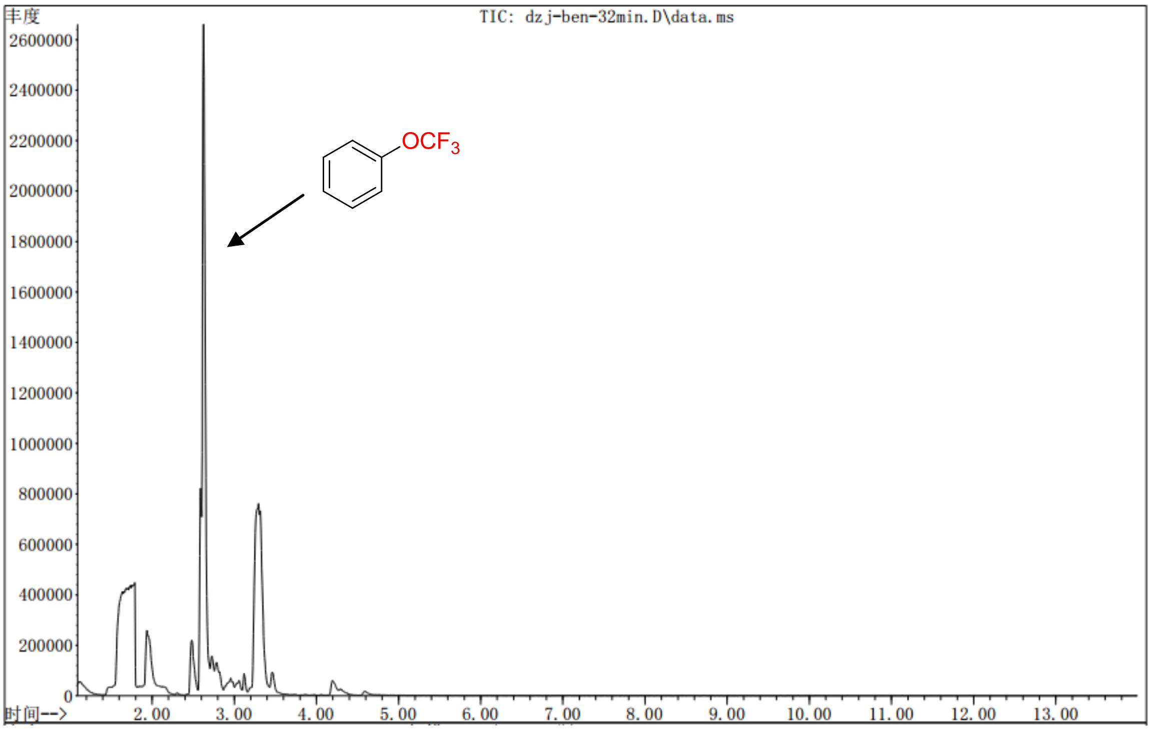

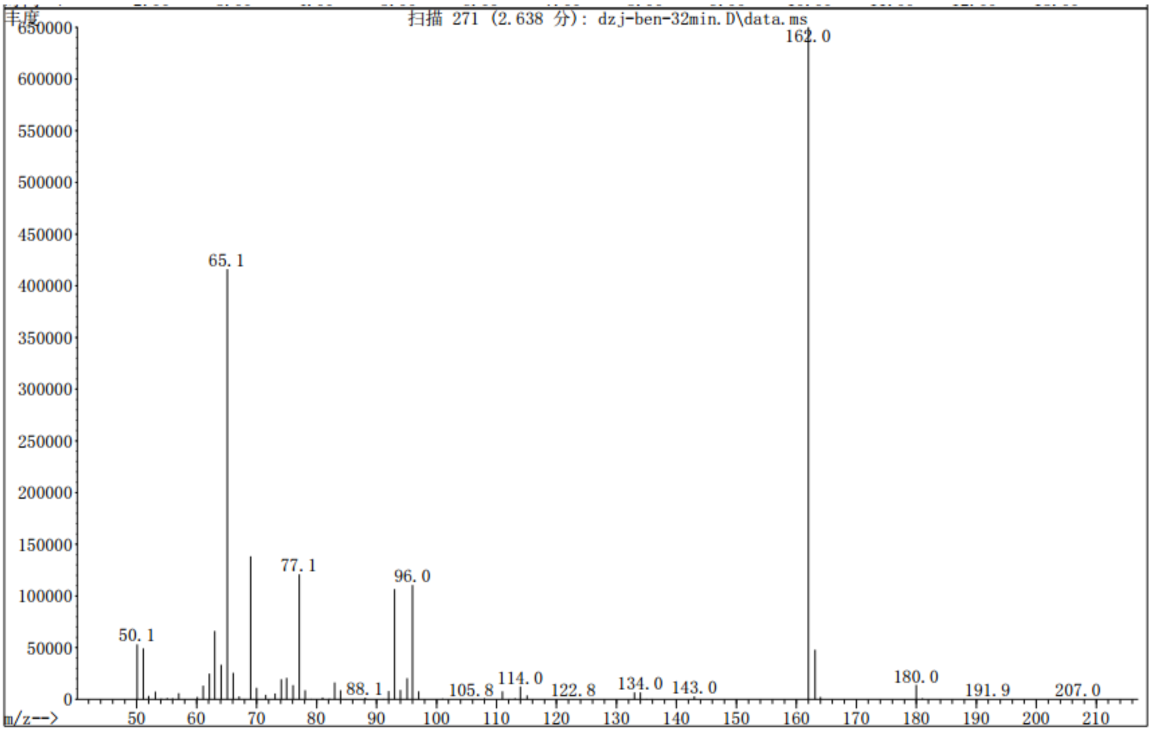

TIC and Mass spectrum for authentic sample **3x** was attached:

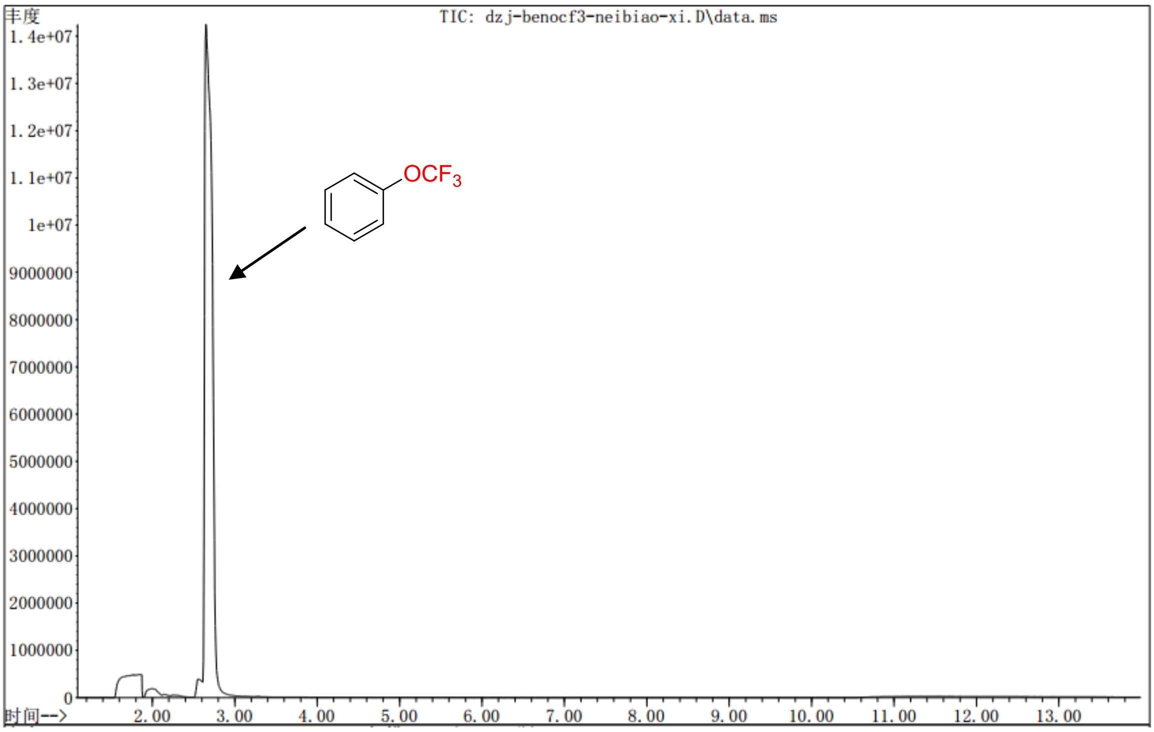

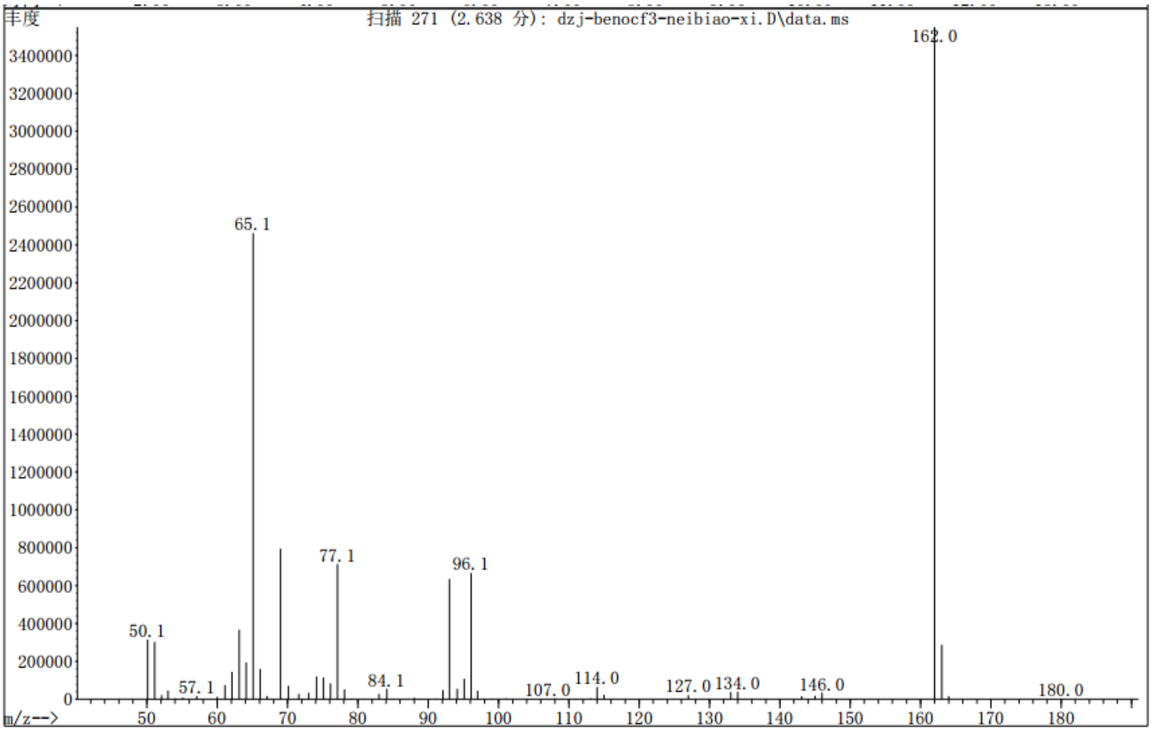

1-(*tert*-Butyl)-4-(trifluoromethoxy)benzene (3u) and 1-(*tert*-butyl)-2-(trifluorome thoxy) benzene (iso-3u)

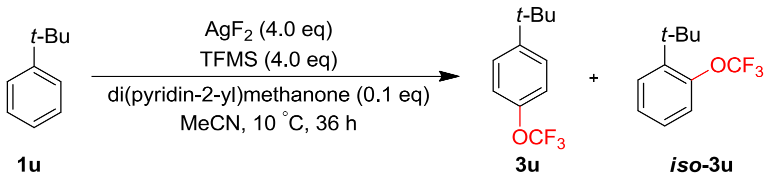

The reaction was performed according to the general procedure D using *tert*-butylbenzene (**1u**) (67.1 mg, 0.50 mmol) as the substrate. After 36 hr, an internal standard $PhCF_3$ (60 µL, 0.5 mmol, 1.0 equiv) was added to the reaction vial. The reaction mixture (59 % yield with isomers **3u** : ***iso*-3u** = 2.9:1 by ^{19}F NMR) was filtered through a short plug of silica gel eluting with approximately 25 mL of CH_2Cl_2 and the filtrate was concentrated *in vacuo*. The residue was purified by preparative HPLC (8 mL/min, detector UV λmax 210 nm, $MeCN/H_2O$ = 80/20 (0 min), $MeCN/H_2O$ = 90:10 (25 min), $MeCN/H_2O$ = 100:0 (30 min)) to afford a mixture of **3u** and ***iso*-3u** (retention time 24.6 min). As a volatile compound, the desired product was extracted with 1 mL $CDCl_3$ and then directly characterized.

Spectral data for a mixture of **3u** and ***iso*-3u**:

^1H NMR (400 MHz, $CDCl_3$) δ 7.41 (d, J = 8.8 Hz, 2H), 7.35 – 7.31 (m, 0.35H), 7.24 (s, 0.22H), 7.15 (d, J = 8.6 Hz, 2H), 7.06 (d, J = 4.7 Hz, 0.2H), 1.34 (s, 10.87H). ^{13}C NMR (101 MHz, $CDCl_3$) δ 153.9, 145.0, 149.4, 147.2, 129.4, 126.8, 123.9, 120.8 (d, J = 252.5 Hz), 120.6, 118.4, 117.9, 35.0, 34.7, 31.5, 31.3. ^{19}F NMR (376 MHz, $CDCl_3$) δ -57.72 (s, 0.63F), -57.92 (s, 3F). Mass Spectrometry: HRMS-EI (m/z): Calcd for $C_{11}H_{13}F_3O$ $[M]^+$, 218.0918. Found, 218.0913. The spectroscopic data of **3y** is in agreement with the literature. [7]

1-Phenoxy-4-(trifluoromethoxy)benzene (3v) and 1-phenoxy-2-(trifluoromethoxy) benzene (iso-3v)

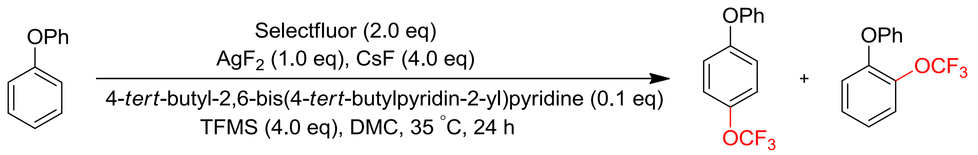

1v **3v** ***iso*-3v**

The reaction was performed according to the general procedure C using diphenyl ether (**1v**) (85.1 mg, 0.50 mmol) as the substrate. After 24 hr, the reaction mixture was filtered through a short plug of silica gel eluting with approximately 25 mL of CH_2Cl_2 and the filtrate was concentrated *in vacuo*. In order to remove 4-fluorobenzene-1-sulfonyl fluoride, the residue was sequentially added H_2O (20.0 mL), acetone (6.0 mL), $NaHCO_3$ (1.64 g, 19.5 mmol, 39.0 equiv) and Na_2SO_3 (1.91 g, 12.0 mmol, 24.0 equiv). Then the reaction mixture was stirred at 50°C for 4 hr, cooled to room temperature, extracted with CH_2Cl_2 (20.0 mL × 3), and washed with brine (20.0 mL). The combined organic layer was dried over $MgSO_4$ and concentrated *in vacuo*. The residue was purified by preparative HPLC (10 mL/min, detector UV λmax 210 nm, $MeCN/H_2O$ = 60/40 (0 min), $MeCN/H_2O$ = 70:30 (25 min), $MeCN/H_2O$ = 80:20 (30 min) , $MeCN/H_2O$ = 90:10 (35 min) , $MeCN/H_2O$ = 100:0 (40 min)) to afford 35.6 mg 1-phenoxy-4-(trifluoromethoxy)benzene (**3v**) (retention time 38.5 min) as a colourless liquid (28 % yield) and 12.7 mg 1-phenoxy-2-(trifluoromethoxy)benzene (***iso*-3v**) (retention time 20.3 min) as a colourless liquid (10 % yield).

Spectral data for **3v**:

R_f = 0.6 (*n*-hexane), ^1H NMR (400 MHz, $CDCl_3$) δ 7.41 – 7.34 (m, 2H), 7.19 (d, J = 9.0 Hz, 2H), 7.15 (t, J = 7.4 Hz, 1H), 7.02 (m, 4H). ^{13}C NMR (101 MHz, $CDCl_3$) δ 156.9, 156.0, 144.6, 130.1, 124.0, 122.7, 120.7 (q, J = 252.5 Hz), 119.7, 119.3. ^{19}F NMR (376 MHz, $CDCl_3$) δ -58.53 (s, 3F). Mass Spectrometry: HRMS-EI (m/z): Calcd for $C_{13}H_9F_3O_2$ $[M]^+$, 254.0555. Found, 254.0553.

Spectral data for ***iso*-3v**:

R$_f$ = 0.6 (*n*-hexane), ^{1}H NMR (400 MHz, CDCl$_3$) δ 7.39 – 7.31 (m, 3H), 7.27 – 7.20 (m, 1H), 7.17 – 7.09 (m, 2H), 7.04 – 6.97 (m, 3H). ^{13}C NMR (101 MHz, CDCl$_3$) δ 156.8, 149.5, 140.2, 123.0, 128.1, 124.0, 123.9, 123.5, 120.7 (q, *J* = 262.6Hz), 120.6, 118.7. ^{19}F NMR (376 MHz, CDCl$_3$) δ -58.40 (s, 3F). Mass Spectrometry: HRMS-EI (m/z): Calcd for $C_{13}H_9F_3O_2$ [M]$^+$, 254.0555. Found, 254.0550.

1-Phenyl-2-(trifluoromethoxy)naphthalene (3w)

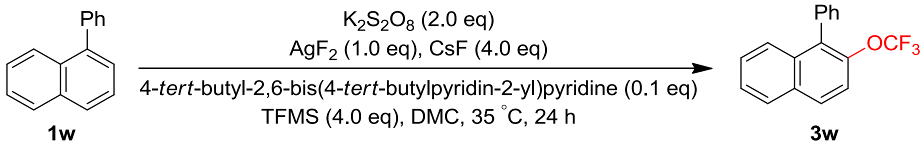

1w **3w**

The reaction was performed according to the general procedure C using 1-phenylnaphthalene (**1w**) (102.1 mg, 0.50 mmol), $K_2S_2O_8$ (270 mg, 1. 0 mmol, 2.00 equiv). After 24 hr, the reaction mixture was filtered through a short plug of silica gel eluting with approximately 25 mL of CH_2Cl_2 and the filtrate was concentrated *in vacuo*. The residue was purified by flash column chromatography on silica eluting with hexanes to afford 64.9 mg 1-phenyl-2-(trifluoromethox-y)naphthalene (**3w**) as a colorless liquid (45 % yield).

R$_f$ = 0.7 (*n*-hexane). ^{1}H NMR (400 MHz, CDCl$_3$) δ 8.20 (d, *J* = 8.3 Hz, 1H), 7.87 (d, *J* = 8.5 Hz, 1H), 7.57 – 7.49 (m, 1H), 7.47 – 7.35 (m, 7H), 7.32 (d, *J* = 7.9 Hz, 1H). ^{13}C NMR (101 MHz, CDCl$_3$) δ 144.8, 139.9, 139.5, 133.0, 130.2, 128.5, 127.7, 127.3, 127.1, 127.0, 126. 5, 126.3, 121.80, 121.2 (q, *J* = 262.6 Hz), 116.1. ^{19}F NMR (376 MHz, CDCl$_3$) δ -57.67 (s, 3F). Mass Spectrometry: HRMS-EI (m/z): Calcd for $C_{17}H_{11}F_3O$ [M]$^+$, 288.0762. Found, 288.0755.

4-(Trifluoromethoxy)-1,1'-biphenyl (3x) and 4,4'-bis(trifluoromethoxy)- 1, 1' - biphenyl (3x')

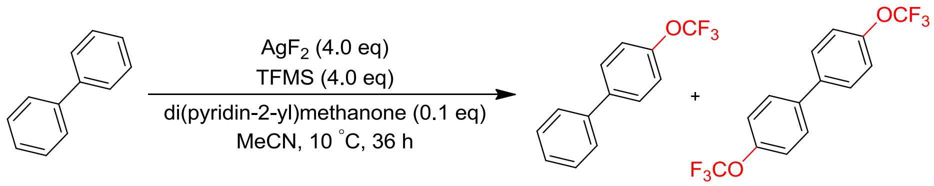

1x **3x** **3x'**

The reaction was performed according to the general procedure D using 1,1'-biphenyl (**1x**) (77.1 mg, 0.50 mmol) as the substrate, 2.0 mL MeCN in 4.0 mL sealed tube. After 36 hr, the reaction mixture was filtered through a short plug of silica gel eluting with approximately 25 mL of CH_2Cl_2 and the filtrate was concentrated *in vacuo*. In order to remove 4-fluorobenzene-1-sulfonyl fluoride, the residue was sequentially added H_2O (20.0 mL), acetone (6.0 mL), $NaHCO_3$ (1.64 g, 19.5 mmol, 39.0 equiv) and Na_2SO_3 (1.91 g, 12.0 mmol, 24.0 equiv). Then the reaction mixture was stirred at 50°C for 4 hr, cooled to room temperature, extracted with CH_2Cl_2 (20.0 mL × 3), and washed with brine (20.0 mL). The combined organic layer was dried over $MgSO_4$ and concentrated *in vacuo*. The residue was purified by preparative HPLC (8 mL/min, detector UV λmax 210 nm, MeCN/H_2O = 80/20 (0 min), MeCN/H_2O = 90:10 (30 min), MeCN/H_2O = 100:0 (40 min)) to afford 92.9 mg 4-(trifluoromethoxy)-1,1'-biphenyl (**3x**) (retention time 35.4 min) as a

white solid (78 % yield) and 8 mg 4,4'-bis(trifluoromethoxy)-1,1'-biphenyl (**3x'**) (retention time 40.3 min) as a white solid (5 % yield)..

Spectral data for **3x**:

R_f = 0.8 (*n*-hexane), ^{1}H NMR (400 MHz, $CDCl_3$) δ 7.63 – 7.55 (m, 4H), 7.47 (t, *J* = 7.5 Hz, 2H), 7.39 (t, *J* = 7.3 Hz, 1H), 7.30 (d, *J* = 8.2 Hz, 2H). ^{13}C NMR (101 MHz, $CDCl_3$) δ 148.8, 140.1, 140.0, 129.0, 128.6, 127.8, 127.3, 121.37, 120.7 (q, *J* = 262.6 Hz). ^{19}F NMR (376 MHz, $CDCl_3$) δ -58.19 (s, 3F). The spectroscopic data is in agreement with the literature.[8]

Spectral data for **3x'**:

R_f = 0.8 (*n*-hexane), ^{1}H NMR (400 MHz, $CDCl_3$) δ 7.59 – 7.54 (m, 4H), 7.30 (d, *J* = 8.0 Hz, 4H). ^{19}F NMR (376 MHz, $CDCl_3$) δ -58.26 (s, 3F). Mass Spectrometry: HRMS-EI (m/z): Calcd for $C_{14}H_8F_6O_2$ $[M]^+$, 322.0428. Found, 322.0416.

4-(*tert*-Butyl)-4'-(trifluoromethoxy)-1,1'-biphenyl (3y) and 4'-(*tert*-butyl)-3-(trifluoromethoxy)-1,1'-biphenyl (iso-3y)

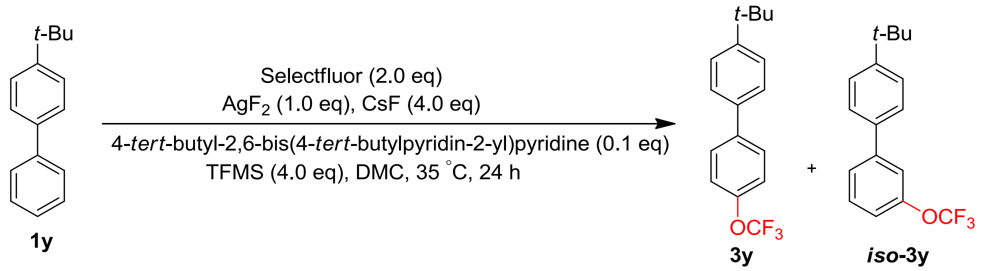

The reaction was performed according to the general procedure C using 4-(tert-butyl)-1,1'-biphenyl (**1y**) (105 mg, 0.50 mmol) as the substrate. After 24 hr, the reaction mixture (78 % yield with isomers **3y : *iso*-3y** = 10.1:1 by ^{19}F NMR) was filtered through a short plug of silica gel eluting with approximately 25 mL of CH_2Cl_2 and the filtrate was concentrated *in vacuo*. In order to remove 4-fluorobenzene-1-sulfonyl fluoride, the residue was sequentially added H_2O (20.0 mL), acetone (6.0 mL), $NaHCO_3$ (1.64 g, 19.5 mmol, 39.0 equiv) and Na_2SO_3 (1.91 g, 12.0 mmol, 24.0 equiv). Then the reaction mixture was stirred at 50°C for 4 hr, cooled to room temperature, extracted with CH_2Cl_2 (20.0 mL × 3), and washed with brine (20.0 mL). The combined organic layer was dried over $MgSO_4$ and concentrated *in vacuo*. The residue was purified by flash column chromatography on silica eluting with hexanes to afford 104.5 mg major isomer 4-(tert-butyl)-4'-(trifluoromethoxy)-1,1'-biphenyl (**3y**) as a white solid (71 % yield).

Spectral data for **3y**:

R_f = 0.7 (*n*-hexane), ^{1}H NMR (400 MHz, $CDCl_3$) δ 7.59 (dd, *J* = 9.2, 2.4 Hz, 2H), 7.54 – 7.46 (m, 4H), 7.28 (d, *J* = 8.1 Hz, 2H), 1.37 (d, *J* = 5.8 Hz, 9H). ^{13}C NMR (101 MHz, $CDCl_3$) δ 150.9, 148.6, 134.0, 137.1, 128.4, 126.9, 126.0, 121.3, 120.7 (q, *J* = 262.6 Hz), 34.7, 31.5. ^{19}F NMR (376 MHz, $CDCl_3$) δ -58.10 (s, 3F). Mass Spectrometry: HRMS-EI (m/z): Calcd for $C_{17}H_{17}F_3O$ $[M]^+$, 294.1231. Found, 294.1223.

4'-(Trifluoromethoxy)-[1,1'-biphenyl]-4-carbonitrile (3z) and 3'-(trifluorometh oxy)-[1,1'-biphenyl]-4-carbonitrile (iso-3z)

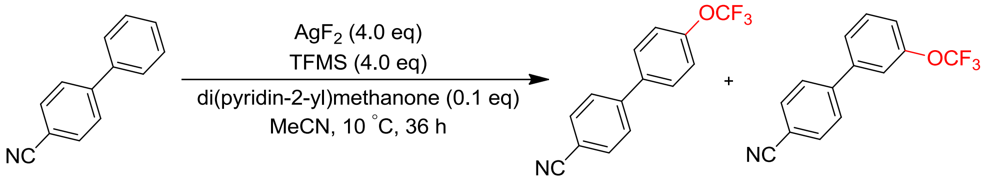

The reaction was performed according to the general procedure D using [1,1'-biphenyl]-4-carbonitrile (**1z**) (89.6 mg, 0.50 mmol) as the substrate, 3.0 mL MeCN in 8.0 mL sealed tube. After 36 hr, the reaction mixture was filtered through a short plug of silica gel eluting with approximately 25 mL of CH_2Cl_2 and the filtrate was concentrated *in vacuo*. The residue was purified by preparative HPLC (8 mL/min, detector UV λmax 210 nm, MeCN/H_2O = 50/50 (0 min), MeCN/H_2O = 70:30 (25 min), MeCN/H_2O = 90:10 (30 min) , MeCN/H_2O = 100:0 (40 min)) to afford 84 mg 4'-(trifluoromethoxy)-[1,1'-biphenyl]-4-carbonitrile (**3z**) (retention time 39.0 min) as a white solid (64 % yield) and 10.5 mg 3'-(trifluoromethoxy)-[1,1'-biphenyl]-4-carbonitrile (retention time 36.7 min) as a light yellow liquid (***iso*-3z**) (8 % yield).

Spectral data for **3z**:

R_f = 0.6 (*n*-hexane/EtOAc 50:1 (v/v)), ^{1}H NMR (400 MHz, $CDCl_3$) δ 7.72 (d, J = 8.4 Hz, 2H), 7.65 (d, J = 8.5 Hz, 2H), 7.61 (d, J = 8.8 Hz, 2H), 7.32 (d, J = 8.1 Hz, 2H). ^{13}C NMR (101 MHz, $CDCl_3$) δ 149.6, 144.2, 137.8, 132.7, 128.7, 127.7, 121.5, 120.5 (q, J = 262.6 Hz), 118.8, 111.42. ^{19}F NMR (376 MHz, $CDCl_3$) δ -58.17 (s, 3F). The spectroscopic data is in agreement with the literature.[9]

Spectral data for ***iso*-3z**:

R_f = 0.6 (*n*-hexane/EtOAc 50:1 (v/v)), ^{1}H NMR (400 MHz, $CDCl_3$) δ 7.76 – 7.71 (m, 2H), 7.60 – 7.56 (m, 2H), 7.49 – 7.36 (m, 4H). ^{13}C NMR (101 MHz, $CDCl_3$) δ 146.1, 141.6, 133.5, 132.2, 131.3, 130.1, 130.1, 127.5, 121.7, 120.4 (q, J = 262.6 Hz), 118.9, 111.70. ^{19}F NMR (376 MHz, $CDCl_3$) δ -57.70. Mass Spectrometry: HRMS-EI (m/z): Calcd for $C_{14}H_8F_3NO$ $[M]^+$, 263.0558. Found, 263.0552.

1-(4'-(Trifluoromethoxy)-[1,1'-biphenyl]-4-yl)ethanone (3aa) and 1-(3'-(trifluoro methoxy) -[1,1'-biphenyl]-4-yl)ethanone (iso-3aa)

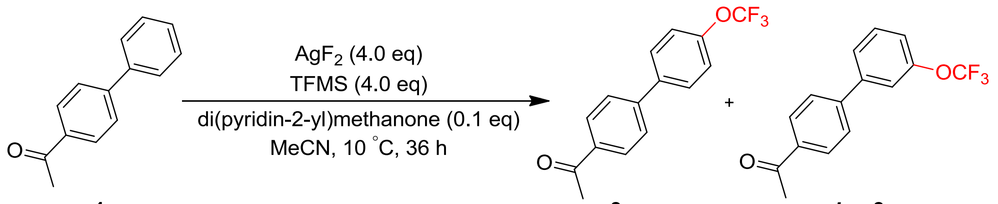

The reaction was performed according to the general procedure D using 1-([1,1'-biphenyl]-4-yl)ethanone (**1aa**) (98.1 mg, 0.50 mmol) as the substrate. After 36 hr, the reaction mixture was filtered through a short plug of silica gel eluting with approximately 25 mL of CH_2Cl_2 and the filtrate was concentrated *in vacuo*. The residue was purified by preparative HPLC (8 mL/min, detector UV λmax 210 nm, MeOH/H_2O = 50/50 (0 min), MeOH/H_2O = 70:30 (25 min), MeOH/H_2O = 90:10 (30 min) , MeOH/H_2O = 100:0 (40 min), MeOH/H_2O = 100:0 (50

min)) to afford 106.5 mg 1-(4'-(trifluoromethoxy)-[1,1'-biphenyl]-4-yl)ethanone (**3aa**) (retention time 41.5 min) as a white solid (76 % yield) and 4.2 mg 1-(3'-(trifluoromethoxy)-[1,1'-biphenyl]-4-yl)ethanone (***iso*-3aa**) (retention time 43.2 min) as a colorless liquid (3 % yield).

Spectral data for **3aa**:

R_f = 0.3 (*n*-hexane/EtOAc 50:1 (v/v)), ^{1}H NMR (400 MHz, $CDCl_3$) δ 8.03 (d, *J* = 8.3 Hz, 2H), 7.65 – 7.61 (m, 4H), 7.30 (d, *J* = 8.1 Hz, 2H), 2.63 (s, 3H). ^{13}C NMR (101 MHz, $CDCl_3$) δ 197.7, 149.4, 144.3, 138.6, 136.2, 129.1, 128.8, 127.3, 121.5, 120.6 (q, *J* = 262.6 Hz), 26.7. ^{19}F NMR (376 MHz, $CDCl_3$) δ -58.17 (s, 3F). Mass Spectrometry: HRMS-EI (m/z): Calcd for $C_{15}H_{11}F_3O_2$ $[M]^+$, 280.0711. Found, 280.0708.

Spectral data for ***iso*-3aa**:

^{1}H NMR (400 MHz, $CDCl_3$) δ 8.03 (d, *J* = 8.4 Hz, 2H), 7.57 (d, *J* = 8.4 Hz, 2H), 7.48 – 7.35 (m, 4H), 2.65 (s, 3H). ^{13}C NMR (101 MHz, $CDCl_3$) δ 198.0, 146.2, 141.8, 136.3, 134.3, 131.5, 129.6, 129.6, 128.5, 127.4, 121.8, 120.5 (d, *J* = 262.6 Hz), 26.8. ^{19}F NMR (376 MHz, $CDCl_3$) δ -57.60 (s, 3F). Mass Spectrometry: HRMS-EI (m/z): Calcd for $C_{15}H_{11}F_3O_2$ $[M]^+$, 280.0711. Found, 280.0704.

2-Phenyl-5-(4'-(trifluoromethoxy)-[1,1'-biphenyl]-4-yl)-1,3,4-oxadiazole (3bb) and 2-(4'-(trifluoromethoxy)-[1,1'-biphenyl]-4-yl)-5-(4-(trifluoromethoxy)phenyl)-1,3,4-oxadiazole (3bb')

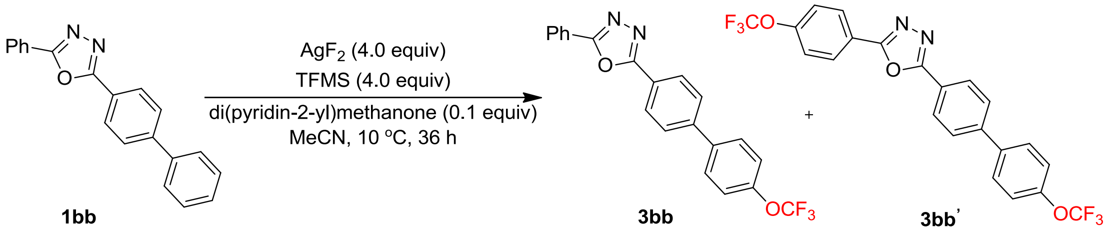

1bb **3bb** **3bb'**

The reaction was performed according to the general procedure D using 2-([1,1'-biphenyl]-4-yl)-5-phenyl-1,3,4-oxadiazole (**1bb**) (149.1 mg, 0.50 mmol) as the substrate, 5.0 mL MeCN in 8.0 mL sealed tube. After 36 hr, the reaction mixture was filtered through a short plug of silica gel eluting with approximately 25 mL of EtOAc and the filtrate was concentrated *in vacuo*. The residue was purified by preparative HPLC (8 mL/min, detector UV λmax 210 nm, $MeCN/H_2O$ = 80/20 (0 min), $MeCN/H_2O$ = 90:10 (25 min), $MeCN/H_2O$ = 100:0 (30 min)) to afford 128 mg 2-phenyl-5-(4'-(trifluoromethoxy)-[1,1'-biphenyl]-4-yl)-1,3,4 -oxadiazole (**3bb**) (retention time 27.1 min) as a white solid (67 % yield) and 9.3 mg 2-(4'-(trifluoromethoxy)-[1,1'-biphenyl]-4-yl)-5-(4-(trifluoromethoxy)phenyl)-1,3,4-oxadiazole (**3bb'**) (retention time 33.4 min) as a white solid (4 % yield).

Spectral data for **3bb**:

R_f = 0.3 (*n*-hexane/EtOAc 10:1 (v/v)). ^{1}H NMR (400 MHz, $CDCl_3$) δ 8.18 (d, *J* = 8.1 Hz, 2H), 8.13 (d, *J* = 7.6 Hz, 2H), 7.69 (d, *J* = 8.2 Hz, 2H), 7.64 (d, *J* = 8.5 Hz, 2H), 7.54 (m, 3H), 7.31 (d, *J* = 8.2 Hz, 2H). ^{13}C NMR (101 MHz, $CDCl_3$) δ 164.7, 164.4, 149.4, 143.0, 138.5, 131.9, 129.2, 128.6, 127.7, 127.5, 127.0, 123.9, 123.2, 121.5, 120.6 (q, *J* = 262.6 Hz). ^{19}F NMR (376 MHz, $CDCl_3$) δ -58.26 (s, 3F). Mass Spectrometry: HRMS-EI (m/z): Calcd for $C_{21}H_{13}F_3N_2O_2$ $[M]^+$, 382.0929. Found, 382.0925.

Spectral data for **3bb'**:
R_f = 0.4 (*n*-hexane/EtOAc 10:1 (v/v)). ^{1}H NMR (400 MHz, $CDCl_3$) δ 8.25 – 8.18 (m, 4H), 7.74 (d, *J* = 8.5 Hz, 2H), 7.67 (d, *J* = 8.7 Hz, 2H), 7.40 (d, *J* = 8.1 Hz, 2H), 7.34 (d, *J* = 8.1 Hz, 2H). ^{13}C NMR (101 MHz, $CDCl_3$) δ 164.7, 163.8, 151.8, 149.5, 143.3, 138.6, 128.9, 128.7, 127. 9, 127.7, 123.0, 122.5, 121.6, 121.5, 120.6 (q, *J* = 262.6 Hz), 120.5 (q, *J* = 252.5 Hz). ^{19}F NMR (376 MHz, $CDCl_3$) δ -58.07 (s, 3F), -58.20 (s, 3F). Mass Spectrometry: HRMS-ESI (m/z): Calcd for $C_{22}H_{13}F_6N_2O_3$ $[M + H]^+$, 467.0825. Found, 467.0824.

9,9-Dimethyl-3-(trifluoromethoxy)-9H-fluorene (3cc) and 9,9-dimethyl-3,6-bis(trifluoromethoxy)-9H-fluorene (3cc')

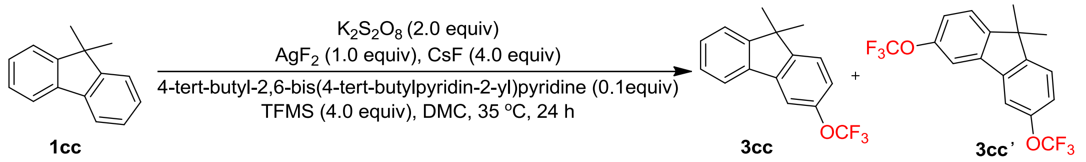

The reaction was performed according to the general procedure C using 9,9-dimethyl-9H-fluorene (**1cc**) (97.2 mg, 0.50 mmol), $K_2S_2O_8$ (270 mg, 1. 0 mmol, 2.00 equiv). After 24 hr, the reaction mixture was filtered through a short plug of silica gel eluting with approximately 25 mL of CH_2Cl_2 and the filtrate was concentrated *in vacuo*. The residue was purified by preparative HPLC (8 mL/min, detector UV λmax 210 nm, MeCN/H_2O = 80/20 (0 min), MeCN/H_2O = 100:0 (25 min), MeCN/H_2O = 100:0 (35 min)) to afford 64 mg 9,9-dimethyl-3-(trifluoromethoxy)-9H-fluorene (**3cc**) (retention time 24.1 min) as a white solid (46 % yield) and 3.6 mg 9,9-dimethyl-3,6-bis(trifluoromethoxy)-9H-fluorene (**3cc'**) (retention time 27.2 min) as a white solid (2 % yield).
Spectral data for **3cc**:
R_f = 0.8 (*n*-hexane), ^{1}H NMR (400 MHz, $CDCl_3$) δ 7.69 – 7.65 (m, 2H), 7.43 – 7.39 (m, 1H), 7.34 – 7.29 (m, 2H), 7.26 (s, 1H), 7.17 (d, *J* = 8.3 Hz, 1H), 1.46 (s, 6H). ^{13}C NMR (101 MHz, $CDCl_3$) δ 155.5, 153.7, 148.7, 138.0, 137.9, 127.6, 127.2, 122.7, 120.8, 120.7 (q, *J* = 252.5Hz), 120.1, 119.9, 115.8, 47.2, 27.0. ^{19}F NMR (376 MHz, $CDCl_3$) δ -58.06 (s, 3F). Mass Spectrometry: HRMS-EI (m/z): Calcd for $C_{16}H_{13}F_3O$ $[M]^+$, 278.0918. Found, 278.0909.
Spectral data for **3cc'**:
R_f = 0.8 (*n*-hexane), ^{1}H NMR (400 MHz, $CDCl_3$) δ 7.66 (d, *J* = 8.2 Hz, 1H), 7.26 (s, 1H), 7.20 (d, *J* = 8.2 Hz, 1H), 1.47 (s, 6H). ^{13}C NMR (101 MHz, $CDCl_3$) δ 155.8, 149.1, 136.8, 121.13, 120.8 (q, *J* = 252.5Hz), 120.30, 116.00, 47.5, 26.9. ^{19}F NMR (376 MHz, CDCl3) δ -57.88 (s, 3F). Mass Spectrometry: HRMS-EI (m/z): Calcd for $C_{17}H_{12}F_6O_2$ $[M]^+$, 362.0741. Found, 362.0731.

2-(Trifluoromethoxy)dibenzo[b,d]furan (3dd) and 4-(trifluoromethoxy)dibenzo [b,d]furan (iso-3dd)

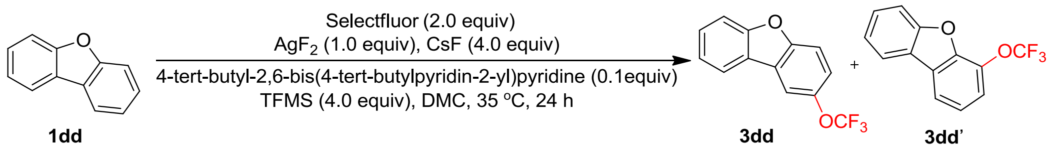

The reaction was performed according to the general procedure C using dibenzo[b,d]furan (**1dd**) (84 mg, 0.50 mmol) as the substrate. After 24 hr, the reaction mixture was filtered through a short plug of silica gel eluting with approximately 25 mL of CH_2Cl_2 and the filtrate was concentrated *in vacuo*. The residue was purified by preparative HPLC (8 mL/min, detector UV λmax 210 nm, MeCN/H_2O = 80/20 (0 min), MeCN/H_2O = 90:10 (20 min), MeCN/H_2O = 100:0 (30 min)) to afford 39 mg 2-(trifluoromethoxy)dibenzo[b,d]furan (**3dd**) (retention time 23.8 min) as a white solid (31 % yield) and 29 mg 4-(trifluoromethoxy)dibenzo[b,d]furan (***iso*-3dd**) (retention time 25.2 min) as a white solid (23 % yield).

Spectral data for **3dd**:

R_f = 0.8 (*n*-hexane), ^{1}H NMR (400 MHz, $CDCl_3$) δ 7.92 (t, *J* = 7.2 Hz, 2H), 7.58 (d, *J* = 8.2 Hz, 1H), 7.52 – 7.43 (m, 2H), 7.37 (t, *J* = 7.4 Hz, 1H), 7.24 (d, *J* = 8.3 Hz, 1H). ^{13}C NMR (101 MHz, $CDCl_3$) δ 157.1, 156.2, 148.3, 127.6, 123.4, 123.3, 123.2, 120.8 (q, *J* = 252.5 Hz), 121.2, 120.8, 116.3, 111.9, 105.5. ^{19}F NMR (376 MHz, $CDCl_3$) δ -58.21 (s, 3F). The spectroscopic data is in agreement with the literature. [10]

Spectral data for ***iso*-3dd**:

R_f = 0.8 (*n*-hexane), ^{1}H NMR (400 MHz, $CDCl_3$) δ 8.10 (d, *J* = 7.7 Hz, 1H), 7.60 (d, *J* = 8.3 Hz, 1H), 7.55 – 7.49 (m, 2H), 7.45 (t, *J* = 8.1 Hz, 1H), 7.40 (t, *J* = 7.5 Hz, 1H), 7.23 (d, *J* = 8.8 Hz, 1H). ^{13}C NMR (101 MHz, $CDCl_3$) δ 157.4, 156.1, 144.1, 128.0, 127.5, 123.5, 123.1, 121.9, 121.0 (q, *J* = 262.6 Hz), 117.7, 114.3, 111.7, 110.4. ^{19}F NMR (376 MHz, $CDCl_3$) δ -57.90 (s, 3F). Mass Spectrometry: HRMS-EI (m/z): Calcd for $C_{13}H_7F_3O_2$ $[M]^+$, 252.0398. Found, 252.0388.

9-Tosyl-3-(trifluoromethoxy)-9H-carbazole (3ee) and 9-tosyl-1-(trifluorometho xy)-9H-carbazole (iso-3ee)

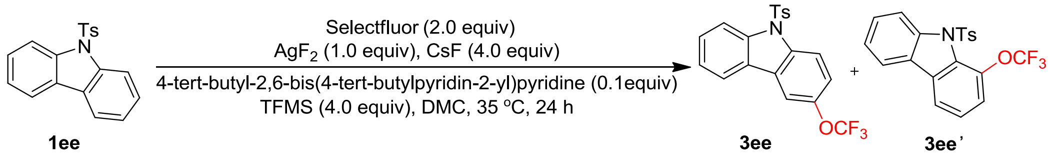

The reaction was performed according to the general procedure C using 9-tosyl-9H-carbazole (**1ee**) (160.7 mg, 0.50 mmol) as the substrate. After 24 hr, the reaction mixture was filtered through a short plug of silica gel eluting with approximately 25 mL of CH_2Cl_2 and the filtrate was concentrated *in vacuo*. The residue was purified by preparative HPLC (8 mL/min, detector UV λmax 210 nm, MeCN/H_2O = 85/15 (0 min), MeCN/H_2O = 90:10 (25 min), MeCN/H_2O = 100:0 (35 min)) to afford 85.1 mg 9-tosyl-3-(trifluoromethoxy)-9H-carbazole (**3ee**) (retention time 25.2 min) as a white solid (42 % yield) and 79 mg 9-tosyl-1-(trifluoromethoxy)-9H-carbazole (***iso*-3ee**) (retention time 26.7 min) as a white solid (39 % yield).

Spectral data for **3ee**:

R_f = 0.6 (*n*-hexane/EtOAc 10:1 (v/v)), ^{1}H NMR (400 MHz, $CDCl_3$) δ 8.32 (d, *J* = 8.4 Hz, 1H), 8.24 (s, 1H), 7.84 (d, *J* = 8.1 Hz, 2H), 7.68 (d, *J* = 7.8 Hz, 2H), 7.50 (t, *J* = 7.8 Hz, 1H), 7.36 (t, *J* = 7.5 Hz, 1H), 7.22 (d, *J* = 8.4 Hz, 1H), 7.09 (d, *J* = 7.9 Hz, 2H), 2.24 (s, 3H). ^{13}C NMR (101 MHz, $CDCl_3$) δ 148.4, 145.5, 139.1, 138.7, 134.7, 129.9, 127.9, 126.6, 125.5, 125.1, 124.4, 120.8, 120.7 (q, *J* = 252.5 Hz), 120.2, 117.4, 115.3, 108.9, 21.6. ^{19}F NMR (376 MHz, $CDCl_3$) δ -58.14 (s, 3F). Mass Spectrometry: HRMS-ESI (m/z): Calcd for $C_{20}H_{14}F_3NNaO_3S$ $[M + Na]^+$, 428.0539. Found, 428.0542.

Spectral data for ***iso*-3ee**:
R_f = 0.6 (*n*-hexane/EtOAc 10:1 (v/v)), ^{1}H NMR (400 MHz, $CDCl_3$) δ 8.40 (d, *J* = 8.4 Hz, 1H), 8.33 (d, *J* = 8.4 Hz, 1H), 8.17 (d, *J* = 7.8 Hz, 1H), 7.76 (d, *J* = 8.2 Hz, 2H), 7.58 (t, *J* = 7.9 Hz, 1H), 7.52 (t, *J* = 8.3 Hz, 1H), 7.44 (t, *J* = 7.6 Hz, 1H), 7.28 (d, *J* = 9.0 Hz, 1H), 7.16 (d, *J* = 8.2 Hz, 2H), 2.30 (s, 3H). ^{13}C NMR (101 MHz, $CDCl_3$) δ 145.4, 144.3, 134.0, 138.4, 134.9, 129.9, 128.1, 127.7, 126.7, 124.5, 123.8, 123.2, 120.9 (q, *J* = 262.6 Hz), 118.8, 114.9, 114.7, 113.3, 21.6. ^{19}F NMR (376 MHz, $CDCl_3$) δ -57.59 (s, 3F). Mass Spectrometry: HRMS-ESI (m/z): Calcd for $C_{20}H_{14}F_3NNaO_3S$ $[M + Na]^+$, 428.0539. Found, 428.0541.

2-Chloro-6-methoxy-5-(trifluoromethoxy)quinoline (3ff)

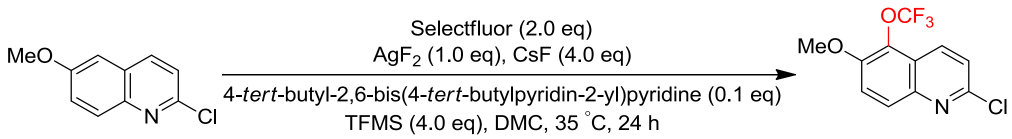

1ff **3ff**

The reaction was performed according to the general procedure C using 2-chloro-6-methoxyquinoline (**1ff**) (96.8 mg, 0.50 mmol) as the substrate. After 24 hr, the reaction mixture was filtered through a short plug of silica gel eluting with approximately 25 mL of EtOAc and the filtrate was concentrated *in vacuo*. The residue was purified by flash column chromatography on silica eluting with hexanes/ EtOAc 10:1 (v/v) to afford 105 mg 2-chloro-6-methoxy-5-(trifluoromethoxy)quinolone (**3ff**) as a light yellow solid (76 % yield).
R_f = 0.3 (*n*-hexane/EtOAc 10:1 (v/v)). ^{1}H NMR (400 MHz, $CDCl_3$) δ 8.26 (d, *J* = 8.9 Hz, 1H), 7.99 (d, *J* = 9.4 Hz, 1H), 7.57 (d, *J* = 9.4 Hz, 1H), 7.42 (d, *J* = 8.9 Hz, 1H). ^{13}C NMR (101 MHz, $CDCl_3$) δ 150.3, 149.3, 142.6, 132.4, 130.5, 129.0, 123.6, 123.1, 121.1 (q, *J* = 262.6 Hz), 118.1, 56.9. ^{19}F NMR (376 MHz, $CDCl_3$) δ -57.73 (s, 3F). Mass Spectrometry: HRMS-ESI (m/z): Calcd for $C_{11}H_8ClF_3NO_2$ $[M + H]^+$, 278.0190. Found, 278.0195.

3-Phenyl-1-tosyl-2-(trifluoromethoxy)-1H-indole (3gg)

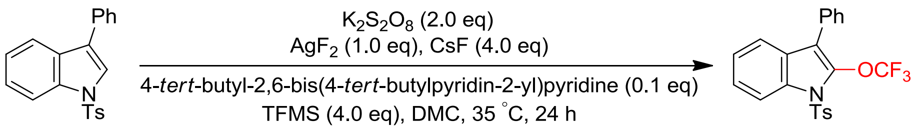

1gg **3gg**

The reaction was performed according to the general procedure C using 3-phenyl-1-tosyl-1H-indole (**1gg**) (173.7 mg, 0.50 mmol) as the substrate, $K_2S_2O_8$ (270 mg, 1. 0 mmol, 2.00 equiv). After 24 hr, the reaction mixture was filtered through a short plug of silica gel eluting with approximately 25 mL of EtOAc and the filtrate was concentrated *in vacuo*. The residue was purified by flash column chromatography on silica eluting with hexanes/ EtOAc 10:1 (v/v) to afford 86.3 mg 3-phenyl-1-tosyl-2-(trifluoromethoxy)-1H-indole (**3gg**) as a light yellow solid (40 % yield).
R_f = 0.5 (*n*-hexane/EtOAc 10:1 (v/v)). ^{1}H NMR (400 MHz, $CDCl_3$) δ 8.27 (d, *J* = 8.4 Hz, 1H), 7.76 (d, *J* = 8.3 Hz, 2H), 7.53 (d, *J* = 7.9 Hz, 1H), 7.46 – 7.34 (m, 6H), 7.28 (t, *J* = 7.6 Hz, 1H), 7.20 (d, *J* = 8.3 Hz, 2H), 2.32 (s, 3H). ^{13}C NMR (101 MHz, $CDCl_3$) δ 145.7, 135.0, 134.8, 133.0, 130.0, 129.6, 129.4, 128.8, 128.4, 127.5, 127.0, 126.1, 124.7, 120.5 (q, *J* = 262.6 Hz), 120.4, 115.43,

114.58, 21.70. ^{19}F NMR (376 MHz, $CDCl_3$) δ -58.33 (s, 3F). Mass Spectrometry: HRMS-ESI (m/z): Calcd for $C_{22}H_{16}F_3NNaO_3S$ $[M + Na]^+$, 454.0695. Found, 454.0698.

1-(2-(Trifluoromethoxy)benzo[b]thiophen-3-yl)ethanone (3hh)

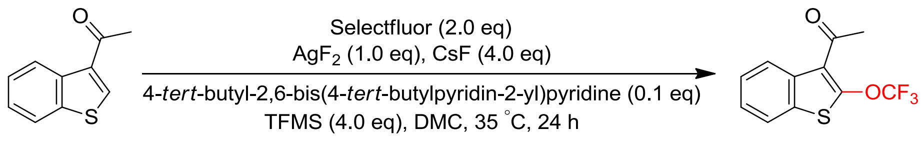

The reaction was performed according to the general procedure C using 1-(benzo[b]thiophen-3-yl)ethanone (**1hh**) (88 mg, 0.50 mmol) as the substrate. After 24 hr, the reaction mixture was filtered through a short plug of silica gel eluting with approximately 25 mL of EtOAc and the filtrate was concentrated *in vacuo*. The residue was purified by flash column chromatography on silica eluting with hexanes/ EtOAc 3:1 (v/v) to afford 63.7 mg 1-(2-(trifluoromethoxy)benzo[b]thiophen-3-yl)ethanone (**3hh**) (retention time 39.0 min) as a white solid (49 % yield).

R_f = 0.3 (*n*-hexane/EtOAc 3:1 (v/v)). ^{1}H NMR (400 MHz, $CDCl_3$) δ 8.52 (d, J = 7.9 Hz, 1H), 7.70 (d, J = 7.6 Hz, 1H), 7.45 (m, 2H), 2.66 (s, 3H). ^{13}C NMR (101 MHz, $CDCl_3$) δ 192.8, 155.5, 134.3, 132.8, 126.5, 126.4, 125.9, 124.9, 121.8, 120.3 (q, J = 272.7 Hz), 31.22. ^{19}F NMR (376 MHz, $CDCl_3$) δ -59.21 3F). Mass Spectrometry: HRMS-EI (m/z): Calcd for $C_{11}H_7F_3O_2S$ $[M]^+$, 260.0119. Found, 260.0107.

3-(Trifluoromethoxy)-4H-chromen-4-one (3ii)

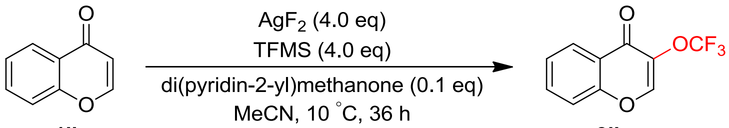

The reaction was performed according to the general procedure D using 4*H*-chromen-4-one (**1ii**) (73 mg, 0.50 mmol) as the substrate. After 36 hr, the reaction mixture was filtered through a short plug of silica gel eluting with approximately 25 mL of CH_2Cl_2 and the filtrate was concentrated *in vacuo*. The residue was purified by flash column chromatography on silica eluting with hexanes/ EtOAc 20:1 (v/v) to afford 54.1 mg 3-(trifluoromethoxy)-4*H*-chromen-4
-one (**3ii**) as a white solid (47 % yield).

R_f = 0.4 (*n*-hexane/EtOAc 20:1 (v/v)), ^{1}H NMR (400 MHz, $CDCl_3$) δ 8.25 (d, J = 8.0 Hz, 1H), 8.16 (s, 1H), 7.71 (t, J = 7.8 Hz, 1H), 7.50 (d, J = 8.5 Hz, 1H), 7.44 (t, J = 7.6 Hz, 1H). ^{13}C NMR (101 MHz, $CDCl_3$) δ 171.6, 155.9, 150.6, 135.5, 134.5, 126.3, 125.9, 124.7, 121.9 (q, J = 262.6 Hz), 118.5. ^{19}F NMR (376 MHz, $CDCl_3$) δ -59.82 (s, 3F). Mass Spectrometry: HRMS-EI (m/z): Calcd for $C_{10}H_5F_3O_3$ $[M]^+$, 230.0191. Found, 230.0183.

3-(Trifluoromethoxy)-2H-chromen-2-one (3jj)

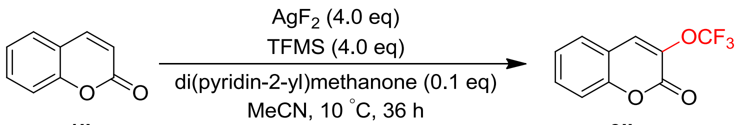

1jj **3jj**

The reaction was performed according to the general procedure D using 2*H*-chromen-2-one (**1jj**) (73 mg, 0.50 mmol) as the substrate, 0.8 mL MeCN. After 36 hr, the reaction mixture was filtered through a short plug of silica gel eluting with approximately 25 mL of CH_2Cl_2 and the filtrate was concentrated *in vacuo*. The residue was purified by flash column chromatography on silica eluting with hexanes/ EtOAc 20:1 (v/v) to afford 58.6 mg 3-(trifluoromethoxy)-2*H*-chromen-2-one (**3jj**) as a white solid (51 % yield).

R_f = 0.5 (*n*-hexane/EtOAc 20:1 (v/v)), ^{1}H NMR (400 MHz, $CDCl_3$) δ 7.65 (s, 1H), 7.58 – 7.52 (m, 2H), 7.35 – 7.31 (m, 2H). ^{13}C NMR (101 MHz, $CDCl_3$) δ 156.1, 152.1, 134.1, 132.3, 131.4, 128.5, 125.4, 121.9 (q, J = 262.6 Hz), 117.6, 116.8. ^{19}F NMR (376 MHz, $CDCl_3$) δ -59.06 (s, 3F). Mass Spectrometry: HRMS-EI (m/z): Calcd for $C_{10}H_5F_3O_3$ $[M]^+$, 230.0191. Found, 230.0183.

5-Chloro-6'-methyl-3-(4-(methylsulfonyl)phenyl)-2'-(trifluoromethoxy)-2,3'-bipyridine (4kk)

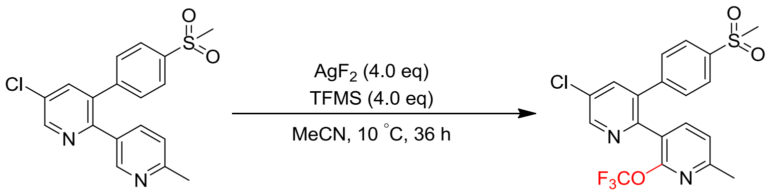

1kk **4kk**

The reaction was performed according to the general procedure B using 5-chloro-6'-methyl-3-(4-(methylsulfonyl)phenyl)-2,3'-bipyridine (**1kk**) (179.4 mg, 0.50 mmol) as the substrate. After 36 hr, the reaction mixture was filtered through a short plug of silica gel eluting with approximately 25 mL of EtOAc and the filtrate was concentrated *in vacuo*. The residue was purified by flash column chromatography on silica eluting with hexanes/ EtOAc 1:1 (v/v) to afford 48.7 mg 5-chloro-6'-methyl-3-(4-(methylsulfonyl)phenyl)-2'-(trifluoromethoxy)-2,3'-bipyridine (**4kk**) (retention time 15.5 min) as a white solid (22 % yield).

R_f = 0.6 (*n*-hexane/EtOAc 1:1 (v/v)). ^{1}H NMR (400 MHz, $CDCl_3$) δ 8.70 (d, J = 2.3 Hz, 1H), 7.87 (dd, J = 8.0, 2.5 Hz, 3H), 7.78 (d, J = 2.3 Hz, 1H), 7.32 (d, J = 8.3 Hz, 2H), 7.16 (d, J = 7.7 Hz, 1H), 3.02 (s, 3H), 2.49 (s, 3H). ^{13}C NMR (101 MHz, $CDCl_3$) δ 158.5, 151.9, 149.9, 148.4, 143.5, 142.1, 140.2, 137.3, 136.8, 132.0, 129.7, 127.8, 121.3, 120.4, 119.6 (q, J = 262.6 Hz),44.59, 24.07. ^{19}F NMR (376 MHz, $CDCl_3$) δ -56.21 (s, 3F). Mass Spectrometry: HRMS-ESI (m/z): Calcd for $C_{19}H_{15}ClF_3N_2O_3S$ $[M + H]^+$, 443.0439. Found, 443.0436.

***N*-ethyl-3-hydroxy-2-phenyl-*N*-((2-(trifluoromethoxy)pyridin-4-yl)methyl)propanamide (4ll)**

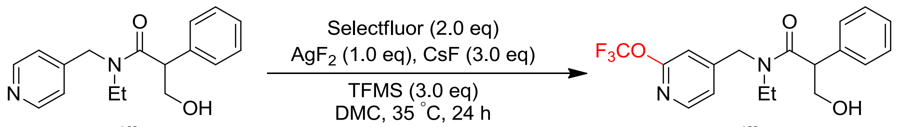

1ll **4ll**

The reaction was performed according to the general procedure A using N-ethyl-3-hydroxy-2-phenyl-N-(pyridin-4-ylmethyl)propanamide (**1ll**) (142 mg, 0.50 mmol) as the substrate. After 24 hr, the reaction mixture was filtered through a short plug of silica gel eluting with approximately 25 mL of EtOAc and the filtrate was concentrated *in vacuo*. In order to remove 4-fluorobenzene-1-sulfonyl fluoride, the residue was sequentially added H_2O (20.0 mL), acetone (6.0 mL), $NaHCO_3$ (1.64 g, 19.5 mmol, 39.0 equiv) and Na_2SO_3 (1.91 g, 12.0 mmol, 24.0 equiv). Then the reaction mixture was stirred at 50°C for 4 hr, cooled to room temperature, extracted with CH_2Cl_2 (20.0 mL × 3), and washed with brine (20.0 mL). The combined organic layer was dried over $MgSO_4$ and concentrated *in vacuo*. The residue was purified by preparative HPLC (8 mL/min, detector UV λmax 210 nm, MeCN/H_2O = 50/50 (0 min), MeCN/H_2O = 70:30 (25 min), MeCN/H_2O = 90:10 (30 min)) to afford 46.1 mg N-ethyl-3-hydroxy-2-phenyl-N-((2-(trifluoromethoxy)pyridin-4-yl)methyl)propanamide (**4ll**) (retention time 21.0 min) as a white solid (25 % yield).

Spectral data for **4ll**: *The compound exists as a 2.5:1 ratio of amide diastereomers on the NMR time scale.*

R_f= 0.4 (*n*-hexane/EtOAc 1:1 (v/v)). ^{1}H NMR (400 MHz, $CDCl_3$) δ 8.23 – 8.20 (m), 7.42 – 7.24 (m), 7.18 (d, *J* = 8.0 Hz), 7.01 (d, *J* = 5.0 Hz), 6.87 (d, *J* = 4.9 Hz), 6.76 (s), 6.60 (s), 4.81 (d, *J* = 16.2 Hz), 4.54 – 4.27 (m), 4.09 (m), 3.88 – 3.54 (m), 3.45 – 3.06 (m), 1.12 (t, *J* = 7.1 Hz), 0.97 (t, *J* = 7.1 Hz). ^{13}C NMR (101 MHz, $CDCl_3$) δ 173.0, 172.7, 157.4, 157.3, 152.1, 151.2, 148.2, 148.0, 135.9, 135.6, 129.3, 129.3, 128.1, 128.0, 127.9, 120.5, 120.1 (q, *J* = 262.6 Hz), 119.5, 111.2, 110.4, 65.9, 52.3, 52.0, 49.2, 47.5, 42.6, 41.5, 13.6, 12.4. ^{19}F NMR (376 MHz, $CDCl_3$) δ -56.48 (s, 3F), -56.49 (s, 3F). Mass Spectrometry: HRMS-EI (m/z): Calcd for $C_{18}H_{19}F_3N_2O_3$ $[M]^+$, 368.1348. Found, 368.1343.

N-(4-(4-fluorophenyl)-6-isopropyl-5-((pyridin-4-yloxy)methyl)pyrimidin-2-yl)-N-methylmethanesulfonamide (1mm)

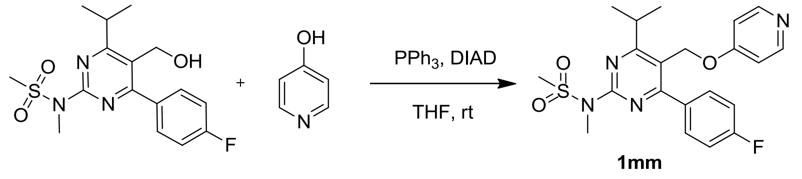

To a solution of N-(4-(4-fluorophenyl)-5-(hydroxymethyl)-6-isopropylpyrimidin-2-yl)-N-methylmethanesulfonamide (812.8 mg, 2.3 mmol), 4-hydroxypyridine (273.9 mg, 1.25 mmol) and triphenylphosphine (755.4 mg, 1.25 mmol) in anhydrous THF (16 ml), a solution of DIAD (812.8 mg, 2.3 mmol) in THF (5 ml) was added dropwise at 0 °C. The resulting dark-green solution was allowed to warm to rt and the mixture was stired for additional 24 hr. The mixture was evaporated to dryness under reduced pressure and the residue was purified by column chromatography eluting with hexanes/ EtOAc 2:1 (v/v) to give the crude product with triphenylphosphine oxide. Then the mixture was purified by preparative HPLC (8 mL/min, detector UV λmax 210 nm, MeCN/H_2O = 50/50 (0 min), MeCN/H_2O = 70:30 (25 min), MeCN/H_2O = 90:10 (40 min)) to afford 514.8 mg N-(4-(4-fluorophenyl)-6-isopropyl-5-((pyridin-4-yloxy)methyl)pyrimidin-2-yl)-N-methylmethanesulfonamide (**1mm**) (retention time 32.5 min) as a white solid (52 % yield).

R_f= 0.3 (*n*-hexane/EtOAc 2:1 (v/v)), ^{1}H NMR (400 MHz, $CDCl_3$) δ 8.49 (dd, *J* = 4.9, 1.4 Hz, 2H), 7.69 – 7.61 (m, 2H), 7.15 – 7.06 (m, 2H), 6.85 (dd, *J* = 4.8, 1.5 Hz, 2H), 4.96 (s, 2H), 3.60 (s, 3H), 3.53 (s, 3H), 3.26 (m, 1H), 1.33 (d, *J* = 6.6 Hz, 6H). ^{13}C NMR (101 MHz, $CDCl_3$) δ 178.5, 166.9, 164.0, 163.9 (d, *J* = 242.2 Hz), 158.7, 151.5, 133.6 (d, *J* = 3.0 Hz), 131.3 (d, *J* = 9.1 Hz), 116.5, 115.8 (d, *J* = 21.2 Hz), 110.4, 63.4, 42.6, 33.2, 32.0, 22.2. Mass Spectrometry: HRMS-ESI (m/z): Calcd for $C_{21}H_{24}FN_4O_3S$ $[M + H]^+$, 431.1548. Found, 431.1551.

_N_-(4-(4-fluorophenyl)-6-isopropyl-5-(((2-(trifluoromethoxy)pyridin-4-yl)oxy)methyl)pyrimidin-2-yl)-_N_-methylmethanesulfonamide (4mm)

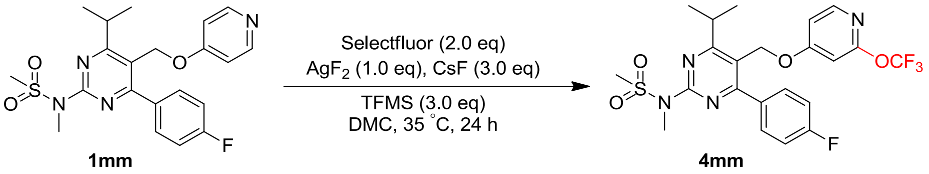

The reaction was performed according to the general procedure A using N-(4-(4-fluorophenyl)-6-isopropyl-5-((pyridin-4-yloxy)methyl)pyrimidin-2-yl)-N-methylmethanesulfonamide (**1mm**) (107.6 mg, 0.25 mmol, 1.0 eq) as the substrate. After 24 hr, the reaction mixture was filtered through a short plug of silica gel eluting with approximately 25 mL of EtOAc and the filtrate was concentrated *in vacuo*. The residue was purified by flash column chromatography on silica eluting with hexanes/ CH_2Cl_2 1:1.5 (v/v) to afford 56.6 mg N-(4-(4-fluorophenyl)-6-isopropyl-5-(((2-(trifluoromethoxy)pyridin-4-yl)oxy)methyl)pyrimidin-2-yl)-N-methylmethanesulfonamide (**4mm**) as a white solid (44 % yield).
R_f= 0.3 (*n*-hexane/CH_2Cl_2 1:1.5 (v/v)), ^{1}H NMR (400 MHz, $CDCl_3$) δ 8.20 (d, *J* = 5.8 Hz, 1H), 7.70 – 7.54 (m, 2H), 7.21 – 7.04 (m, 2H), 6.79 (dd, *J* = 5.8, 2.2 Hz, 1H), 6.49 (d, *J* = 2.1 Hz, 1H), 4.98 (s, 2H), 3.60 (s, 3H), 3.53 (s, 3H), 3.24 (m, 1H), 1.34 (d, *J* = 6.6 Hz, 6H). ^{13}C NMR (101 MHz, $CDCl_3$) δ 178.5, 167.1, 167.0, 164.0 (d, *J* = 251.5 Hz), 158.8, 158.5, 148.9, 133.5 (d, *J* = 3.0 Hz), 131.3 (d, *J* = 8.1 Hz), 120.2 (d, *J* = 262.6 Hz), 115.9 (d, *J* = 21.2 Hz), 109.8, 98.6, 64.2, 42.6, 33.2, 32.1, 22.2. ^{19}F NMR (376 MHz, $CDCl_3$) δ -56.29 (s, 3F). Mass Spectrometry: HRMS-ESI (m/z): Calcd for $C_{22}H_{23}F_4N_4O_4S$ $[M + H]^+$, 515.1371. Found, 515.1375.

Methyl(4-chloro-3-(6-(trifluoromethoxy)pyridin-2-yl)phenyl)(2-chloro-4-(methylsulfonyl)benzoyl)carbamate (4nn)

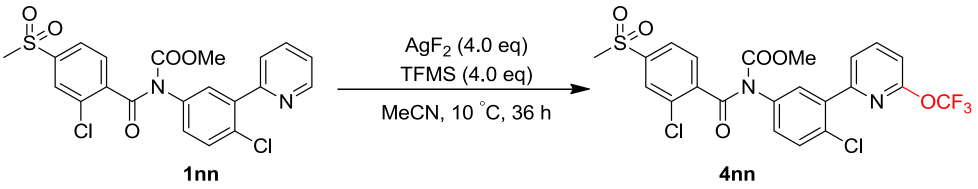

The reaction was performed according to the general procedure B using (CO_2Me)-vismodegib [11] (**1nn**) (239.6 mg, 0.50 mmol) as the substrate, 2.0 mL MeCN in 4.0 mL sealed tube. After 36 hr, the reaction mixture was filtered through a short plug of silica gel eluting with approximately 25 mL of EtOAc and the filtrate was concentrated *in vacuo*. The residue was purified by flash column

chromatography on silica eluting with hexanes/ EtOAc 1:1 (v/v) to afford 62 mg methyl(4-chloro-3-(6-(trifluoromethoxy) pyridin-2-yl)phenyl)(2-chloro-4-(meth-ylsulfonyl) benzoyl)carbamate (**4nn**) (retention time 18.9 min) as a white solid (22 % yield). R_f = 0.5 (*n*-hexane/EtOAc 1:1 (v/v)). ^{1}H NMR (400 MHz, $CDCl_3$) δ 8.00 (d, *J* = 1.5 Hz, 1H), 7.95 – 7.86 (m, 2H), 7.76 (d, *J* = 7.6 Hz, 1H), 7.67 – 7.56 (m, 3H), 7.32 (dd, *J* = 8.5, 2.5 Hz, 1H), 7.05 (d, *J* = 8.1 Hz, 1H), 3.69 (s, 3H), 3.09 (s, 3H). ^{13}C NMR (101 MHz, $CDCl_3$) δ 167.9, 156.3, 153.8, 153.4, 142.6, 141.9, 140.4, 138.5, 135.7, 132.9, 131.8, 131.6, 131.1, 129.8, 129.0, 128.5, 126.2, 122. 9, 120.3 (q, *J* = 262.6 Hz), 112.0, 54.7, 44.6. ^{19}F NMR (376 MHz, $CDCl_3$) δ -56.55 (s, 3F). Mass Spectrometry: HRMS-ESI (m/z): Calcd for $C_{22}H_{15}Cl_2F_3N_2NaO_6S$ $[M + Na]^+$, 584.9872. Found, 584.9875.

(2S,5R)-3-(Pyridin-4-yloxy)propyl 3,3-dimethyl-7-oxo-4-thia-1-azabicyclo[3.2.0] heptane-2-carboxylate 4,4-dioxide (1oo)

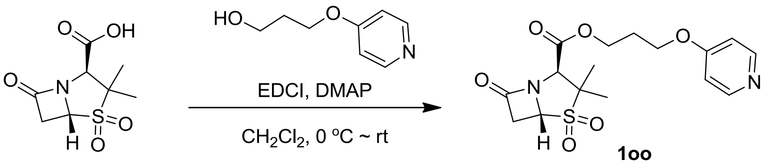

To a stirring solution of sulbactam (466.5 mg, 2.0 mmol, 1.0 equiv.) and 3-(pyridin-4-yloxy)propan-1-ol (459.5 mg, 3.0 mmol, 1.5 equiv.) in CH_2Cl_2 (4 mL, 0.5 M) at 0 °C was added EDC·HCl (575 mg, 3.0 mmol, 1.5 equiv.) and DMAP (89.6 mg, 0.8 mmol, 0.4 equiv.). The reaction mixture was warmed to rt and stirred overnight. The reaction contents were added to an appropriately sized separatory funnel and washed with a 1:1 volume each of saturated aqueous solution of $NaHCO_3$, 10 wt% aqueous solution of citric acid, and brine. Following each of the first two washes, the aqueous layer was extracted with CH_2Cl_2 (twice), and the combined organic layers were taken on to the next wash. The combined organic layers were then dried over $MgSO_4$, filtered, and concentrated under reduced pressure. The crude mixture was purified by flash column chromatography on silica eluting with hexanes/ EtOAc 1:3 (v/v) to afford 368 mg sulbactam derivative (**1oo)** a colorless oil (50 %).
R_f = 0.3 (n-hexane/EtOAc 1:3 (v/v)), ^{1}H NMR (400 MHz, $CDCl_3$) δ 8.41 (d, *J* = 3.3 Hz, 2H), 6.78 (d, *J* = 5.5 Hz, 2H), 4.59 (dd, *J* = 4.1, 2.2 Hz, 1H), 4.50 – 4.27 (m, 3H), 4.08 (t, *J* = 5.9 Hz, 2H), 3.43 (qd, *J* = 16.2, 3.1 Hz, 2H), 2.18 (p, *J* = 6.2 Hz, 2H), 1.56 (s, 3H), 1.36 (s, 3H). ^{13}C NMR (101 MHz, $CDCl_3$) δ 170.9, 167.0, 164.6, 151.2, 110.2, 63.9, 63.3, 63.1, 62.7, 61.2, 38.4, 28.1, 20.4, 18.7. Mass Spectrometry: HRMS-ESI (m/z): Calcd for $C_{16}H_{21}N_2O_6S$ $[M + H]^+$, 369.1115. Found, 369.1119.

(2S,5R)-3-((2-(Trifluoromethoxy)pyridin-4-yl)oxy)propyl 3,3-dimethyl-7-oxo-4- thia-1-azabicyclo[3.2.0]heptane-2-carboxylate 4,4-dioxide (4oo)

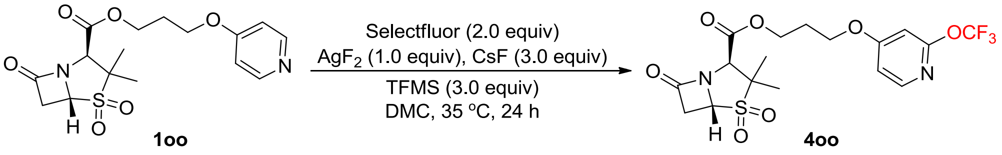

The reaction was performed according to the general procedure A using sulbactam derivative (**1oo**) (184.1 mg, 0.5 mmol) as the substrate. After 24 hr, the reaction mixture was filtered through a short plug of silica gel eluting with approximately 25 mL of EtOAc and the filtrate was concentrated *in vacuo*. The residue was purified by flash column chromatography on silica eluting with hexanes/ EtOAc 1:1 (v/v) to afford 90.5 mg (2S,5R)-3-((2-(trifluoromethoxy)pyri-din-4-yl)oxy)propyl 3,3-dimethyl-7-oxo-4-thia-1-azabicyclo[3.2.0]heptane-2-carboxylate4,4 -dioxide (**4oo**) as a colorless liquid (40 % yield).

R_f = 0.5 (n-hexane/EtOAc 1:1 (v/v)), ^{1}H NMR (400 MHz, $CDCl_3$) δ 8.13 (d, J = 5.8 Hz, 1H), 6.74 (dd, J = 5.8, 2.1 Hz, 1H), 6.46 (d, J = 1.9 Hz, 1H), 4.60 (dd, J = 4.1, 2.2 Hz, 1H), 4.47 – 4.31 (m, 3H), 4.11 (t, J = 5.9 Hz, 2H), 3.54 – 3.37 (m, 2H), 2.28 – 2.13 (m, 2H), 1.58 (s, 3H), 1.38 (s, 3H). ^{13}C NMR (101 MHz, $CDCl_3$) δ 170.9, 167.7, 167.0, 158.4, 148.7, 120.2 (q, J = 262.6 Hz), 109.7, 98.4, 64.7, 63.3, 62.9, 62.7, 61.2, 38.5, 28.1, 20.4, 18.7. ^{19}F NMR (376 MHz, $CDCl_3$) δ -56.27 (s, 3F). Mass Spectrometry: HRMS-ESI (m/z): Calcd for $C_{17}H_{20}F_3N_2O_7S$ $[M + H]^+$, 453.0938. Found, 453.0944.

(1S,4R)-3-(Pyridin-4-yloxy)propyl 4,7,7-trimethyl-3-oxo-2-oxabicyclo[2.2.1]hep -tane-1-carboxylate (1pp)

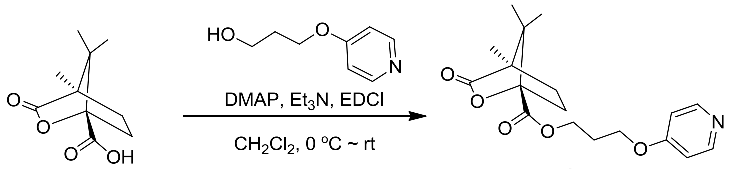

1pp

To a solution of (1S)-(-)-camphanic acid (495.6 mg, 2.50 mmol, 1.00 equiv), DMAP (61 mg, 0.50 mmol, 0.20 equiv) and Et_3N (555 mg, 5.5 mmol, 2.20 equiv) in CH_2Cl_2 (5.0 mL) at room temperature were added EDCI (1-ethyl-(3-(3-dimethylamino)-propyl)-carbodiimide hydrochloride) (960 mg, 5.0 mmol, 2.00 equiv) and 3-(pyridin-4-yloxy)propan-1-ol (651 mg, 4.25 mmol, 1.50 equiv). The reaction mixture was stirred at rt for 12 hr before quenched with H_2O (15.0 mL) and extracted three times with CH_2Cl_2 (15.0 mL). The combined organic layer was dried over $MgSO_4$. The filtrate was concentrated *in vacuo.* Then the mixture was purified by preparative HPLC (8 mL/min, detector UV λmax 210 nm, MeCN/H_2O = 50/50 (0 min), MeCN/H_2O = 70:30 (25 min), MeCN/H_2O = 90:10 (30 min) , MeCN/H_2O = 100:0 (40 min)) to afford 291.6 mg (1S)-(-)-camphanic acid derivative (**1pp**) (retention time 38.5 min) a white solid (35 %).

R_f = 0.2 (n-hexane/EtOAc 1:1 (v/v)), ^{1}H NMR (400 MHz, $CDCl_3$) δ 8.39 (d, J = 5.9 Hz, 1H), 6.76 (d, J = 6.2 Hz, 1H), 4.51 – 4.28 (m, 2H), 4.08 (t, J = 6.0 Hz, 2H), 2.38 (m, 1H), 2.28 – 2.09 (m, 2H), 1.98 (m, 1H), 1.93 – 1.83 (m, 1H), 1.65 (m, 1H), 1.07 (s, 3H), 1.00 (s, 3H), 0.88 (s, 3H). ^{13}C NMR (101 MHz, $CDCl_3$) δ 178.1, 167.5, 164.7, 151.2, 110.2, 91.1, 63.9, 62.1, 54.8, 54.3, 30.7, 28.9, 28.2, 16.8, 16.8, 9.7. Mass Spectrometry: HRMS-ESI (m/z): Calcd for $C_{18}H_{24}NO_5$ $[M + H]^+$, 334.1649. Found, 334.1652.

(1R,4R)-3-((2-(Trifluoromethoxy)pyridin-4-yl)oxy)propyl 4,7,7-trimethyl-3-oxo-2-oxabicyclo[2.2.1]heptane-1-carboxylate (4pp)

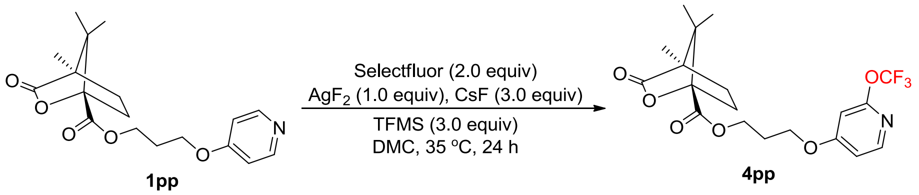

The reaction was performed according to the general procedure A using (1S)-(-)-camphanic acid derivative (**1pp**) (83.4 mg, 0.25 mmol, 1.0 eq) as the substrate. After 24 hr, the reaction mixture was filtered through a short plug of silica gel eluting with approximately 25 mL of EtOAc and the filtrate was concentrated *in vacuo*. The residue was purified by flash column chromatography on silica eluting with hexanes/ EtOAc 1.5:1 (v/v) to afford 48 mg (1R,4R)-3-((2-(trifluoromethoxy)pyridin-4-yl)oxy)propyl 4,7,7-trimethyl-3-oxo-2-oxabicyclo [2.2.1]heptane-1-carboxylate (**4pp**) as a colorless liquid (46 % yield).

R_f = 0.5 (n-hexane/EtOAc 1.5:1 (v/v)), ^{1}H NMR (400 MHz, $CDCl_3$) δ 8.11 (d, J = 5.7 Hz, 1H), 6.73 (d, J = 4.7 Hz, 1H), 6.45 (s, 1H), 4.40 (m, 2H), 4.11 (t, J = 5.8 Hz, 2H), 2.45 – 2.34 (m, 1H), 2.25 – 2.15 (m, 2H), 2.00 (m, 1H), 1.95 – 1.84 (m, 1H), 1.72 – 1.59 (m, 1H), 1.09 (s, 3H), 1.02 (s, 3H), 0.90 (s, 3H). ^{13}C NMR (101 MHz, $CDCl_3$) δ 178.2, 167.8, 167.6, 158.4, 148.7, 120.2 (d, J = 262.6 Hz), 109.7, 98.5, 91.1, 64.8, 61.9, 54.9, 54.3, 30.8, 29.0, 28.2, 16. 8, 9.8. ^{19}F NMR (376 MHz, $CDCl_3$) δ -56.30 (s, 3F). Mass Spectrometry: HRMS-ESI (m/z): Calcd for $C_{19}H_{23}F_3NO_6$ [M + H]$^+$, 418.1472. Found, 418.1475.

(R)-3-(Pyridin-4-yloxy)propyl 4-((3R,5R,8R,9S,10S,13R,14S,17R)-3-methoxy-10, 13-dimethylhexadecahydro-1H-cyclopenta[a]phenanthren-17-yl)pentanoate (1qq)

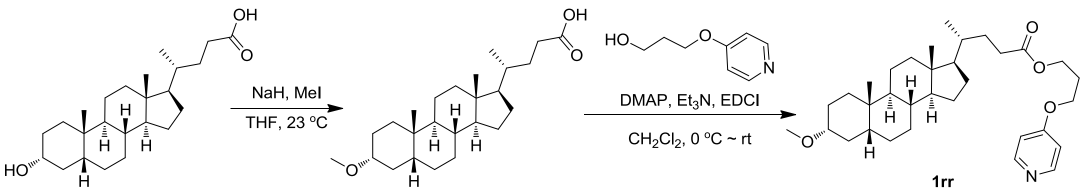

To a suspension of NaH (342 mg of a 60 % suspension in mineral oil, 8.5 mmol, 3.40 equiv) in 20.0 mL of dry THF at 0 °C, was added lithocholic acid (941mg, 2.50 mmol, 1.00 equiv) in portions. The resulting solution was stirred at 0 °C for 10 min, and then was added methyl iodide (0.25 mL, 3.9 mmol, 1.56 equiv). The reaction mixture was stirred for 18 h at rt and then was added a second portion of NaH (280 mg, 7.0 mmol, 2.78 equiv), followed by more methyl iodide (0.20 mL, 3.2 mmol, 1.30 equiv). The reaction was stirred for an additional 36 h at ambient temperature. The reaction mixture was quenched by slow addition of methanol then concentrated *in vacuo*. The resulting mixture was diluted with ethyl acetate (50.0 mL) and poured on water (40.0 mL). The aqueous layer was then extracted three times with EtOAc (25.0 mL). The combined organics were washed with brine (25.0 mL), dried over magnesium sulfate, filtered and concentrated *in vacuo*. The crude product *O*-methyl lithocholic acid was taken on to the next reaction without further purification.

To a solution of crude *O*-methyl lithocholic acid, DMAP (61 mg, 0.50 mmol, 0.20 equiv) and Et_3N (555 mg, 5.5 mmol, 2.20 equiv) in CH_2Cl_2 (5.0 mL) at room temperature were added EDCI

(1-ethyl-(3-(3-dimethylamino)-propyl)-carbodiimide hydrochloride) (960 mg, 5.0 mmol, 2.00 equiv) and 3-(pyridin-4-yloxy)propan-1-ol (651 mg, 4.25 mmol, 1.50 equiv). The reaction mixture was stirred at rt for 12 hr before quenched with H_2O (30.0 mL) and extracted three times with CH_2Cl_2 (30.0 mL). The combined organic layer was dried over $MgSO_4$. The filtrate was concentrated *in vacuo*. The residue was purified by chromatography on silica gel, eluting with PE/EtOAc 2:1 (v/v) to afford 394.4 mg *O*-methyl lithocholic acid derivative (**1qq**) a colorless oil (30 %).

R_f = 0.3 (n-hexane/EtOAc 2:1 (v/v)), ^{1}H NMR (400 MHz, $CDCl_3$) δ 8.43 (d, *J* = 5.4 Hz, 2H), 6.80 (d, *J* = 6.1 Hz, 2H), 4.25 (t, *J* = 6.3 Hz, 2H), 4.09 (t, *J* = 6.1 Hz, 2H), 3.35 (s, 3H), 3.16 (ddd, *J* = 15.4, 10.9, 4.5 Hz, 1H), 2.41 – 2.29 (m, 1H), 2.28 – 2.19 (m, 1H), 2.19 – 2.10 (m, 2H), 1.93 (d, *J* = 12.4 Hz, 1H), 1.88 – 1.72 (m, 7H), 1.45 – 1.21 (m, 13H), 1.14 – 0.99 (m, 5H), 0.90 (d, *J* = 8.2 Hz, 6H), 0.62 (s, 3H). ^{13}C NMR (101 MHz, $CDCl_3$) δ 173.9, 164.6, 150.9, 110.1, 80.2, 64.1, 60.5, 56.3, 55.7, 55.3, 42.5, 41.8, 40.2, 40.0, 35.6, 35.2, 35.2, 34.7, 32.6, 31.0, 30.8, 28.2, 28.1, 27.2, 26.6, 26.2, 24.0, 23.3, 20.6, 18.1, 11.9. Mass Spectrometry: HRMS-ESI (m/z): Calcd for $C_{33}H_{52}NO_4$ $[M + H]^+$, 526.3891. Found, 526.3893.

(R)-3-((2-(Trifluoromethoxy)pyridin-4-yl)oxy)propyl 4-((3R,5R,8R,9S,10S,13R, 14S,17R)-3-methoxy-10,13-dimethylhexadecahydro-1H-cyclopenta[a]phenanthren-17-yl)pentanoate (4qq)

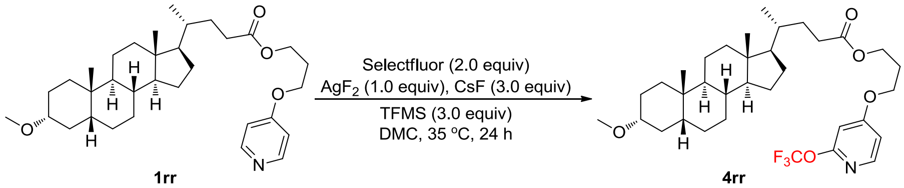

The reaction was performed according to the general procedure A using *O*-methyl lithocholic acid derivative (**1qq**) (26.3 mg, 0.05 mmol, 1.0 eq) as the substrate. After 24 hr, the reaction mixture was filtered through a short plug of silica gel eluting with approximately 25 mL of EtOAc and the filtrate was concentrated *in vacuo*. The residue was purified by flash column chromatography on silica eluting with hexanes/ EtOAc 4:1 (v/v) to afford 13.1 mg (R)-3-((2-(trifluoromethoxy)pyridin-4-yl)oxy)propyl 4-((3R,5R,8R,9S,10S,13R,14S,17R)-3-methoxy-10,13-dimethylhexadecahydro-1H-cyclopenta[a]phenanthren-17-yl)pentanoate (**4qq**) as a colorless liquid (43 % yield).

R_f = 0.5 (n-hexane/EtOAc 4:1 (v/v)), ^{1}H NMR (400 MHz, $CDCl_3$) δ 8.13 (d, *J* = 5.7 Hz, 1H), 6.74 (dd, *J* = 5.6, 1.4 Hz, 1H), 6.47 (d, *J* = 1.3 Hz, 1H), 4.25 (t, *J* = 6.1 Hz, 2H), 4.10 (t, *J* = 6.1 Hz, 2H), 3.34 (s, 3H), 3.23 – 3.06 (m, 1H), 2.35 (ddd, *J* = 15.1, 10.0, 5.0 Hz, 1H), 2.28 – 2.19 (m, 1H), 2.19 – 2.10 (m, 2H), 1.92 (d, *J* = 12.1 Hz, 1H), 1.88 – 1.62 (m, 7H), 1.46 – 0.98 (m, 18H), 0.90 (d, *J* = 7.8 Hz, 6H), 0.61 (s, 3H). ^{13}C NMR (101 MHz, $CDCl_3$) δ 174.4, 168.0, 158.5, 148.6, 120.2 (q, *J* = 262.6 Hz), 109.9, 98.4, 80.5, 65.2, 60.6, 56.6, 56.0, 55.7, 55.0, 42.9, 42.2, 40.4, 40.3, 34.0, 35.5, 35.4, 35.0, 32.9, 31.3, 31.1, 28.4, 27.4, 26.9, 26.5, 24.3, 23.5, 20.9, 18.4, 12.2. ^{19}F NMR (376 MHz, $CDCl_3$) δ -56.15 (s, 3F). Mass Spectrometry: HRMS-ESI (m/z): Calcd for $C_{34}H_{51}F_3NO_5$ $[M + H]^+$, 610.3714. Found, 610.3712.

1,6,6-Trimethyl-2-(trifluoromethoxy)-6,7,8,9-tetrahydrophenanthro[1,2-b]furan-10,11-dione (4rr)

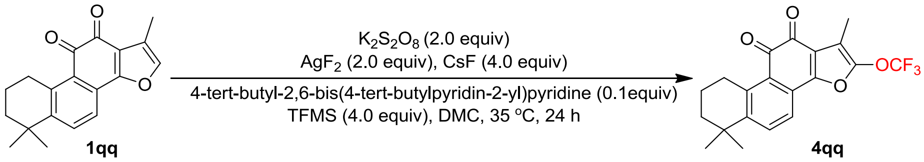

The reaction was performed according to the general procedure C using 1,6,6-trimethyl-6,7,8,9-tetrahydrophenanthro[1,2-b]furan-10,11-dione (**1rr**) (147 mg, 0.50 mmol, 1.0 eq) as the substrate, $K_2S_2O_8$ (270 mg, 1.0 mmol, 2.00 equiv), AgF_2 (145 mg, 1.0 mmol, 2.00 equiv). After 24 hr, the reaction mixture was filtered through a short plug of silica gel eluting with approximately 25 mL of EtOAc and the filtrate was concentrated *in vacuo*. The residue was purified by flash column chromatography on silica eluting with hexanes/ EtOAc 10:1 (v/v) to afford 60.5 mg 1,6,6-trimethyl-2-(trifluoromethoxy)-6,7,8,9-tetrahydrophenanth-ro[1,2-b]furan-10,11-dione (**4rr**) as a red solid (32 % yield).

R_f = 0.6 (*n*-hexane/EtOAc 10:1 (v/v)), ^{1}H NMR (400 MHz, $CDCl_3$) δ 7.61 (d, *J* = 8.2 Hz, 1H), 7.44 (d, *J* = 8.1 Hz, 1H), 3.12 (t, *J* = 6.4 Hz, 2H), 2.18 (s, 3H), 1.77 (m, 2H), 1.68 – 1.57 (m, 2H), 1.29 (s, 6H). ^{13}C NMR (101 MHz, $CDCl_3$) δ 182.3, 175.1, 155.9, 150.9, 146.8, 145.1, 133.7, 127.0, 126.3, 120.4 (q, *J* = 262.6 Hz), 120.3, 120.0, 106.0, 37.8, 34.8, 31.9, 30.1, 19.1, 7.3. ^{19}F NMR (376 MHz, $CDCl_3$) δ -59.95 (s, 3F). Mass Spectrometry: HRMS-ESI (m/z): Calcd for $C_{20}H_{17}F_3NaO_4$ $[M + Na]^+$, 401.0971. Found, 401.0975.

5,7-Diisopropoxy-2-(4-isopropoxyphenyl)-3-(trifluoromethoxy)-4H-chromen-4-one (4ss)

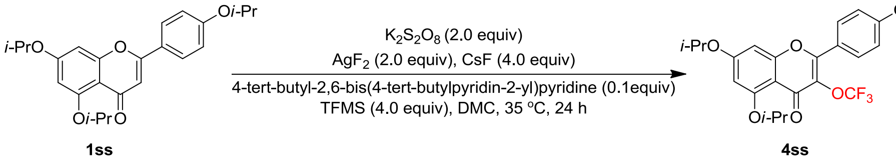

The reaction was performed according to the general procedure C using 5,7-diisopropoxy-2-(4-isopropoxyphenyl)-4H-chromen-4-one[12] (**1ss**) (198.2 mg, 0.50 mmol, 1.0 eq), $K_2S_2O_8$ (270 mg, 1.0 mmol, 2.00 equiv), AgF_2 (145 mg, 1.0 mmol, 2.00 equiv). After 24 hr, the reaction mixture was filtered through a short plug of silica gel eluting with approximately 25 mL of EtOAc and the filtrate was concentrated *in vacuo*. The residue was purified by flash column chromatography on silica eluting with hexanes/ EtOAc 5:1 (v/v) to afford 182.6 mg 5,7-diisopropoxy-2-(4-isopropoxyphenyl)-3-(trifluoromethoxy)-4H-chromen-4-one (**4ss**) as a yellow solid (76 % yield).

R_f = 0.3 (*n*-hexane/EtOAc 5:1 (v/v)), ^{1}H NMR (400 MHz, $CDCl_3$) δ 7.85 (d, *J* = 8.3 Hz, 2H), 6.95 (d, *J* = 7.5 Hz, 2H), 6.45 (s, 1H), 6.33 (s, 1H), 4.74 – 4.44 (m, 3H), 1.42 (d, *J* = 6.0 Hz, 3H), 1.37 – 1.33 (m, 6H). ^{13}C NMR (101 MHz, $CDCl_3$) δ 170.9, 162.7, 160.4, 159.8, 158.9, 156.1, 131.8, 130.2, 121.3, 121.2 (q, *J* = 262.6 Hz), 115.5, 109.6, 100.5, 94.1, 72.5, 70.8, 70.1, 21.9, 21.8, 21.8. ^{19}F NMR (376 MHz, $CDCl_3$) δ -57.36 (s, 3F). Mass Spectrometry: HRMS-ESI (m/z): Calcd for $C_{25}H_{28}F_3O_6$ $[M + H]^+$, 481.1832. Found, 481.1836.

Isopropyl 2-(4-(4-chlorobenzoyl)-2-(trifluoromethoxy)phenoxy)-2-methylpro panoate (4tt)

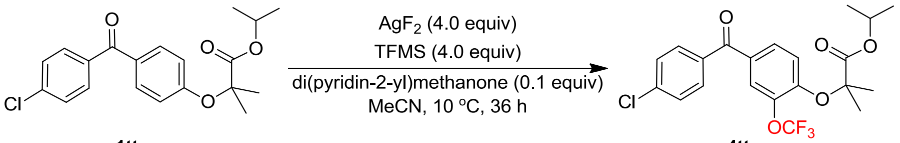

1tt **4tt**

The reaction was performed according to the general procedure D using isopropyl 2-(4-(4-chlorobenzoyl)phenoxy)-2-methylpropanoate (**1tt**) (180.4 mg, 0.50 mmol) as the substrate. After 36 hr, the reaction mixture was filtered through a short plug of silica gel eluting with approximately 25 mL of EtOAc and the filtrate was concentrated *in vacuo*. The residue was purified by flash column chromatography on silica eluting with hexanes/ EtOAc 20:1 (v/v) to afford 77.8 mg isopropyl 2-(4-(4-chlorobenzoyl)-2-(trifluoromethoxy)phenoxy)-2-methylpropanoate (**4tt**) as a white solid (35 % yield).

R_f= 0.5 (*n*-hexane/EtOAc 20:1 (v/v)), ^{1}H NMR (400 MHz, $CDCl_3$) δ 7.73 (s, 1H), 7.69 (d, *J* = 6.8 Hz, 2H), 7.63 (d, *J* = 8.7 Hz, 1H), 7.46 (d, *J* = 6.5 Hz, 2H), 6.86 (d, *J* = 9.4 Hz, 1H), 5.15 – 4.95 (m, 1H), 1.67 (s, 6H), 1.18 (d, *J* = 6.2 Hz, 6H). ^{13}C NMR (101 MHz, $CDCl_3$) δ 193.0, 172.6, 152.6, 139.3, 139.0, 135.7, 131.3, 130.5, 129.9, 128.9, 125.6, 120.7 (q, *J* = 262.6 Hz), 116.9, 80.9, 69.7, 25.2, 21.6. ^{19}F NMR (376 MHz, $CDCl_3$) δ -58.53 (s, 3F). Mass Spectrometry: HRMS-ESI (m/z): Calcd for $C_{21}H_{21}ClF_3O_5$ $[M + H]^+$, 445.1024. Found, 445.1026.

Gram scale synthesis of 4-((Trifluoromethoxy)methyl)phenyl benzoate (3p)

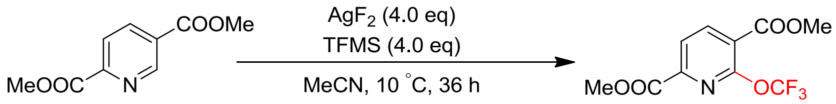

1p **3p**

In a glove box, to a 30.0 mL sealed tube were added in sequence dimethyl pyridine-2,5-dicarboxylate (1.46 g, 7.50 mmol, 1.00 equiv), 3.75 mL MeCN (the solvent was pre-cooled to -30 °C) and TFMS (4.8 mL, 30.0 mmol, 4.00 equiv), then AgF_2 (4.35 g, 30.0 mmol, 4.00 equiv) was added at once in one portion. The mixture was stirred at 10 ºC for 36 hr. After warming up to 23 °C, the reaction mixture was filtered through a short plug of silica gel eluting with approximately 400 mL EtOAc, and the filtrate was concentrated *in vacuo*. The residue was purified by silica gel column chromatography, eluting with hexanes/ EtOAc 2:1 (v/v) to afford 1.61 g dimethyl 6-(trifluoromethoxy)pyridine-2,5-dicarboxylate (**3p**) as a white solid (75 % yield).

Gram scale synthesis of 2-chloro-6-methoxy-5-(trifluoromethoxy)quinoline (3ff)

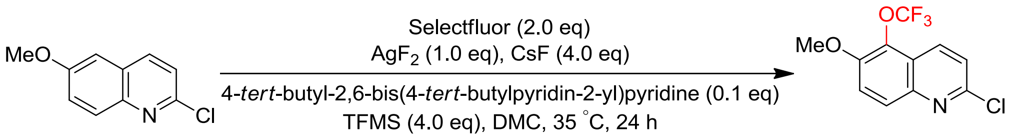

1ff **3ff**

In a glove box, to a 200.0 mL sealed tube were added in sequence 2-chloro-6-methoxyquinoline (1.16 g, 6.0 mmol, 1.00 equiv), Selectfluor (4.25 g, 12.0 mmol, 2.00 equiv), AgF_2 (864 mg, 6.0 mmol, 1.00 equiv), CsF (3.65 g, 24.0 mmol, 4.00 equiv), 4-*tert*-butyl-2,6-bis(4-*tert*-butylpyridin-2-yl)pyridine (240 mg, 0.60 mmol, 0.10 equiv), 120.0 mL

DMC and TFMS (3.84 mL, 24.0 mmol, 4.00 equiv). The mixture was stirred at 35 ºC for 24 hr. After cooling to 23 °C, the reaction mixture was filtered through a short plug of silica gel eluting with approximately 500 mL of EtOAc. The filtrate was concentrated, and the residue was purified by preparative TLC, eluting with hexanes/ EtOAc 10:1 (v/v) to afford 1.21 g 2-chloro-6-methoxy-5-(trifluoromethoxy)quinolone (**3ff**) as a light yellow solid (73 % yield).

Mechanism Studies

H/D isotope scrambling

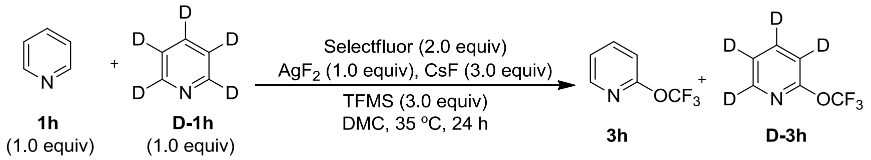

In a glove box, to a 2.0 mL sealed tube were added in sequence Selectfluor (35.4 mg, 0.10 mmol, 2.00 equiv), AgF_2 (7.2 mg, 0.05 mmol, 1.00 equiv), CsF (22.8 mg, 0.15 mmol, 3.00 equiv), Pyridine (**1h**) (4.0 mg, 0.05 mmol, 1.00 equiv) in 0.5 mL DMC, Pyridine-d_5 (**D-1h**) (4.2 mg, 0.05 mmol, 1.00 equiv) in 0.5 mL DMC and TFMS (24 µL, 0.15 mmol, 3.00 equiv). The mixture was then stirred at 35 ºC for 24 hr. After cooling to 23 °C, the mixture was diluted with 1.0 mL $CDCl_3$, then benzotrifluoride (6 µL, 0.05 mmol) was added as an internal standard, and the reaction mixture was analyzed by the ^{19}F NMR. The ratio of **3h**:**D-3h** was determined by ^{19}F NMR to be 2.1:1.

Kinetic H/D isotope effect analysis

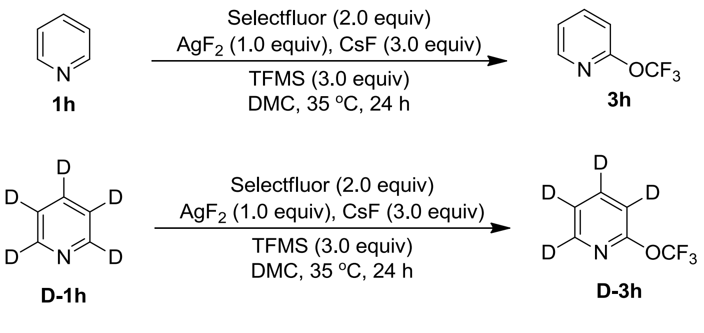

In a glove box, to a 2.0 mL sealed tube were added in sequence Selectfluor (35.4 mg, 0.10 mmol, 2.00 equiv), AgF_2 (7.2 mg, 0.05 mmol, 1.00 equiv), CsF (22.8 mg, 0.15 mmol, 3.00 equiv), Pyridine (**1h**) (4.0 mg, 0.05 mmol, 1.00 equiv) or Pyridine-d_5 (**D-1h**) (4.2 mg, 0.05 mmol, 1.00 equiv) in 1.0 mL DMC and TFMS (24 µL, 0.15 mmol, 3.00 equiv). The mixture was then stirred at 35 ºC. After the indicated time, benzotrifluoride (6 µL, 0.05 mmol) was added as an internal standard, and the reaction mixture was analyzed by the ^{19}F NMR. The yield of 2-(trifluoromethoxy)pyridine (**3h**) or 2-(trifluoromethoxy)pyridine-d_5 (**D-3h**)was plotted as a

function of time. Yields reflect an average of three independent trials (Supplementary Table 10 and 11). An isotope effect (k_H/k_D = 1.83:1) was obtained by comparing the two independent initial reaction rates for trifluoromethoxylation of Pyridine and Pyridine-d_5 (Figure S1).

Supplementary Table 10: Kinetic data obtained for pyridine

time (min)	yield of pyridine (%)			
	trial 1	trial 2	trial 3	average
15	2.3	3.2	2.5	2.7
30	6.2	6.3	7.0	6.5
50	8.1	9.6	6.9	8.2
70	12.9	12.6	11.0	12.2
90	12.0	12.5	14.0	12.8
110	19.9	22.1	20.5	20.8
140	26.1	24.6	28.1	26.3

Supplementary Table 11: Kinetic data obtained for pyridine-d_5.

time (min)	yield of pyridine-d_5 (%)			
	trial 1	trial 2	trial 3	average
15	0	0.1	0.6	0.2
30	2.3	2.2	2.2	2.2
50	2.7	3.6	3.4	3.2
70	5.0	4.3	4.9	4.7
90	4.7	7.1	7.5	6.4
110	10.9	11.0	8.2	10.0
140	11.0	12.8	14.9	12.9

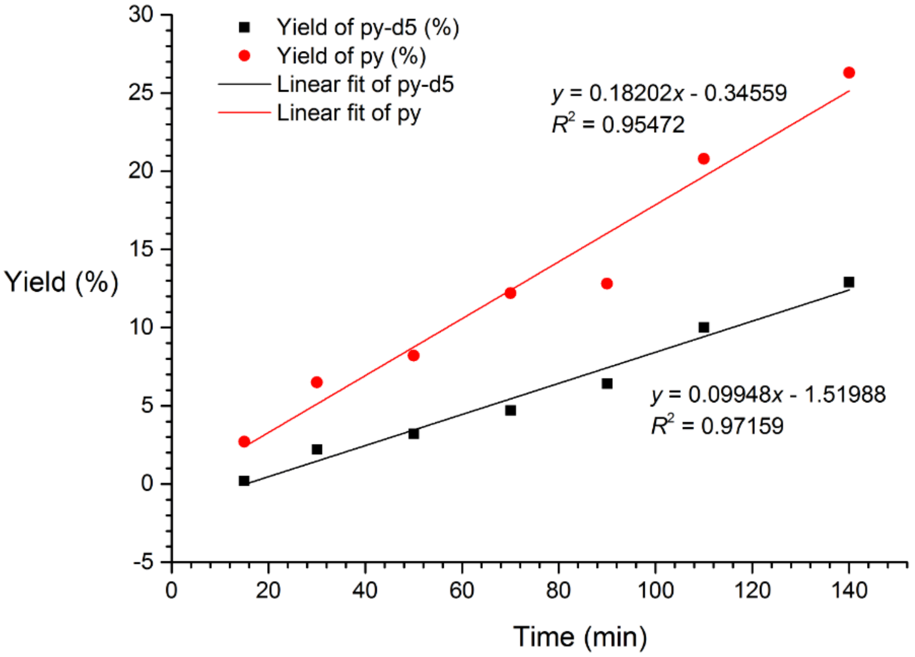

Supplementary Figure 1. Initial rates for trifluoromethoxylation of pyridine and pyridine-d_5

Reaction of N-Fluoropyridinium salt with or without Selectfluor

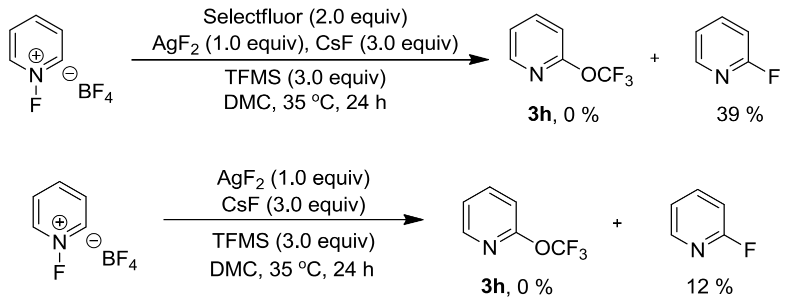

In a glove box, to a 2.0 mL sealed tube were added in sequence 1-fluoropyridin-1-ium tetrafluoroborate (9.3 mg, 0.05 mmol, 1.00 equiv), with or without Selectfluor (35.4 mg, 0.10 mmol, 2.00 equiv), AgF_2 (7.2 mg, 0.05 mmol, 1.00 equiv), CsF (22.8 mg, 0.15 mmol, 3.00 equiv), 1.0 mL DMC and TFMS (24 µL, 0.15 mmol, 3.00 equiv). The mixture was stirred at 35 ºC for 24 hr. After cooling to 23 °C, benzotrifluoride (6 µL, 0.05 mmol) was added. The yield of 2-(trifluoromethoxy)pyridine (**3h**) and 2-fluoropyridine was determined by comparing the integration of the ^{19}F NMR resonance with benzotrifluoride (-62.80 ppm).

Reaction of 2-Fluoropyridine with 1.0 eq AgF_2 in DMC

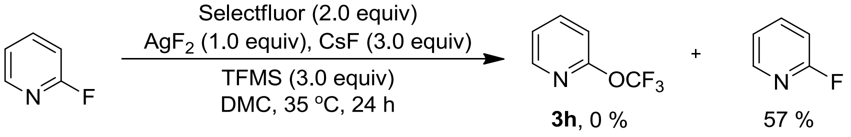

In a glove box, to a 2.0 mL sealed tube were added in sequence Selectfluor (35.4 mg, 0.10 mmol, 2.00 equiv), AgF_2 (7.2 mg, 0.05 mmol, 1.00 equiv), CsF (22.8 mg, 0.15 mmol, 3.00 equiv),

2-fluoropyridine (48.5 mg, 0.05 mmol, 1.00 equiv) in 1.0 mL DMC and TFMS (24 μL, 0.15 mmol, 3.00 equiv). The mixture was stirred at 35 ºC for 24 hr. After cooling to 23 °C, benzotrifluoride (6 μL, 0.05 mmol) was added. The yield of 2-(trifluoromethoxy)pyridine (**3h**) was 0 % and 2-fluoropyridine was 57 % remained by comparing the integration of the ^{19}F NMR resonance with benzotrifluoride (-62.80 ppm).

Reaction of 2-Fluoropyridine with 4.0 eq AgF_2 in MeCN.

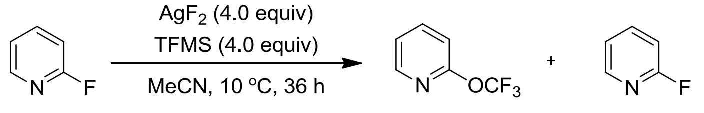

3h, 0 %   39 %

In a glove box, to a 4.0 mL sealed tube were added in sequence 2-fluoropyridine (48.5 mg, 0.5 mmol, 1.00 equiv), 0.50 mL MeCN (the solvent was pre-cooled to -30 °C) and TFMS (320 μL, 2.00 mmol, 4.00 equiv), then AgF_2 (290 mg, 2.00 mmol, 4.00 equiv) was added at once in one portion. The mixture was stirred at 10 ºC for 36 hr. After warming up to 23 °C, benzotrifluoride (60 μL, 0.50 mmol) was added. The yield of 2-(trifluoromethoxy)pyridine (**3h**) was 0 % and 2-fluoropyridine was 39 % remained by comparing the integration of the ^{19}F NMR resonance with benzotrifluoride (-62.80 ppm).

Reaction Conditions for the S_NAr of 2-Fluoropyridine

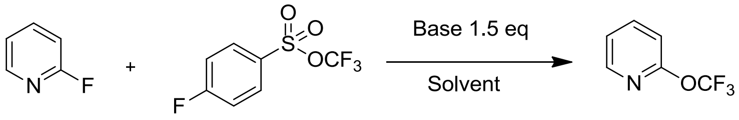

TFMS, 1.5 eq   **3h**

The reaction Conditions for the S_NAr of 2-Fluoropyridine was followed the procedure of John F. Hartwig et al.[11] 2-Fluoropyridine (19.4 mg, 0.20 mmol, 1.00 equiv) was dissolved in 1 mL of Solvent and the TFMS (38.4 μL , 0.24 mmol, 1.2 equiv) was added followed by the base (0.24 mmol, 1.2 equiv). The vial was sealed with a Teflon-lined cap, and stirred at different temperatures for 3h. After cooled to 23 °C, benzotrifluoride (60 μL, 0.50 mmol) was added. The yield of 2-(trifluoromethoxy)pyridine (**3h**) was determined by comparing the integration of the ^{19}F NMR resonance with benzotrifluoride (-62.80 ppm). Yields are reported in Supplementary Table 12.

Supplementary Table 12: Reaction conditions for the S_NAr of 2-Fluoropyridine

Solvent	Base	Temperature	Yield [%] (^{19}F NMR)
THF	CsF	50 ºC	0
THF	KO^{t}Bu	50 ºC	0
THF	^{i}Pr$_2$NEt	50 ºC	0
DMF	CsF	80 ºC	0
DMF	KO^{t}Bu	80 ºC	0
DMF	NaH	100 ºC	0
DMSO	^{i}Pr$_2$NEt	120 ºC	0

Reaction with AgOTf instead of AgF$_2$

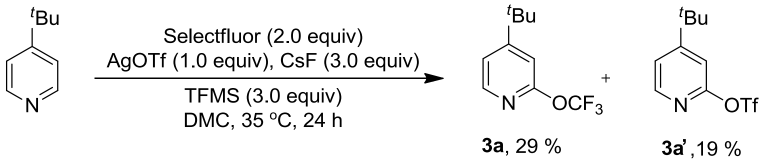

In a glove box, to a 2.0 mL sealed tube were added in sequence Selectfluor (35.4 mg, 0.10 mmol, 2.00 equiv), AgOTf (12.8 mg, 0.05 mmol, 1.00 equiv), CsF (22.8 mg, 0.15 mmol, 3.00 equiv), 4-(*tert*-butyl)pyridine (6.7 mg, 0.050 mmol, 1.00 equiv) in 1.0 mL DMC and TFMS (24 μL, 0.15 mmol, 3.00 equiv). The mixture was stirred at 35 ºC for 24 hr. After cooling to 23 °C, benzotrifluoride (6 μL, 0.05 mmol) was added. The yield of 4-(*tert*-butyl)-2-(trifluoromethoxy)pyridine (**3a**) was 29 % and 4-(*tert*-butyl)pyridin-2-yl trifluoromethanesulfonate (**3a**') was 19 % remained by comparing the integration of the ^{19}F NMR resonance with benzotrifluoride (-62.80 ppm) and GC-MS.

Mechanism studies using radical inhibitor

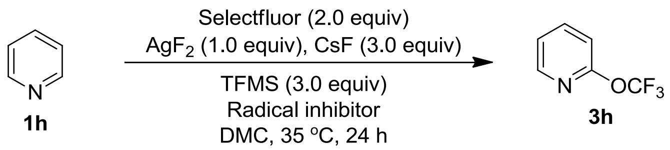

In a glove box, to a 2.0 mL sealed tube were added in sequence Selectfluor (35.4 mg, 0.10 mmol, 2.00 equiv), AgF$_2$ (7.2 mg, 0.05 mmol, 1.00 equiv), CsF (22.8 mg, 0.15 mmol, 3.00 equiv), the radical inhibitor, pyridine (**1h**) (4.0 mg, 0.05 mmol, 1.00 equiv) in 1.0 mL DMC and TFMS (24 μL, 0.15 mmol, 3.00 equiv). The mixture was stirred at 35 ºC for 24 hr. After cooling to 23 °C, benzotrifluoride (6 μL, 0.05 mmol) was added. The yield of 2-(trifluoromethoxy)pyridine (**3h**) was determined by comparing the integration of the ^{19}F NMR resonance of 2-(trifluoromethoxy)pyridine (**3h**) (-56.78 ppm) with that of benzotrifluoride (-62.80 ppm). Yields are reported in Supplementary Table 13.

Supplementary Table 13: Effect of radical inhibitors on the reaction

Radical inhibitors	Yield [%] (^{19}F NMR)
2,6-di-tert-butyl-4-methylphenol (BHT) (0.5 equiv)	31
2,6-di-tert-butyl-4-methylphenol (BHT) (1.0 equiv)	23
2,6-di-tert-butyl-4-methylphenol (BHT) (2.0 equiv)	14
2,6-di-tert-butyl-4-methylphenol (BHT) (4.0 equiv)	0
TEMPO (0.5 equiv)	25
TEMPO (1.0 equiv)	0

EPR spectra

Electron paramagnetic resonance (EPR) spectra were recorded on a Bruker ELEXSYS E580 spectrometer (X-band). The solution was measured with microwave frequency of about 9.85 GHz, microwave power of 1.5 mW, modulation amplitude of 0.5 G, a time constant of 12 ms. α-(4-pyridyl *N*-oxide)-*N*-tert-butylnitrone (POBN) was employed as the radical trapper.

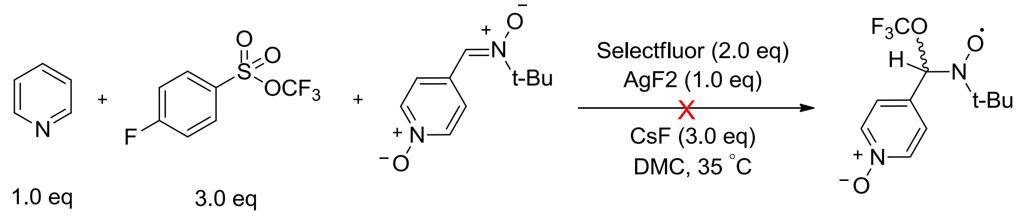

In a glove box, to a 2.0 mL sealed tube were added in sequence Selectfluor (35.4 mg, 0.10 mmol, 2.00 equiv), AgF_2 (7.2 mg, 0.05 mmol, 1.00 equiv), CsF (22.8 mg, 0.15 mmol, 3.00 equiv), pyridine (**1h**) (4.0 mg, 0.05 mmol, 1.00 equiv) in 1.0 mL DMC and TFMS (24 μL, 0.15 mmol, 3.00 equiv). The mixture was stirred at 35 ºC for 3 hr. Then 0.4 mL reaction mixture in a 2 mL tube was added 1.0 mL POBN (0.1 mol/L in DMC) under N_2.

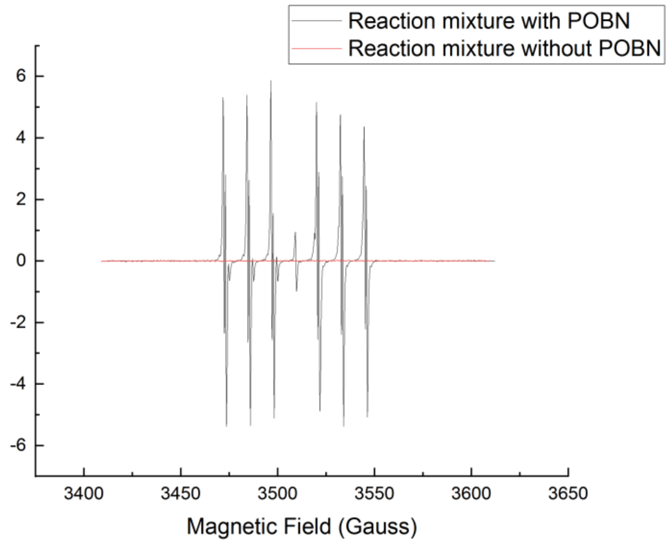

Supplementary Figure 2. The EPR spectra of our reaction with or without POBN

The EPR spectra of our reaction didn't match previously reported POBN-OCF_3 adducts.[13]

X-ray Crystal Structure Data for 5-chloro-6'-methyl-3-(4-(methyl sulfonyl)phenyl)-2'-(trifluoromethoxy)-2,3'-bipyridine (4kk) (CCDC: 1954409)

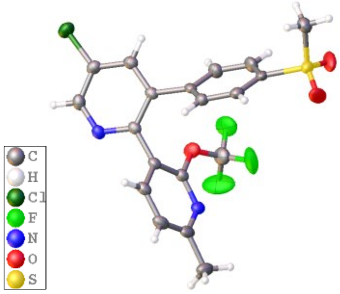

Supplementary Table 14: Crystal data and structure refinement for **4kk**.

Identification code	**4kk**
CCDC	1954409
Empirical formula	C19 H14 Cl F3 N2 O3 S
Formula weight	442.83
Temperature	113(2) K
Wavelength	0.71073 A
Crystal system, space group	Triclinic, P-1
Unit cell dimensions	a = 11.602(2) A alpha = 100.05<br>b = 11.679(2) A beta = 106.36(<br>c = 16.134(3) A gamma = 108.7
Volume	1899.3(8) A^3
Z, Calculated density	4, 1.549 Mg/m^3
Absorption coefficient	0.364 mm^-1
F(000)	904
Crystal size	0.200 x 0.180 x 0.120 mm
Theta range for data collection	1.375 to 27.827 deg.
Limiting indices	-14<=h<=15, -15<=k<=15, -21<=
Reflections collected / unique	22641 / 8975 [R(int) = 0.0577]
Completeness to theta = 25.242	99.7 %
Absorption correction	Semi-empirical from equivalents
Max. and min. transmission	1 and 0.8031
Refinement method	Full-matrix least-squares on F^2
Data / restraints / parameters	8975 / 72 / 555
Goodness-of-fit on F^2	1.035
Final R indices [I>2sigma(I)]	R1 = 0.0451, wR2 = 0.0953
R indices (all data)	R1 = 0.0741, wR2 = 0.1037
Extinction coefficient	n/a

Supplementary Table 15: Atomic coordinates (x 10^4) and equivalent isotropic displacement parameters (A^2 x 10^3) for **4kk**. U(eq) is defined as one third of the trace of the orthogonalized Uij tensor.

	x	y	z	U(eq)
S(1)	4454(1)	7862(1)	10222(1)	26(1)
S(2)	6202(1)	812(1)	7103(1)	43(1)
Cl(1)	4542(1)	2802(1)	4964(1)	35(1)
Cl(2)	12047(1)	8765(1)	7519(1)	43(1)
F(1)	10200(3)	6121(4)	9663(4)	58(1)
F(2)	8859(7)	6719(3)	10083(3)	69(2)
F(3)	8295(13)	4756(6)	9227(11)	52(2)
F(1')	9999(11)	6457(12)	10049(8)	100(5)
F(2')	8049(13)	6438(10)	9637(9)	66(3)
F(3')	8200(30)	4812(15)	9350(20)	56(5)
F(4)	11079(2)	1415(2)	7494(1)	94(1)
F(5)	12256(2)	3123(2)	8579(1)	63(1)
F(6)	13137(2)	2229(2)	7829(1)	89(1)
O(1)	8679(2)	6220(2)	8608(1)	46(1)
O(2)	5455(2)	9100(2)	10715(1)	34(1)
O(3)	4234(2)	6911(2)	10690(1)	40(1)
O(4)	11945(2)	3217(2)	7229(1)	36(1)
O(5)	5640(2)	-244(2)	6306(2)	76(1)
O(6)	6704(2)	612(2)	7975(1)	60(1)
N(1)	10090(2)	8351(2)	9163(1)	29(1)
N(2)	7515(2)	6016(2)	6430(1)	27(1)
N(3)	11732(2)	1693(2)	5989(1)	32(1)
N(4)	12179(2)	5746(2)	6066(1)	28(1)
C(1)	11668(2)	10524(2)	9744(2)	43(1)
C(2)	10376(2)	9533(2)	9080(2)	29(1)
C(3)	9553(2)	9796(2)	8407(2)	32(1)
C(4)	8465(2)	8828(2)	7756(2)	27(1)
C(5)	8171(2)	7588(2)	7807(1)	22(1)
C(6)	9012(2)	7467(2)	8551(2)	26(1)
C(7)	8928(3)	5991(2)	9392(2)	40(1)
C(8)	7126(2)	6446(2)	7089(1)	22(1)
C(9)	6695(2)	4936(2)	5784(2)	27(1)
C(10)	5490(2)	4238(2)	5792(1)	26(1)
C(11)	5068(2)	4673(2)	6458(1)	26(1)
C(12)	5897(2)	5820(2)	7126(1)	22(1)
C(13)	5487(2)	6319(2)	7862(1)	23(1)
C(14)	5124(2)	5575(2)	8406(2)	31(1)
C(15)	4782(2)	6042(2)	9111(2)	33(1)
C(16)	4797(2)	7247(2)	9276(1)	22(1)
C(17)	5141(2)	8000(2)	8731(1)	23(1)
C(18)	5480(2)	7529(2)	8026(1)	22(1)
C(19)	2979(2)	8028(3)	9758(2)	42(1)

C(20)	11644(3)	90(2)	4767(2)	48(1)
C(21)	11441(2)	1278(2)	5091(2)	34(1)
C(22)	11033(2)	1938(2)	4521(2)	35(1)
C(23)	10966(2)	3069(2)	4874(2)	30(1)
C(24)	11248(2)	3510(2)	5793(1)	24(1)
C(25)	11612(2)	2753(2)	6294(1)	26(1)
C(26)	12108(3)	2480(3)	7767(2)	46(1)
C(27)	11313(2)	4784(2)	6212(1)	23(1)
C(28)	12371(2)	6924(2)	6467(1)	29(1)
C(29)	11728(2)	7202(2)	7022(1)	28(1)
C(30)	10806(2)	6220(2)	7157(2)	29(1)
C(31)	10572(2)	4983(2)	6738(1)	23(1)
C(32)	9517(2)	3915(2)	6823(2)	24(1)
C(33)	8463(2)	3075(2)	6062(2)	28(1)
C(34)	7435(2)	2132(2)	6134(2)	31(1)
C(35)	7493(2)	2017(2)	6987(2)	30(1)
C(36)	8542(3)	2831(2)	7751(2)	39(1)
C(37)	9551(2)	3788(2)	7674(2)	34(1)
C(38)	5046(2)	1476(2)	7133(2)	40(1)

Supplementary Table 16: Bond lengths [A] and angles [deg] for **4kk**.

S(1)-O(2)	1.4384(18)
S(1)-O(3)	1.4454(17)
S(1)-C(19)	1.752(2)
S(1)-C(16)	1.775(2)
S(2)-O(5)	1.434(2)
S(2)-O(6)	1.453(2)
S(2)-C(38)	1.759(3)
S(2)-C(35)	1.776(2)
Cl(1)-C(10)	1.735(2)
Cl(2)-C(29)	1.732(2)
F(1)-C(7)	1.365(4)
F(2)-C(7)	1.317(4)
F(3)-C(7)	1.328(6)
F(1')-C(7)	1.258(7)
F(2')-C(7)	1.402(6)
F(3')-C(7)	1.345(9)
F(4)-C(26)	1.313(3)
F(5)-C(26)	1.326(3)
F(6)-C(26)	1.301(3)
O(1)-C(7)	1.311(3)
O(1)-C(6)	1.409(3)

O(4)-C(26)	1.342(3)
O(4)-C(25)	1.403(3)
N(1)-C(6)	1.313(3)
N(1)-C(2)	1.352(3)
N(2)-C(9)	1.338(3)
N(2)-C(8)	1.347(3)
N(3)-C(25)	1.317(3)
N(3)-C(21)	1.350(3)
N(4)-C(28)	1.331(3)
N(4)-C(27)	1.352(3)
C(1)-C(2)	1.509(3)
C(1)-H(1A)	0.9800
C(1)-H(1B)	0.9800
C(1)-H(1C)	0.9800
C(2)-C(3)	1.381(3)
C(3)-C(4)	1.376(3)
C(3)-H(3)	0.9500
C(4)-C(5)	1.400(3)
C(4)-H(4)	0.9500
C(5)-C(6)	1.378(3)
C(5)-C(8)	1.490(3)
C(8)-C(12)	1.402(3)
C(9)-C(10)	1.379(3)
C(9)-H(9)	0.9500
C(10)-C(11)	1.384(3)
C(11)-C(12)	1.402(3)
C(11)-H(11)	0.9500
C(12)-C(13)	1.494(3)
C(13)-C(14)	1.394(3)
C(13)-C(18)	1.395(3)
C(14)-C(15)	1.387(3)
C(14)-H(14)	0.9500
C(15)-C(16)	1.379(3)
C(15)-H(15)	0.9500
C(16)-C(17)	1.395(3)
C(17)-C(18)	1.387(3)
C(17)-H(17)	0.9500
C(18)-H(18)	0.9500
C(19)-H(19A)	0.9800
C(19)-H(19B)	0.9800
C(19)-H(19C)	0.9800
C(20)-C(21)	1.509(3)
C(20)-H(20A)	0.9800
C(20)-H(20B)	0.9800

C(20)-H(20C)	0.9800
C(21)-C(22)	1.378(4)
C(22)-C(23)	1.380(3)
C(22)-H(22)	0.9500
C(23)-C(24)	1.386(3)
C(23)-H(23)	0.9500
C(24)-C(25)	1.383(3)
C(24)-C(27)	1.491(3)
C(27)-C(31)	1.408(3)
C(28)-C(29)	1.377(3)
C(28)-H(28)	0.9500
C(29)-C(30)	1.391(3)
C(30)-C(31)	1.386(3)
C(30)-H(30)	0.9500
C(31)-C(32)	1.498(3)
C(32)-C(33)	1.388(3)
C(32)-C(37)	1.396(3)
C(33)-C(34)	1.387(3)
C(33)-H(33)	0.9500
C(34)-C(35)	1.390(3)
C(34)-H(34)	0.9500
C(35)-C(36)	1.380(3)
C(36)-C(37)	1.387(3)
C(36)-H(36)	0.9500
C(37)-H(37)	0.9500
C(38)-H(38A)	0.9800
C(38)-H(38B)	0.9800
C(38)-H(38C)	0.9800
O(2)-S(1)-O(3)	118.52(11)
O(2)-S(1)-C(19)	108.18(12)
O(3)-S(1)-C(19)	108.44(12)
O(2)-S(1)-C(16)	108.58(10)
O(3)-S(1)-C(16)	107.48(10)
C(19)-S(1)-C(16)	104.81(11)
O(5)-S(2)-O(6)	118.65(14)
O(5)-S(2)-C(38)	108.68(14)
O(6)-S(2)-C(38)	108.36(12)
O(5)-S(2)-C(35)	108.21(12)
O(6)-S(2)-C(35)	107.75(13)
C(38)-S(2)-C(35)	104.25(11)
C(7)-O(1)-C(6)	121.0(2)
C(26)-O(4)-C(25)	120.6(2)
C(6)-N(1)-C(2)	116.1(2)
C(9)-N(2)-C(8)	118.59(19)

C(25)-N(3)-C(21)	117.1(2)
C(28)-N(4)-C(27)	118.24(19)
C(2)-C(1)-H(1A)	109.5
C(2)-C(1)-H(1B)	109.5
H(1A)-C(1)-H(1B)	109.5
C(2)-C(1)-H(1C)	109.5
H(1A)-C(1)-H(1C)	109.5
H(1B)-C(1)-H(1C)	109.5
N(1)-C(2)-C(3)	121.6(2)
N(1)-C(2)-C(1)	115.8(2)
C(3)-C(2)-C(1)	122.6(2)
C(4)-C(3)-C(2)	120.0(2)
C(4)-C(3)-H(3)	120.0
C(2)-C(3)-H(3)	120.0
C(3)-C(4)-C(5)	119.4(2)
C(3)-C(4)-H(4)	120.3
C(5)-C(4)-H(4)	120.3
C(6)-C(5)-C(4)	114.8(2)
C(6)-C(5)-C(8)	120.5(2)
C(4)-C(5)-C(8)	124.5(2)
N(1)-C(6)-C(5)	127.7(2)
N(1)-C(6)-O(1)	118.1(2)
C(5)-C(6)-O(1)	114.1(2)
F(1')-C(7)-O(1)	127.8(6)
O(1)-C(7)-F(2)	120.5(3)
O(1)-C(7)-F(3)	106.6(7)
F(2)-C(7)-F(3)	115.6(8)
F(1')-C(7)-F(3')	112.0(15)
O(1)-C(7)-F(3')	113.8(16)
O(1)-C(7)-F(1)	105.5(3)
F(2)-C(7)-F(1)	104.5(3)
F(3)-C(7)-F(1)	102.1(7)
F(1')-C(7)-F(2')	108.6(7)
O(1)-C(7)-F(2')	95.1(5)
F(3')-C(7)-F(2')	90.2(17)
N(2)-C(8)-C(12)	123.1(2)
N(2)-C(8)-C(5)	113.12(19)
C(12)-C(8)-C(5)	123.64(19)
N(2)-C(9)-C(10)	121.9(2)
N(2)-C(9)-H(9)	119.0
C(10)-C(9)-H(9)	119.0
C(9)-C(10)-C(11)	120.2(2)
C(9)-C(10)-Cl(1)	118.33(17)
C(11)-C(10)-Cl(1)	121.44(18)

C(10)-C(11)-C(12)	118.7(2)
C(10)-C(11)-H(11)	120.6
C(12)-C(11)-H(11)	120.6
C(8)-C(12)-C(11)	117.4(2)
C(8)-C(12)-C(13)	122.0(2)
C(11)-C(12)-C(13)	120.54(19)
C(14)-C(13)-C(18)	119.1(2)
C(14)-C(13)-C(12)	119.7(2)
C(18)-C(13)-C(12)	121.17(19)
C(15)-C(14)-C(13)	120.3(2)
C(15)-C(14)-H(14)	119.8
C(13)-C(14)-H(14)	119.8
C(16)-C(15)-C(14)	120.1(2)
C(16)-C(15)-H(15)	120.0
C(14)-C(15)-H(15)	120.0
C(15)-C(16)-C(17)	120.5(2)
C(15)-C(16)-S(1)	119.32(17)
C(17)-C(16)-S(1)	120.13(16)
C(18)-C(17)-C(16)	119.3(2)
C(18)-C(17)-H(17)	120.3
C(16)-C(17)-H(17)	120.3
C(17)-C(18)-C(13)	120.7(2)
C(17)-C(18)-H(18)	119.7
C(13)-C(18)-H(18)	119.7
S(1)-C(19)-H(19A)	109.5
S(1)-C(19)-H(19B)	109.5
H(19A)-C(19)-H(19B)	109.5
S(1)-C(19)-H(19C)	109.5
H(19A)-C(19)-H(19C)	109.5
H(19B)-C(19)-H(19C)	109.5
C(21)-C(20)-H(20A)	109.5
C(21)-C(20)-H(20B)	109.5
H(20A)-C(20)-H(20B)	109.5
C(21)-C(20)-H(20C)	109.5
H(20A)-C(20)-H(20C)	109.5
H(20B)-C(20)-H(20C)	109.5
N(3)-C(21)-C(22)	121.2(2)
N(3)-C(21)-C(20)	115.8(2)
C(22)-C(21)-C(20)	123.0(2)
C(21)-C(22)-C(23)	119.8(2)
C(21)-C(22)-H(22)	120.1
C(23)-C(22)-H(22)	120.1
C(22)-C(23)-C(24)	120.1(2)
C(22)-C(23)-H(23)	119.9

C(24)-C(23)-H(23)	119.9
C(25)-C(24)-C(23)	115.0(2)
C(25)-C(24)-C(27)	122.9(2)
C(23)-C(24)-C(27)	121.7(2)
N(3)-C(25)-C(24)	126.7(2)
N(3)-C(25)-O(4)	117.9(2)
C(24)-C(25)-O(4)	115.32(19)
F(6)-C(26)-F(4)	109.1(3)
F(6)-C(26)-F(5)	108.5(2)
F(4)-C(26)-F(5)	108.5(2)
F(6)-C(26)-O(4)	112.8(2)
F(4)-C(26)-O(4)	111.5(3)
F(5)-C(26)-O(4)	106.2(2)
N(4)-C(27)-C(31)	122.5(2)
N(4)-C(27)-C(24)	114.03(18)
C(31)-C(27)-C(24)	123.49(19)
N(4)-C(28)-C(29)	122.9(2)
N(4)-C(28)-H(28)	118.5
C(29)-C(28)-H(28)	118.5
C(28)-C(29)-C(30)	119.3(2)
C(28)-C(29)-Cl(2)	119.67(18)
C(30)-C(29)-Cl(2)	120.98(17)
C(31)-C(30)-C(29)	119.0(2)
C(31)-C(30)-H(30)	120.5
C(29)-C(30)-H(30)	120.5
C(30)-C(31)-C(27)	117.9(2)
C(30)-C(31)-C(32)	119.68(19)
C(27)-C(31)-C(32)	122.38(19)
C(33)-C(32)-C(37)	119.2(2)
C(33)-C(32)-C(31)	120.44(19)
C(37)-C(32)-C(31)	120.3(2)
C(34)-C(33)-C(32)	121.1(2)
C(34)-C(33)-H(33)	119.4
C(32)-C(33)-H(33)	119.4
C(33)-C(34)-C(35)	118.7(2)
C(33)-C(34)-H(34)	120.6
C(35)-C(34)-H(34)	120.6
C(36)-C(35)-C(34)	120.9(2)
C(36)-C(35)-S(2)	119.12(19)
C(34)-C(35)-S(2)	119.94(19)
C(35)-C(36)-C(37)	119.9(2)
C(35)-C(36)-H(36)	120.0
C(37)-C(36)-H(36)	120.0
C(36)-C(37)-C(32)	120.1(2)

C(36)-C(37)-H(37)	120.0
C(32)-C(37)-H(37)	120.0
S(2)-C(38)-H(38A)	109.5
S(2)-C(38)-H(38B)	109.5
H(38A)-C(38)-H(38B)	109.5
S(2)-C(38)-H(38C)	109.5
H(38A)-C(38)-H(38C)	109.5
H(38B)-C(38)-H(38C)	109.5

X-ray Crystal Structure Data for 5,7-diisopropoxy-2-(4-isopro poxyphenyl)-3-(trifluoromethoxy)-4H-chromen-4-one (4ss) (CCDC: 1954410)

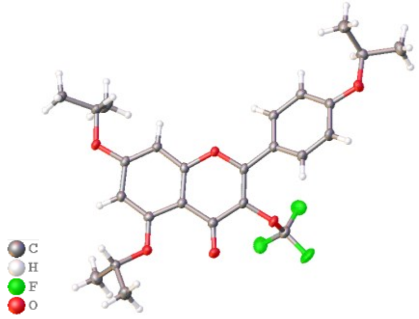

Supplementary Table 17: Crystal data and structure refinement for **4ss**.

Identification code	**4ss**
CCDC	1954410
Empirical formula	$C_{25}H_{27}F_3O_6$
Formula weight	480.46
Temperature/K	114.98(10)
Crystal system	monoclinic
Space group	$P2_1/c$
a/Å	9.6778(2)
b/Å	22.3435(4)
c/Å	11.0846(2)
α/°	90
β/°	97.499(2)
γ/°	90
Volume/Å^3	2376.39(8)
Z	4
ρ_{calc}g/cm^3	1.343
μ/mm^{-1}	0.940
F(000)	1008.0

Crystal size/mm^3	0.2 × 0.1 × 0.1
Radiation	CuKα (λ = 1.54184)
2Θ range for data collection/°	7.914 to 149.492
Index ranges	-11 ≤ h ≤ 12, -27 ≤ k ≤ 26, -13 ≤ l ≤ 13
Reflections collected	43900
Independent reflections	4767 [R_{int} = 0.0309, R_{sigma} = 0.0155]
Data/restraints/parameters	4767/0/313
Goodness-of-fit on F^2	1.103
Final R indexes [I>=2σ (I)]	R_1 = 0.0355, wR_2 = 0.0918
Final R indexes [all data]	R_1 = 0.0400, wR_2 = 0.0968
Largest diff. peak/hole / e Å^{-3}	0.23/-0.24

Supplementary Table 18: Fractional Atomic Coordinates (×10^4) and Equivalent Isotropic Displacement Parameters (Å^2×10^3) for **4ss**. U_{eq} is defined as 1/3 of of the trace of the orthogonalised U_{IJ} tensor.

	**x**	**y**	**z**	**U(eq)**
O4	2286.1(9)	4975.7(4)	5026.9(8)	19.33(19)
F2	4105.9(10)	3814.5(4)	8162.4(9)	41.5(2)
O2	3247.5(10)	4742.4(4)	8269.6(8)	23.1(2)
F1	5472.5(9)	4557.7(5)	8083.7(9)	41.9(2)
O6	-790.1(10)	3972.8(4)	2002.3(8)	23.1(2)
F3	4649.1(11)	4362.6(5)	9745.7(8)	45.4(3)
O5	-809.9(10)	3465.7(4)	6136.3(8)	24.7(2)
O1	6615.7(10)	7001.9(4)	6753.0(9)	27.1(2)
O3	1001.7(11)	4024.4(5)	7804.7(8)	28.5(2)
C11	1298.5(13)	4535.8(5)	4752.4(11)	18.0(2)
C4	3900.9(13)	5555.5(6)	6306.6(11)	19.3(3)
C12	828.4(13)	4488.5(6)	3515.8(11)	19.2(3)
C9	1392.5(13)	4269.7(6)	6923.1(11)	20.0(3)
C15	-304.5(13)	3779.3(6)	5252.0(11)	19.1(3)
C14	-773.9(13)	3720.0(6)	4026.5(11)	19.9(3)
C13	-208.9(13)	4072.9(6)	3164.5(11)	18.7(3)
C10	793.6(13)	4190.5(6)	5648.4(11)	18.0(3)
C7	2914.2(13)	5051.2(6)	6191.5(11)	18.8(3)
C2	5547.8(13)	6196.1(6)	5447.9(12)	20.6(3)
C8	2570.5(13)	4686.9(6)	7073.7(11)	19.4(3)
C1	5740.5(14)	6529.6(6)	6521.5(12)	21.7(3)

C3	4620.7(13)	5717.6(6)	5346.7(11)	19.7(3)
C19	-213.9(14)	4264.6(6)	1003.4(11)	22.4(3)
C6	4987.0(15)	6379.9(6)	7470.6(12)	25.7(3)
C5	4092.7(14)	5899.7(6)	7372.0(12)	23.3(3)
C22	7503.4(14)	7183.4(6)	5863.8(12)	23.6(3)
C16	-2126.8(13)	3151.9(6)	5880.9(12)	22.0(3)
C00S	4343.9(15)	4373.0(7)	8551.2(13)	28.5(3)
C20	-1328.5(16)	4196.4(7)	-78.5(12)	28.0(3)
C18	-1894.3(17)	2513.1(7)	5510.5(15)	32.5(3)
C24	8667.1(16)	7536.1(7)	6580.9(15)	32.4(3)
C17	-2773.6(16)	3190.5(8)	7045.7(14)	33.9(3)
C21	1144.8(16)	3974.1(8)	811.6(13)	35.8(4)
C23	6679.5(18)	7553.5(7)	4875.4(14)	35.3(4)

Supplementary Table 19: Bond lengths [A] and angles [deg] for **4ss**.

O4-C11	1.3769(15)
O4-C7	1.3636(15)
F2-C00S	1.3304(18)
O2-C8	1.4055(14)
O2-C00S	1.3483(18)
F1-C00S	1.3340(18)
O6-C13	1.3553(15)
O6-C19	1.4570(15)
F3-C00S	1.3186(16)
O5-C15	1.3476(15)
O5-C16	1.4498(15)
O1-C1	1.3564(16)
O1-C22	1.4474(16)
O3-C9	1.2227(16)
C11-C12	1.3908(17)
C11-C10	1.3954(18)
C4-C7	1.4717(18)
C4-C3	1.3935(18)
C4-C5	1.4013(18)
C12-C13	1.3855(18)
C9-C10	1.4662(17)
C9-C8	1.4652(18)
C15-C14	1.3813(17)
C15-C10	1.4300(18)
C14-C13	1.4036(18)

C7-C8	1.3469(18)
C2-C1	1.3958(18)
C2-C3	1.3908(18)
C1-C6	1.3966(19)
C19-C20	1.5124(18)
C19-C21	1.506(2)
C6-C5	1.3738(19)
C22-C24	1.5125(19)
C22-C23	1.514(2)
C16-C18	1.510(2)
C16-C17	1.509(2)
C7-O4-C11	120.55(10)
C00S-O2-C8	114.85(10)
C13-O6-C19	119.73(10)
C15-O5-C16	120.18(10)
C1-O1-C22	120.04(10)
O4-C11-C12	113.60(11)
O4-C11-C10	122.33(11)
C12-C11-C10	124.02(12)
C3-C4-C7	121.08(11)
C3-C4-C5	118.45(12)
C5-C4-C7	120.42(12)
C13-C12-C11	117.37(12)
O3-C9-C10	126.27(12)
O3-C9-C8	120.75(11)
C8-C9-C10	112.98(11)
O5-C15-C14	124.16(12)
O5-C15-C10	115.86(11)
C14-C15-C10	119.98(12)
C15-C14-C13	120.55(12)
O6-C13-C12	124.99(11)
O6-C13-C14	113.84(11)
C12-C13-C14	121.16(11)
C11-C10-C9	119.21(11)
C11-C10-C15	116.86(11)
C15-C10-C9	123.93(11)
O4-C7-C4	112.38(11)
C8-C7-O4	119.39(11)
C8-C7-C4	128.22(11)
C3-C2-C1	119.48(12)
O2-C8-C9	115.06(11)
C7-C8-O2	120.17(11)
C7-C8-C9	124.33(11)
O1-C1-C2	125.96(12)

O1-C1-C6	114.73(11)
C2-C1-C6	119.31(12)
C2-C3-C4	121.32(12)
O6-C19-C20	104.68(11)
O6-C19-C21	110.01(11)
C21-C19-C20	112.97(12)
C5-C6-C1	120.84(12)
C6-C5-C4	120.56(12)
O1-C22-C24	104.81(11)
O1-C22-C23	110.03(12)
C24-C22-C23	112.43(12)
O5-C16-C18	110.60(11)
O5-C16-C17	104.92(11)
C17-C16-C18	112.32(12)
F2-C00S-O2	113.66(11)
F2-C00S-F1	106.28(13)
F1-C00S-O2	112.34(12)
F3-C00S-F2	108.38(12)
F3-C00S-O2	108.03(12)
F3-C00S-F1	107.94(12)

Spectrum Data

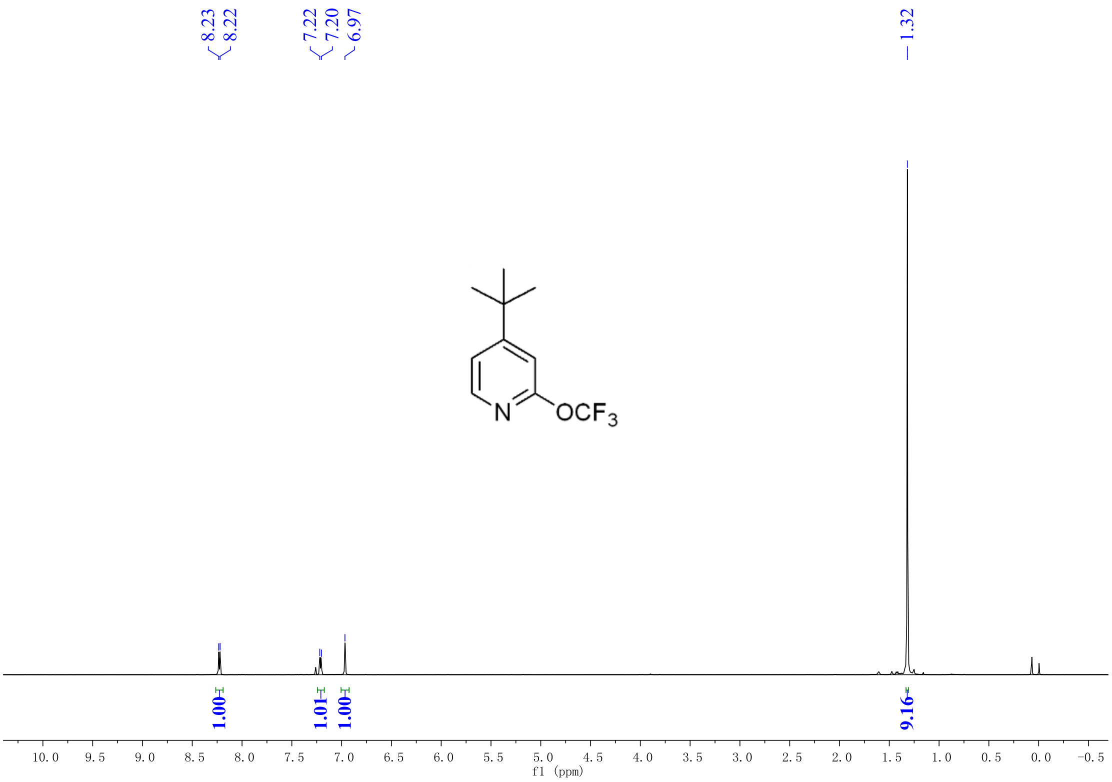

Supplementary Figure 3. ^{1}H NMR spectrum (400 MHz, $CDCl_3$) of **3a**

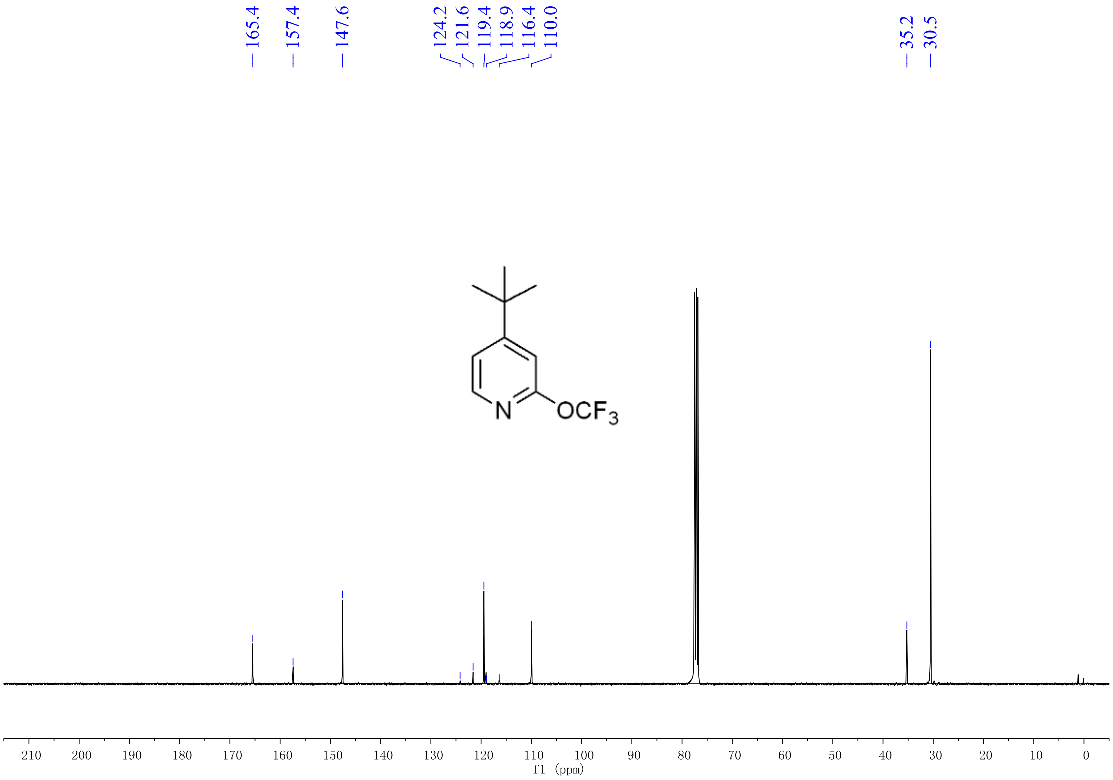

Supplementary Figure 4. ^{13}C NMR spectrum (101 MHz, $CDCl_3$) of **3a**

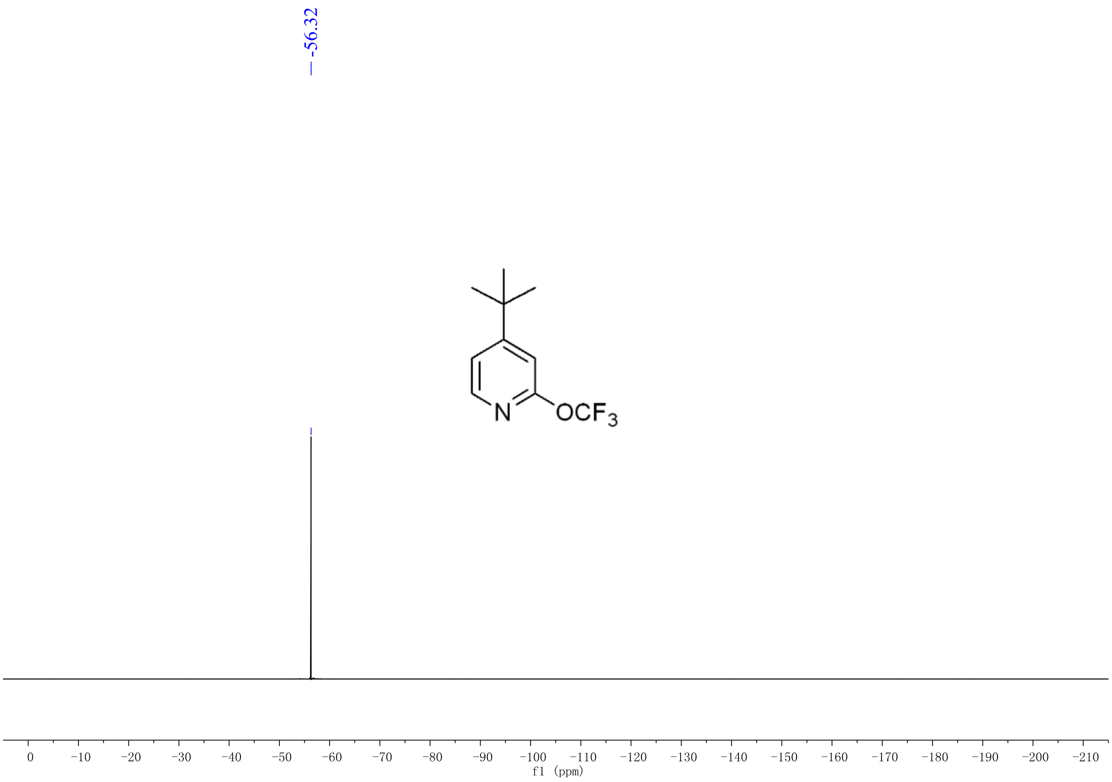

Supplementary Figure 5. ^{19}F NMR spectrum (376 MHz, $CDCl_3$) of **3a**

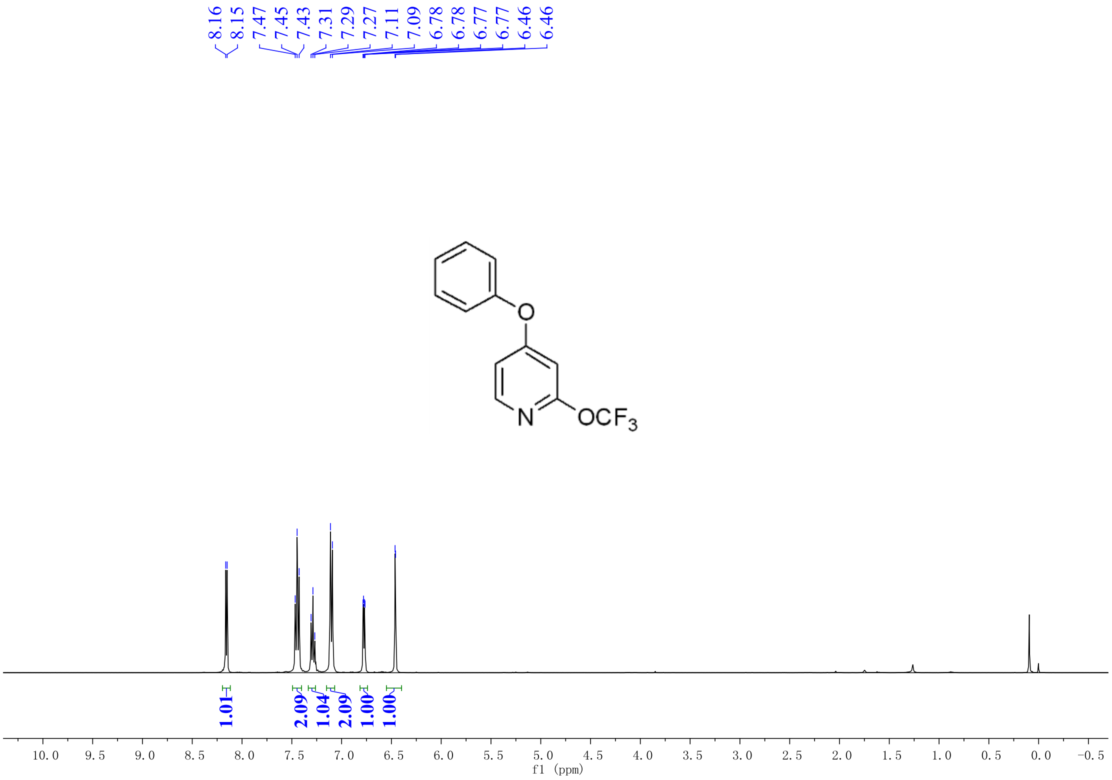

Supplementary Figure 6. ^{1}H NMR spectrum (400 MHz, $CDCl_3$) of **3b**

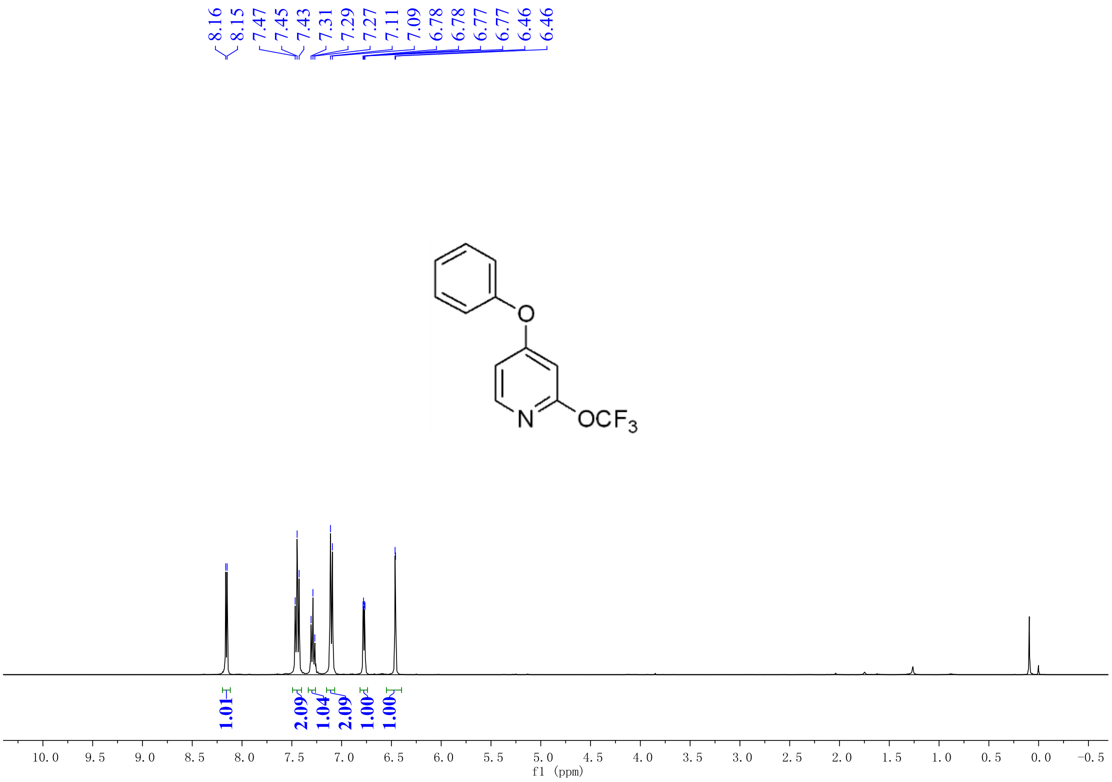

Supplementary Figure 7. ^{13}C NMR spectrum (101 MHz, $CDCl_3$) of **3b**

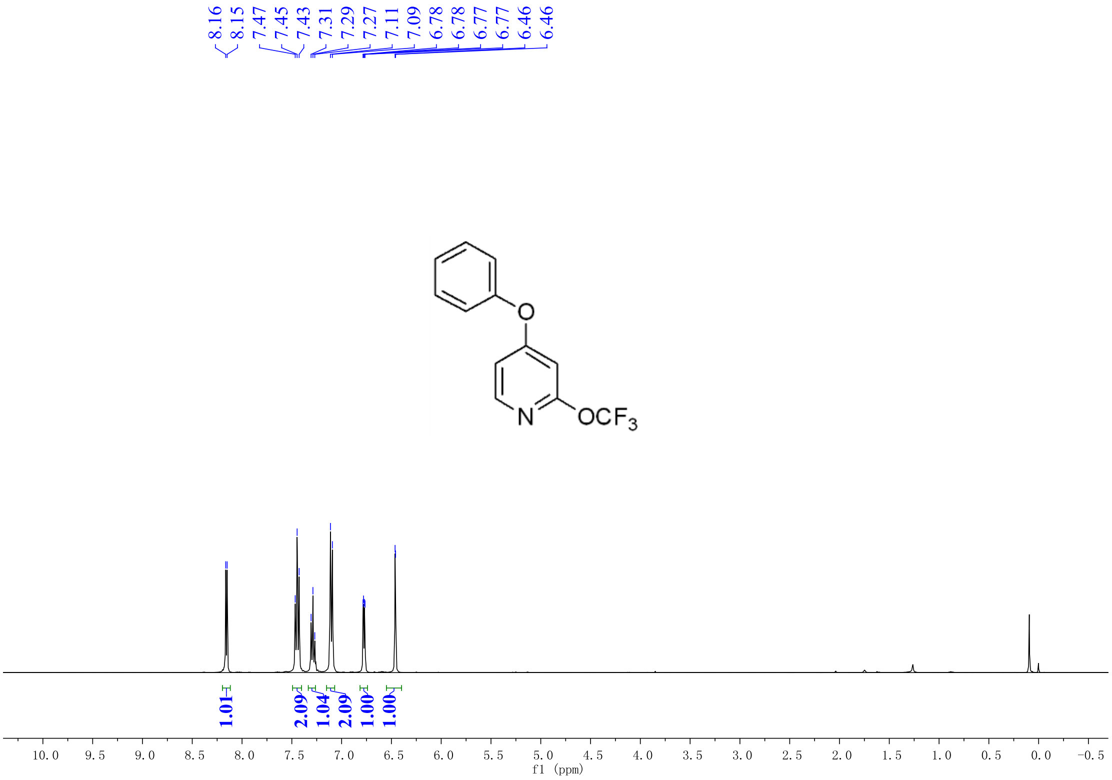

Supplementary Figure 8. ^{19}F NMR spectrum (376 MHz, $CDCl_3$) of **3b**

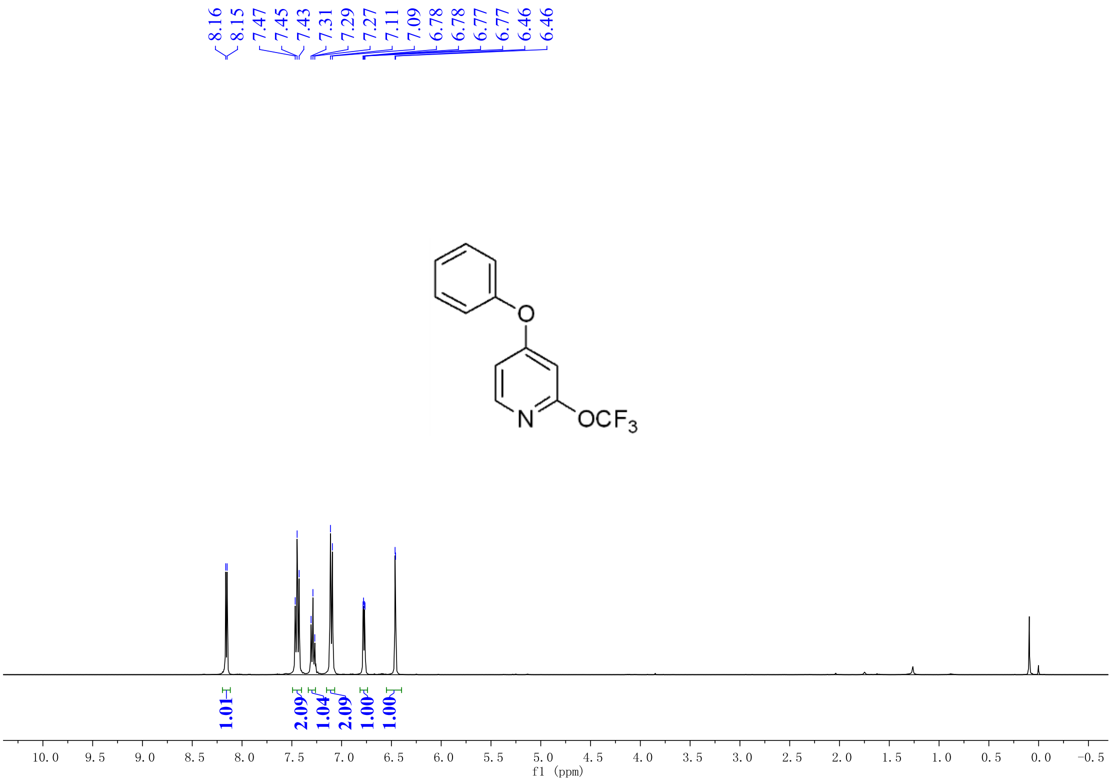

Supplementary Figure 9. ^{1}H NMR spectrum (400 MHz, $CDCl_3$) of **3c**

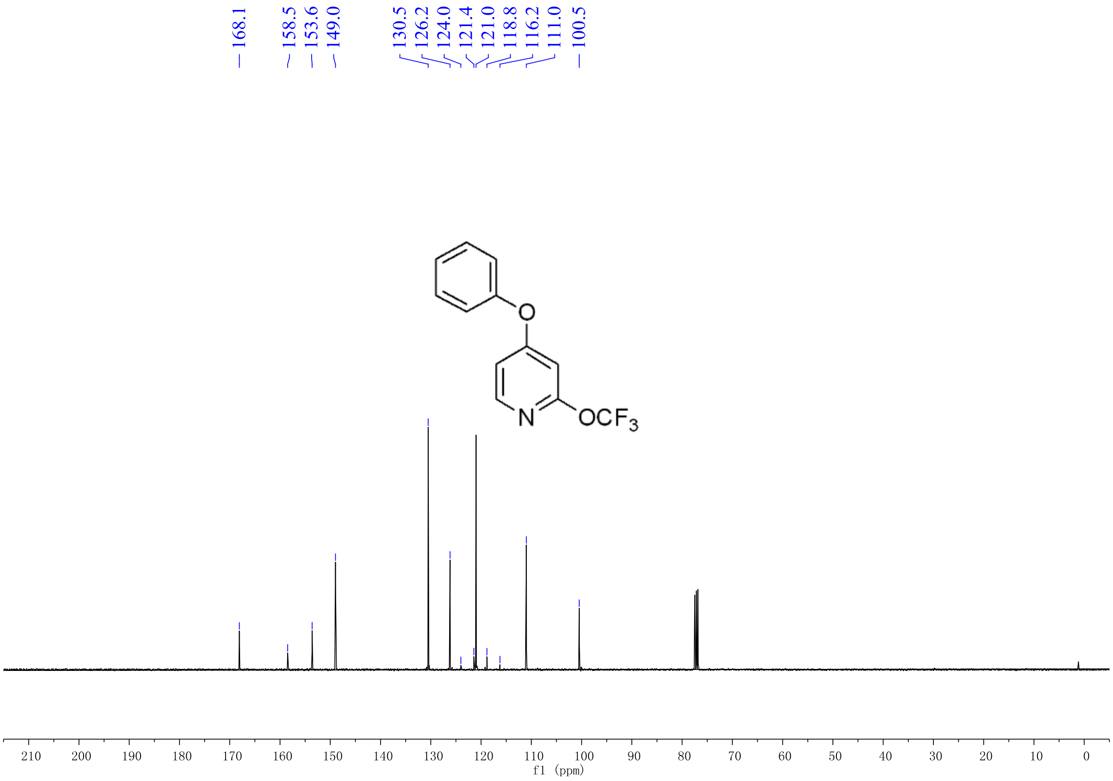

Supplementary Figure 10. ^{13}C NMR spectrum (101 MHz, $CDCl_3$) of **3c**

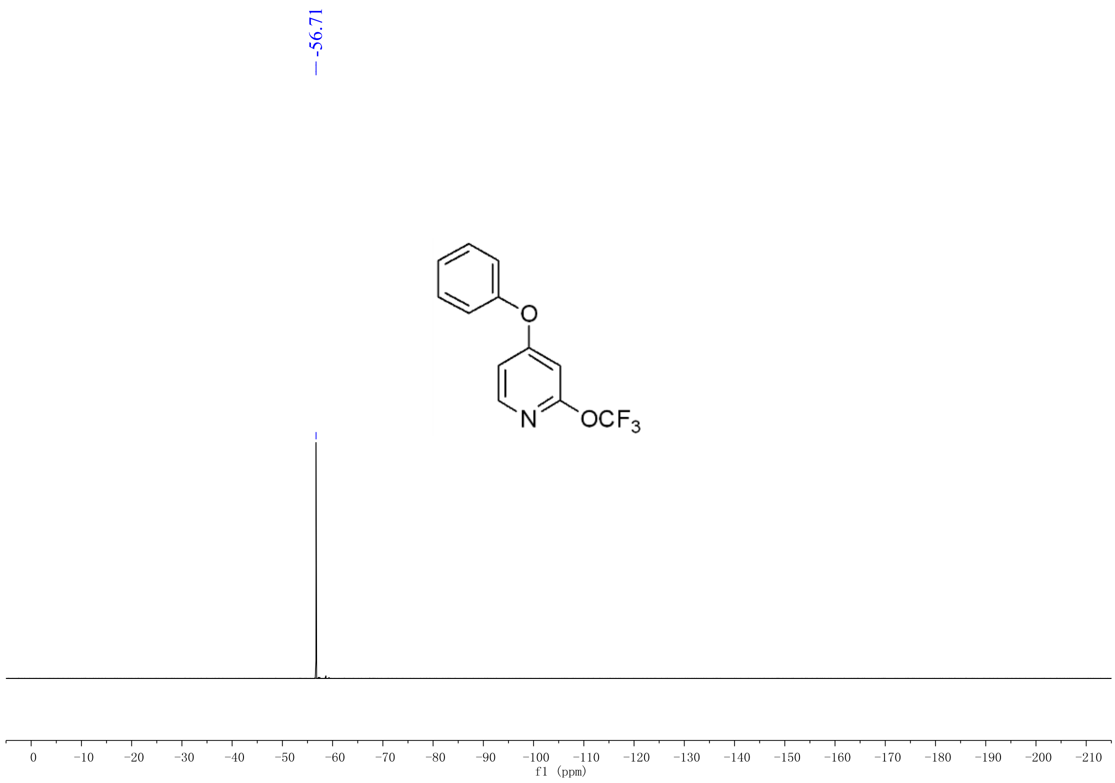

Supplementary Figure 11. ^{19}F NMR spectrum (376 MHz, $CDCl_3$) of **3c**

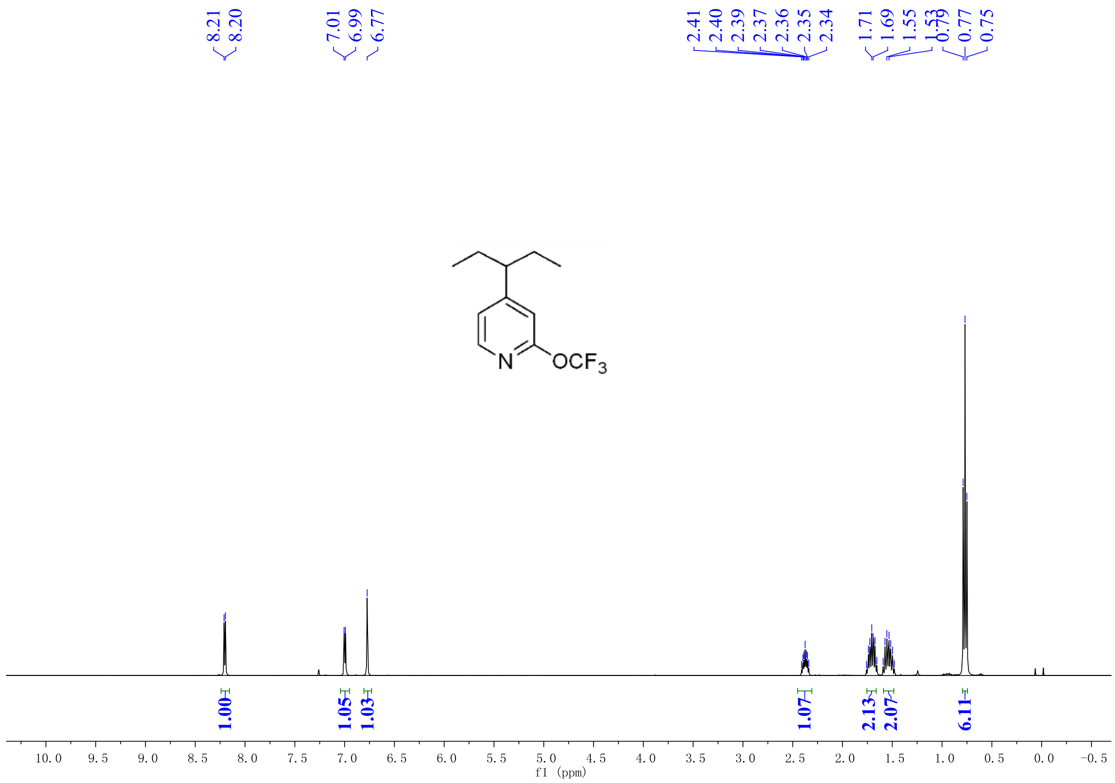

Supplementary Figure 12. ^{1}H NMR spectrum (400 MHz, $CDCl_3$) of **3d**

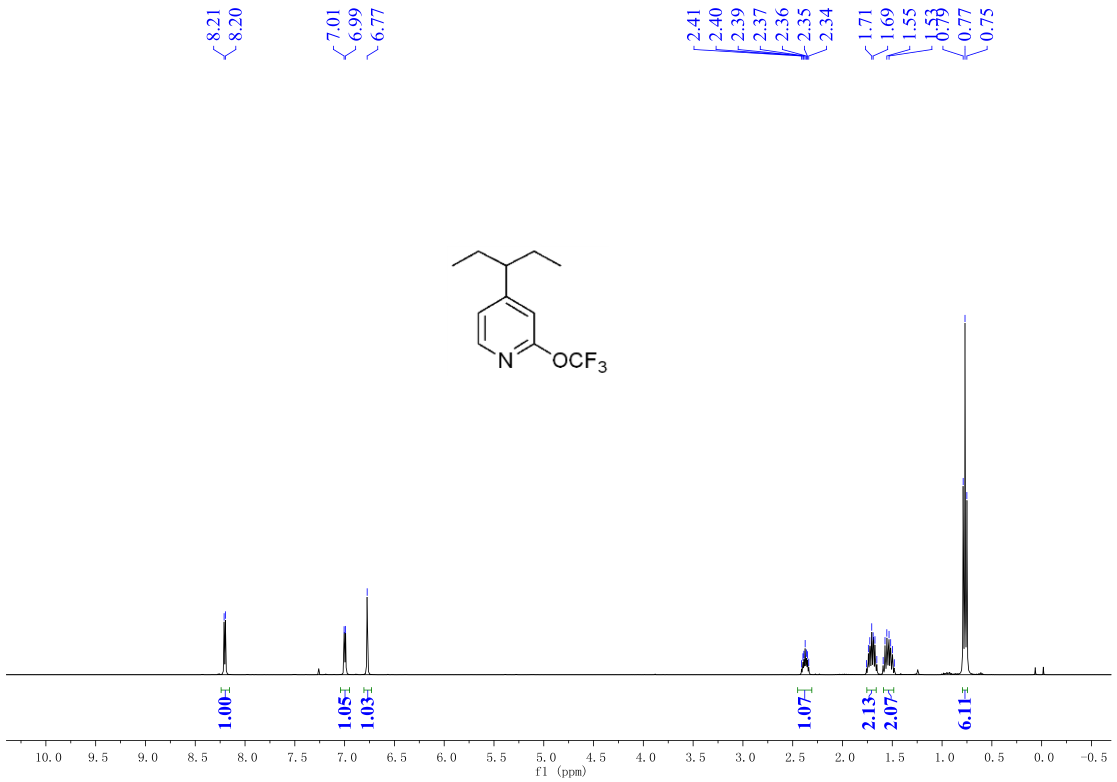

Supplementary Figure 13. ^{13}C NMR spectrum (101 MHz, $CDCl_3$) of **3d**

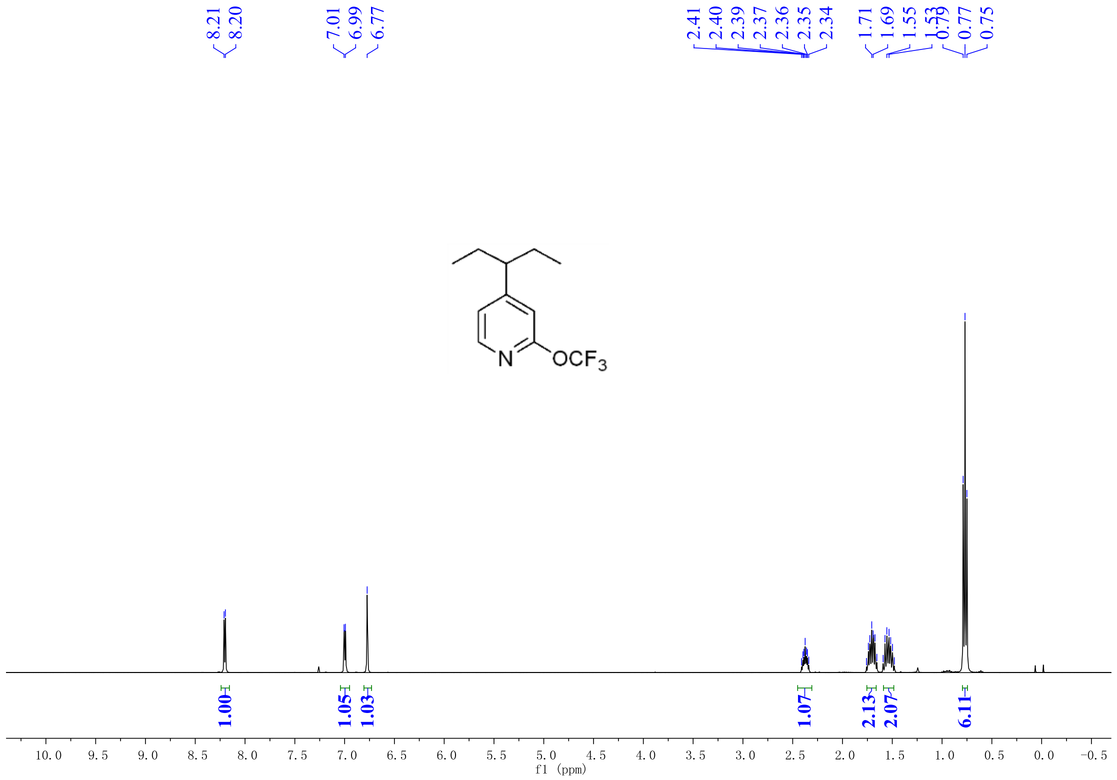

Supplementary Figure 14. ^{19}F NMR spectrum (376 MHz, $CDCl_3$) of **3d**

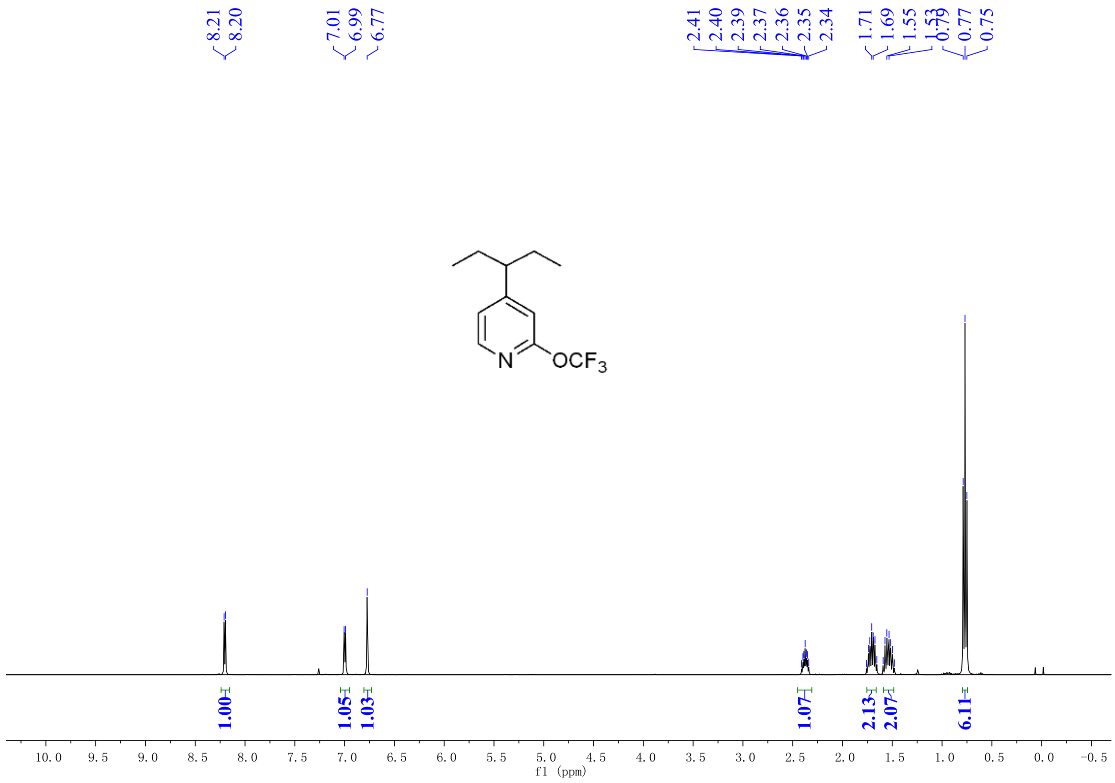

Supplementary Figure 15. ^{1}H NMR spectrum (400 MHz, $CDCl_3$) of **3e**

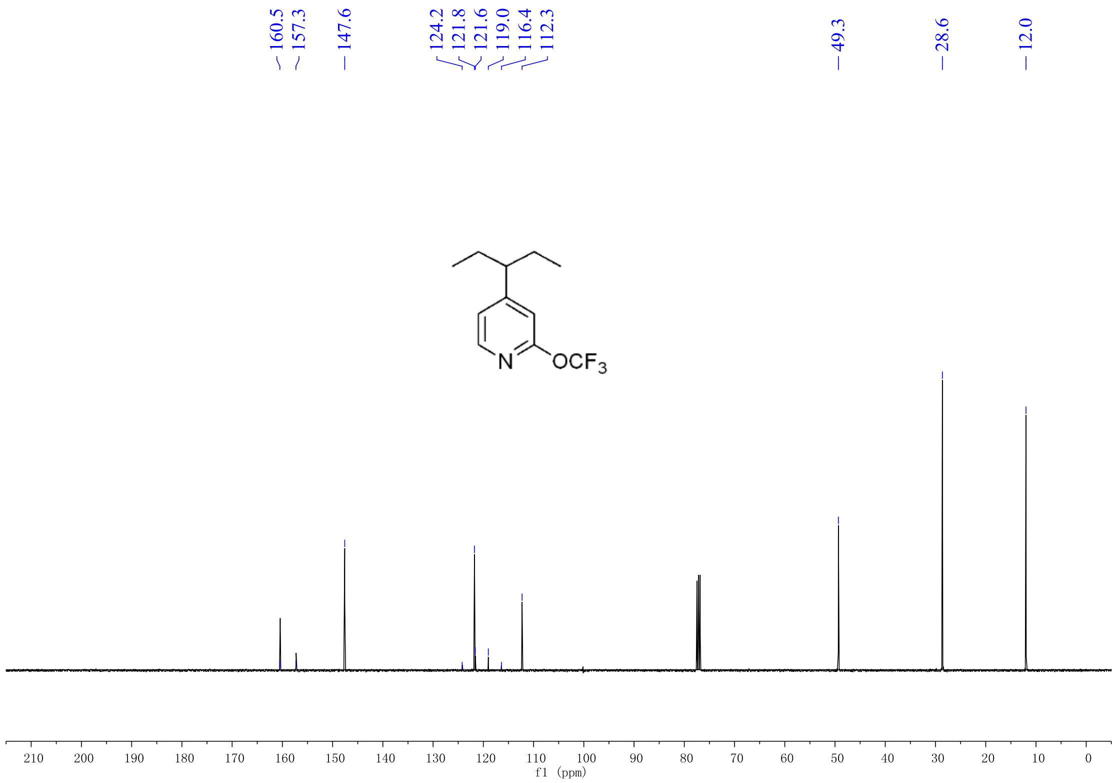

Supplementary Figure 16. ^{13}C NMR spectrum (101 MHz, $CDCl_3$) of **3e**

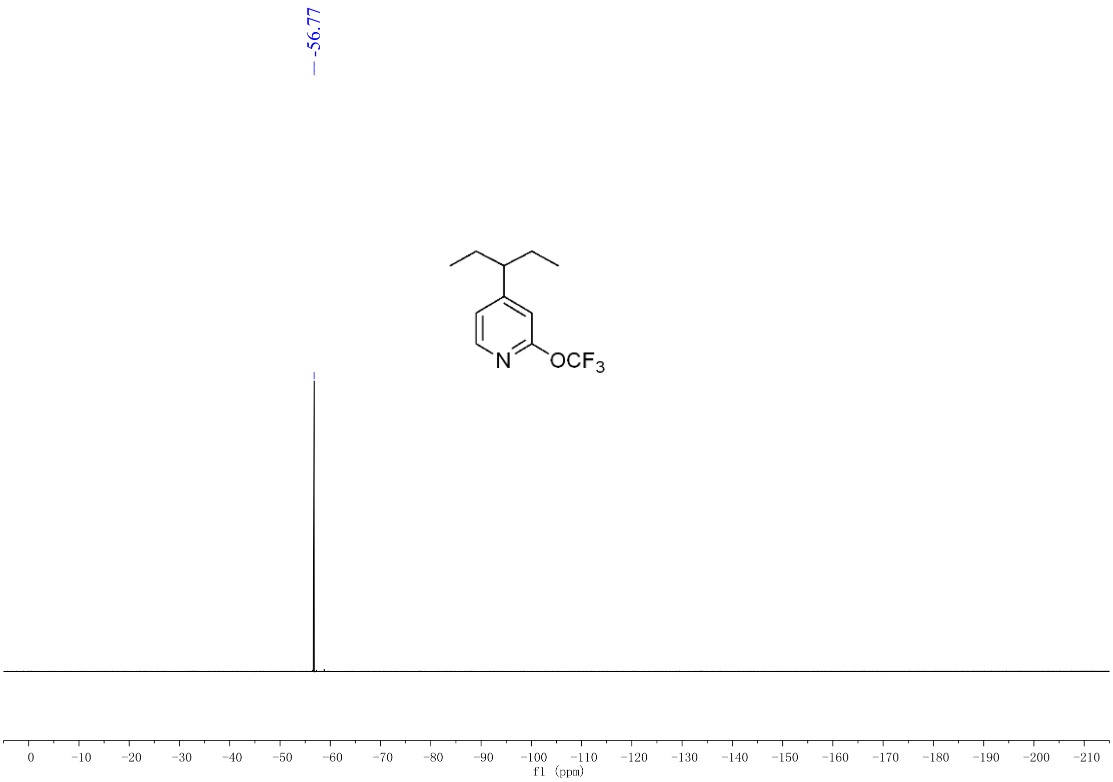

Supplementary Figure 17. ^{19}F NMR spectrum (376 MHz, $CDCl_3$) of **3e**

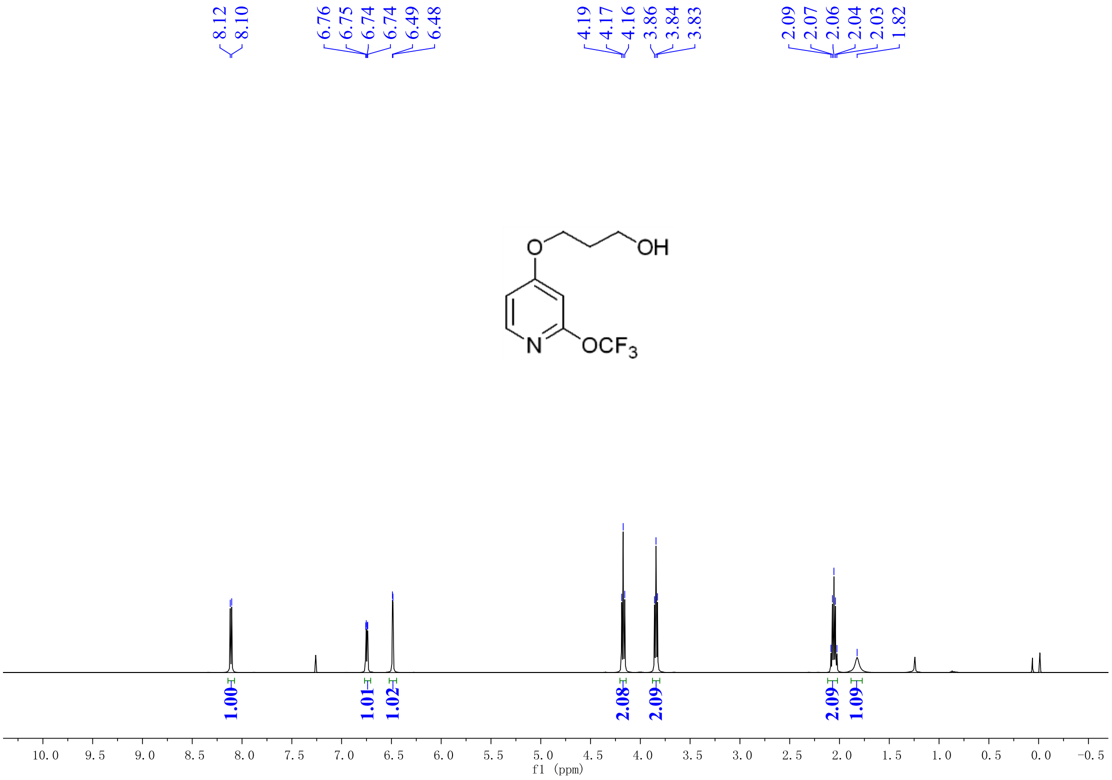

Supplementary Figure 18. ^{1}H NMR spectrum (400 MHz, $CDCl_3$) of **3f**

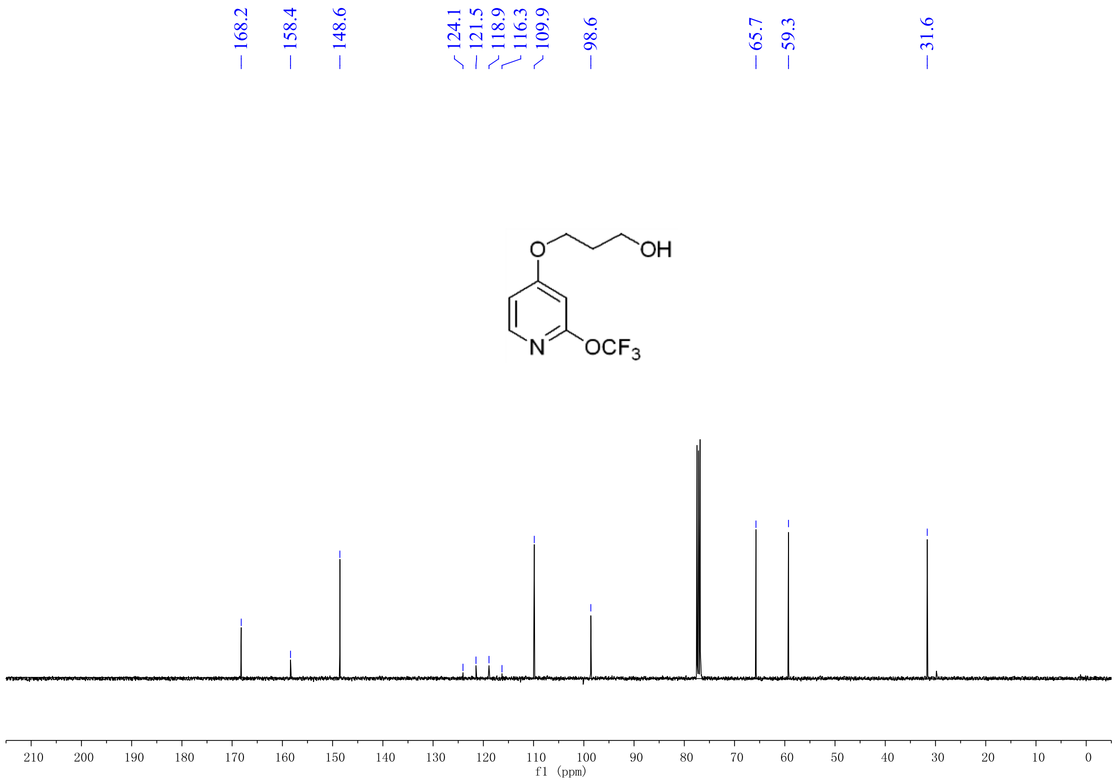

Supplementary Figure 19. ^{13}C NMR spectrum (101 MHz, $CDCl_3$) of **3f**

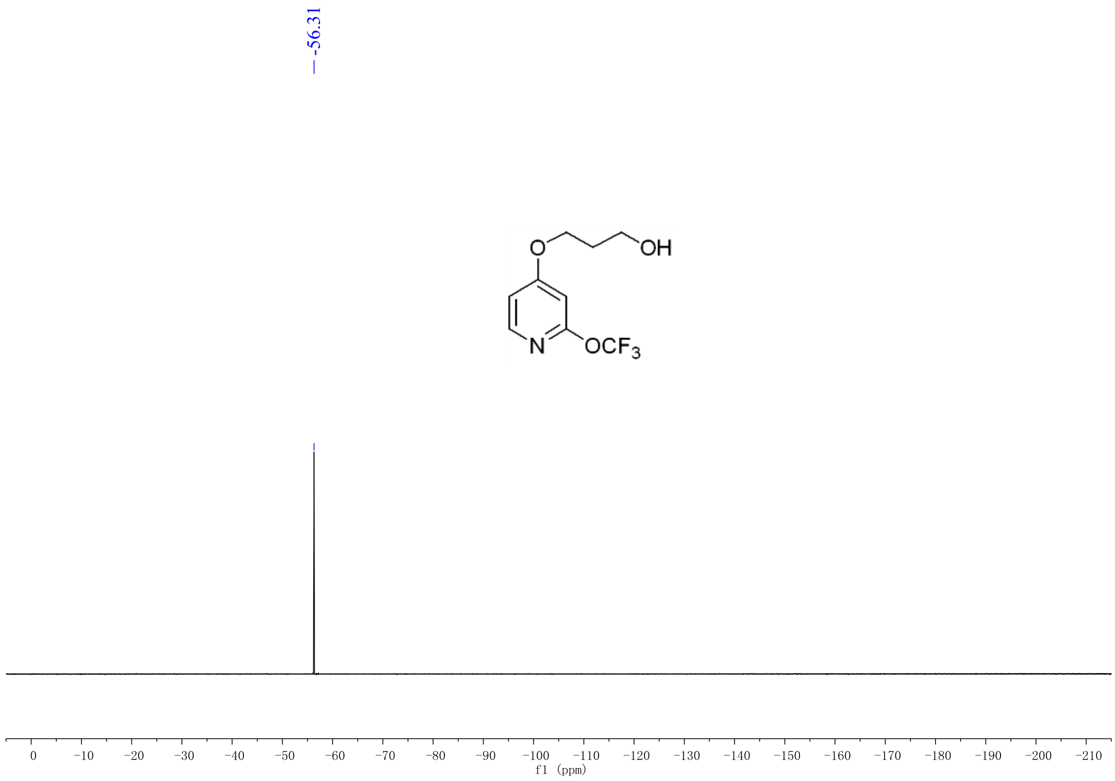

Supplementary Figure 20. ^{19}F NMR spectrum (376 MHz, $CDCl_3$) of **3f**

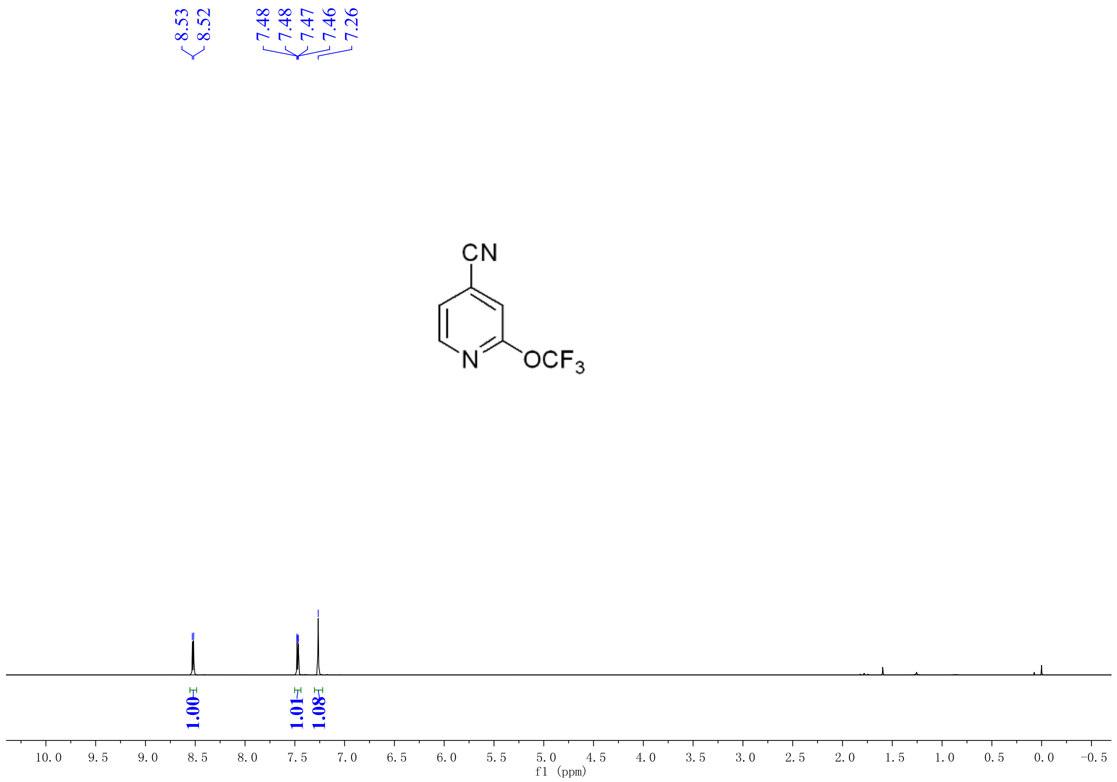

Supplementary Figure 21. ^{1}H NMR spectrum (400 MHz, $CDCl_3$) of **3g**

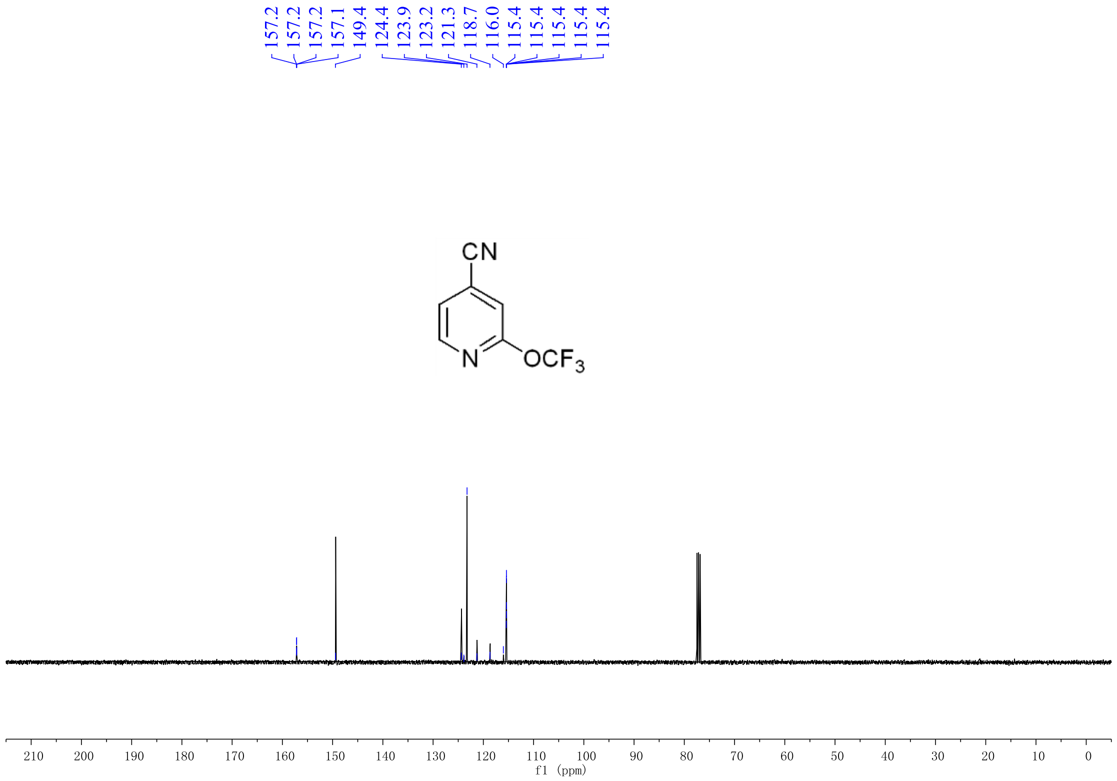

Supplementary Figure 22. ^{13}C NMR spectrum (101 MHz, $CDCl_3$) of **3g**

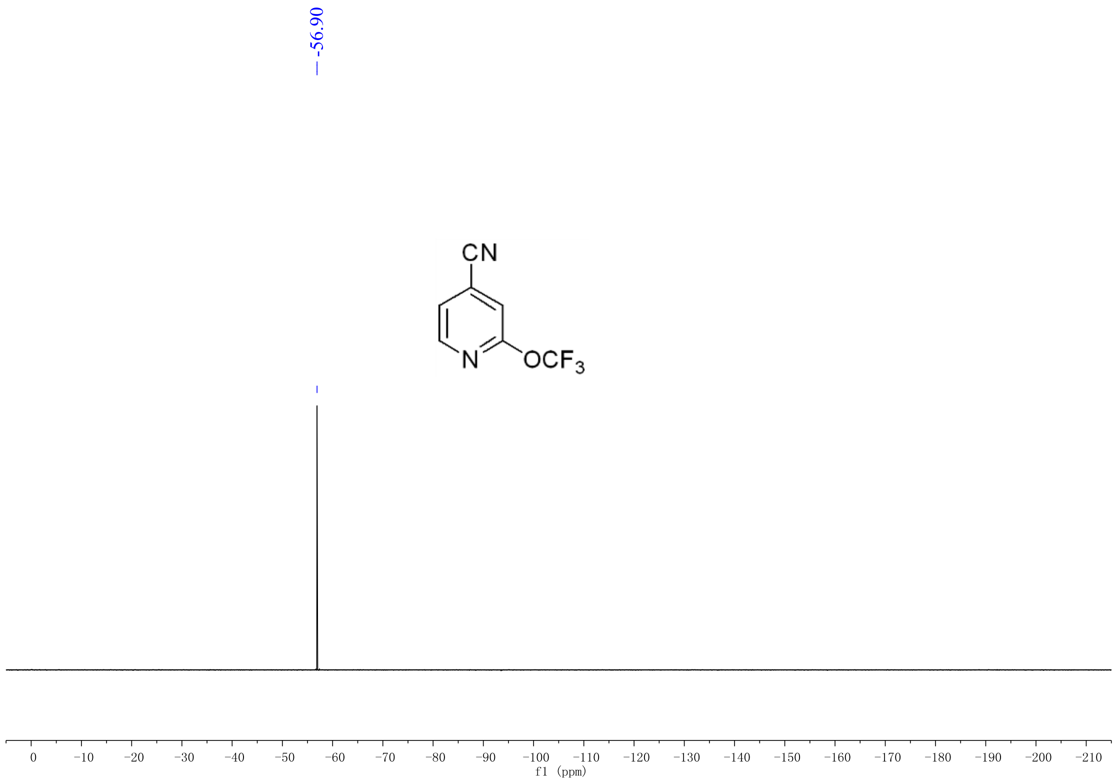

Supplementary Figure 23. ^{19}F NMR spectrum (376 MHz, $CDCl_3$) of **3g**

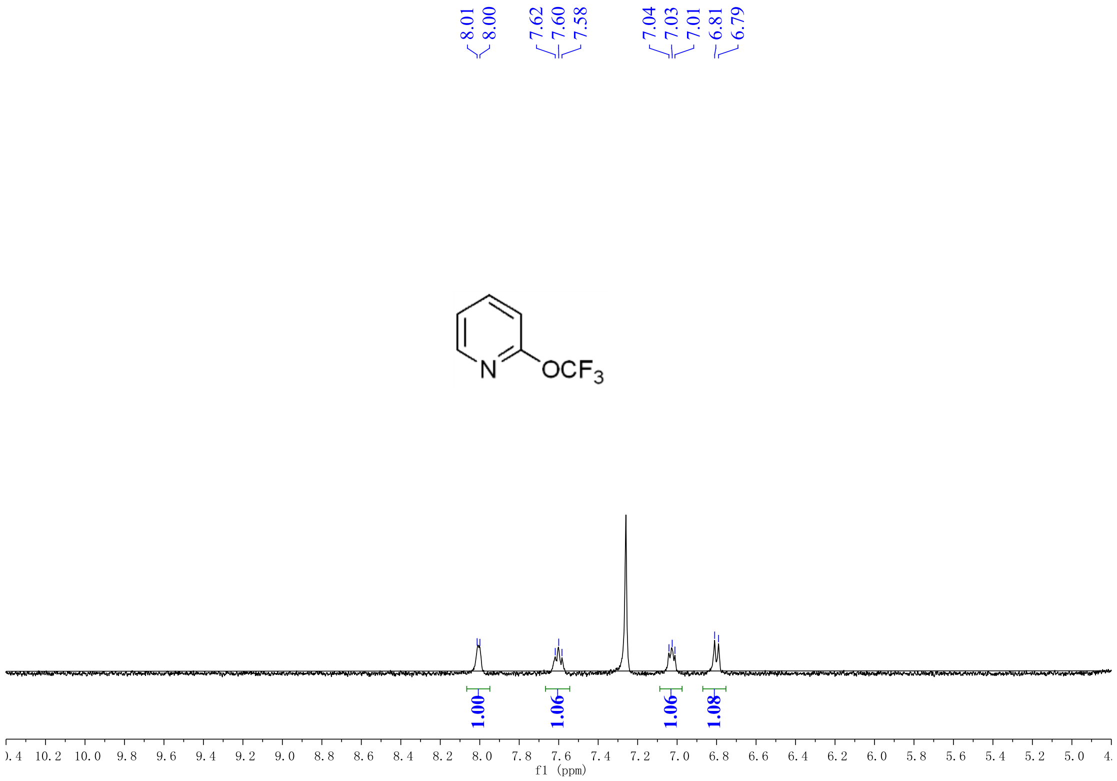

Supplementary Figure 24. ^{1}H NMR spectrum (400 MHz, $CDCl_3$) of **3h**

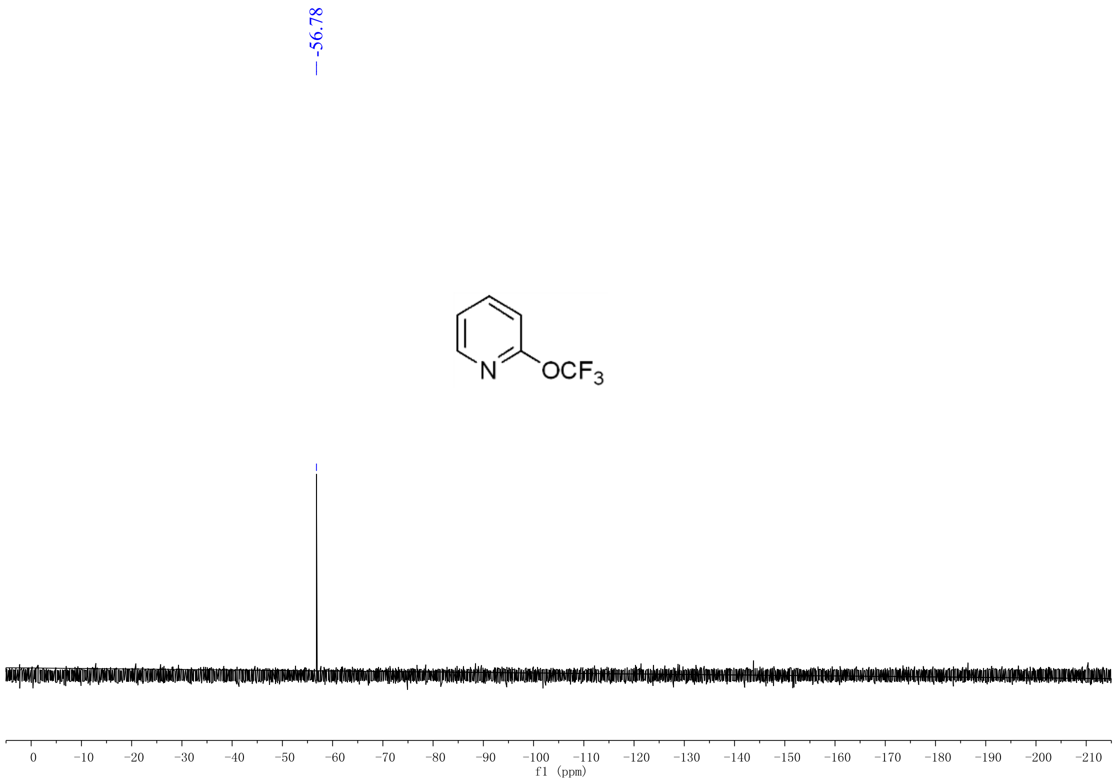

Supplementary Figure 25. ^{19}F NMR spectrum (376 MHz, $CDCl_3$) of **3h**

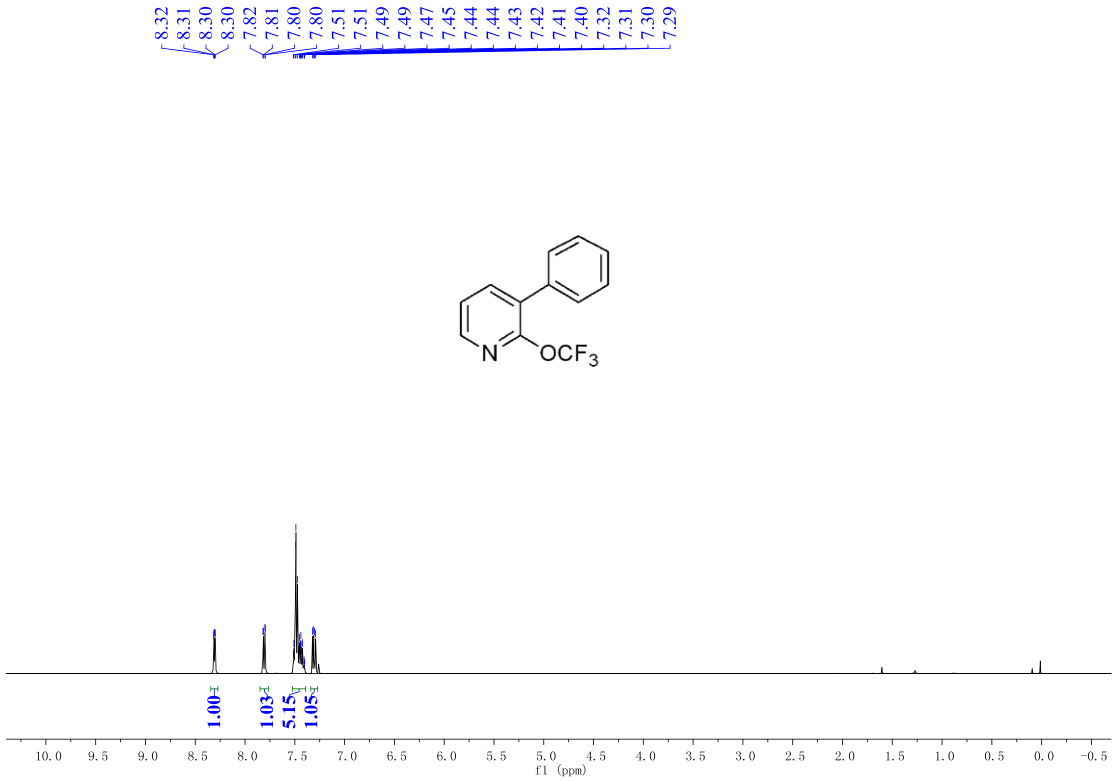

Supplementary Figure 26. ^{1}H NMR spectrum (400 MHz, $CDCl_3$) of **3i**

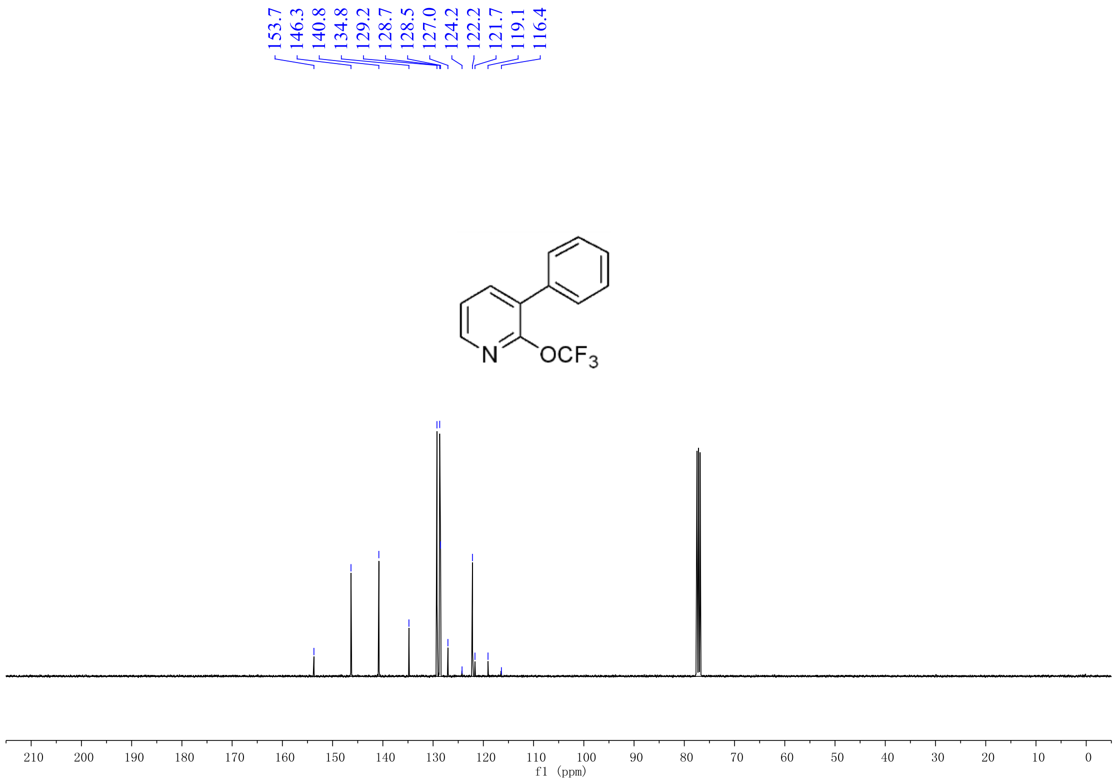

Supplementary Figure 27. ^{13}C NMR spectrum (101 MHz, $CDCl_3$) of **3i**

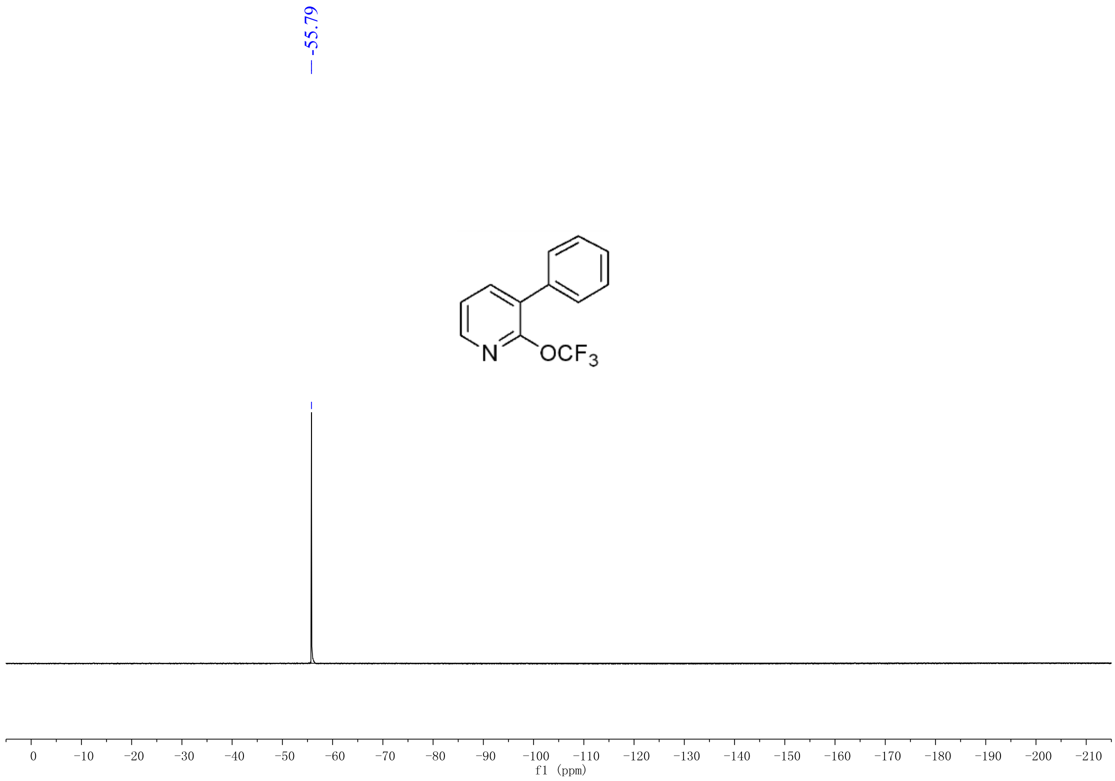

Supplementary Figure 28. ^{19}F NMR spectrum (376 MHz, $CDCl_3$) of **3i**

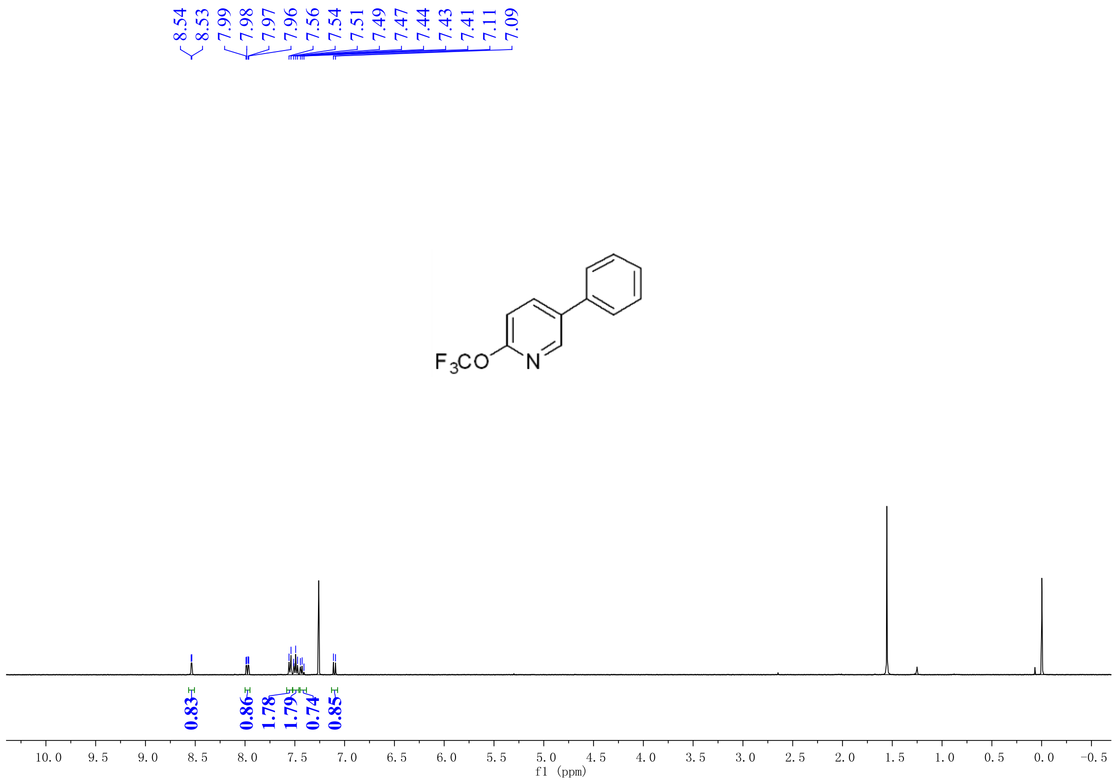

Supplementary Figure 29. ^{1}H NMR spectrum (400 MHz, $CDCl_3$) of ***iso*-3i**

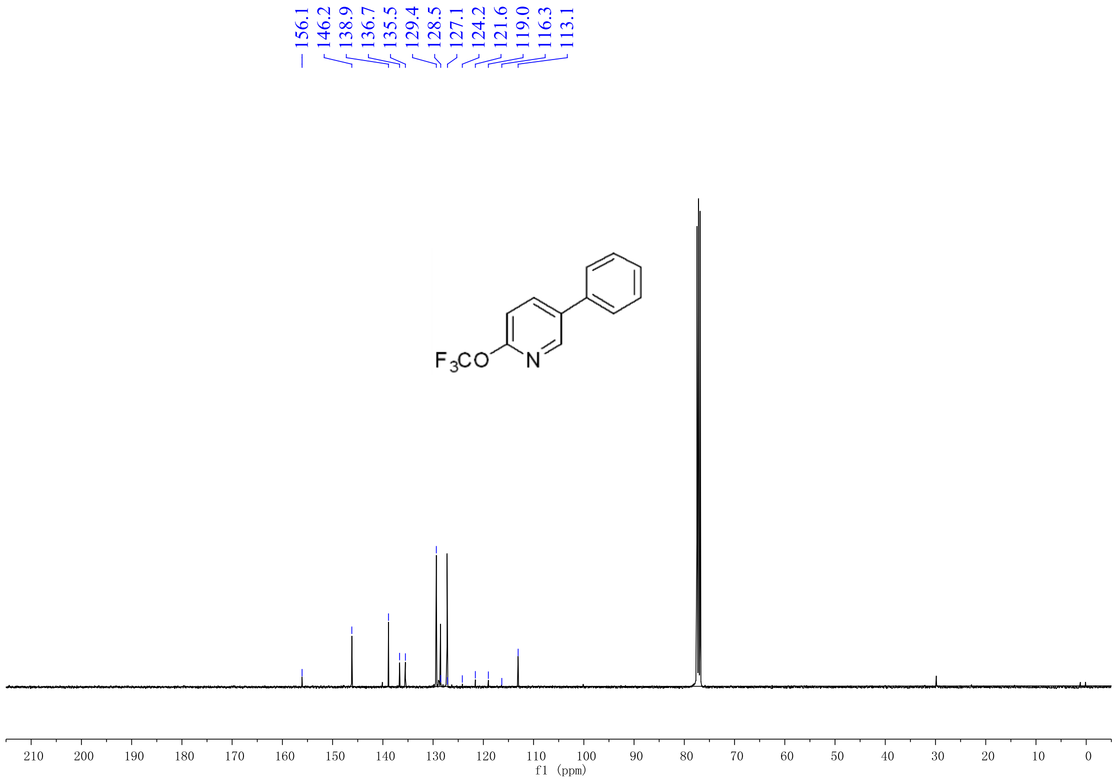

Supplementary Figure 30. ^{13}C NMR spectrum (101 MHz, $CDCl_3$) of ***iso*-3i**

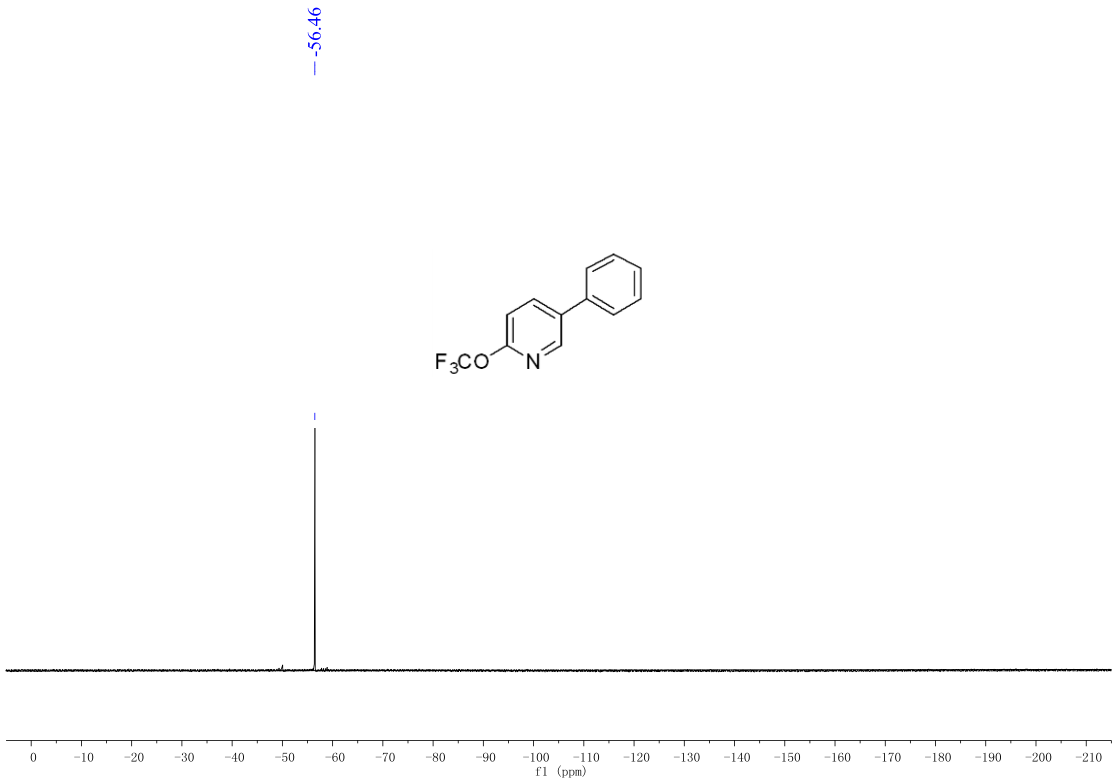

Supplementary Figure 31. ^{19}F NMR spectrum (376 MHz, $CDCl_3$) of ***iso*-3i**

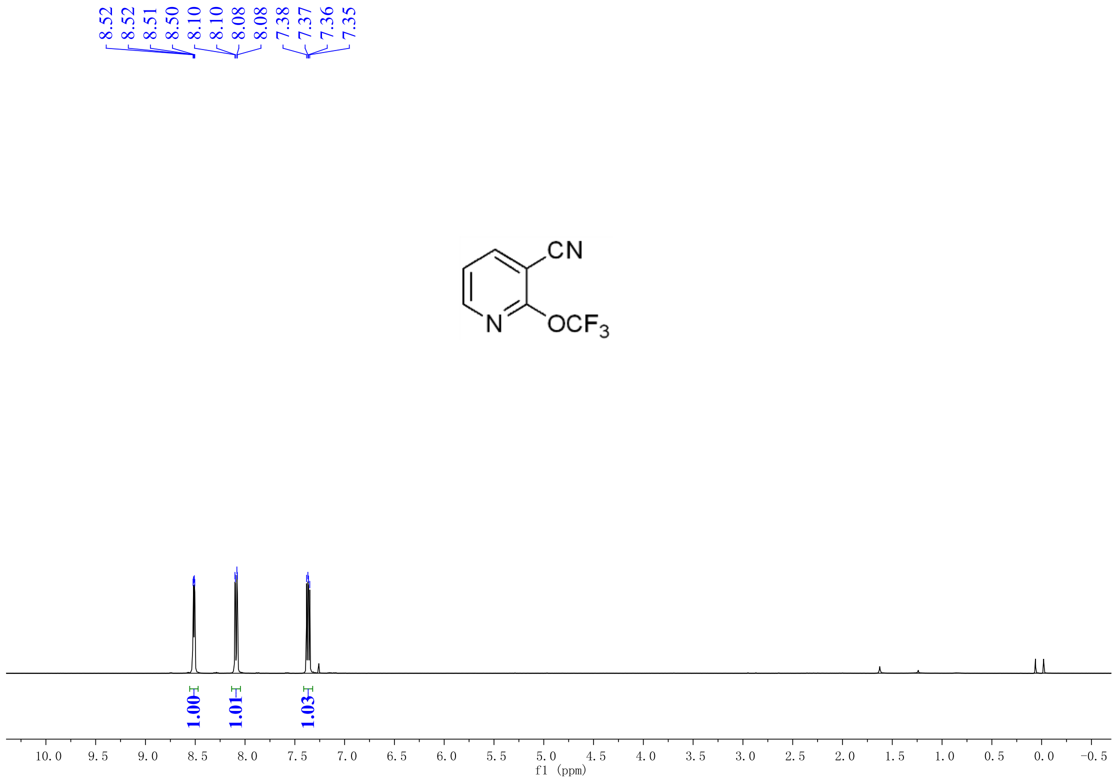

Supplementary Figure 32. ^{1}H NMR spectrum (400 MHz, $CDCl_3$) of **3j**

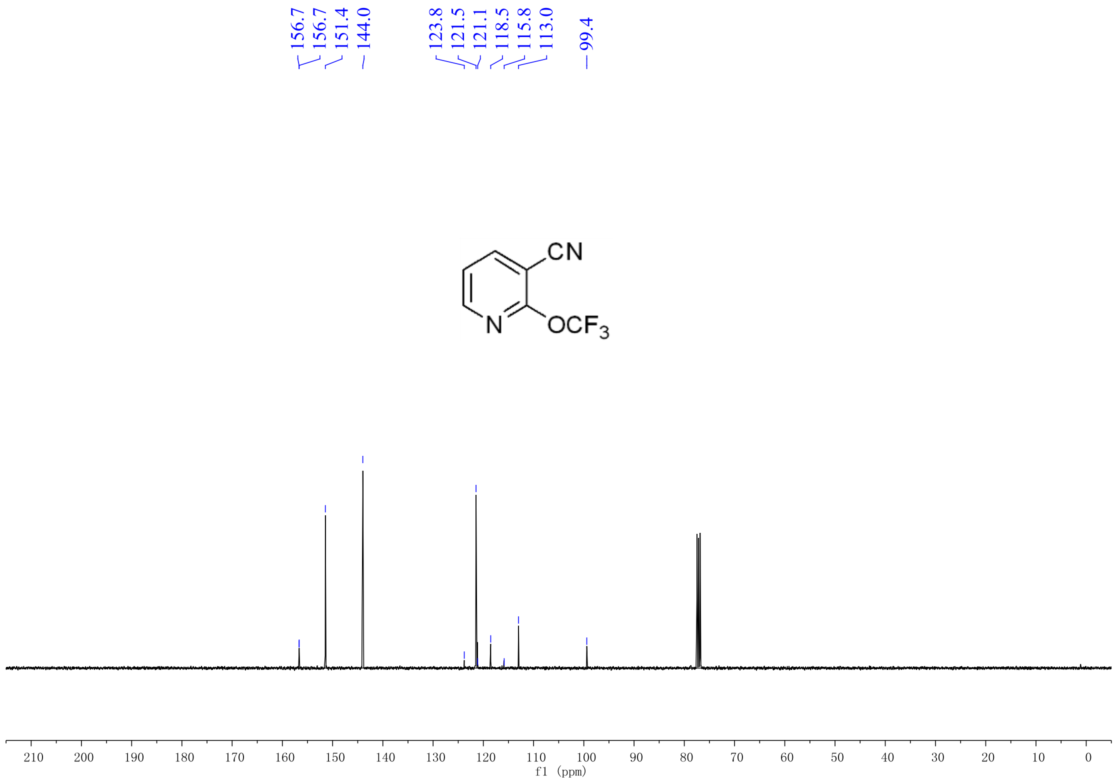

Supplementary Figure 33. ^{13}C NMR spectrum (101 MHz, $CDCl_3$) of **3j**

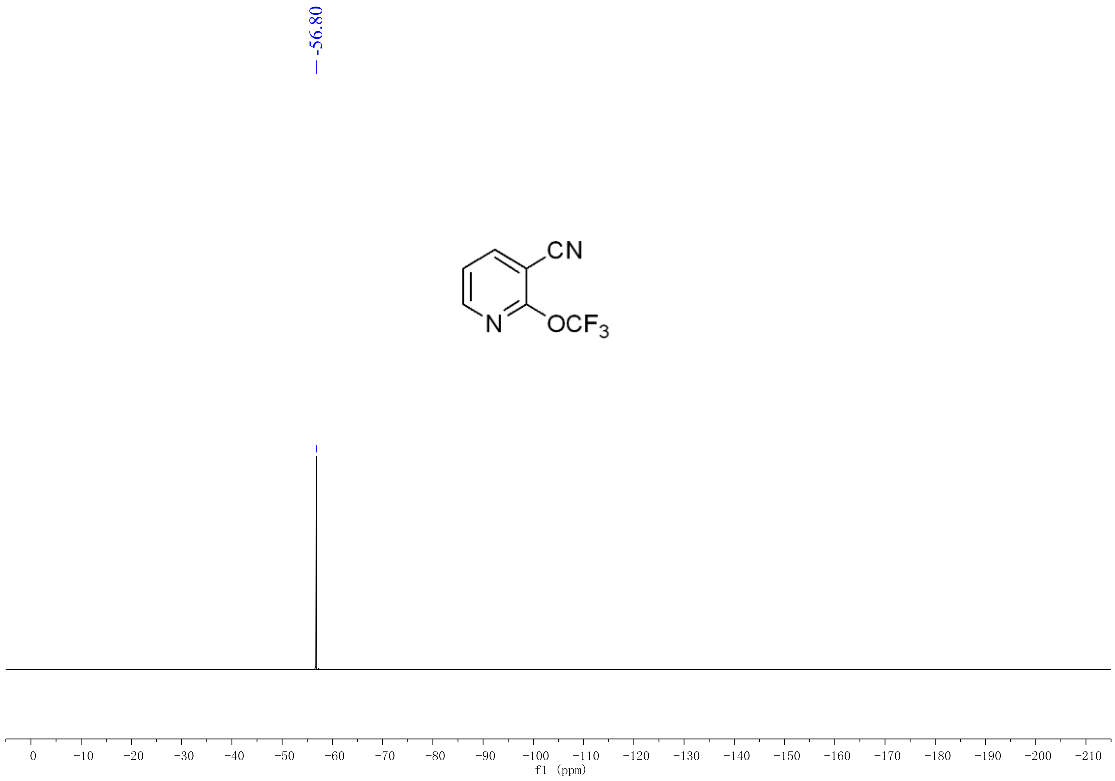

Supplementary Figure 34. ^{19}F NMR spectrum (376 MHz, $CDCl_3$) of **3j**

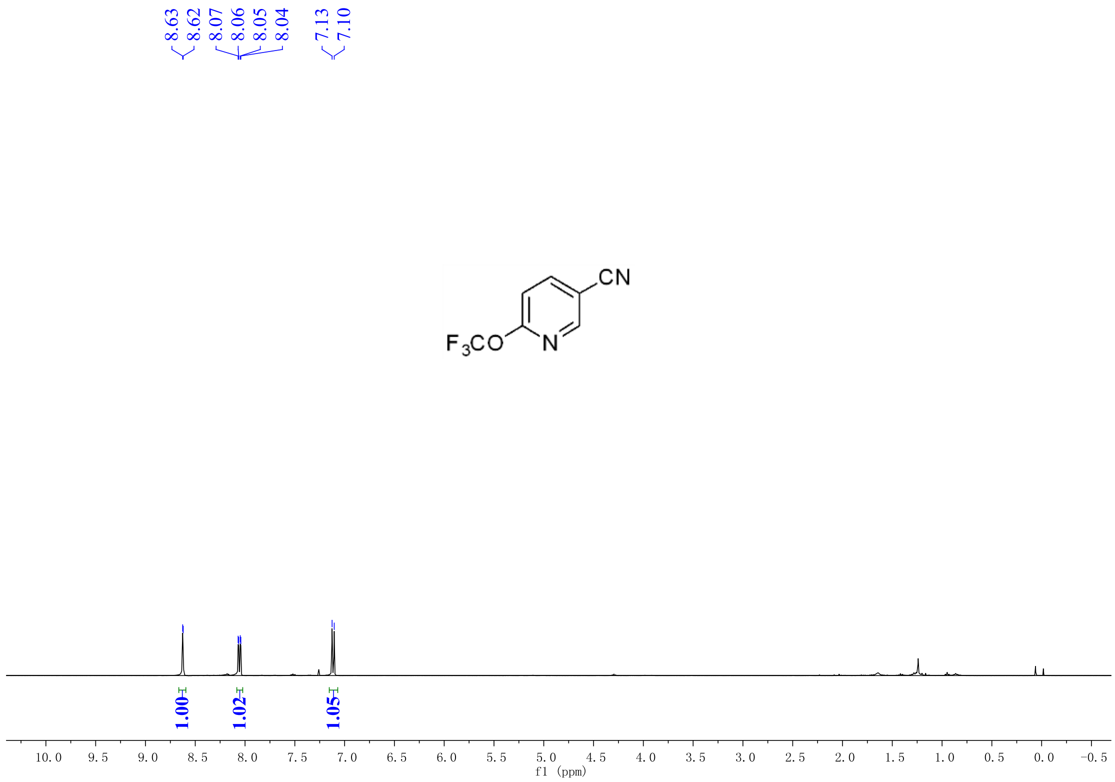

Supplementary Figure 35. ^{1}H NMR spectrum (400 MHz, $CDCl_3$) of ***iso*-3j**

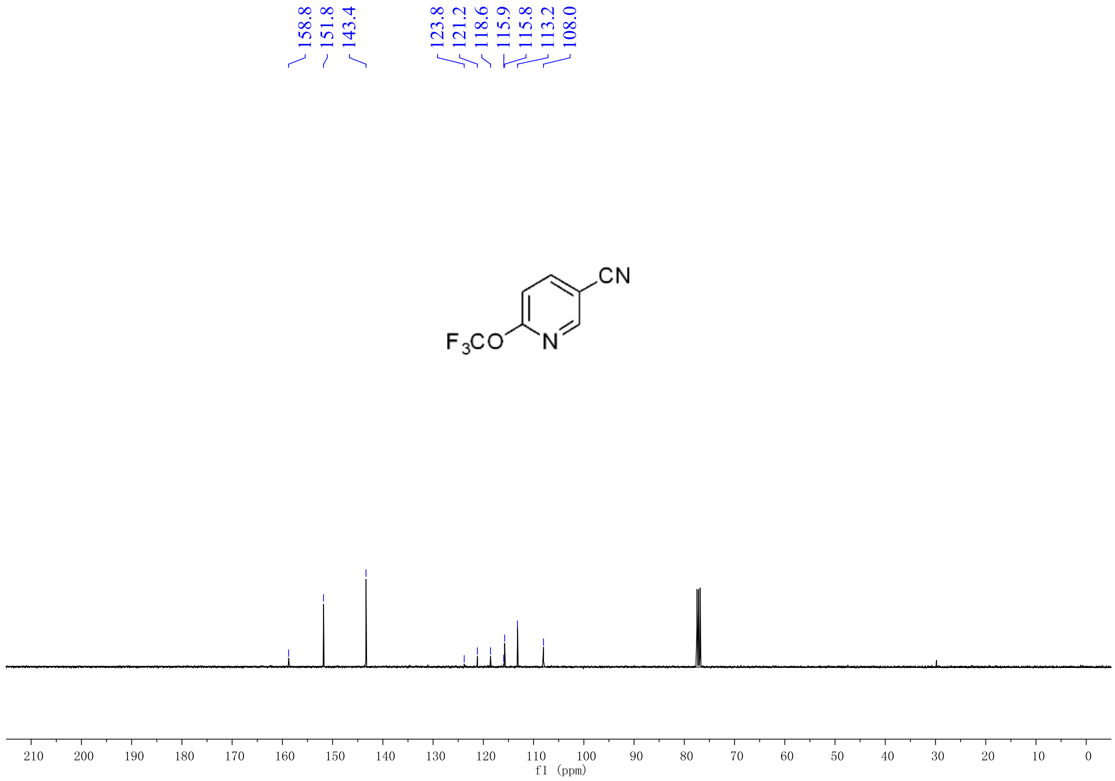

Supplementary Figure 36. ^{13}C NMR spectrum (101 MHz, $CDCl_3$) of ***iso*-3j**

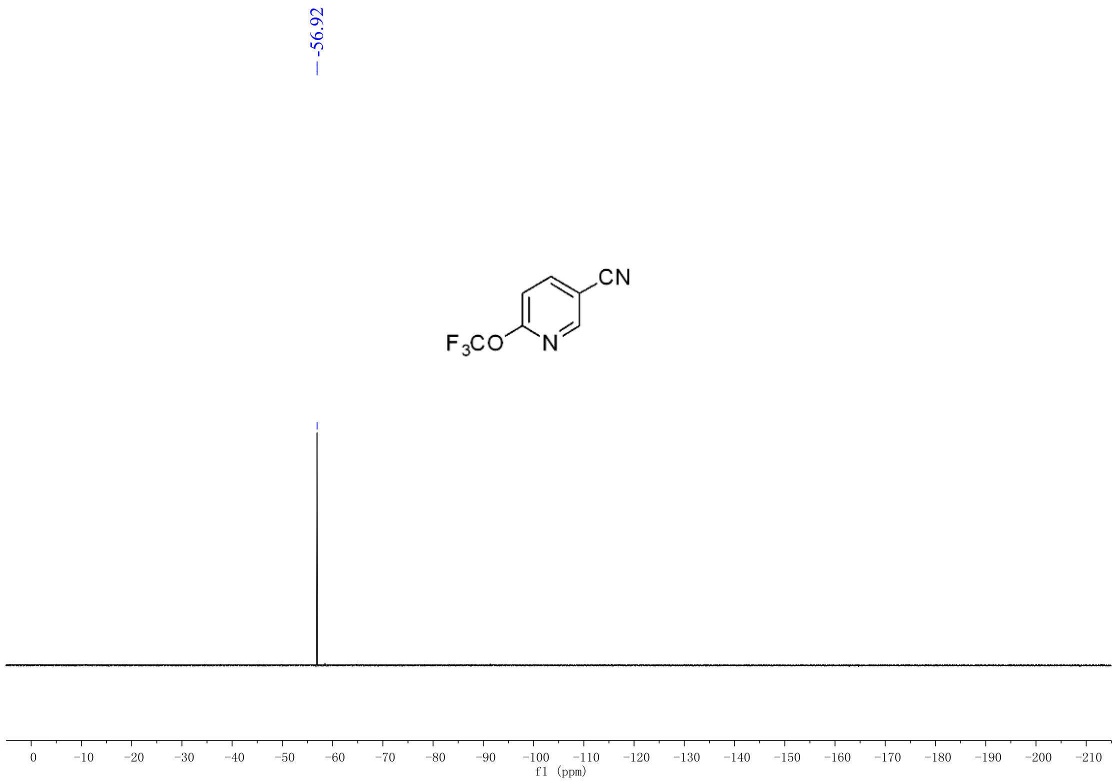

Supplementary Figure 37. ^{19}F NMR spectrum (376 MHz, $CDCl_3$) of ***iso*-3j**

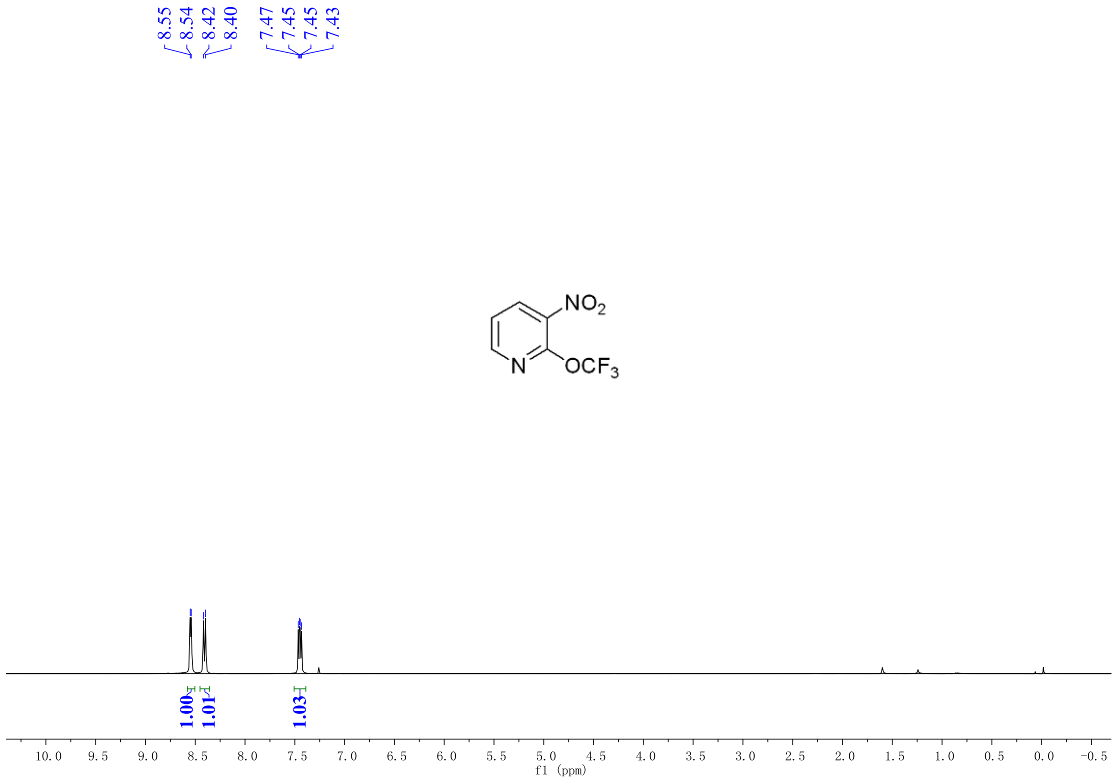

Supplementary Figure 38. ^{1}H NMR spectrum (400 MHz, $CDCl_3$) of **3k**

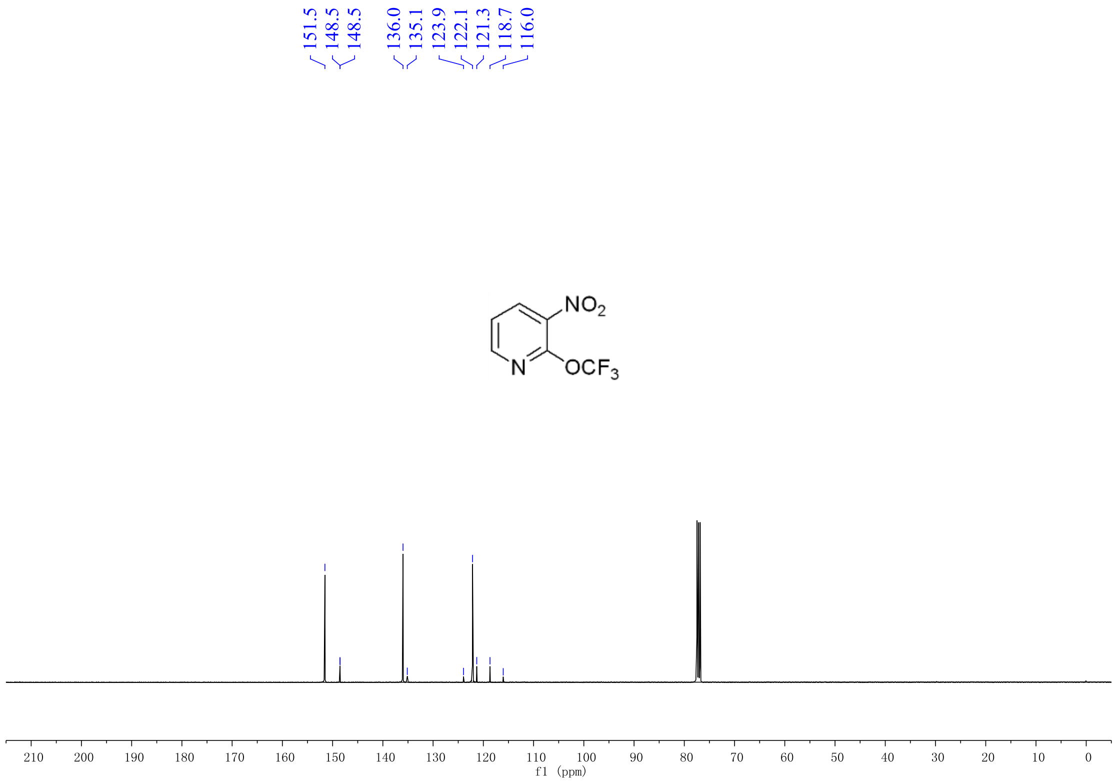

Supplementary Figure 39. ^{13}C NMR spectrum (101 MHz, $CDCl_3$) of **3k**

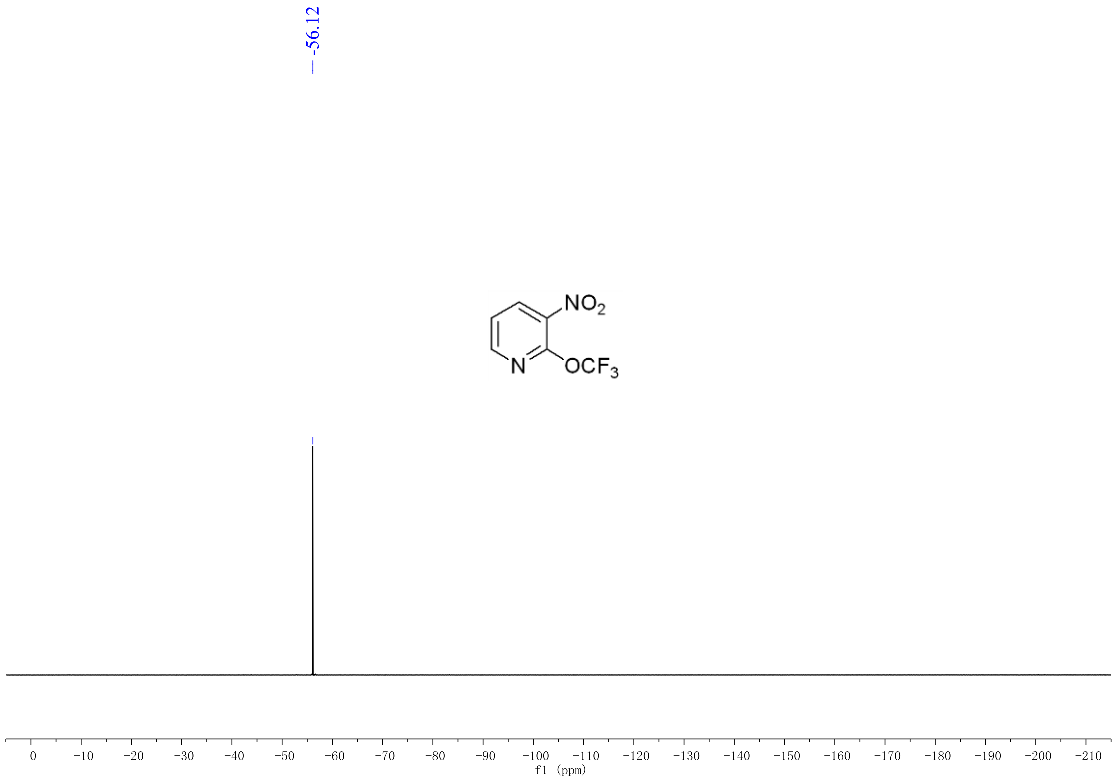

Supplementary Figure 40. ^{19}F NMR spectrum (376 MHz, $CDCl_3$) of **3k**

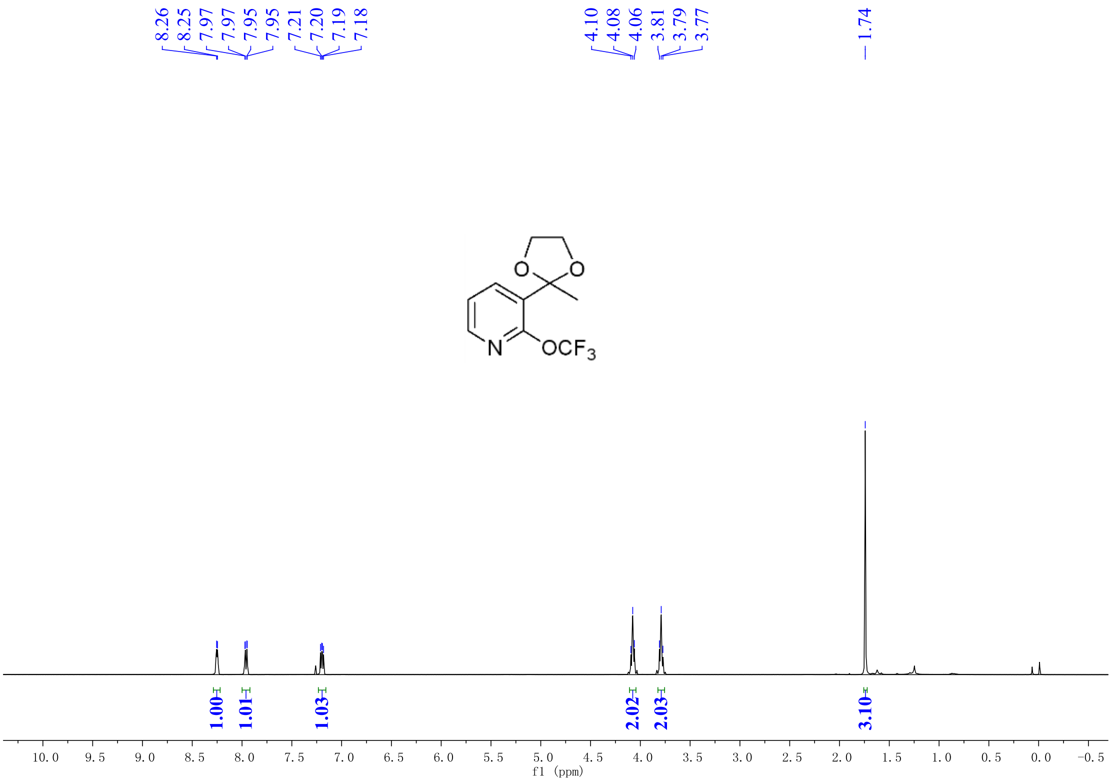

Supplementary Figure 41. ^{1}H NMR spectrum (400 MHz, $CDCl_3$) of **3l**

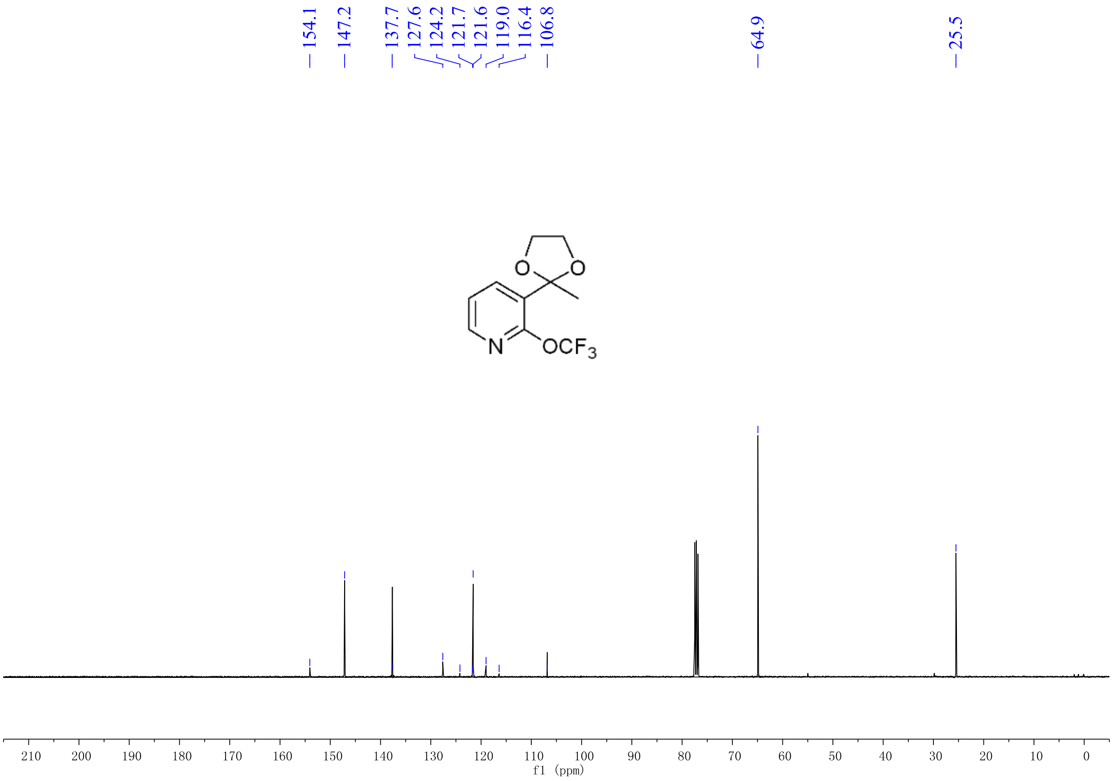

Supplementary Figure 42. ^{13}C NMR spectrum (101 MHz, $CDCl_3$) of **3l**

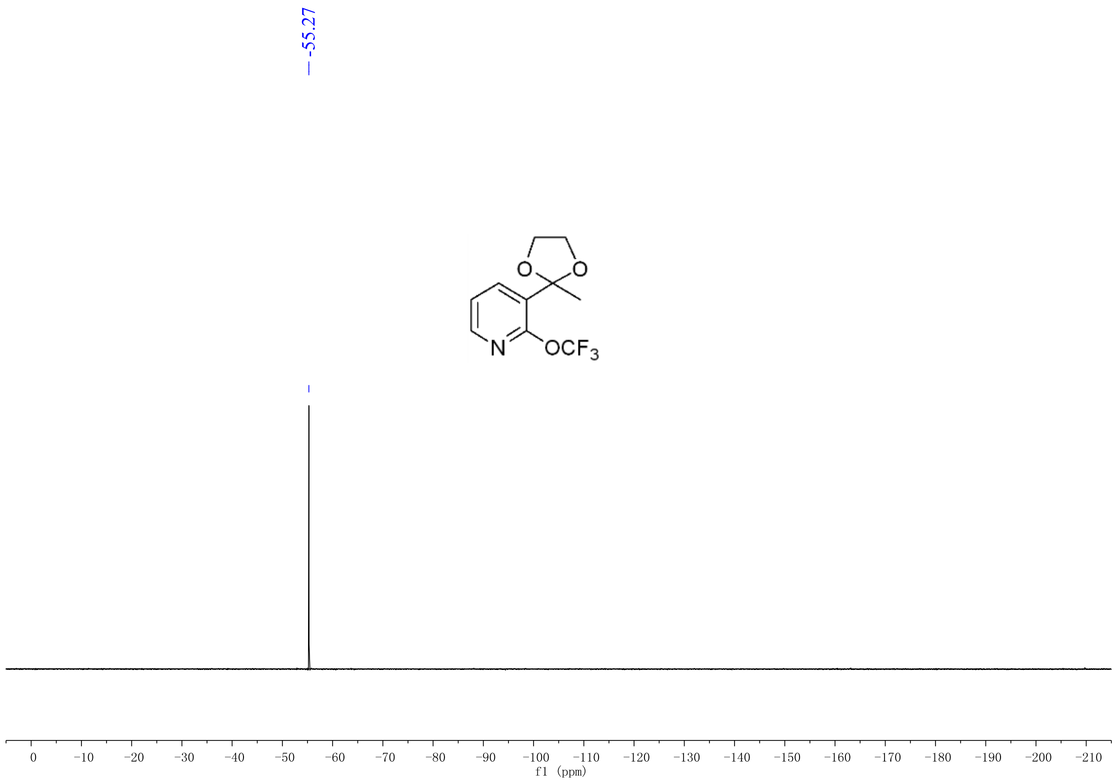

Supplementary Figure 43. ^{19}F NMR spectrum (376 MHz, $CDCl_3$) of **3l**

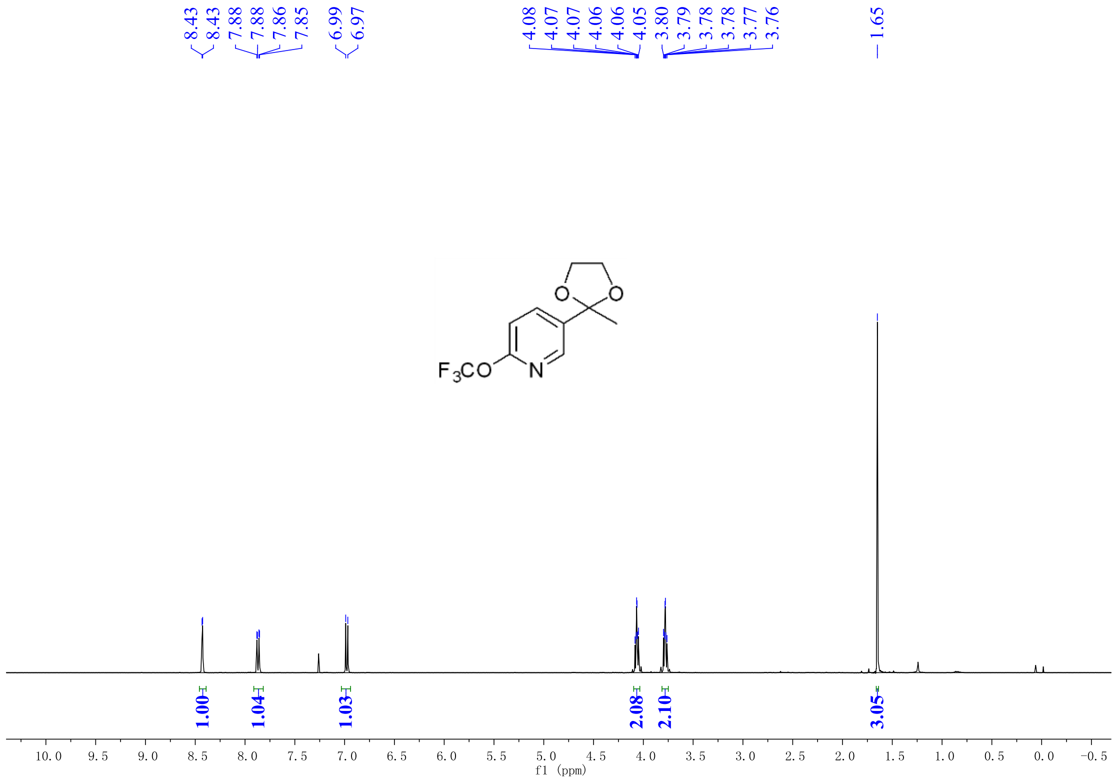

Supplementary Figure 44. ^{1}H NMR spectrum (400 MHz, $CDCl_3$) of ***iso*-3l**

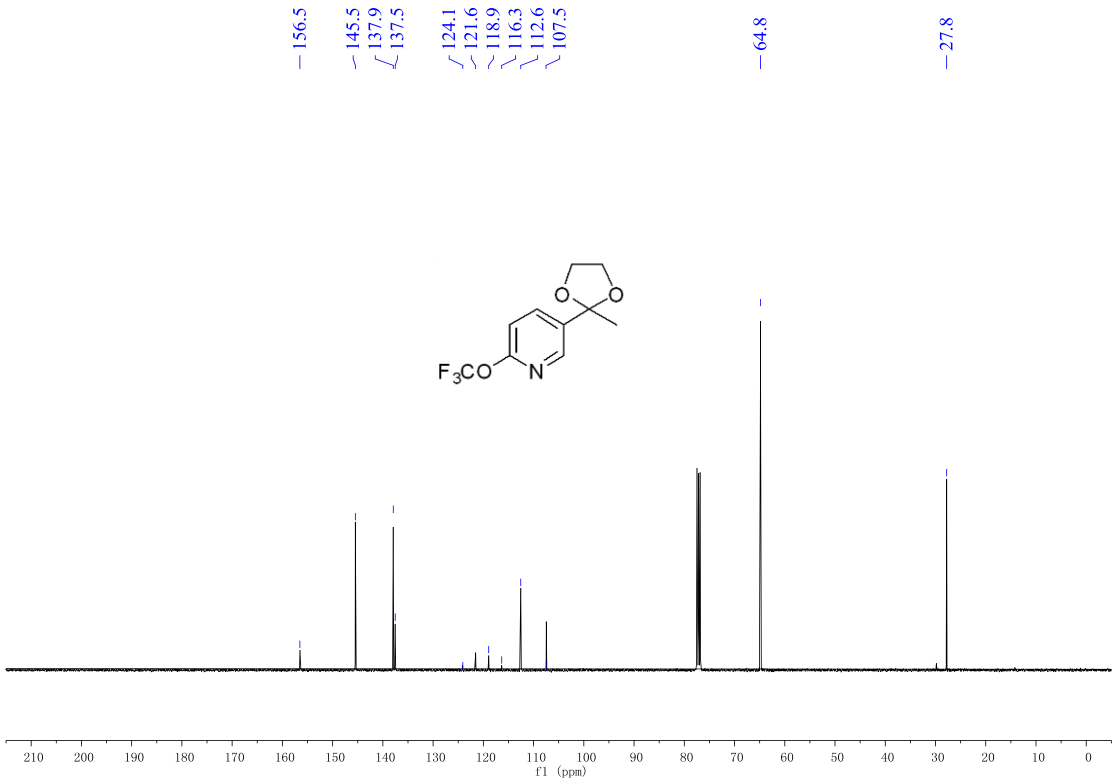

Supplementary Figure 45. ^{13}C NMR spectrum (101 MHz, $CDCl_3$) of ***iso*-3l**

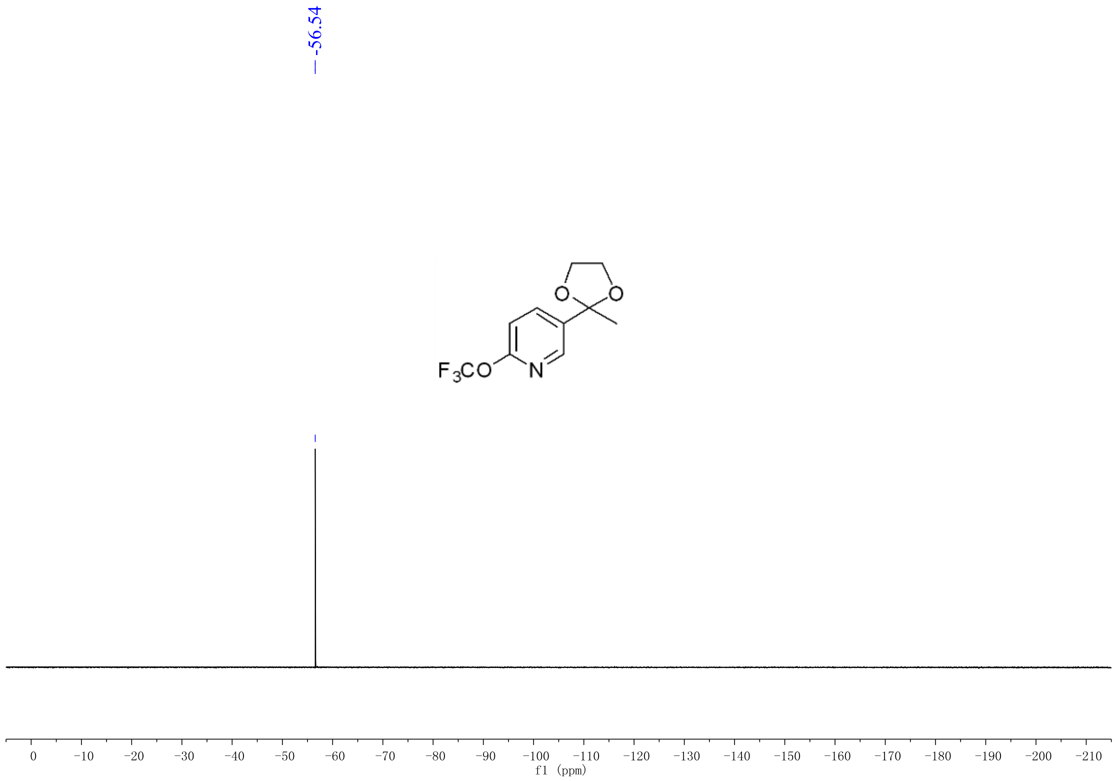

Supplementary Figure 46. ^{19}F NMR spectrum (376 MHz, $CDCl_3$) of ***iso*-3l**

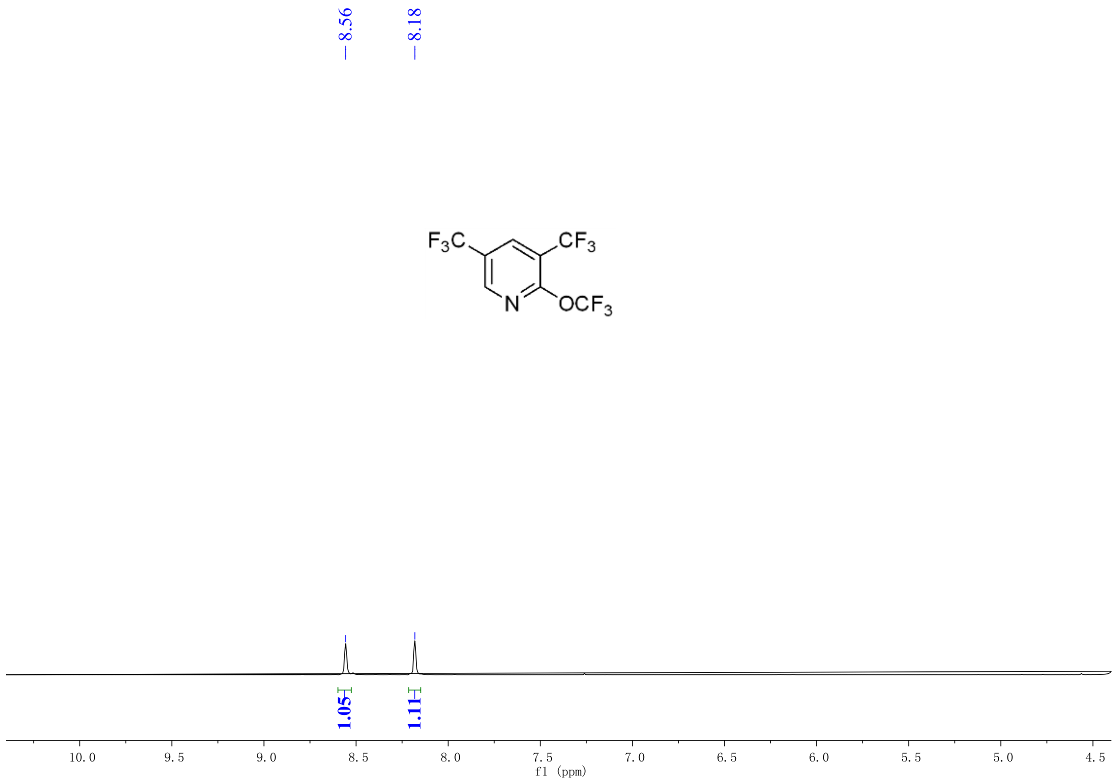

Supplementary Figure 47. ^{1}H NMR spectrum (400 MHz, $CDCl_3/CH_3CN$) of **3m**

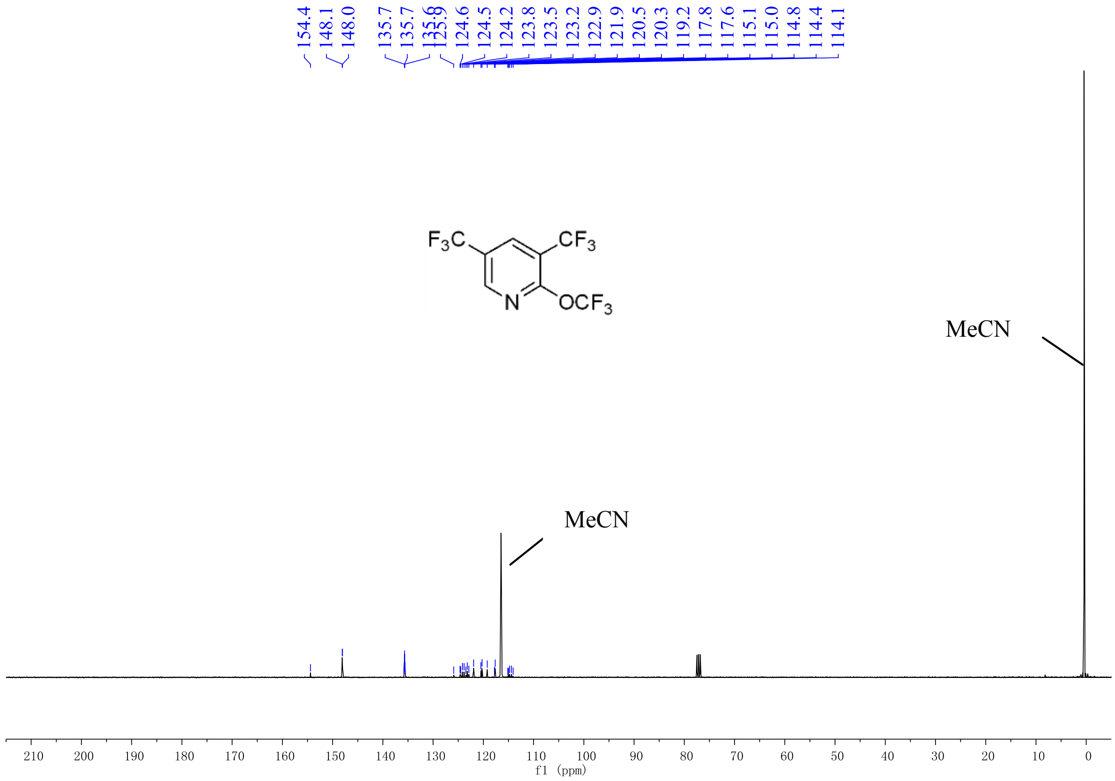

Supplementary Figure 48. ^{13}C NMR spectrum (101 MHz, $CDCl_3/CH_3CN$) of **3m**

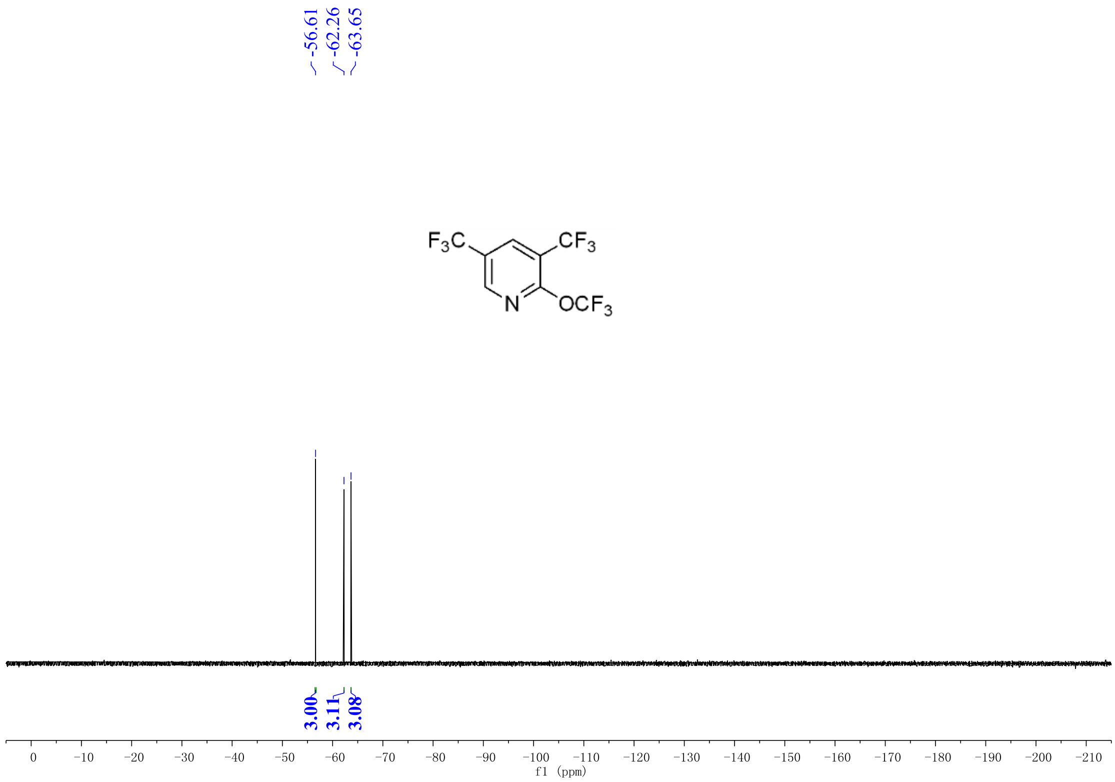

Supplementary Figure 49. ^{19}F NMR spectrum (376 MHz, $CDCl_3/CH_3CN$) of **3m**

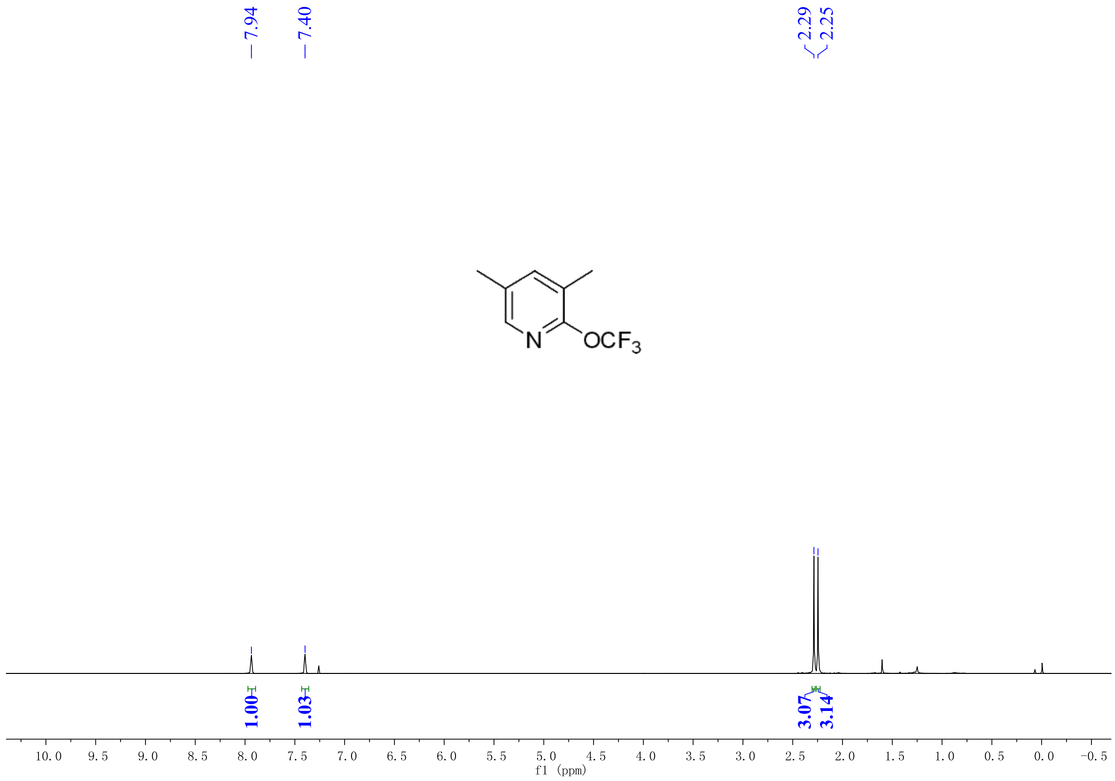

Supplementary Figure 50. ^{1}H NMR spectrum (400 MHz, $CDCl_3$) of **3n**

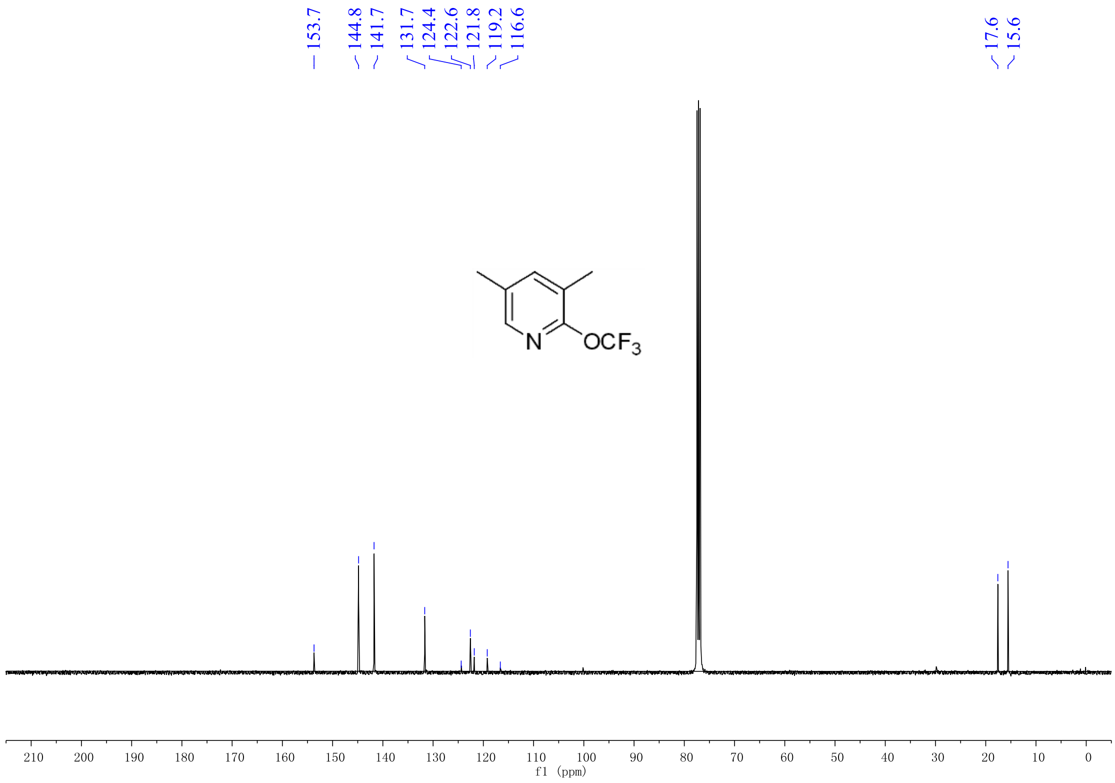

Supplementary Figure 51. ^{13}C NMR spectrum (101 MHz, $CDCl_3$) of **3n**

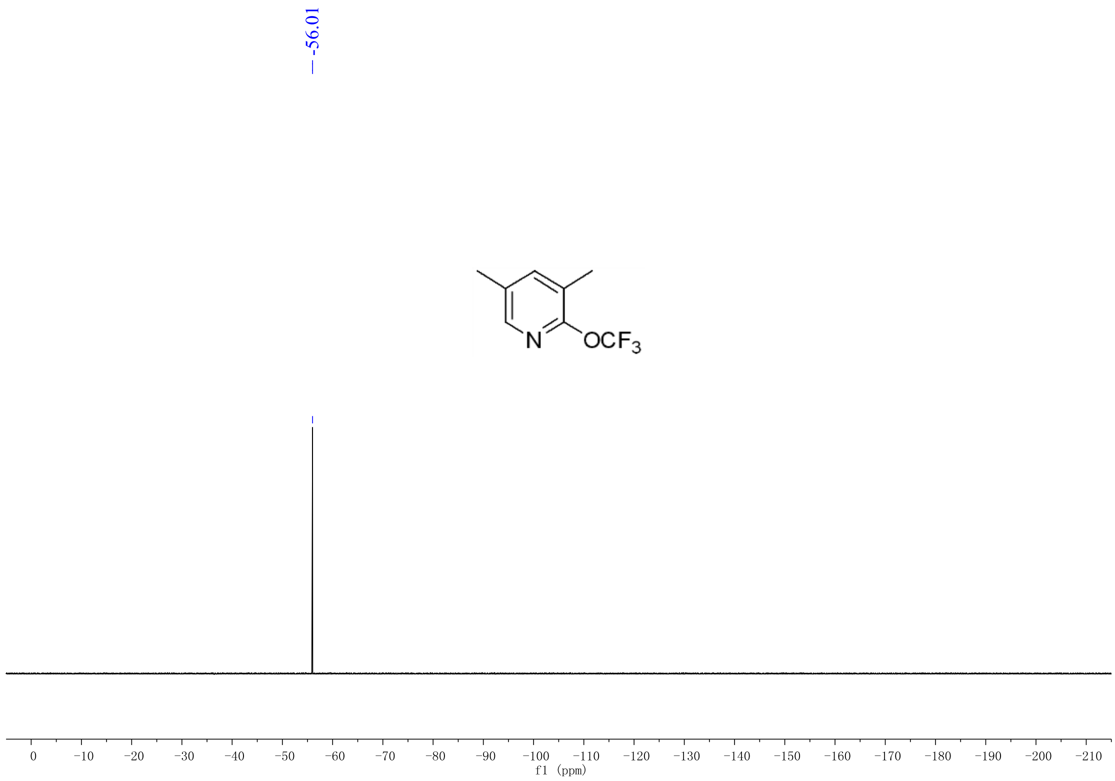

Supplementary Figure 52. ^{19}F NMR spectrum (376 MHz, $CDCl_3$) of **3n**

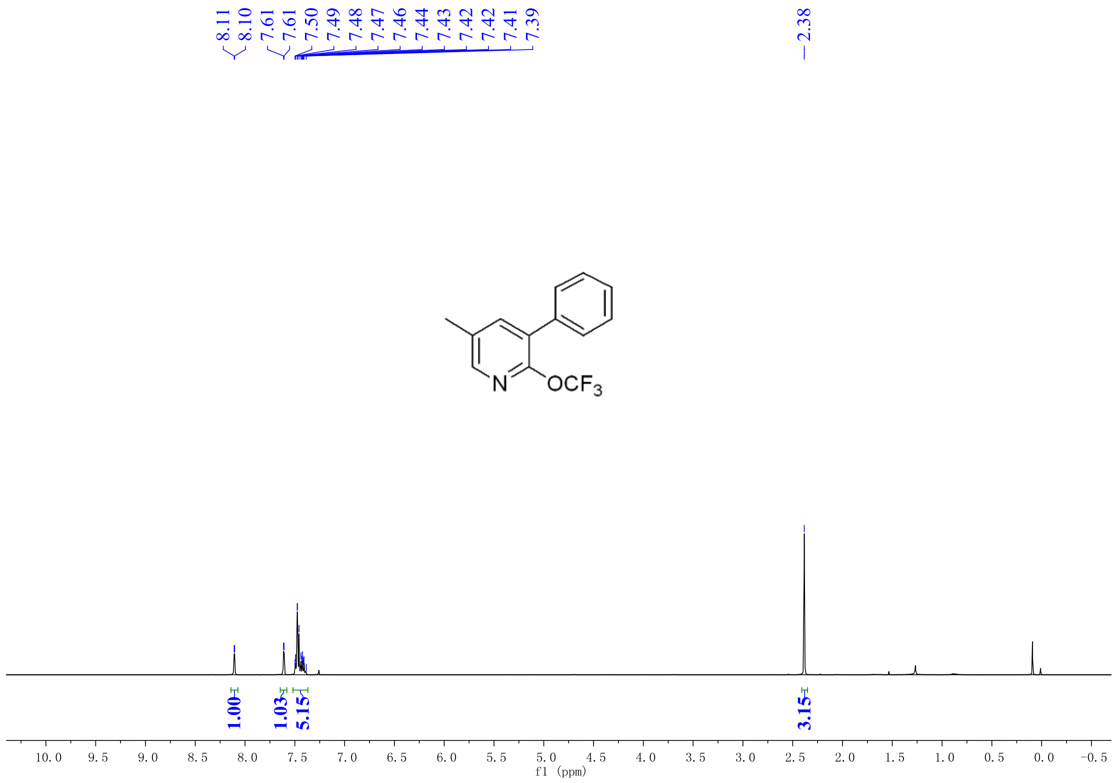

Supplementary Figure 53. ^{1}H NMR spectrum (400 MHz, $CDCl_3$) of **3o**

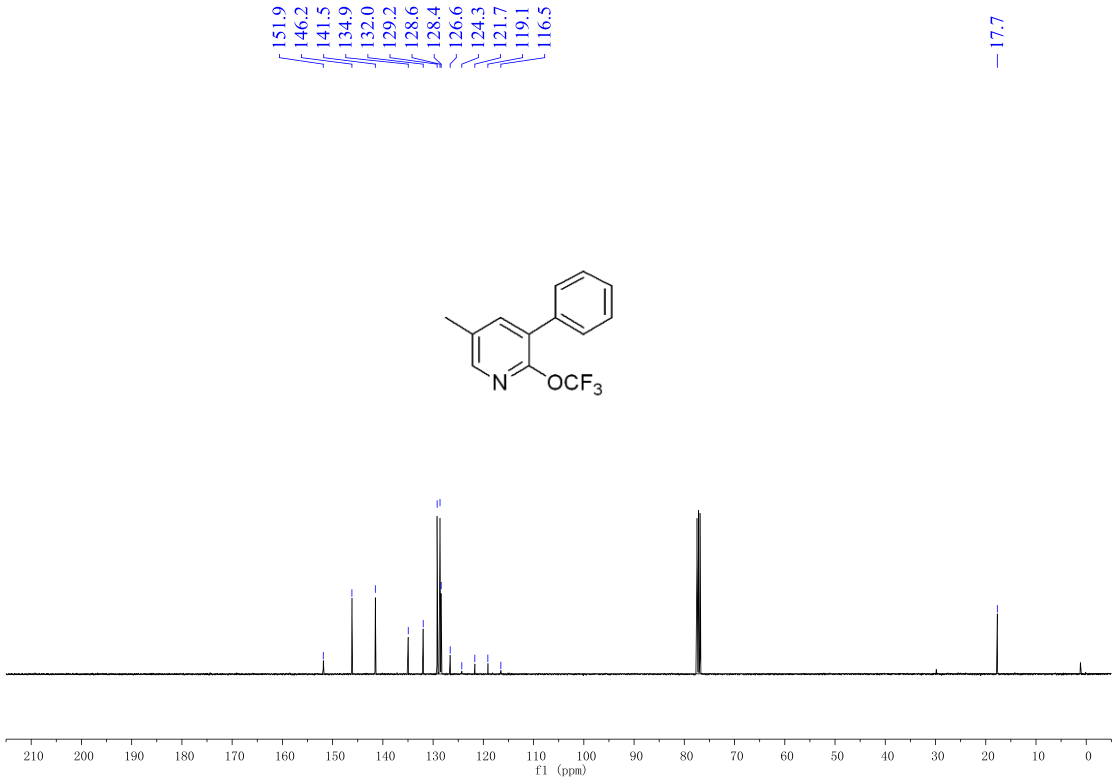

Supplementary Figure 54. ^{13}C NMR spectrum (101 MHz, $CDCl_3$) of **3o**

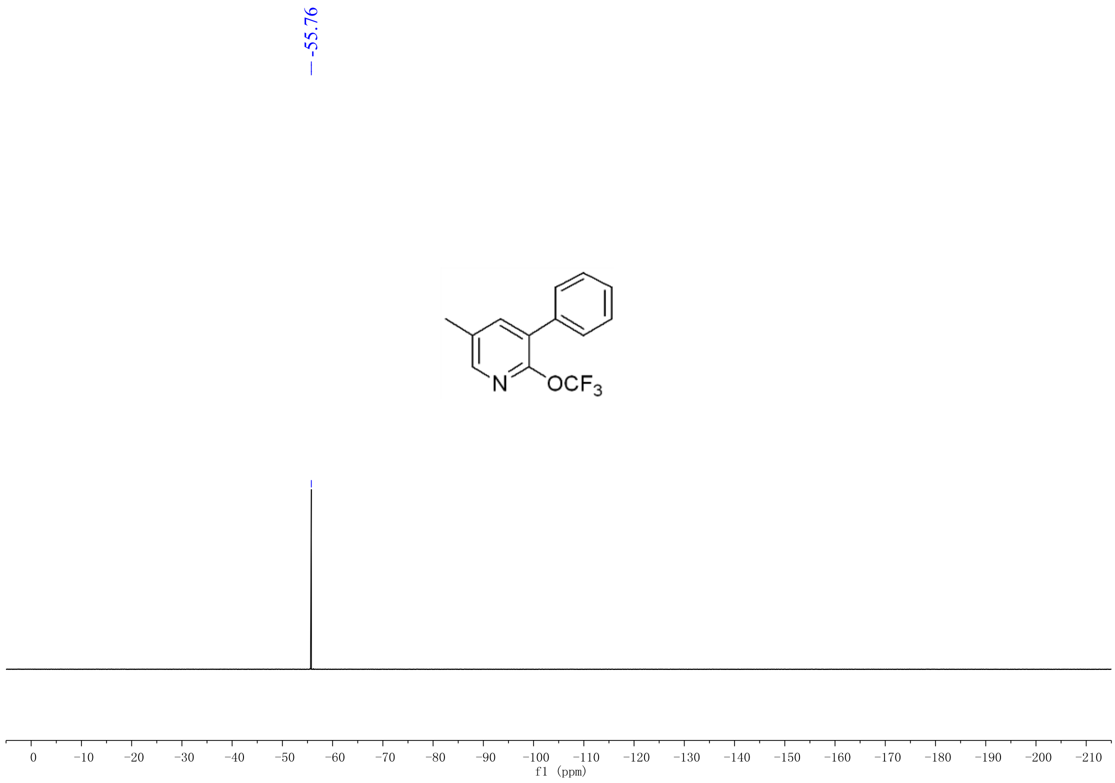

Supplementary Figure 55. ^{19}F NMR spectrum (376 MHz, $CDCl_3$) of **3o**

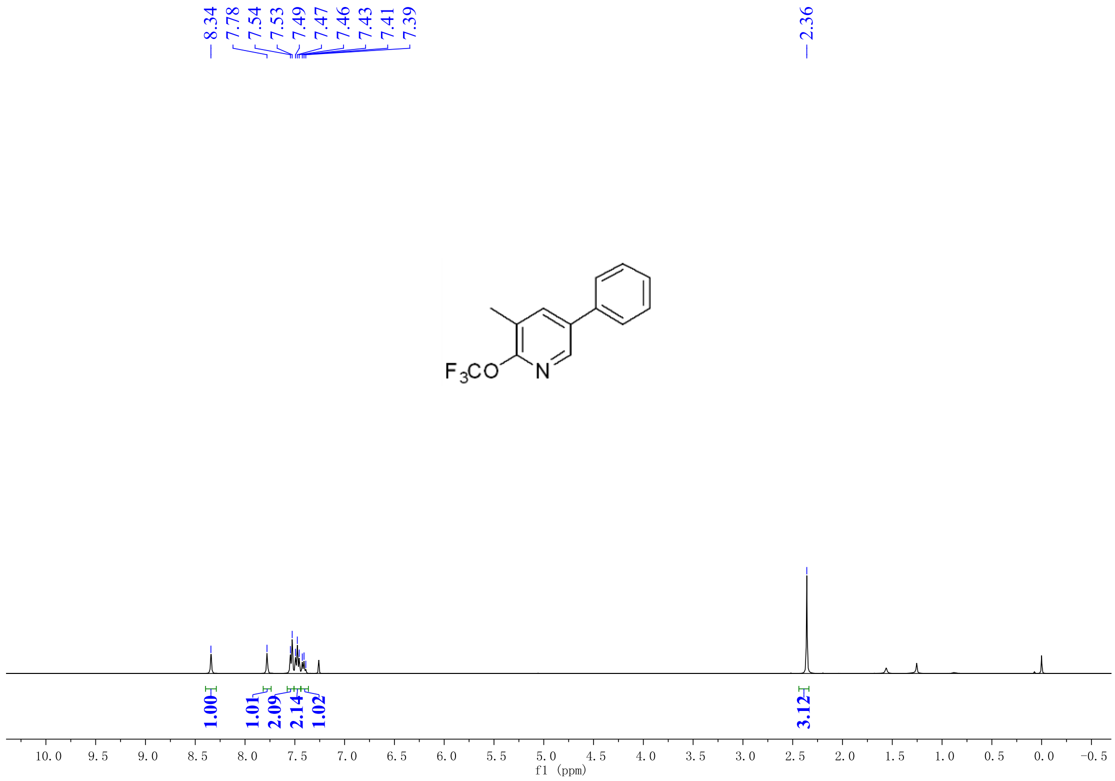

Supplementary Figure 56. ^{1}H NMR spectrum (400 MHz, $CDCl_3$) of ***iso*-3o**

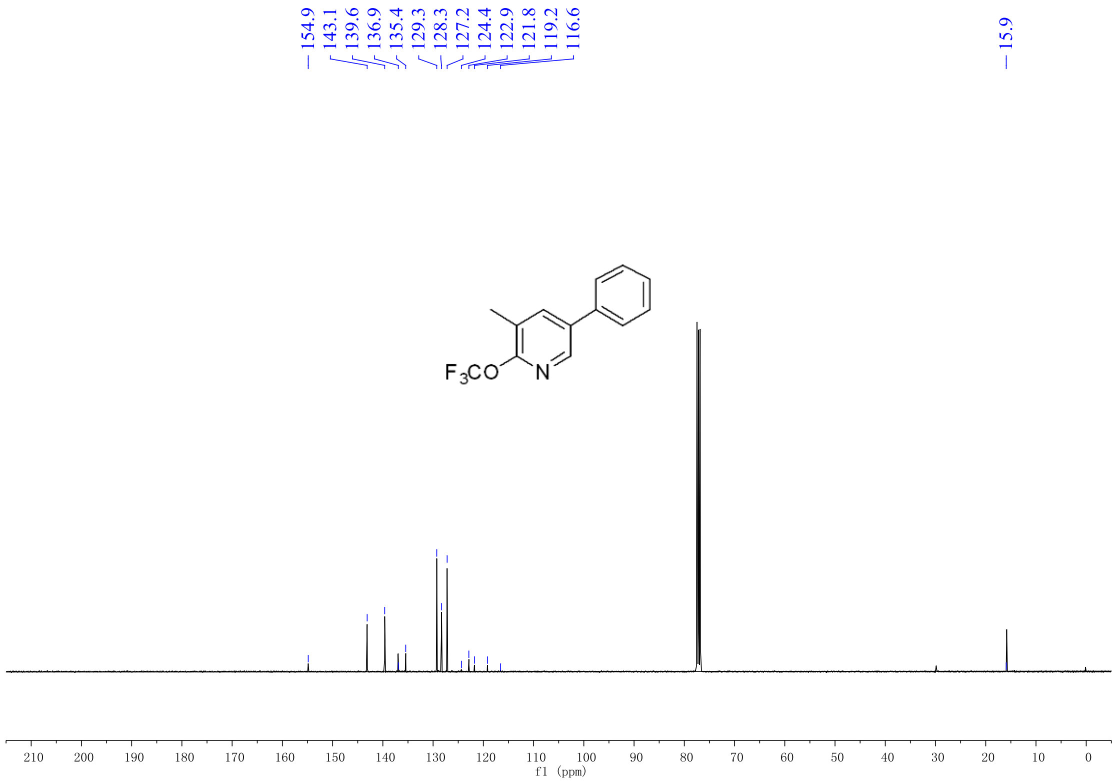

Supplementary Figure 57. ^{13}C NMR spectrum (101 MHz, $CDCl_3$) of ***iso*-3o**

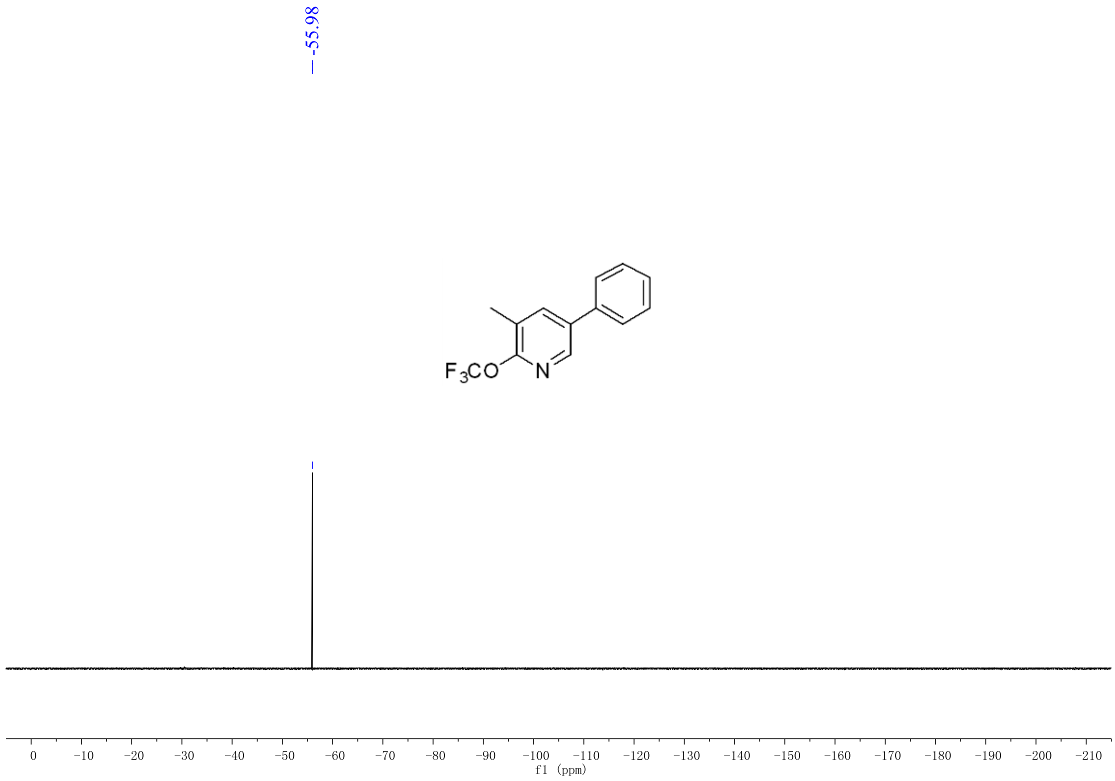

Supplementary Figure 58. ^{19}F NMR spectrum (376 MHz, $CDCl_3$) of ***iso*-3o**

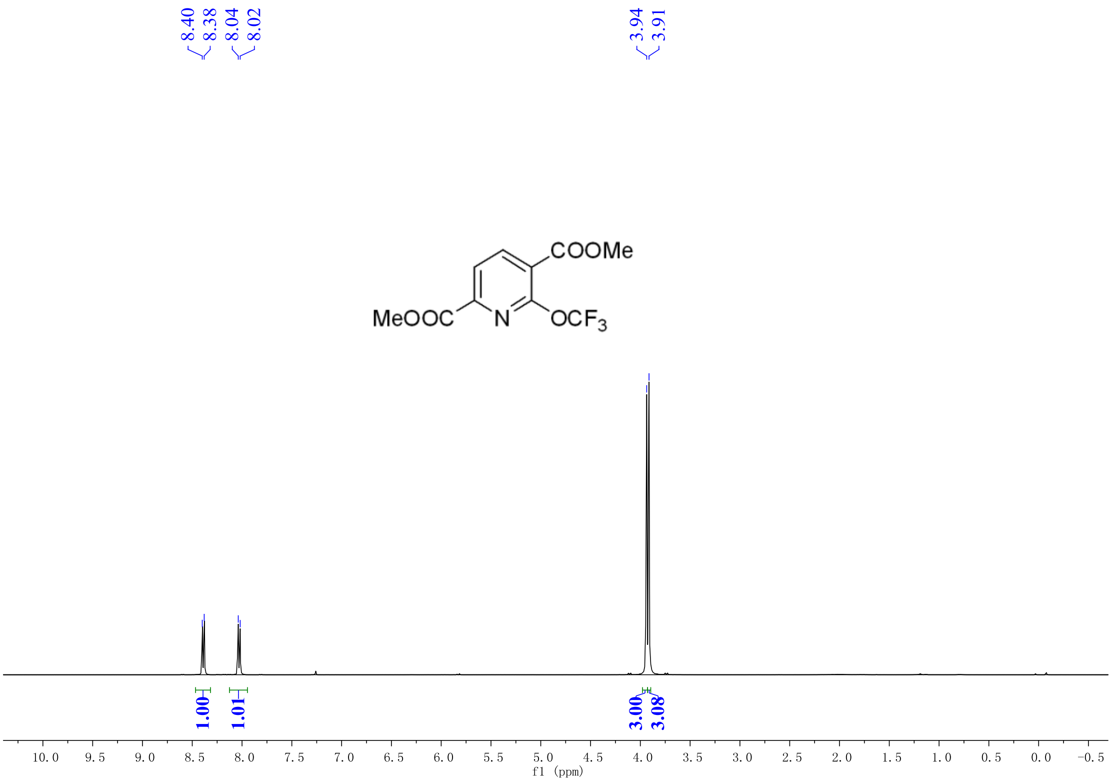

Supplementary Figure 59. ^{1}H NMR spectrum (400 MHz, $CDCl_3$) of **3p**

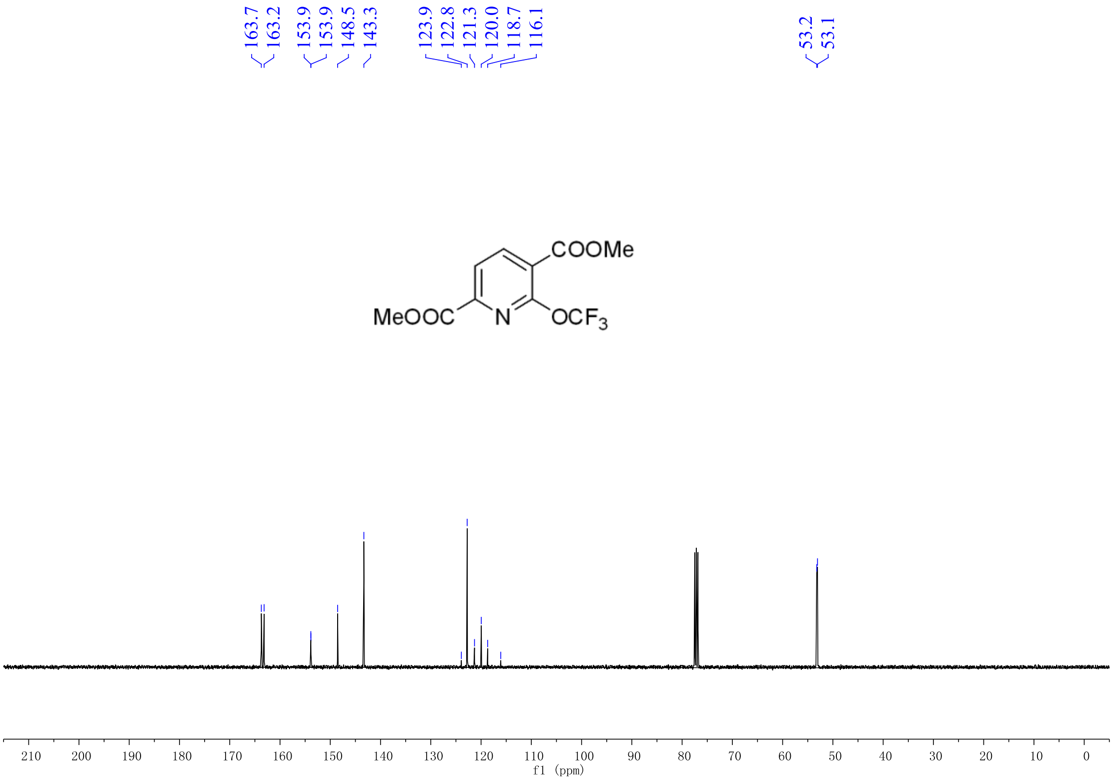

Supplementary Figure 60. ^{13}C NMR spectrum (101 MHz, $CDCl_3$) of **3p**

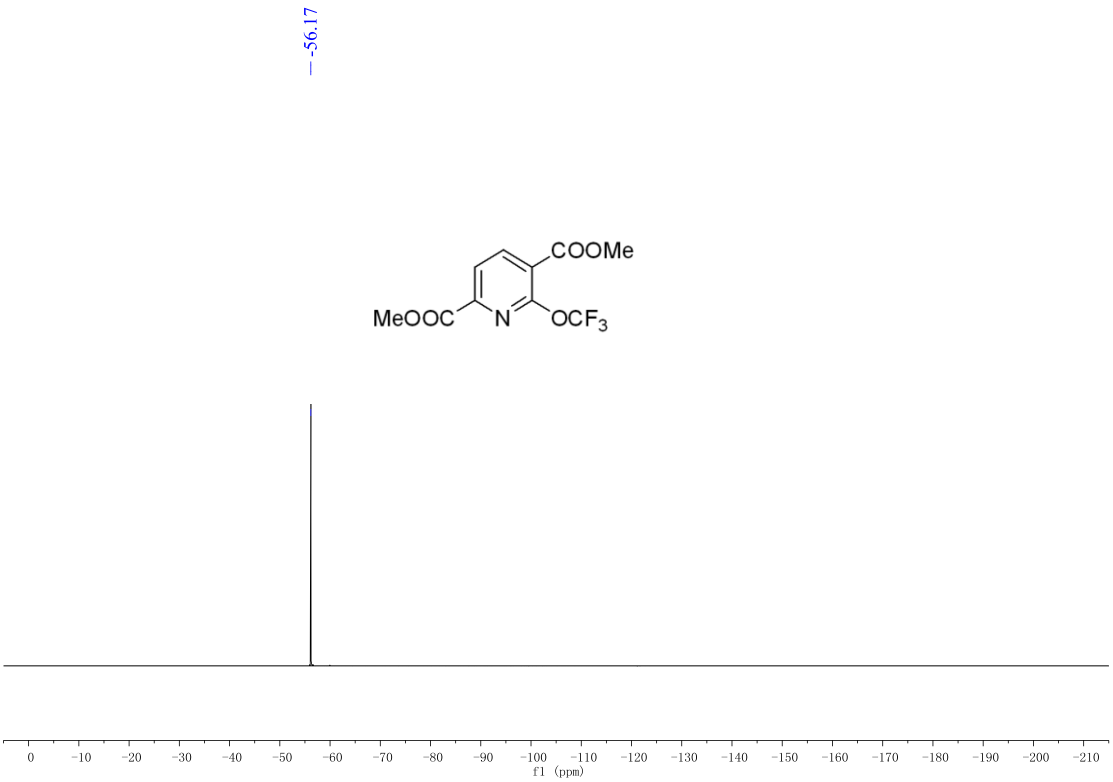

Supplementary Figure 61. ^{19}F NMR spectrum (376 MHz, $CDCl_3$) of **3p**

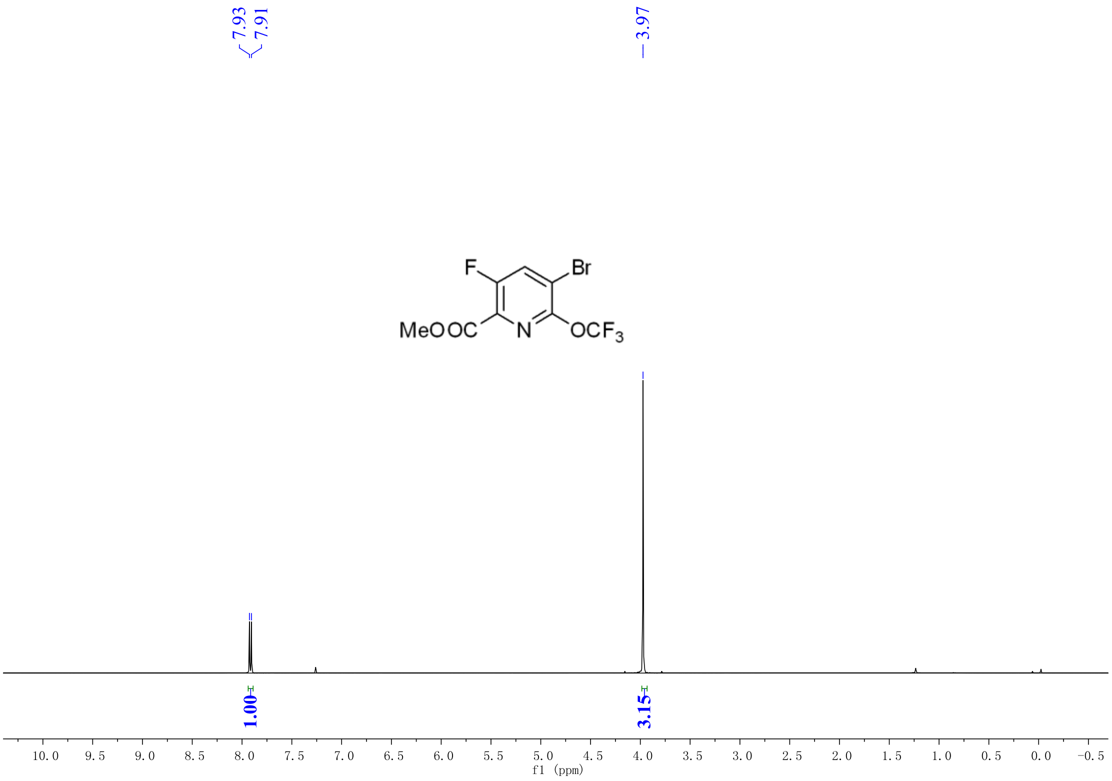

Supplementary Figure 62. ^{1}H NMR spectrum (400 MHz, $CDCl_3$) of **3q**

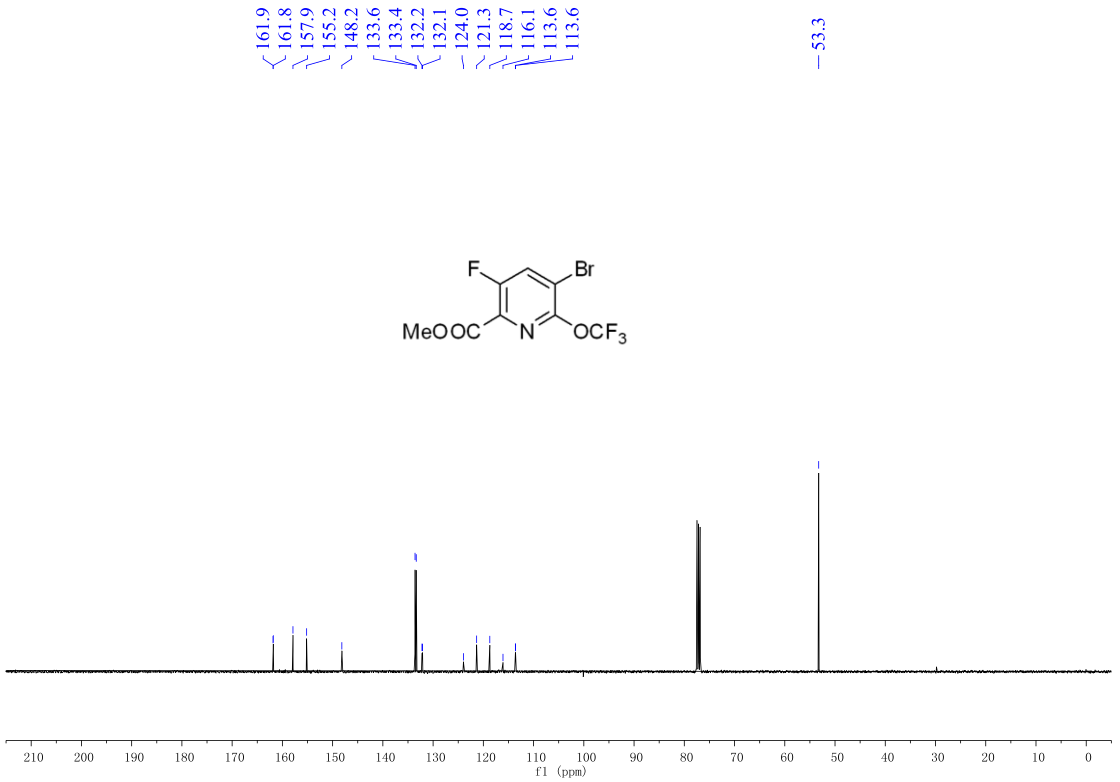

Supplementary Figure 63. ^{13}C NMR spectrum (101 MHz, $CDCl_3$) of **3q**

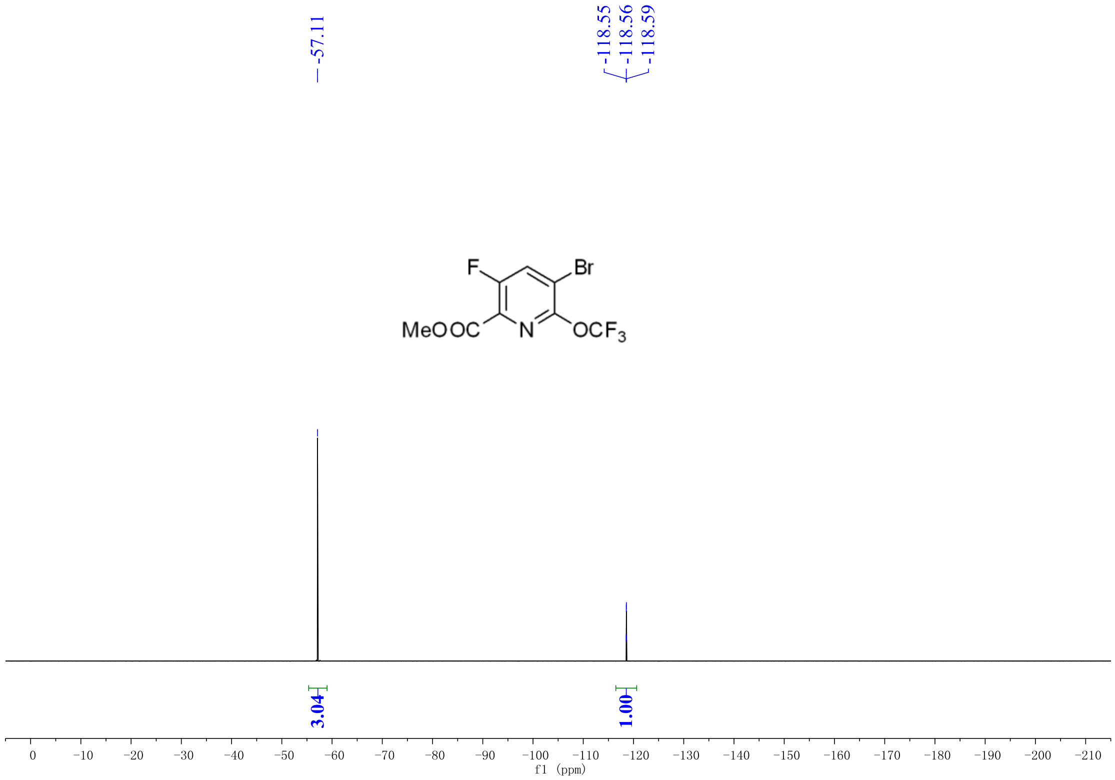

Supplementary Figure 64. ^{19}F NMR spectrum (376 MHz, $CDCl_3$) of **3q**

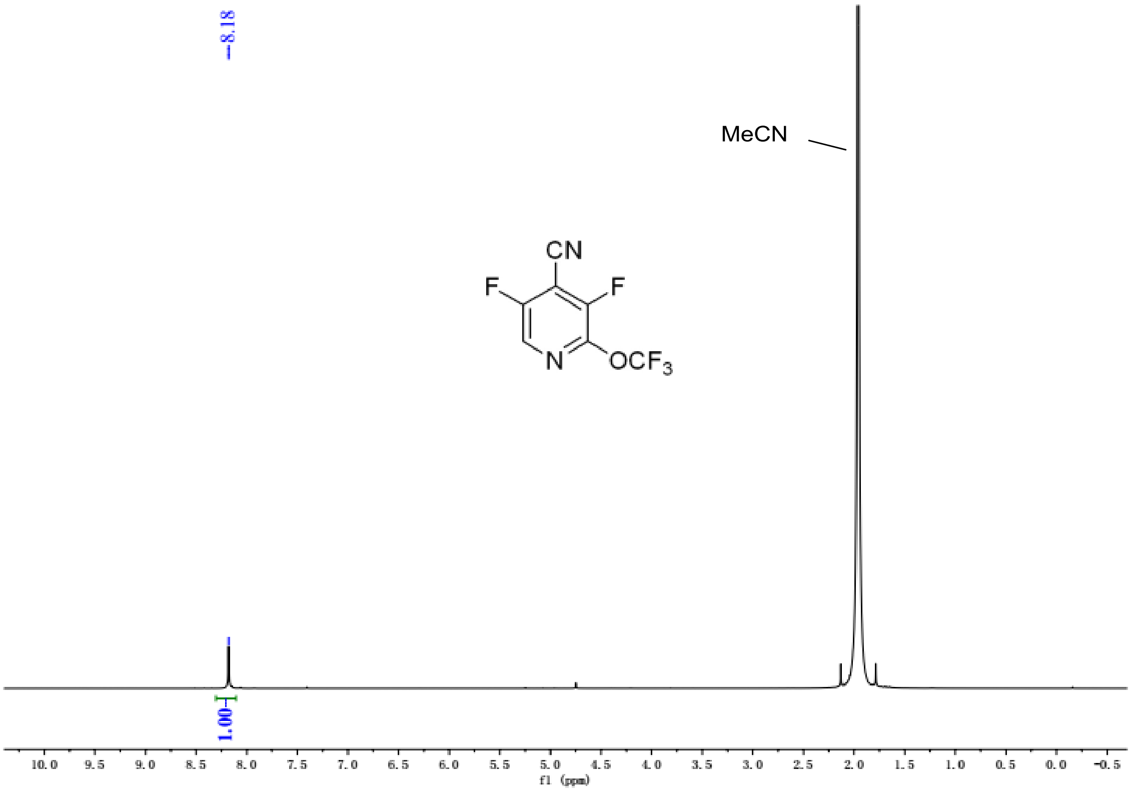

Supplementary Figure 65. ^{1}H NMR spectrum (400 MHz, $CDCl_3/CH_3CN$) of **3r**

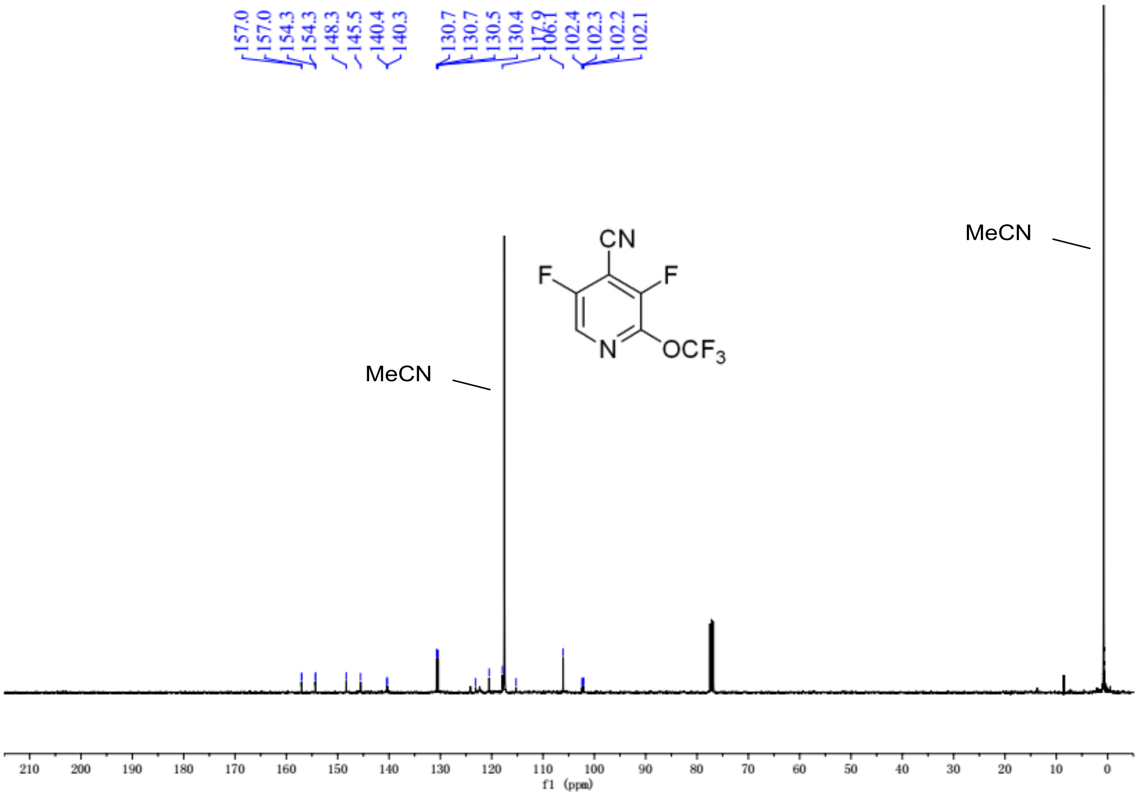

Supplementary Figure 66. ^{13}C NMR spectrum (101 MHz, $CDCl_3/CH_3CN$) of **3r**

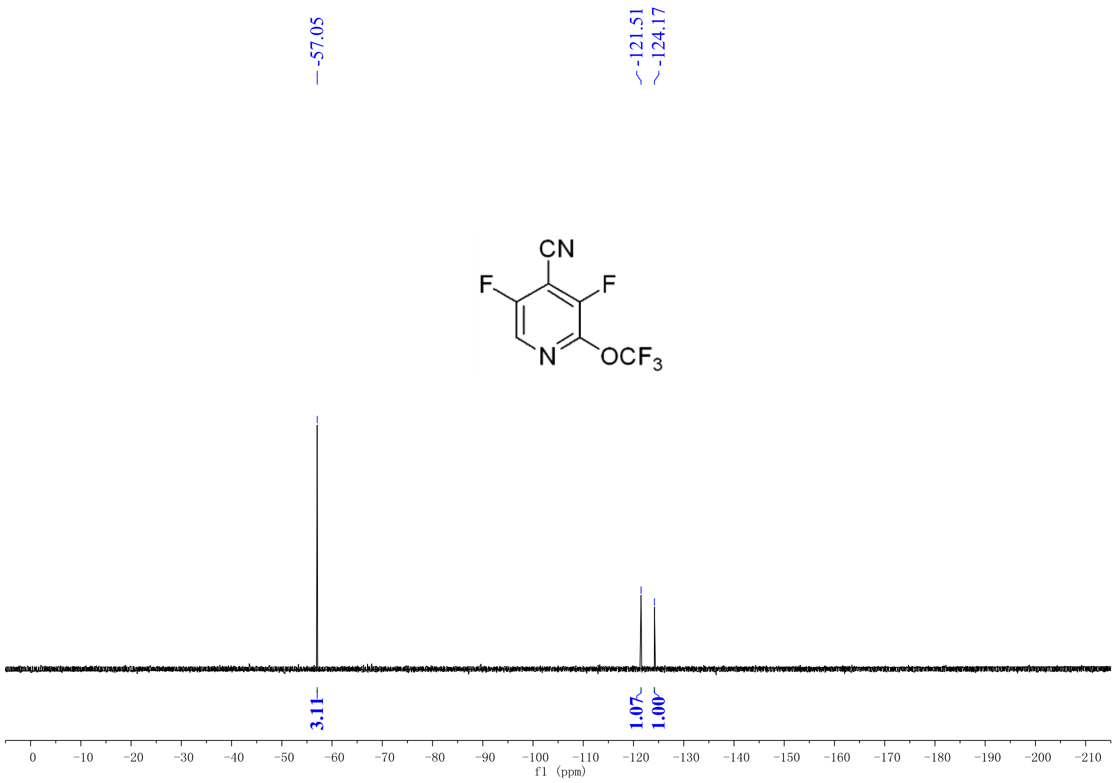

Supplementary Figure 67. ^{19}F NMR spectrum (376 MHz, $CDCl_3/CH_3CN$) of **3r**

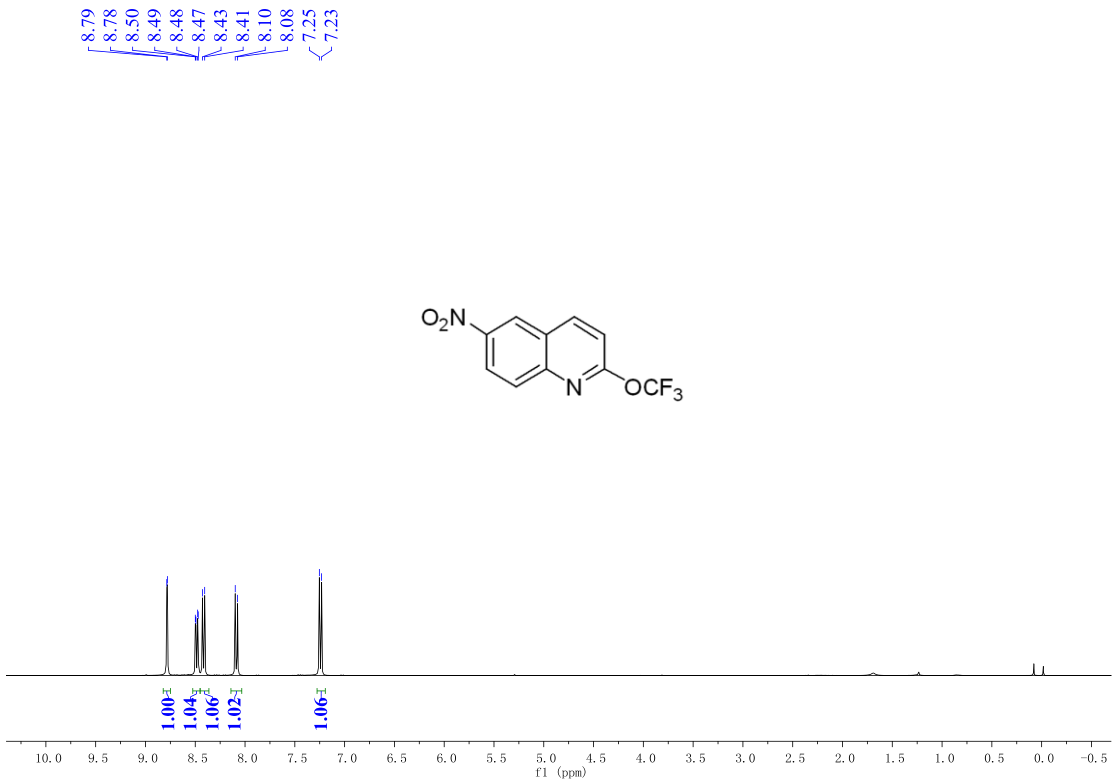

Supplementary Figure 68. ^{1}H NMR spectrum (400 MHz, $CDCl_3$) of **3s**

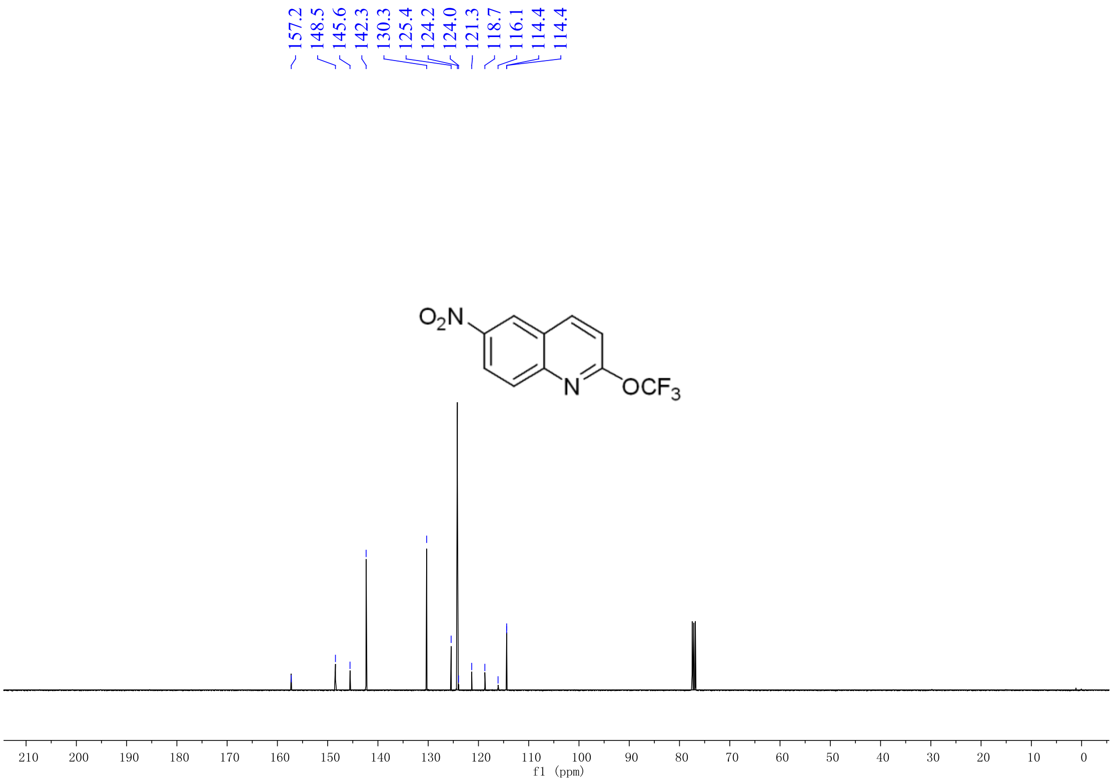

Supplementary Figure 69. ^{13}C NMR spectrum (101 MHz, $CDCl_3$) of **3s**

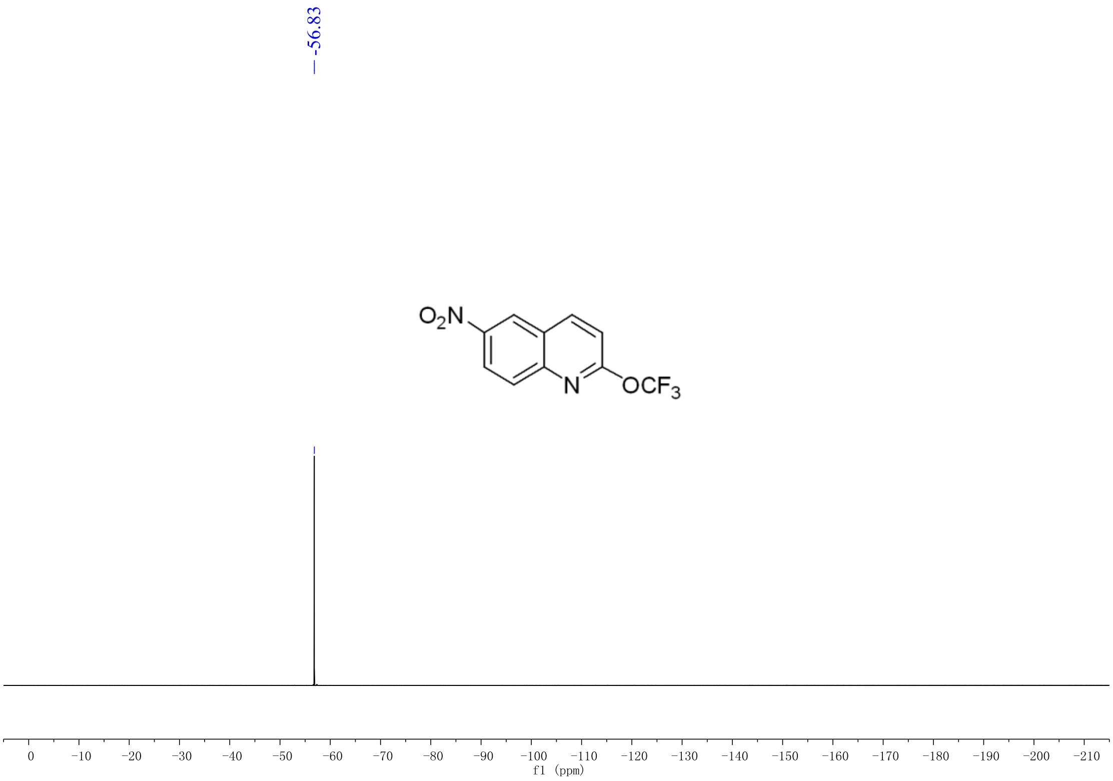

Supplementary Figure 70. ^{19}F NMR spectrum (376 MHz, $CDCl_3$) of **3s**

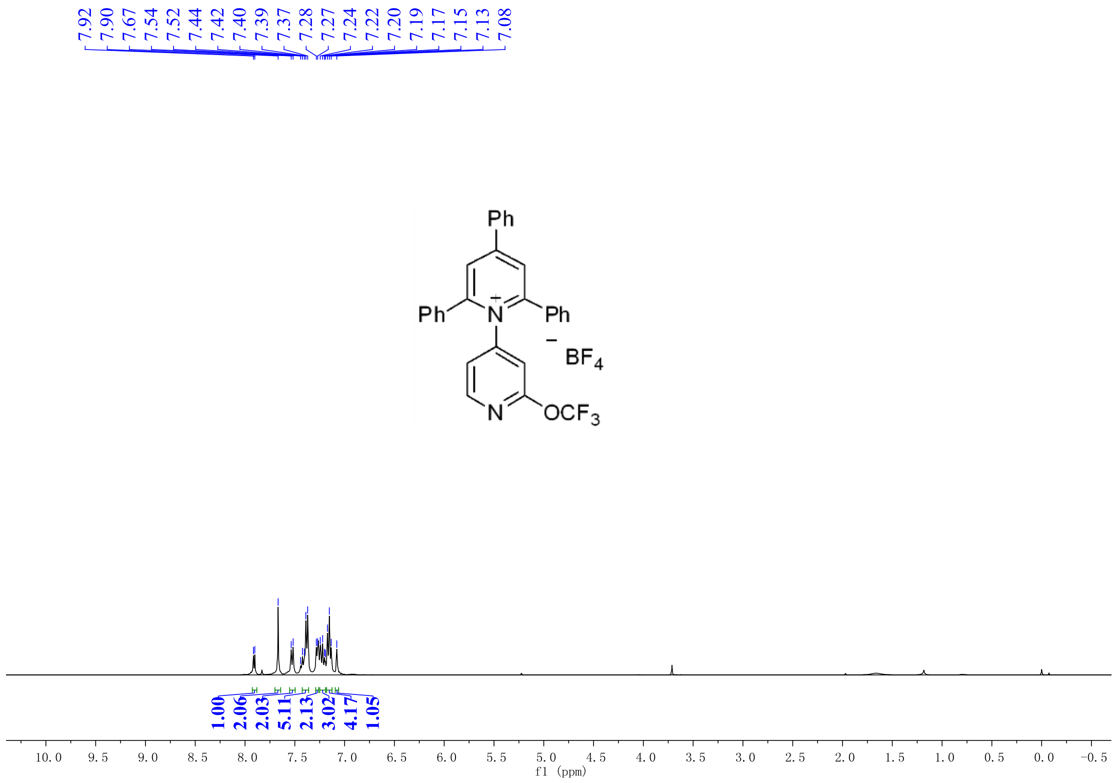

Supplementary Figure 71. ^{1}H NMR spectrum (400 MHz, $CDCl_3$) of **3t**

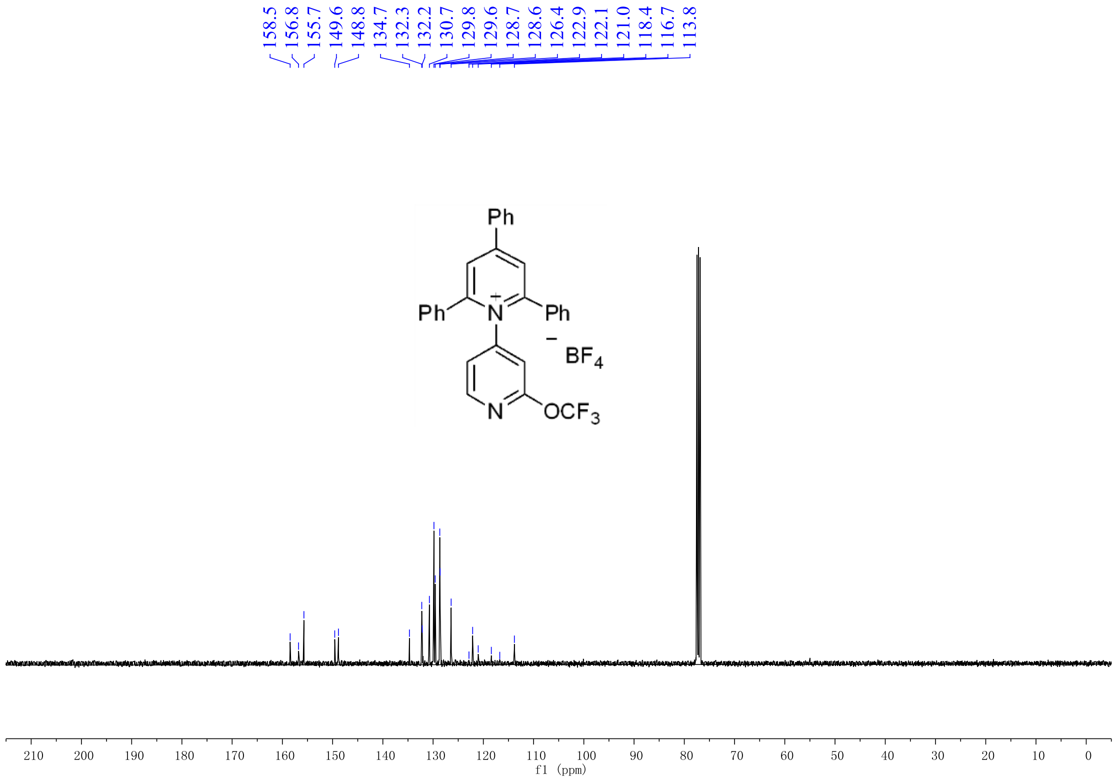

Supplementary Figure 72. ^{13}C NMR spectrum (101 MHz, $CDCl_3$) of **3t**

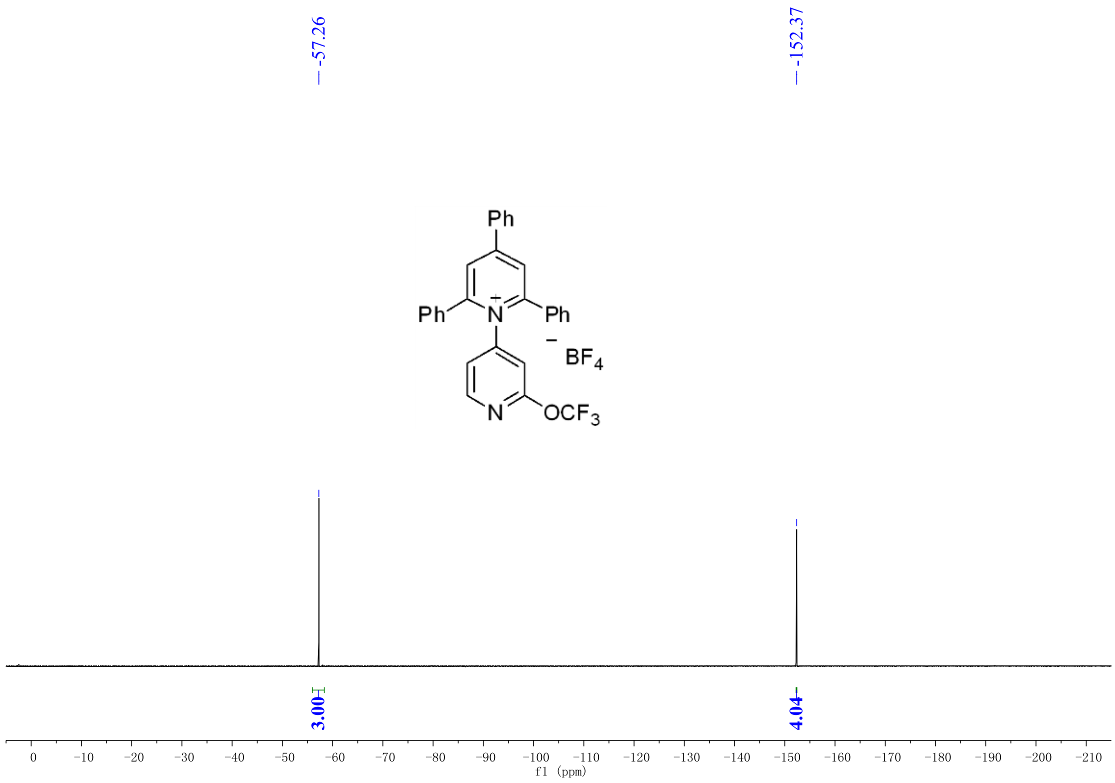

Supplementary Figure 73. ^{19}F NMR spectrum (376 MHz, $CDCl_3$) of **3t**

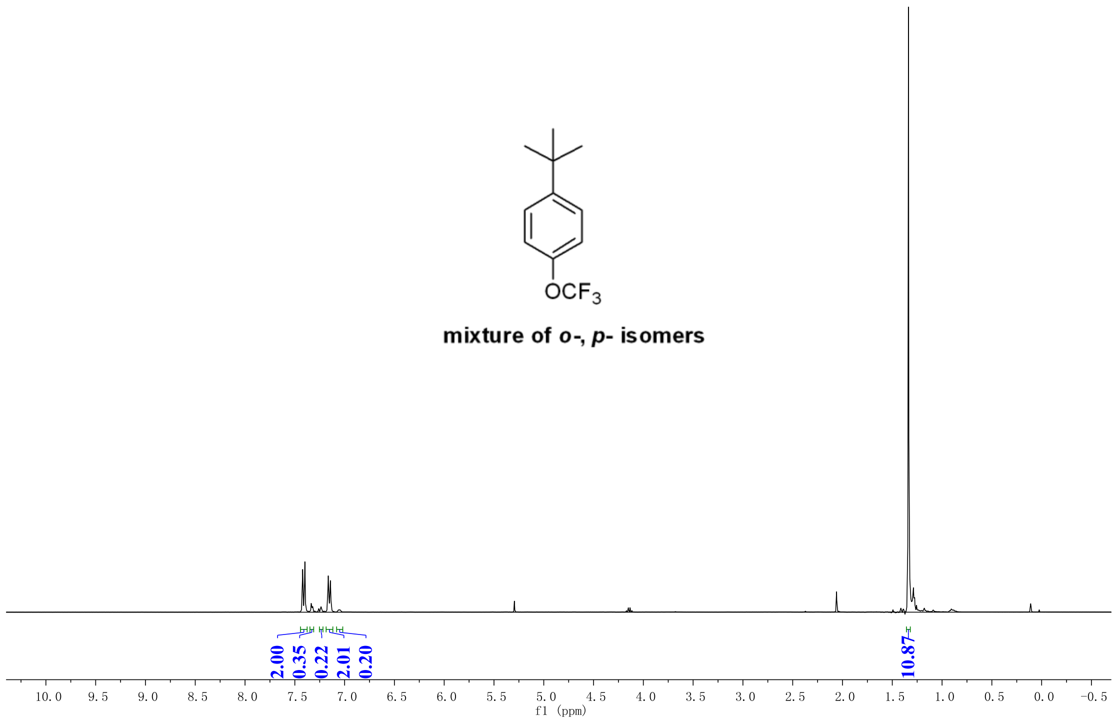

Supplementary Figure 74. ^{1}H NMR spectrum (400 MHz, $CDCl_3$) for a mixture of **3u** and ***iso*-3u**

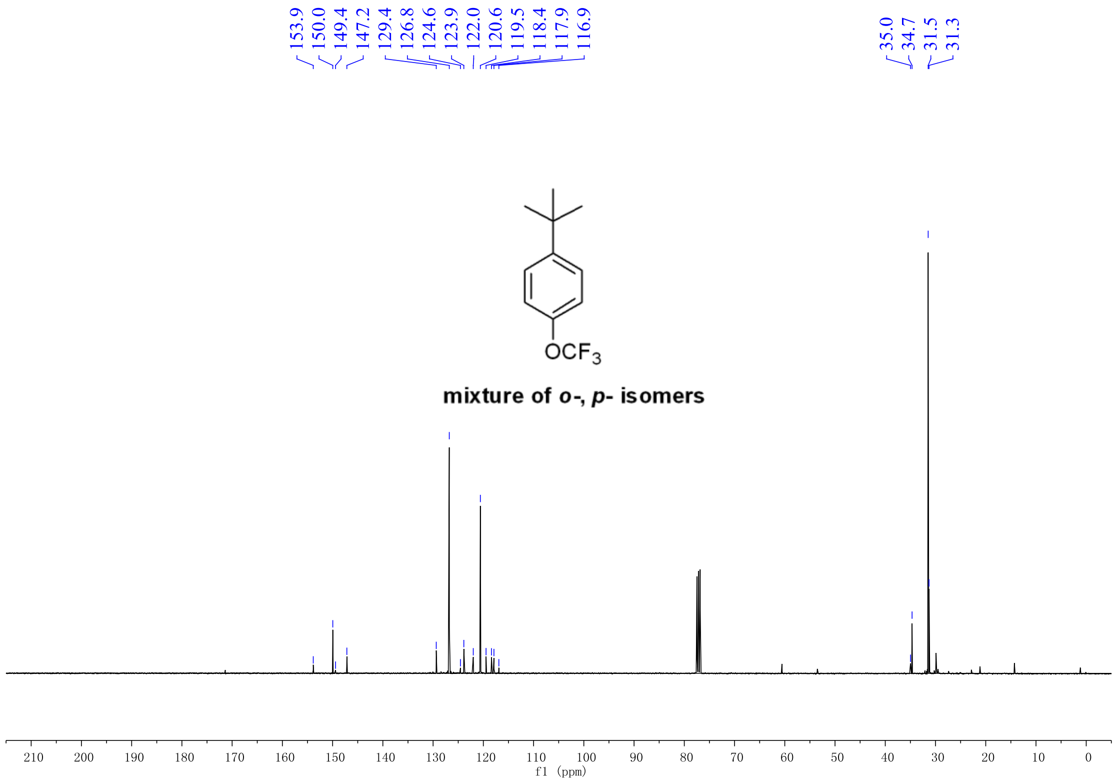

Supplementary Figure 75. ^{13}C NMR spectrum (101 MHz, $CDCl_3$) for a mixture of **3u** and ***iso*-3u**

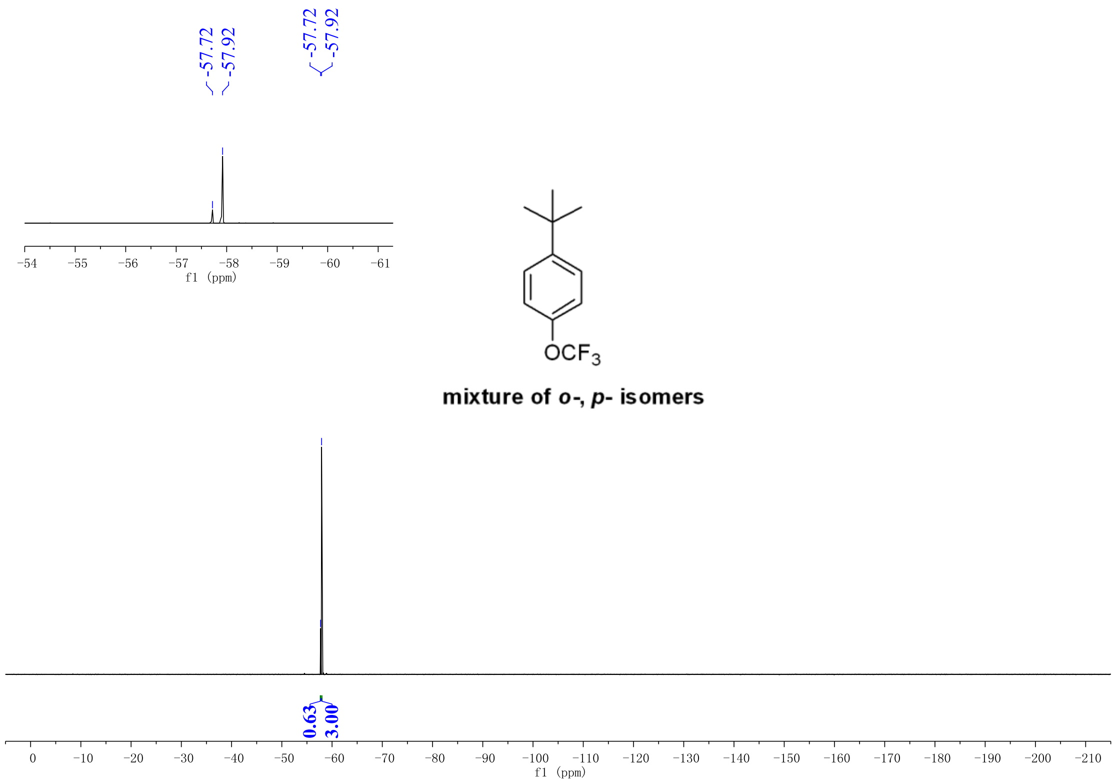

Supplementary Figure 76. ^{19}F NMR spectrum (376 MHz, $CDCl_3$) for a mixture of **3u** and ***iso*-3u**

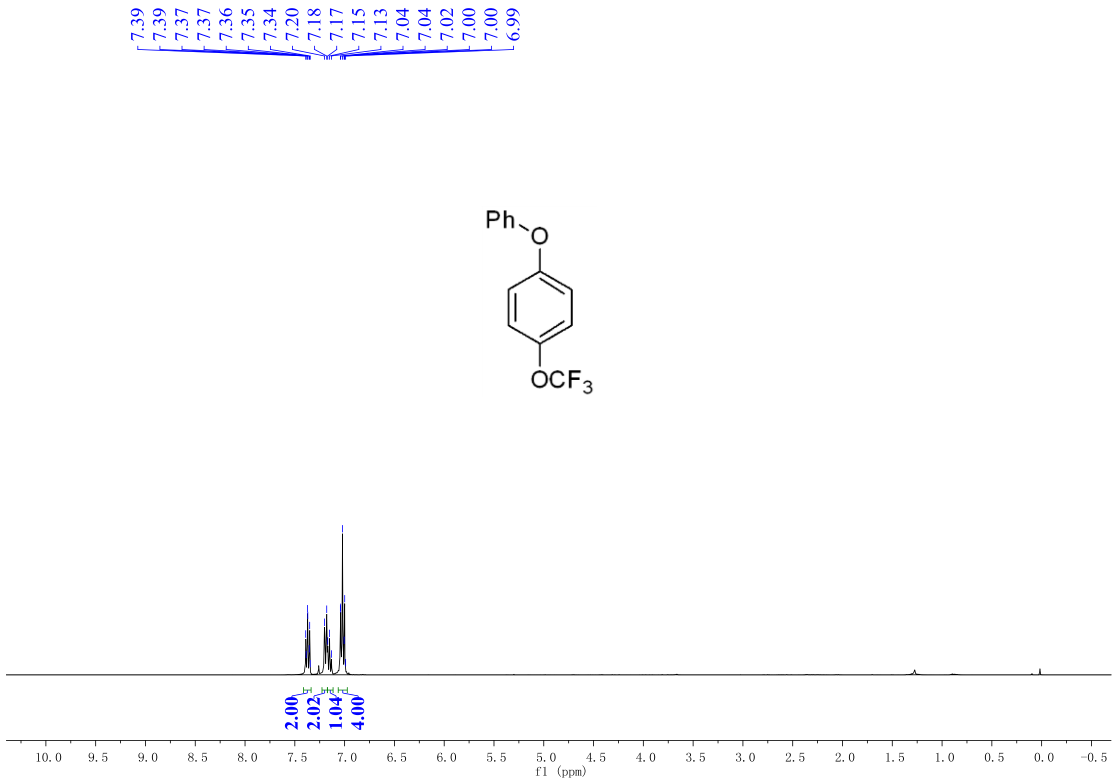

Supplementary Figure 77. ^{1}H NMR spectrum (400 MHz, $CDCl_3$) of **3v**

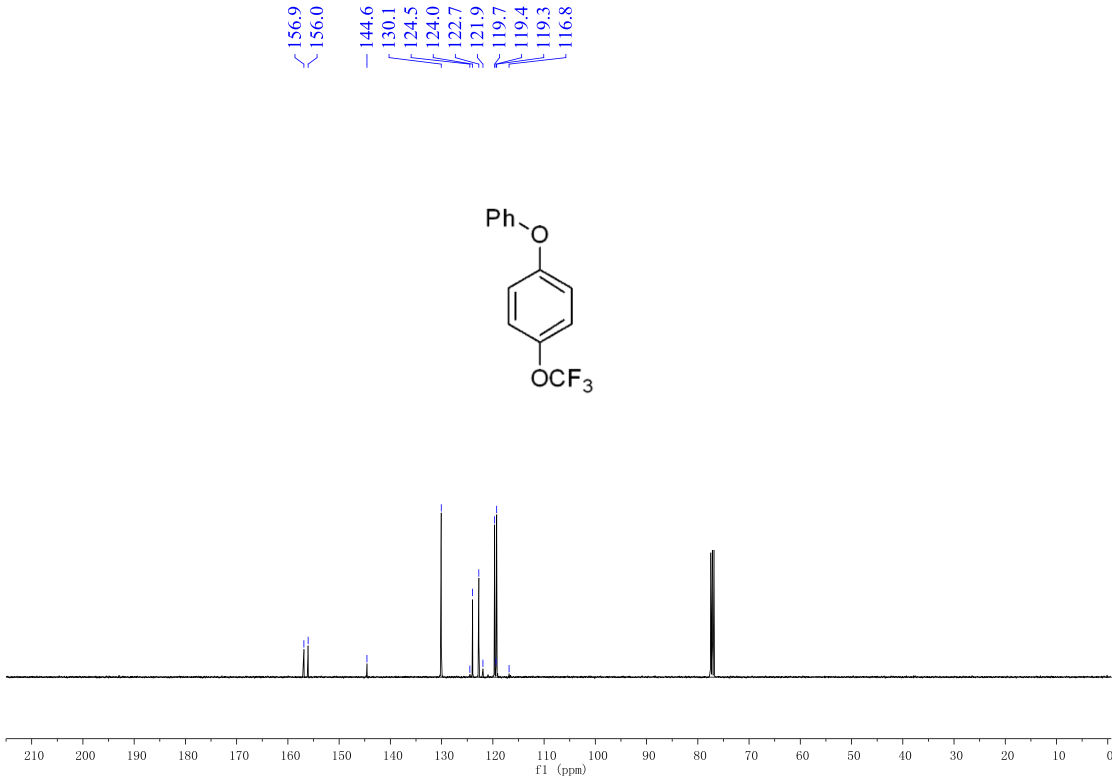

Supplementary Figure 78. ^{13}C NMR spectrum (101 MHz, $CDCl_3$) of **3v**

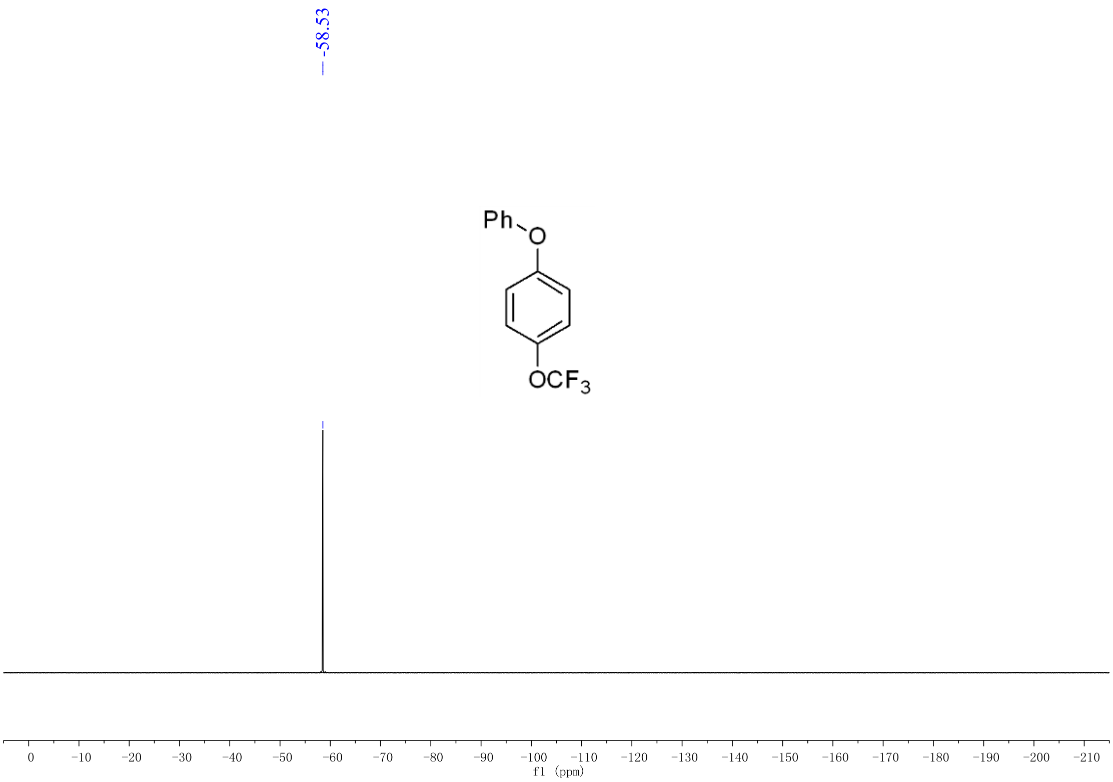

Supplementary Figure 79. ^{19}F NMR spectrum (376 MHz, $CDCl_3$) of **3v**

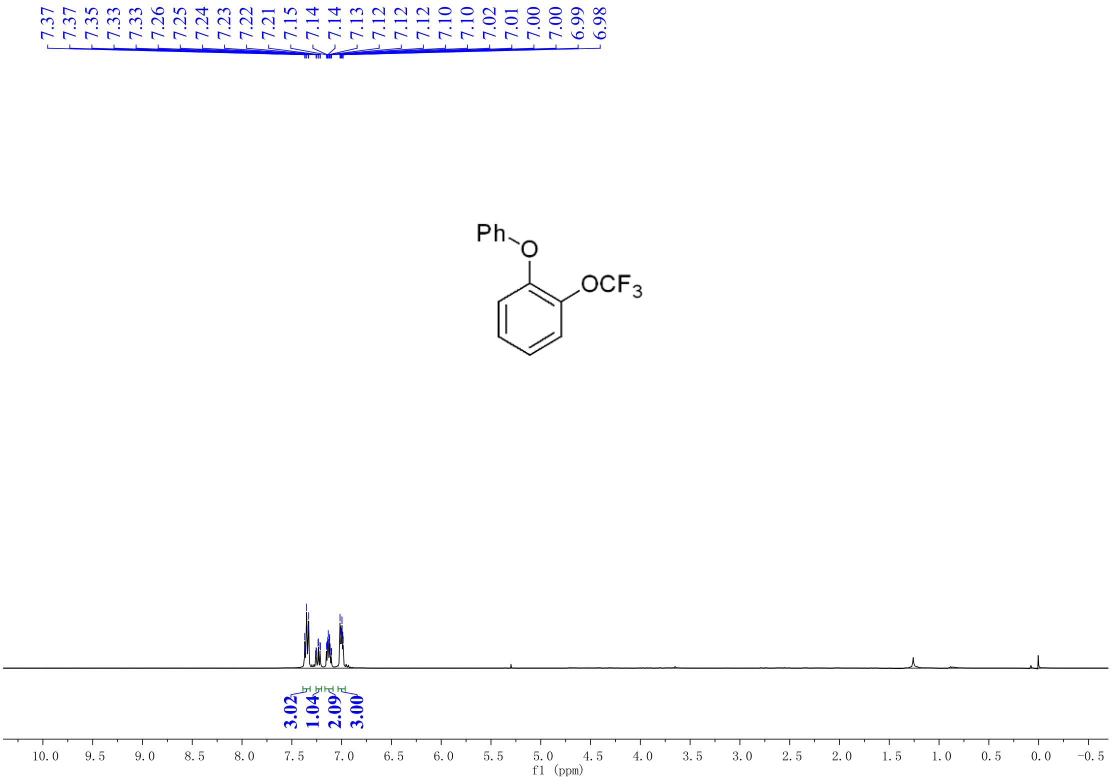

Supplementary Figure 80. ^{1}H NMR spectrum (400 MHz, $CDCl_3$) of ***iso*-3v**

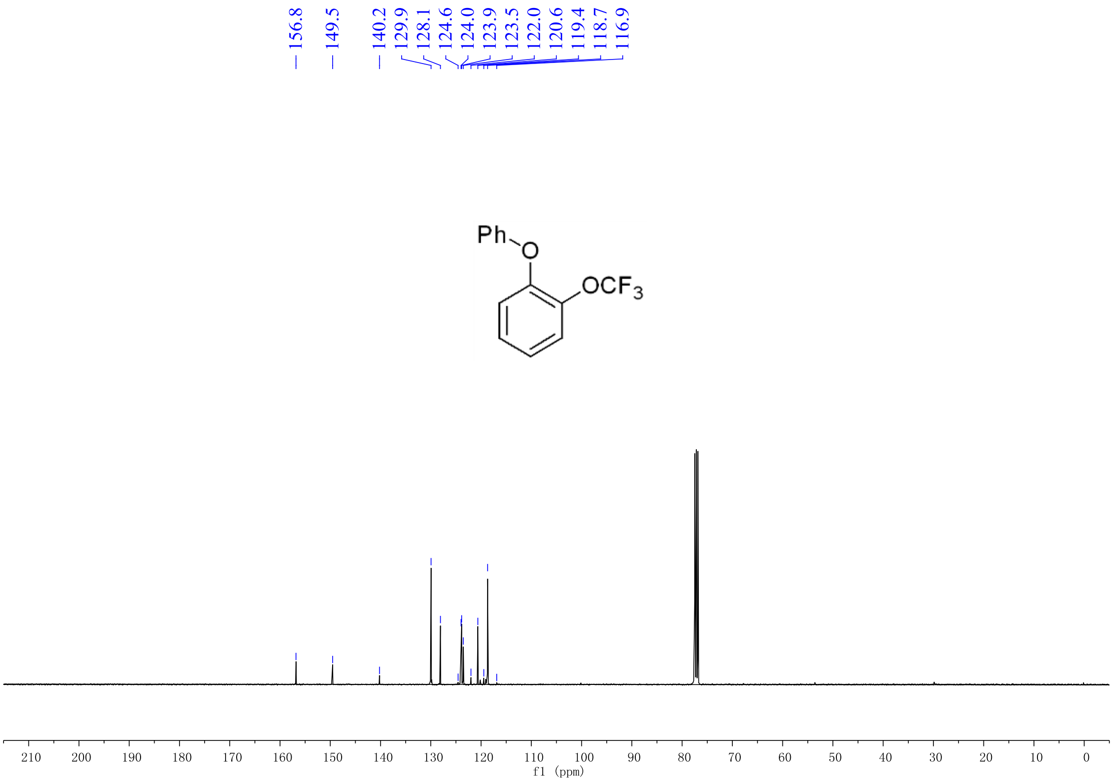

Supplementary Figure 81. ^{13}C NMR spectrum (101 MHz, $CDCl_3$) of ***iso*-3v**

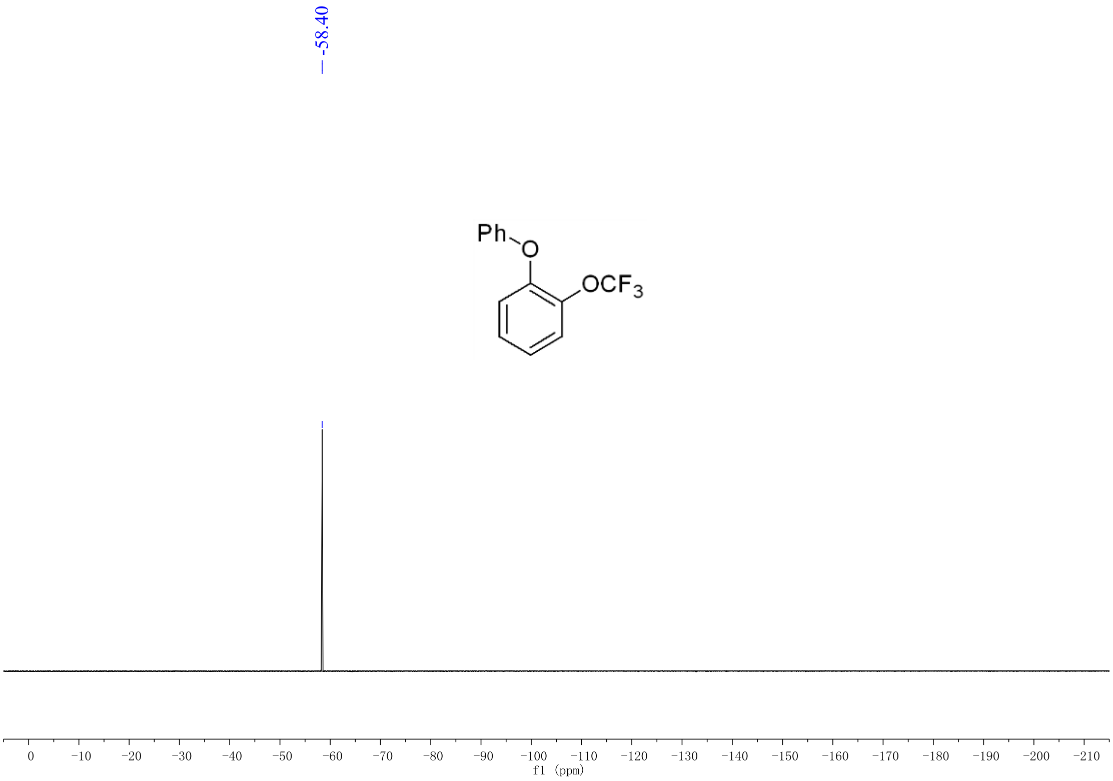

Supplementary Figure 82. ^{19}F NMR spectrum (376 MHz, $CDCl_3$) of ***iso*-3v**

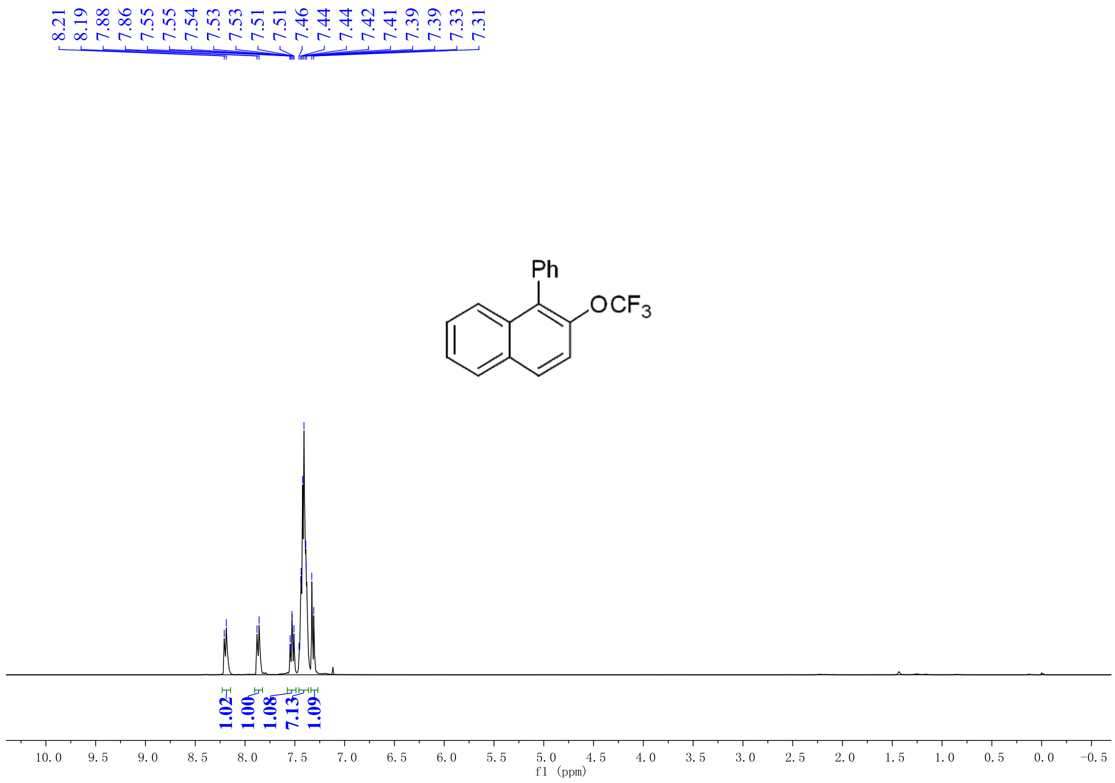

Supplementary Figure 83. ^{1}H NMR spectrum (400 MHz, $CDCl_3$) of **3w**

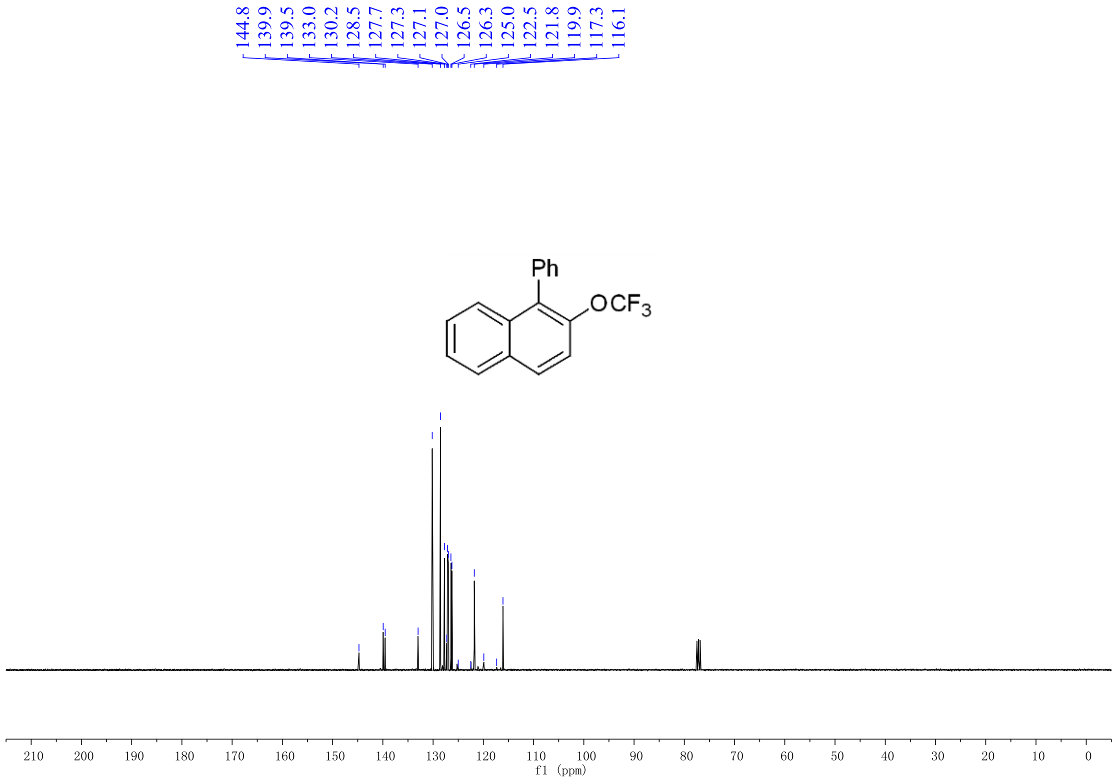

Supplementary Figure 84. ^{13}C NMR spectrum (101 MHz, $CDCl_3$) of **3w**

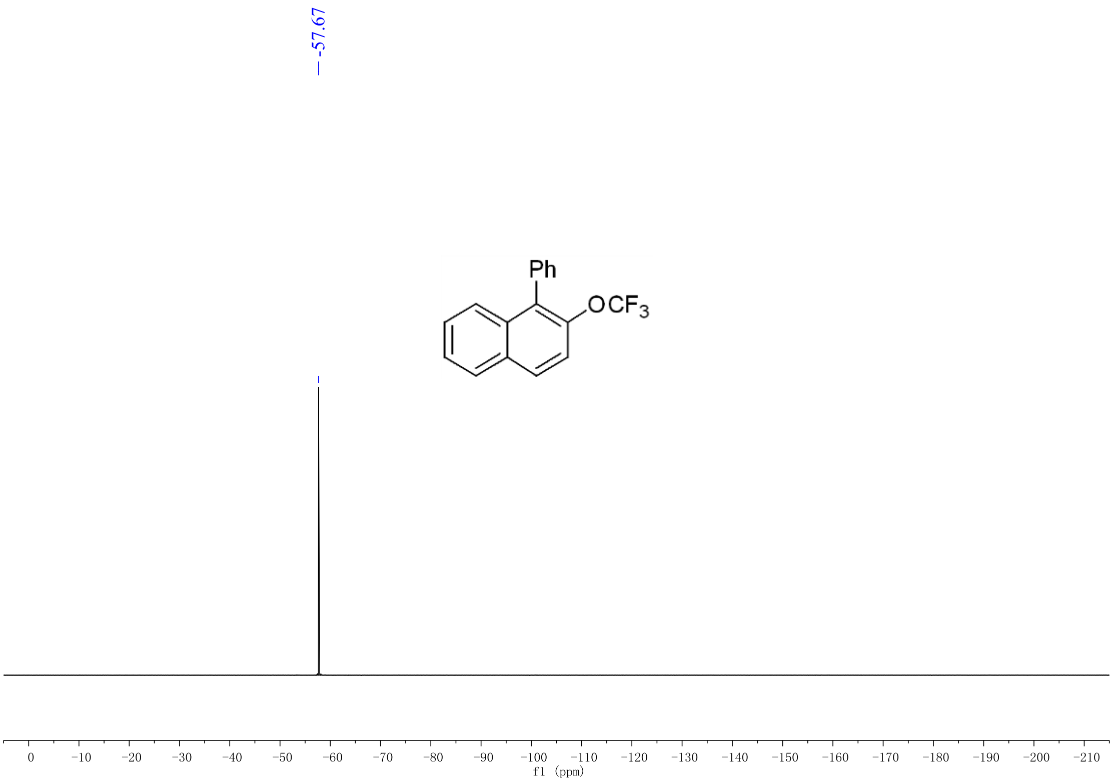

Supplementary Figure 85. ^{19}F NMR spectrum (376 MHz, $CDCl_3$) of **3w**

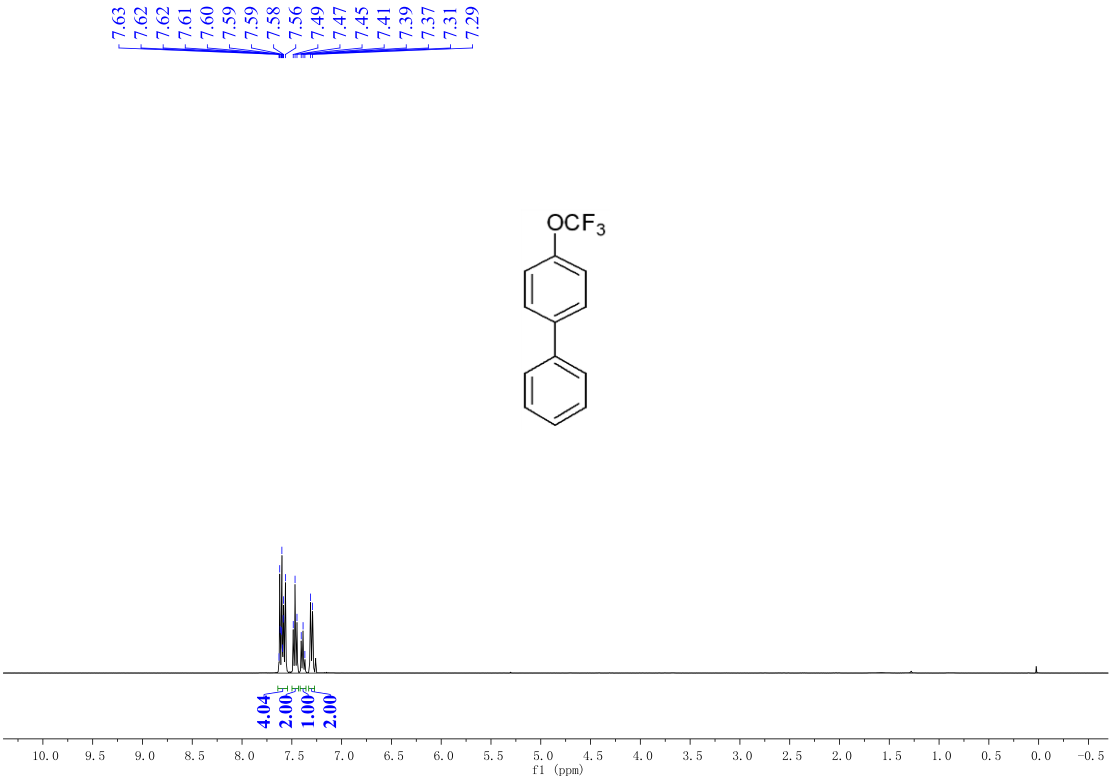

Supplementary Figure 86. ^{1}H NMR spectrum (400 MHz, $CDCl_3$) of **3x**

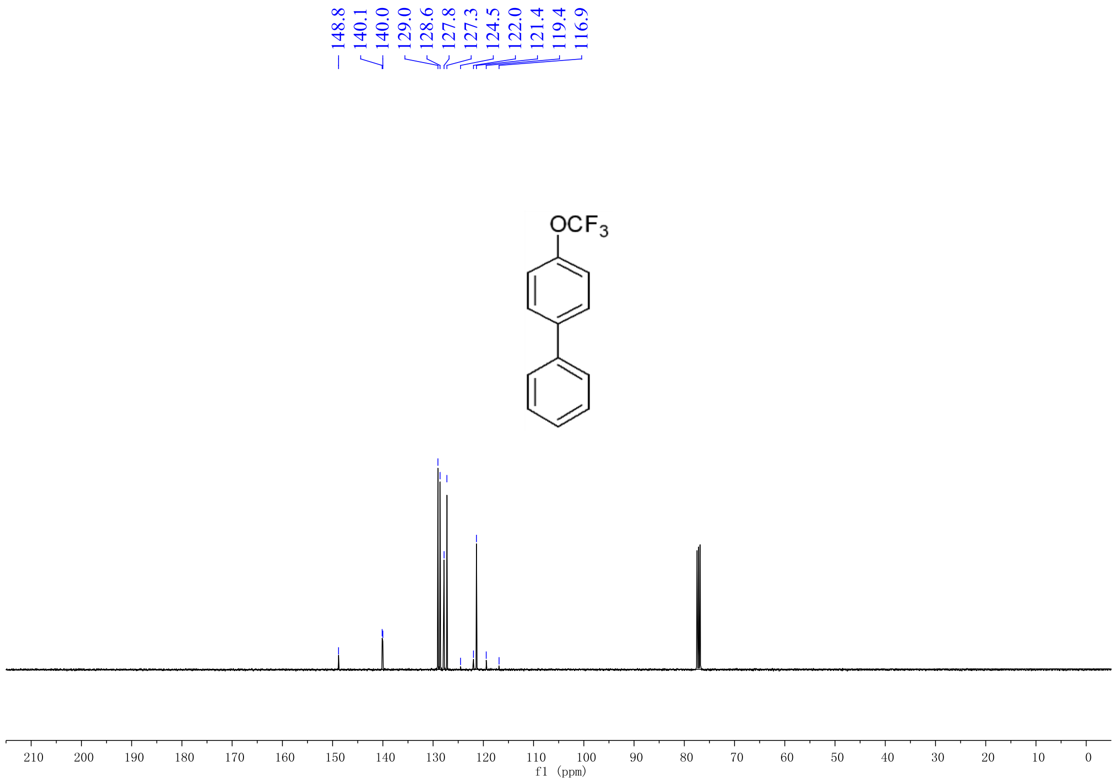

Supplementary Figure 87. ^{13}C NMR spectrum (101 MHz, $CDCl_3$) of **3x**

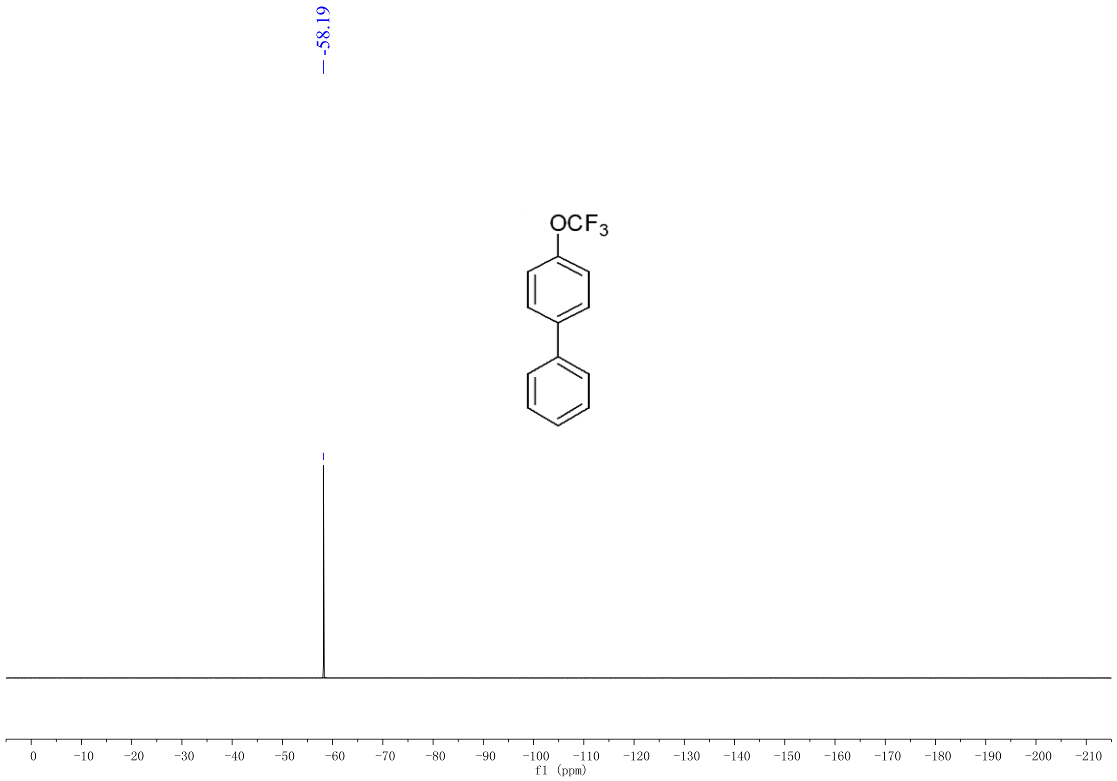

Supplementary Figure 88. ^{19}F NMR spectrum (376 MHz, $CDCl_3$) of **3x**

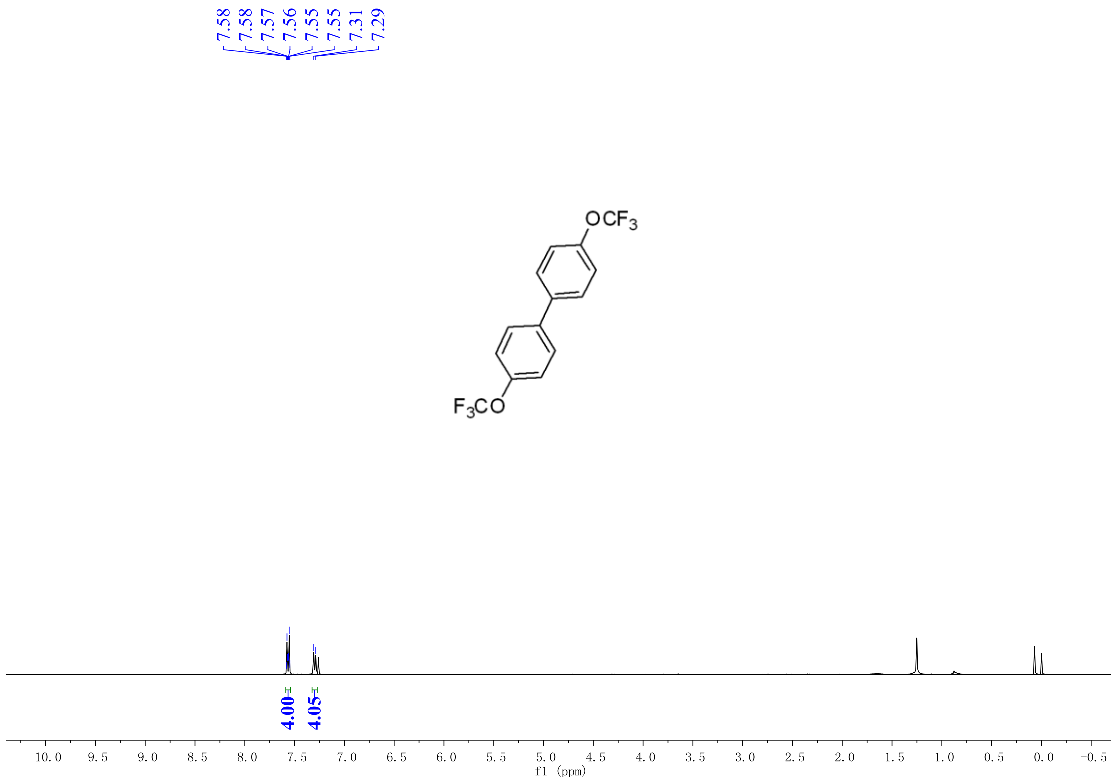

Supplementary Figure 89. ^{1}H NMR spectrum (400 MHz, $CDCl_3$) of **3x'**

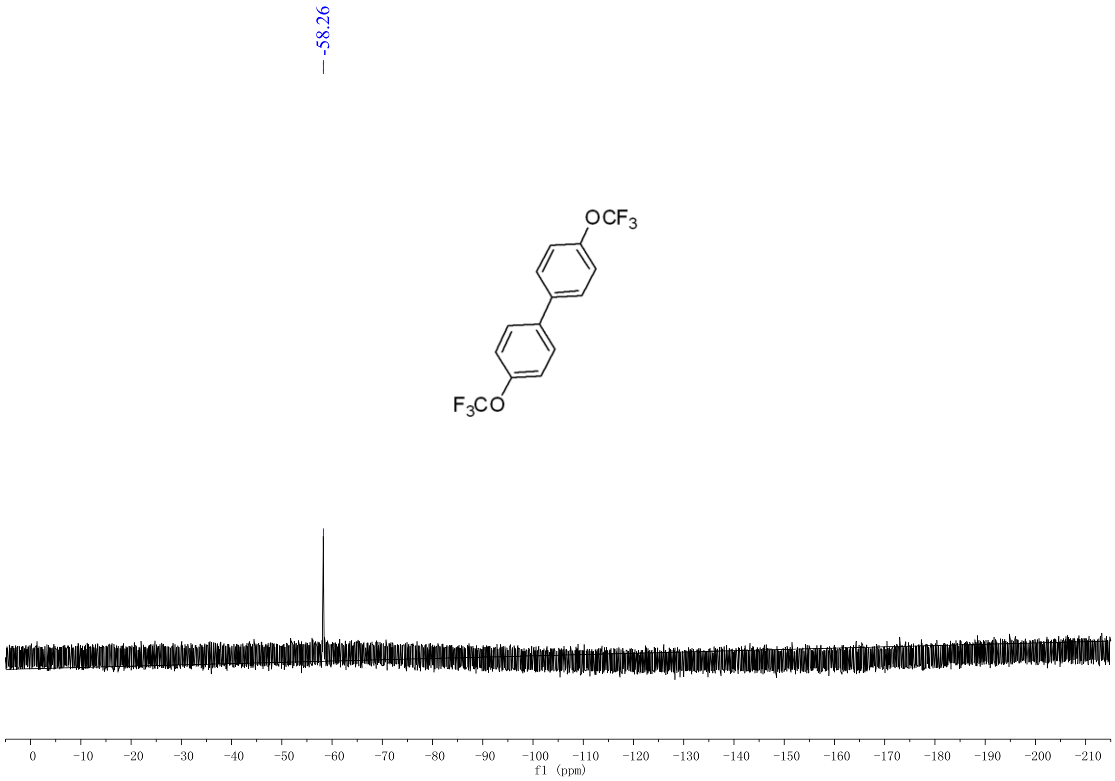

Supplementary Figure 90. ^{19}F NMR spectrum (376 MHz, $CDCl_3$) of **3x'**

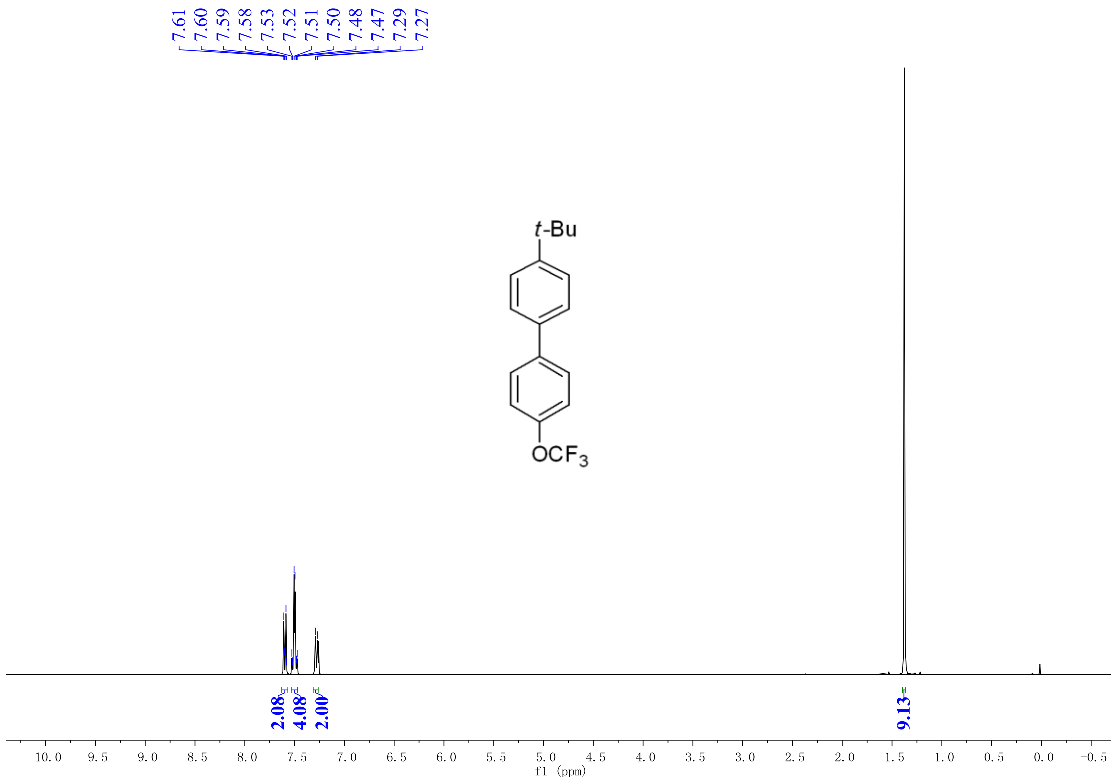

Supplementary Figure 91. ^{1}H NMR spectrum (400 MHz, $CDCl_3$) of **3y**

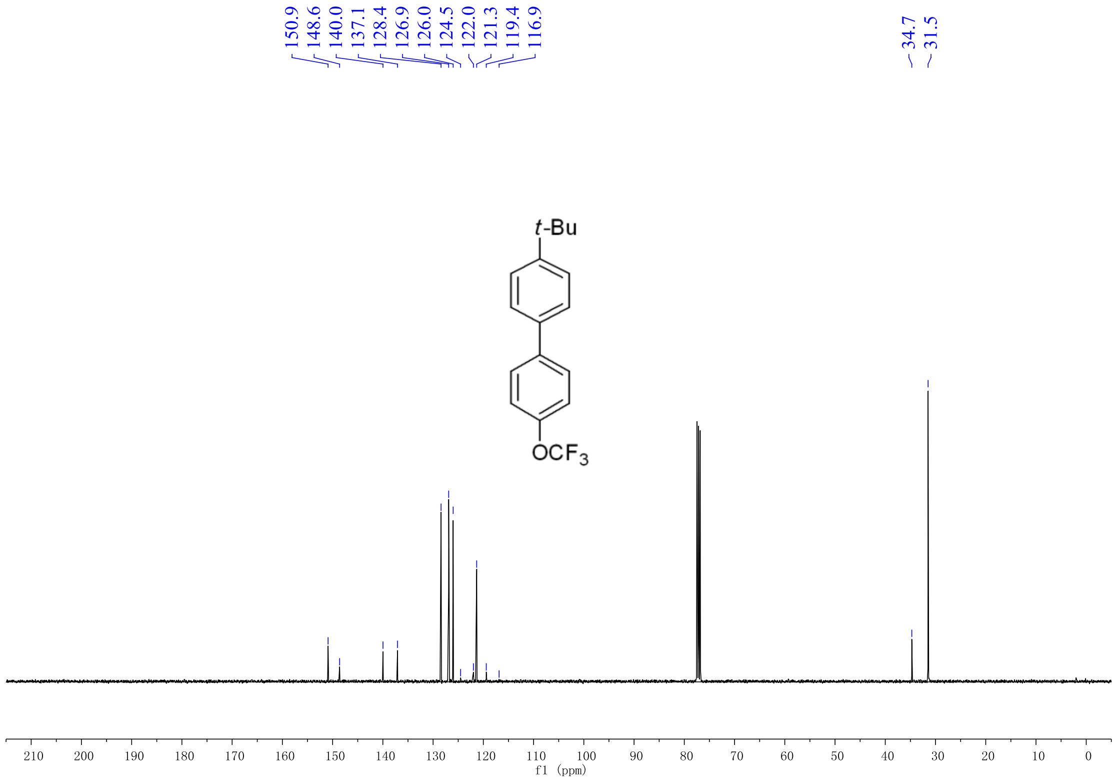

Supplementary Figure 92. ^{13}C NMR spectrum (101 MHz, $CDCl_3$) of **3y**

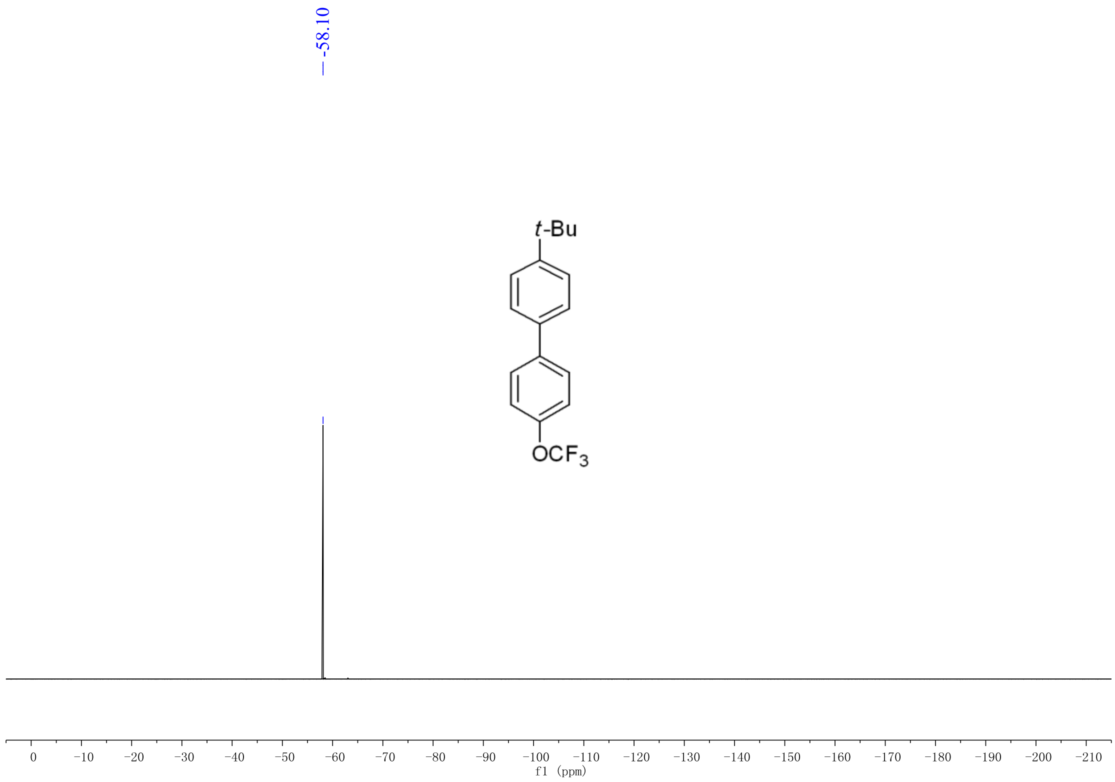

Supplementary Figure 93. ^{19}F NMR spectrum (376 MHz, $CDCl_3$) of **3y**

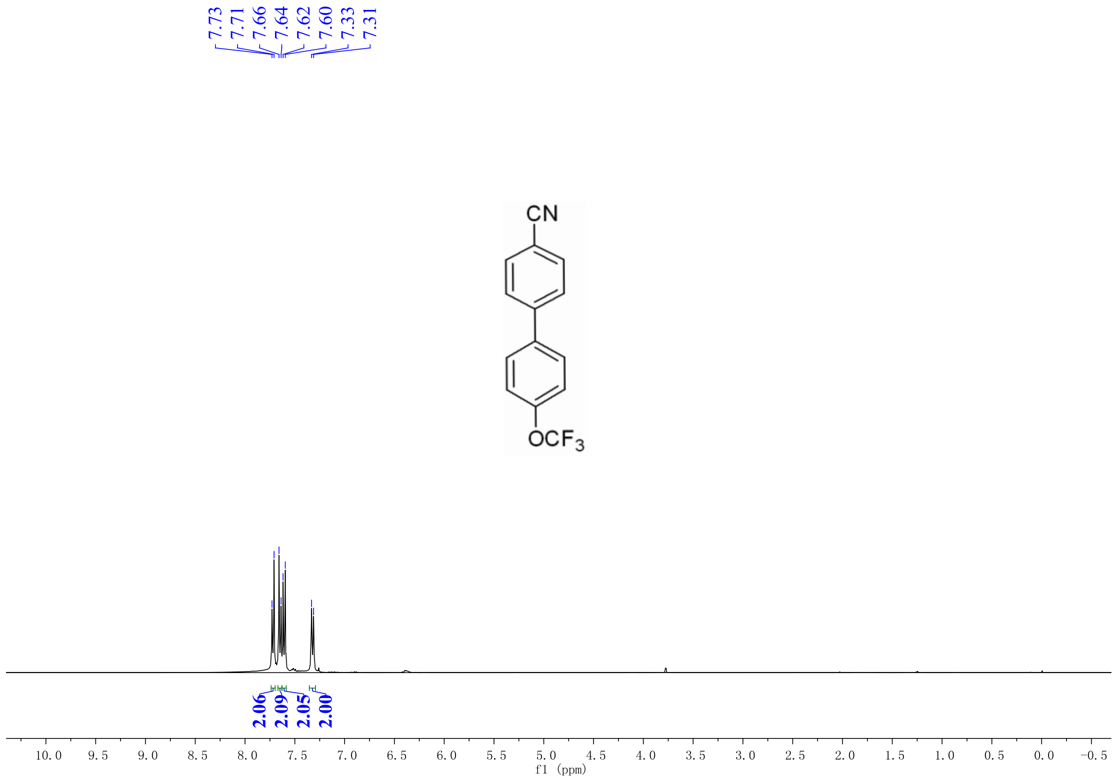

Supplementary Figure 94. ^{1}H NMR spectrum (400 MHz, $CDCl_3$) of **3z**

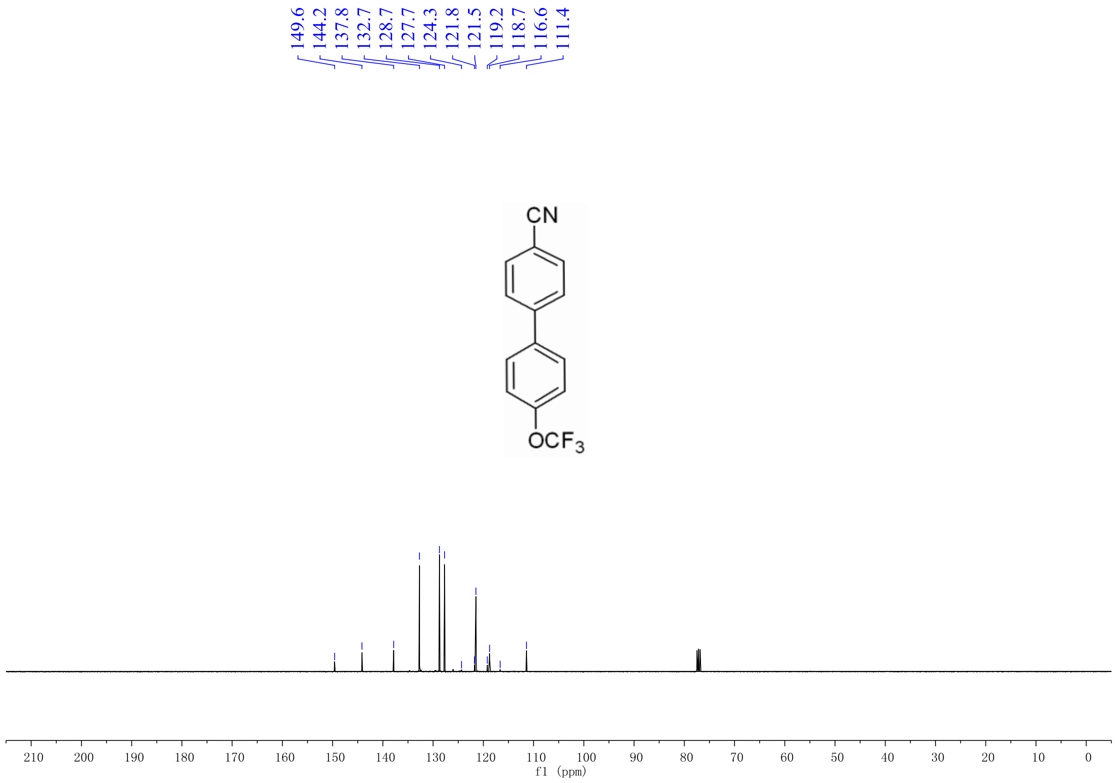

Supplementary Figure 95. ^{13}C NMR spectrum (101 MHz, $CDCl_3$) of **3z**

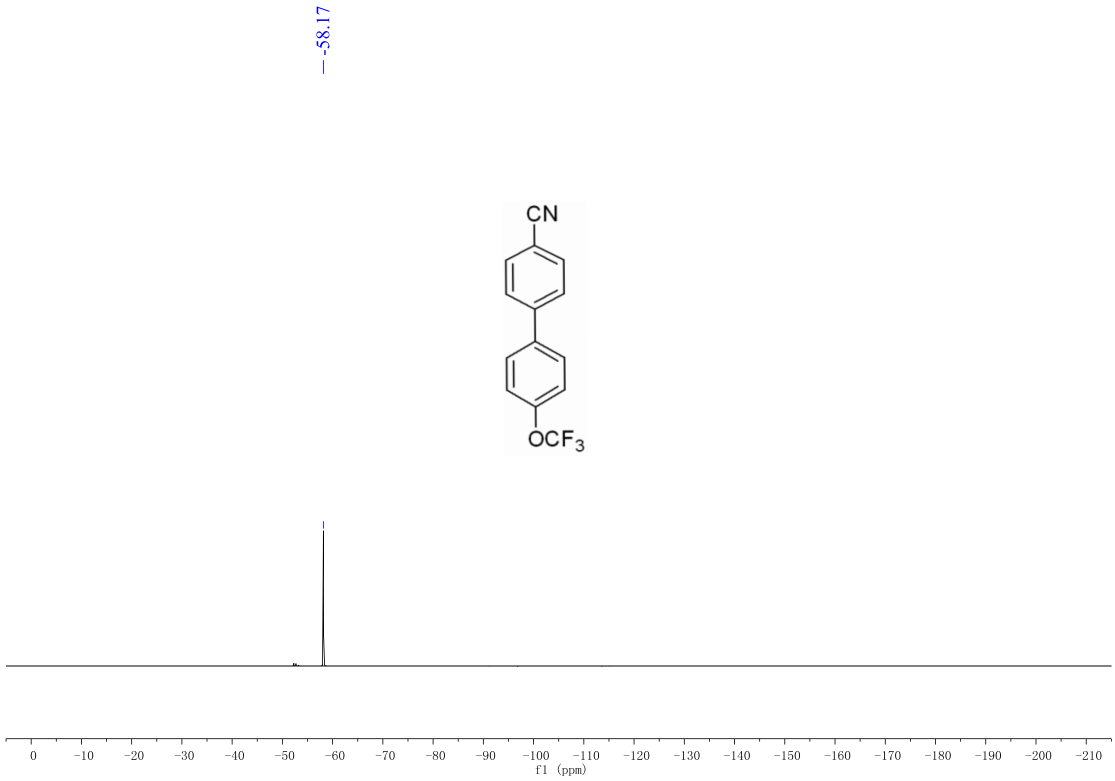

Supplementary Figure 96. ^{19}F NMR spectrum (376 MHz, $CDCl_3$) of **3z**

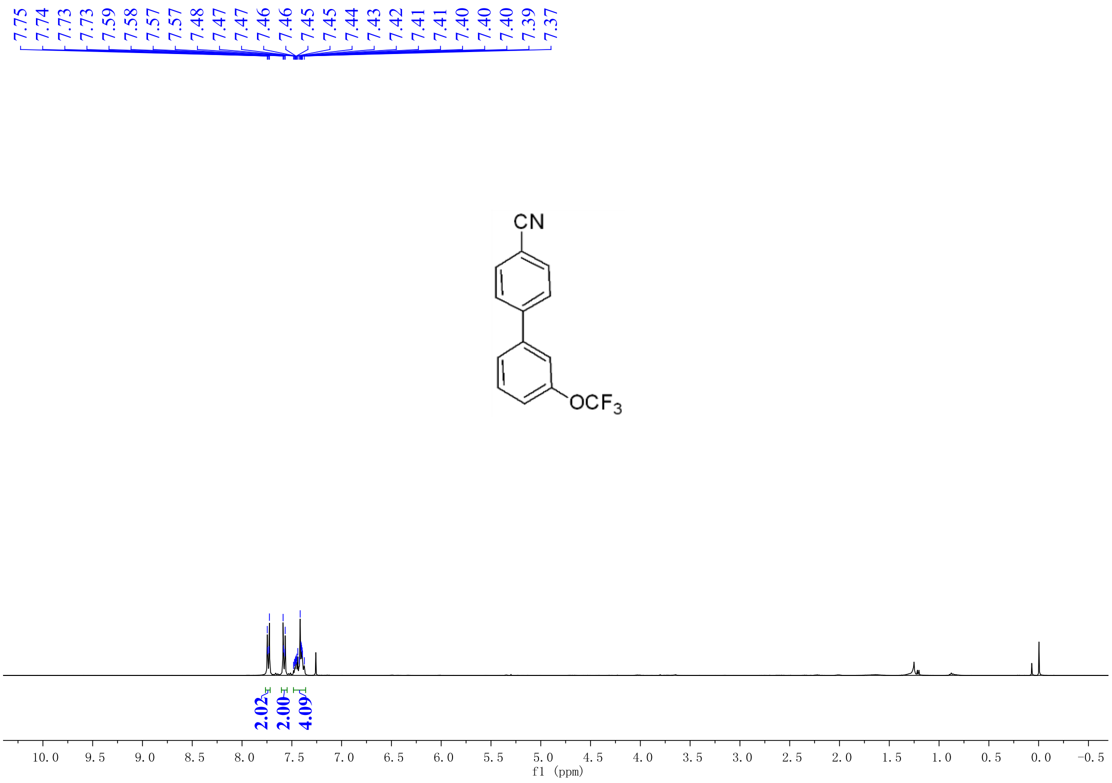

Supplementary Figure 97. ^{1}H NMR spectrum (400 MHz, $CDCl_3$) of ***iso*-3z**

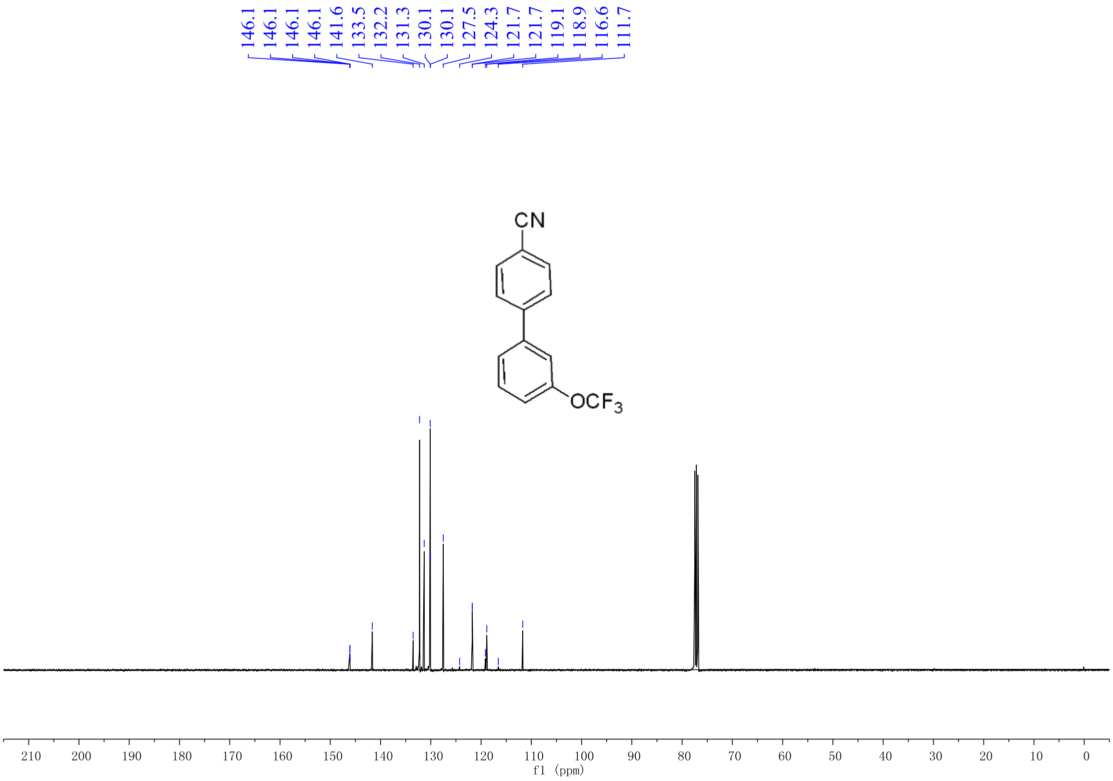

Supplementary Figure 98. ^{13}C NMR spectrum (101 MHz, $CDCl_3$) of ***iso*-3z**

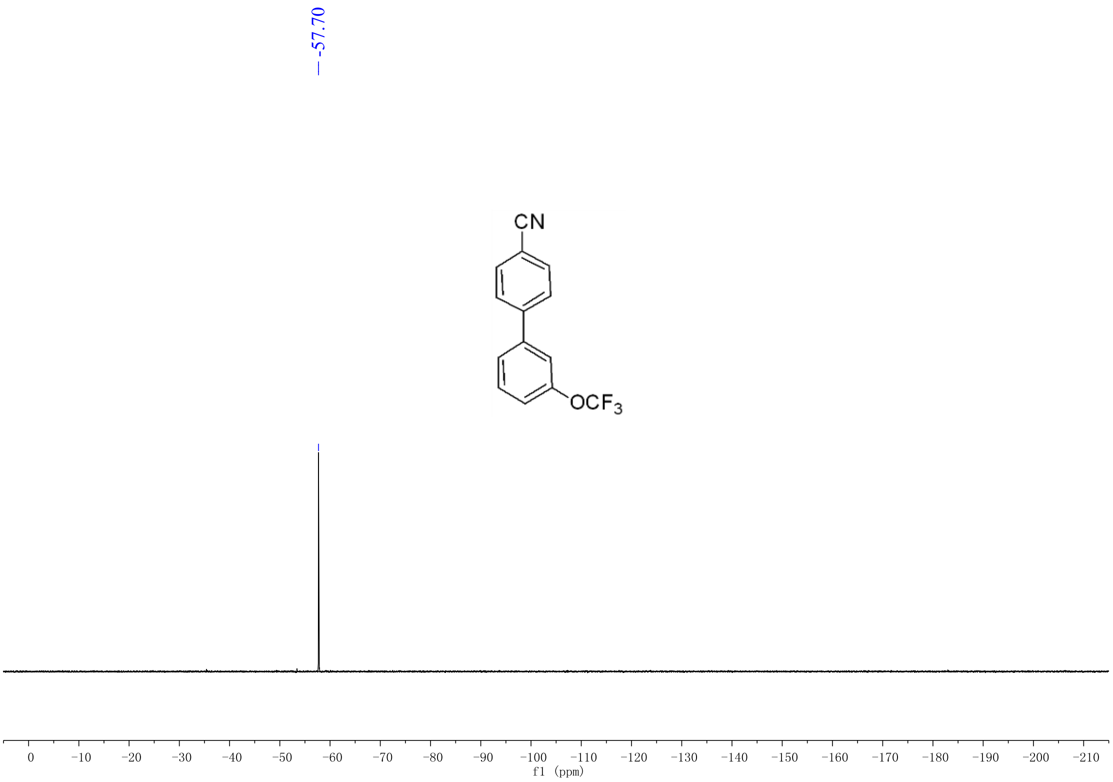

Supplementary Figure 99. ^{19}F NMR spectrum (376 MHz, $CDCl_3$) of ***iso*-3z**

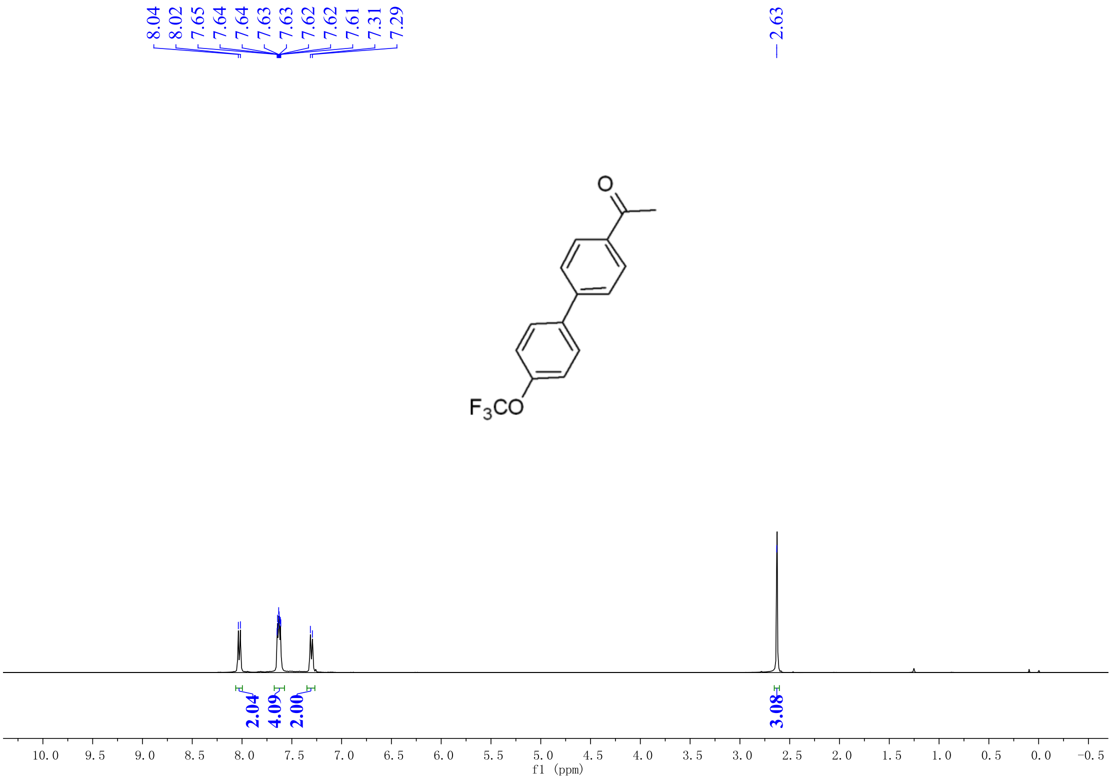

Supplementary Figure 100. ^{1}H NMR spectrum (400 MHz, $CDCl_3$) of **3aa**

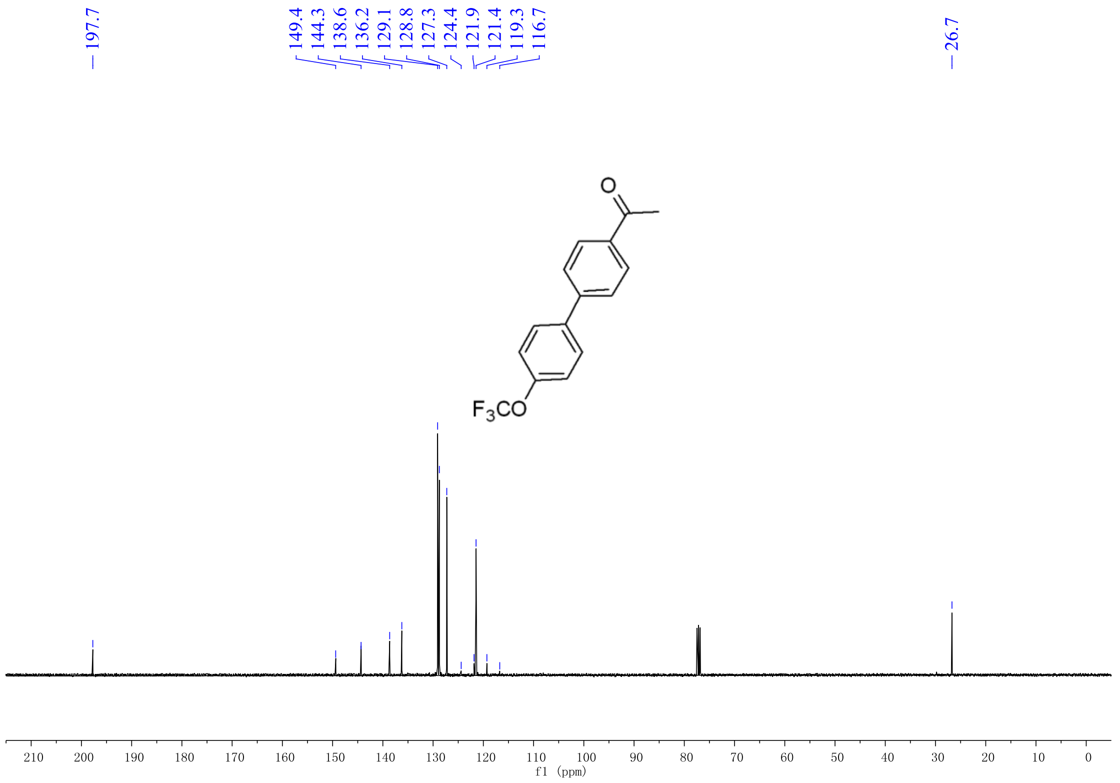

Supplementary Figure 101. ^{13}C NMR spectrum (101 MHz, $CDCl_3$) of **3aa**

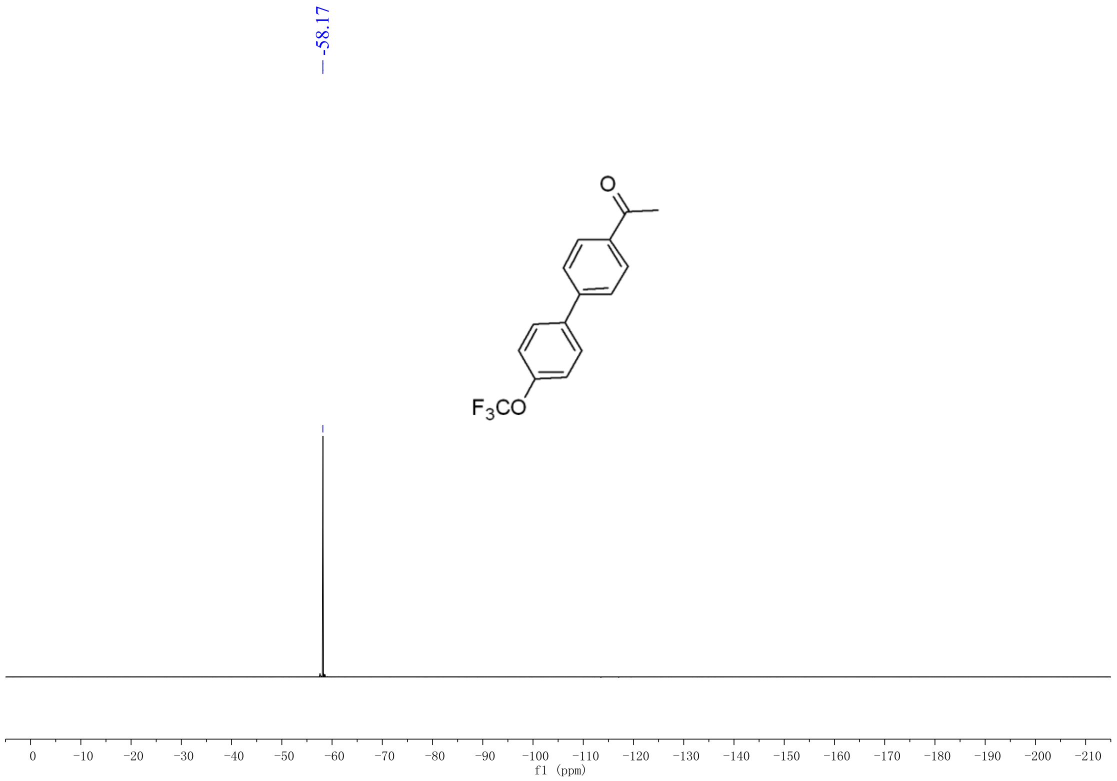

Supplementary Figure 102. ^{19}F NMR spectrum (376 MHz, $CDCl_3$) of **3aa**

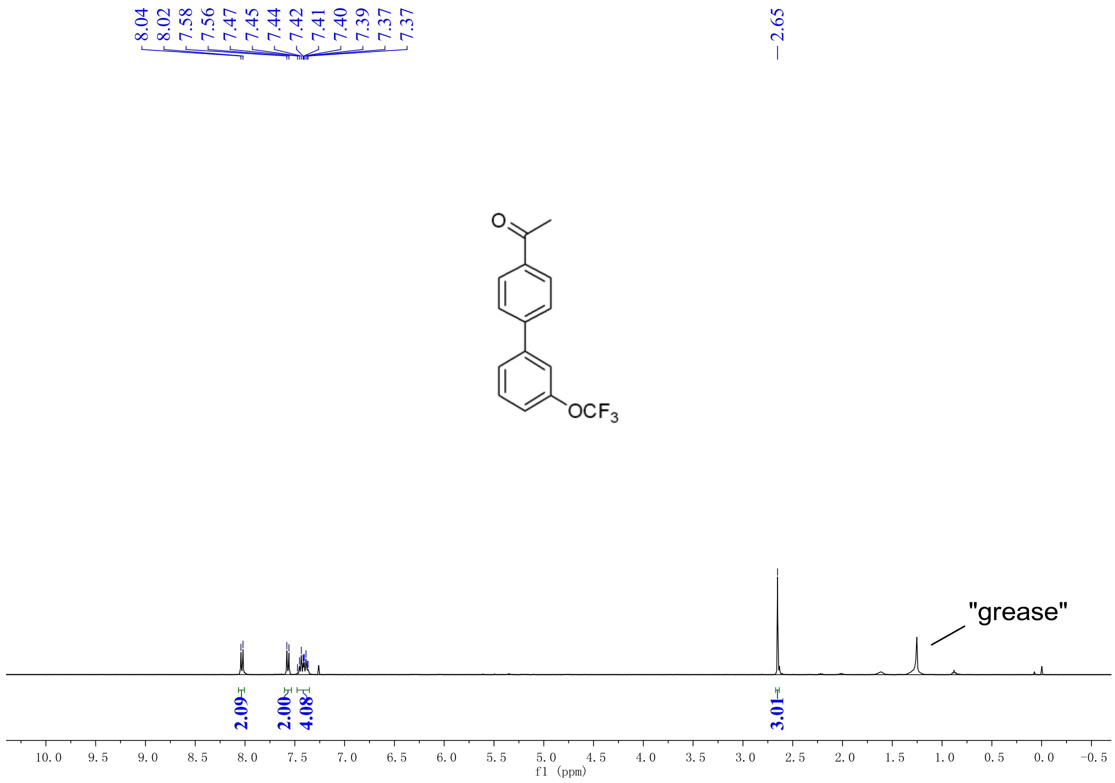

Supplementary Figure 103. ^{1}H NMR spectrum (400 MHz, $CDCl_3$) of ***iso*-3aa**

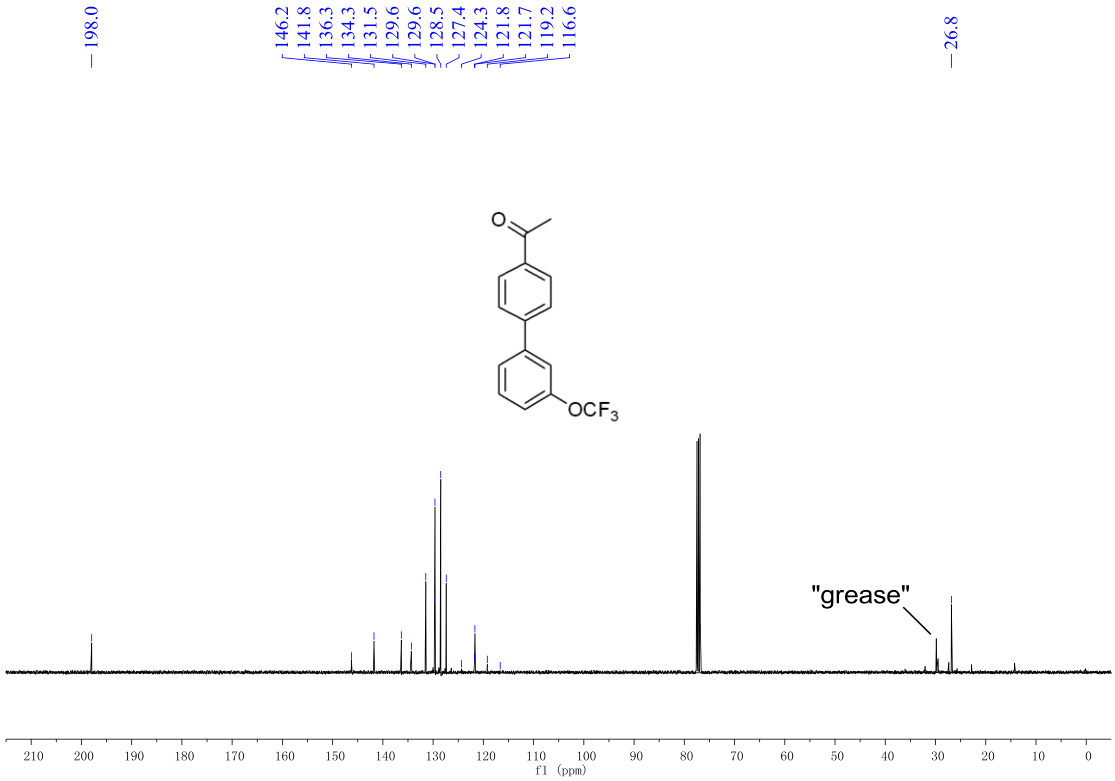

Supplementary Figure 104. ^{13}C NMR spectrum (101 MHz, $CDCl_3$) of ***iso*-3aa**

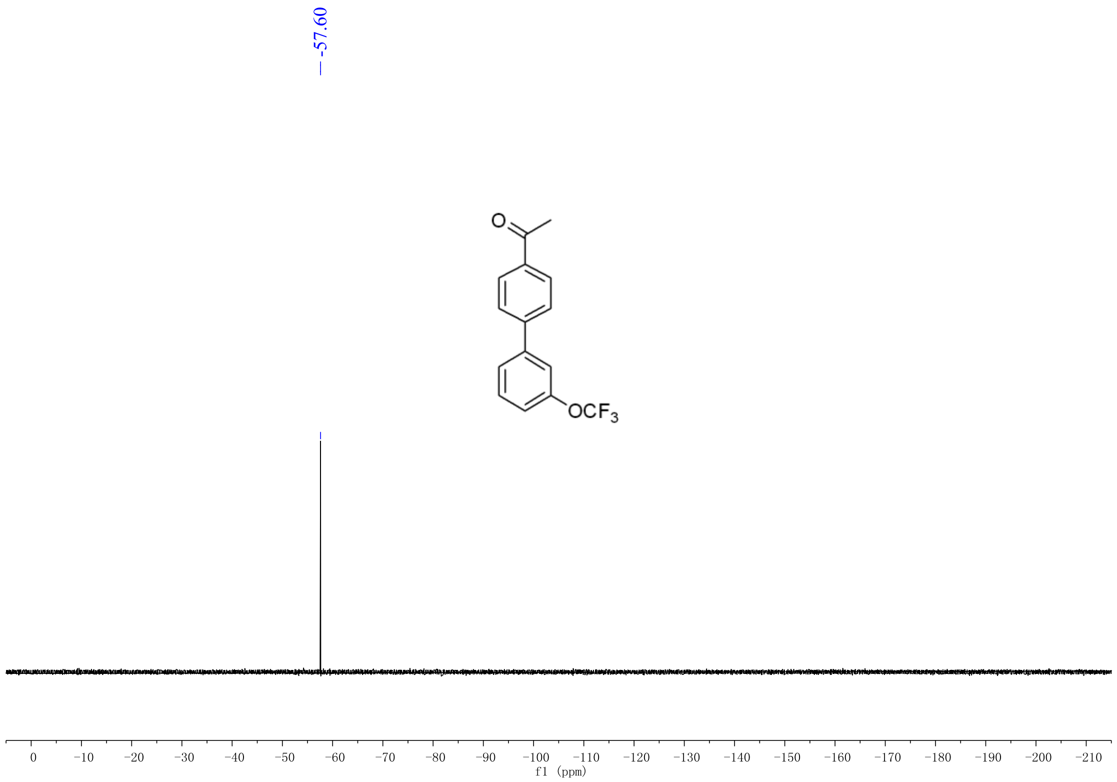

Supplementary Figure 105. ^{19}F NMR spectrum (376 MHz, $CDCl_3$) of ***iso*-3aa**

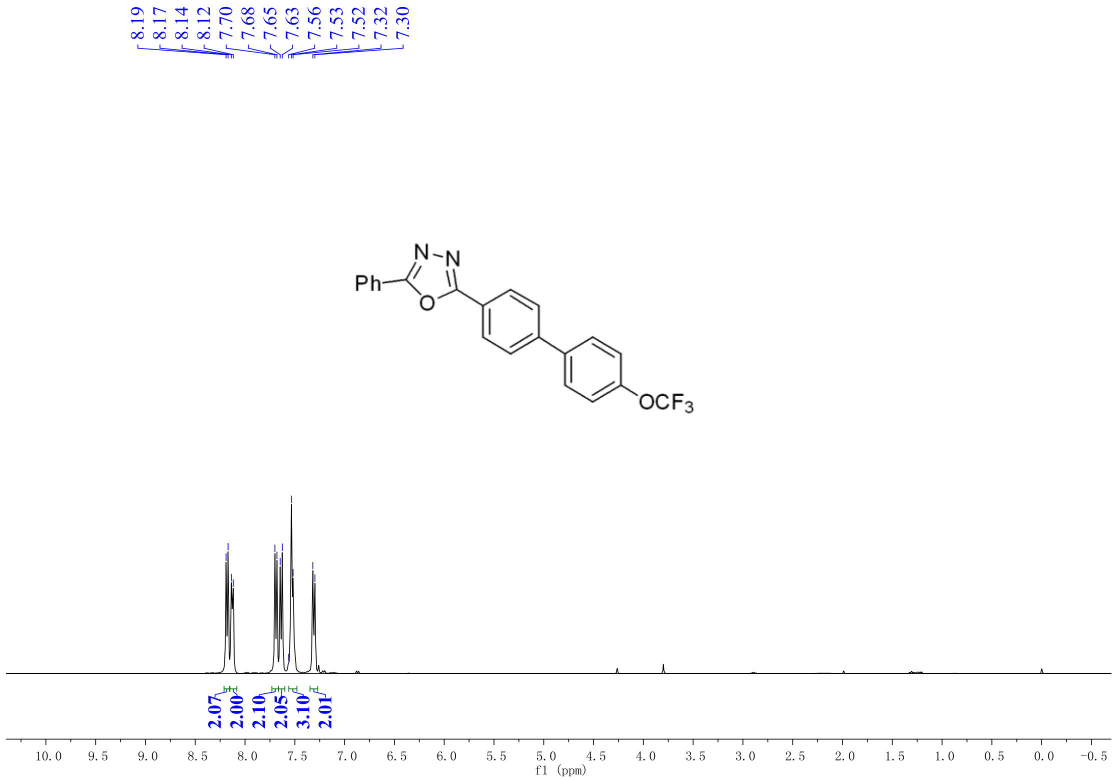

Supplementary Figure 106. ^{1}H NMR spectrum (400 MHz, $CDCl_3$) of **3bb**

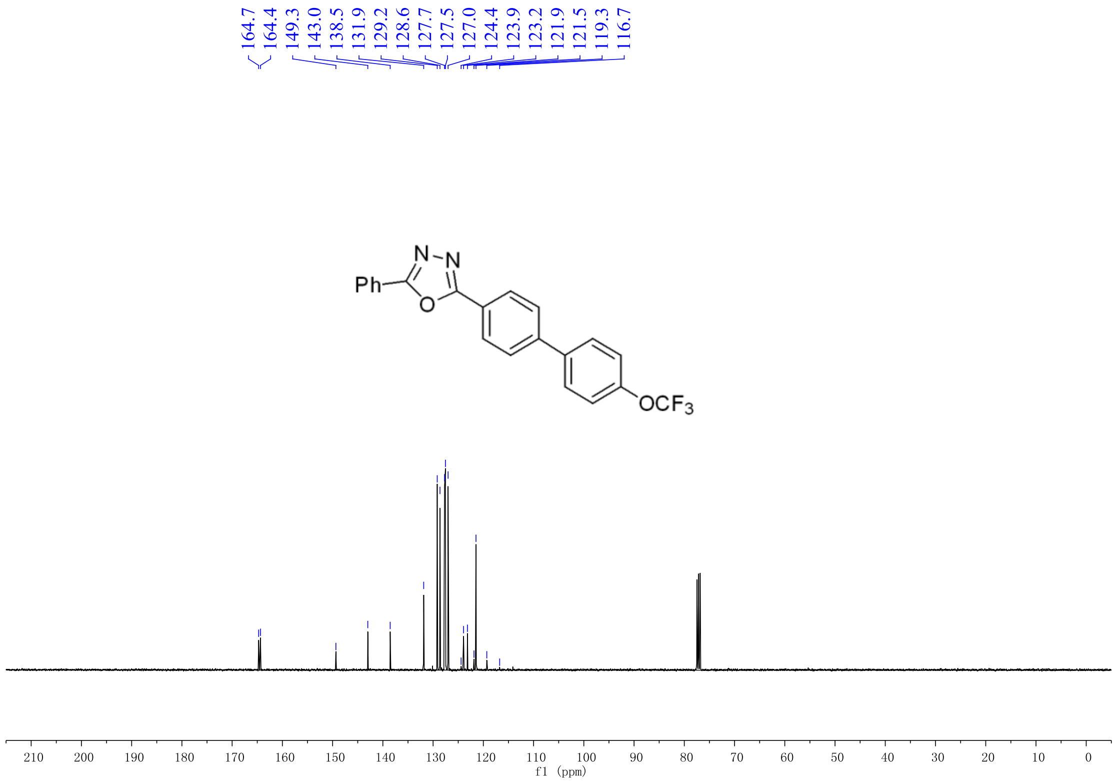

Supplementary Figure 107. ^{13}C NMR spectrum (101 MHz, $CDCl_3$) of **3bb**

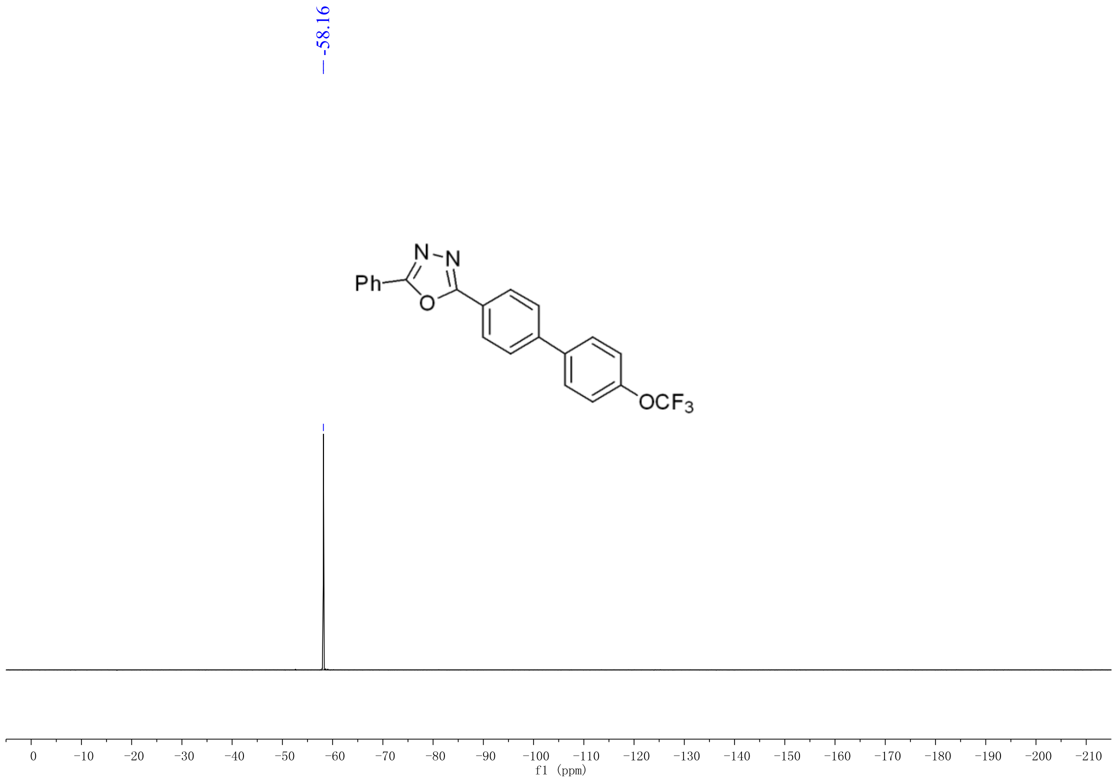

Supplementary Figure 108. ^{19}F NMR spectrum (376 MHz, $CDCl_3$) of **3bb**

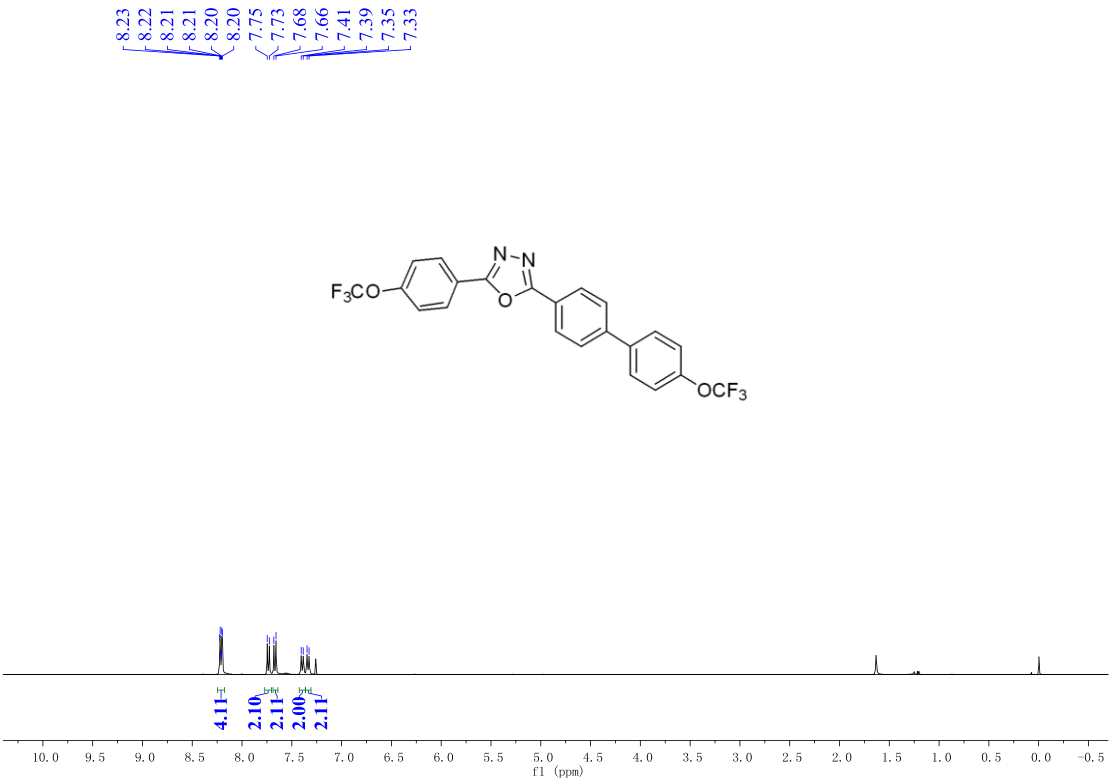

Supplementary Figure 109. ^{1}H NMR spectrum (400 MHz, $CDCl_3$) of **3bb'**

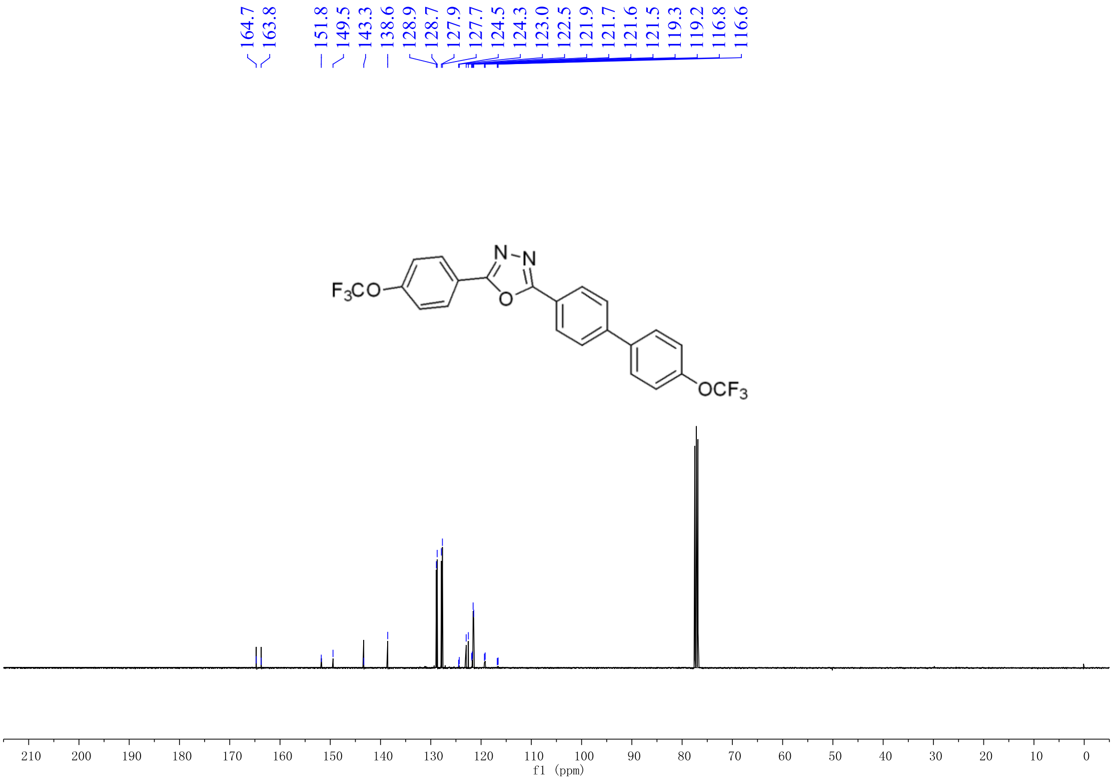

Supplementary Figure 110. ^{13}C NMR spectrum (101 MHz, $CDCl_3$) of **3bb'**

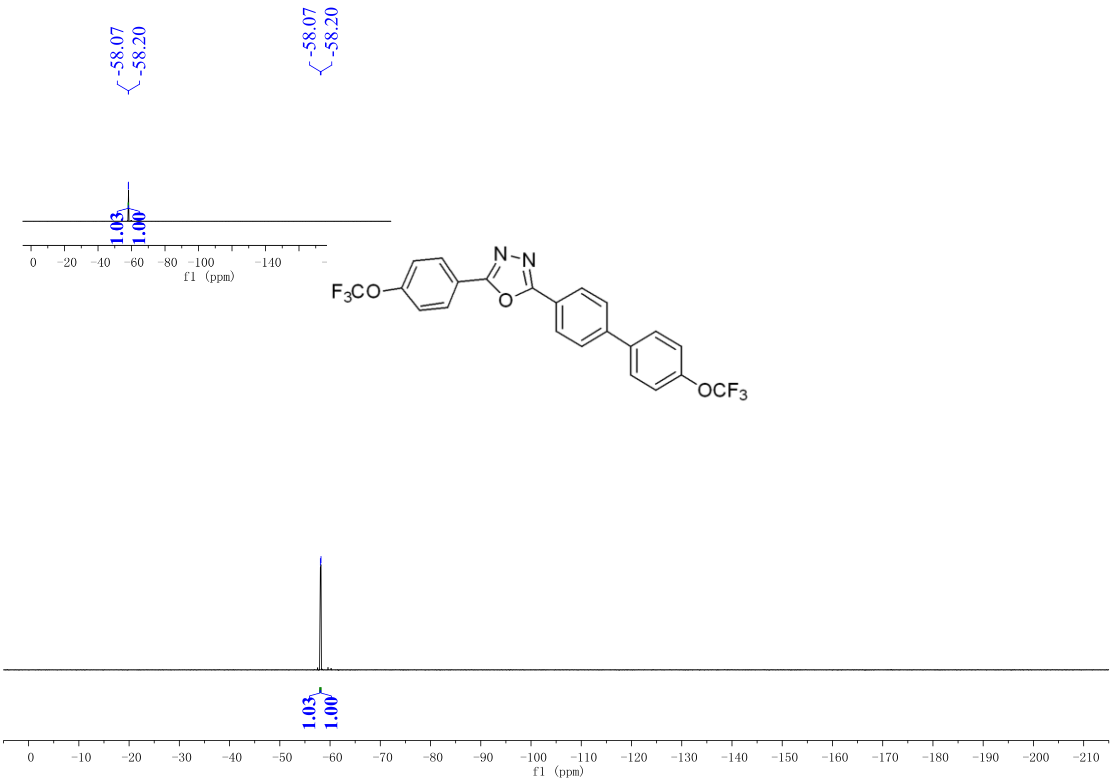

Supplementary Figure 111. ^{19}F NMR spectrum (376 MHz, $CDCl_3$) of **3bb'**

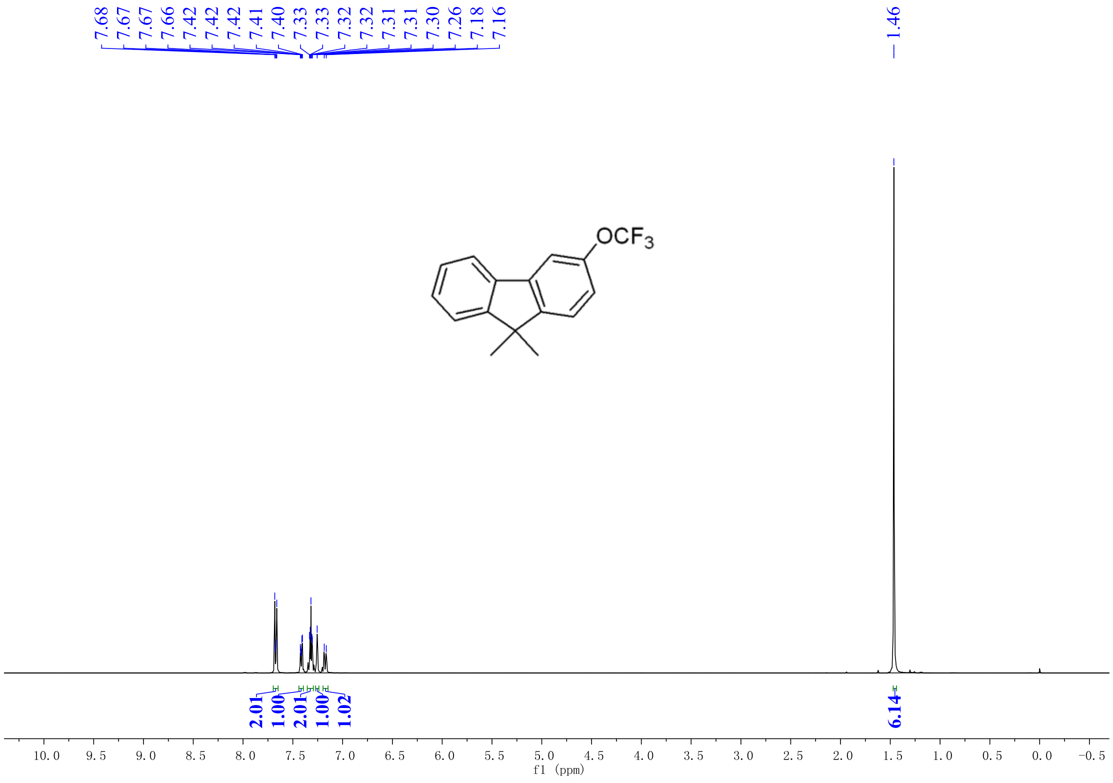

Supplementary Figure 112. ^{1}H NMR spectrum (400 MHz, $CDCl_3$) of **3cc**

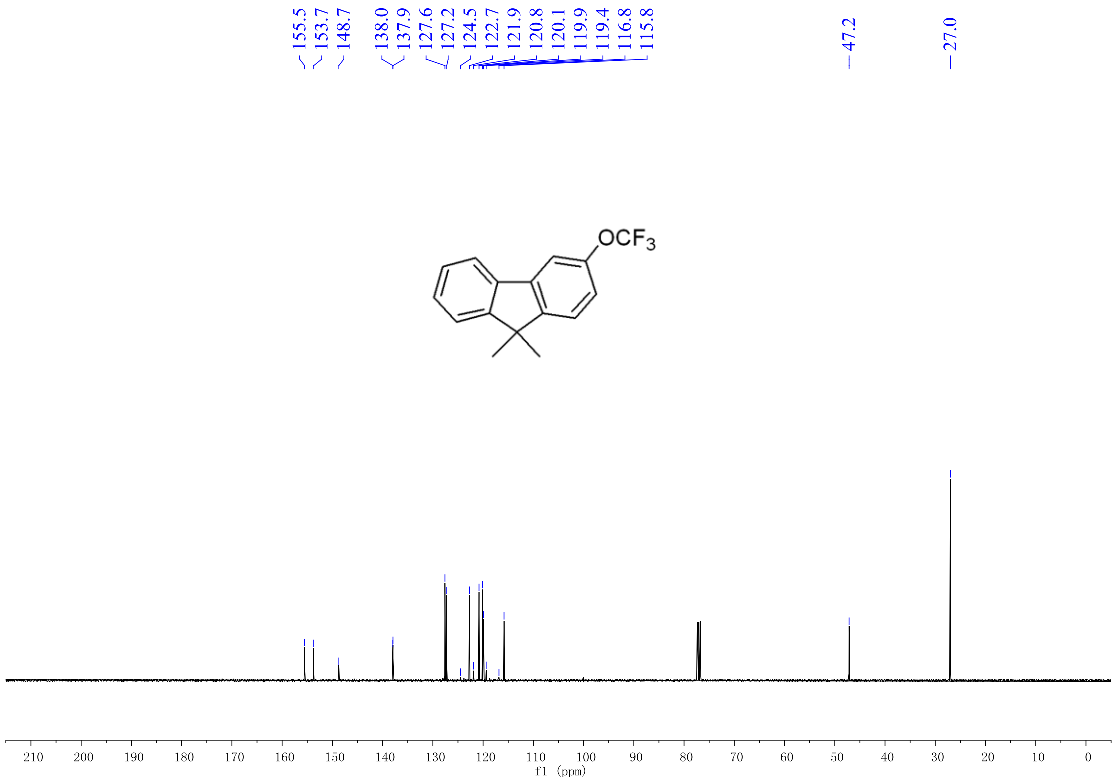

Supplementary Figure 113. ^{13}C NMR spectrum (101 MHz, $CDCl_3$) of **3cc**

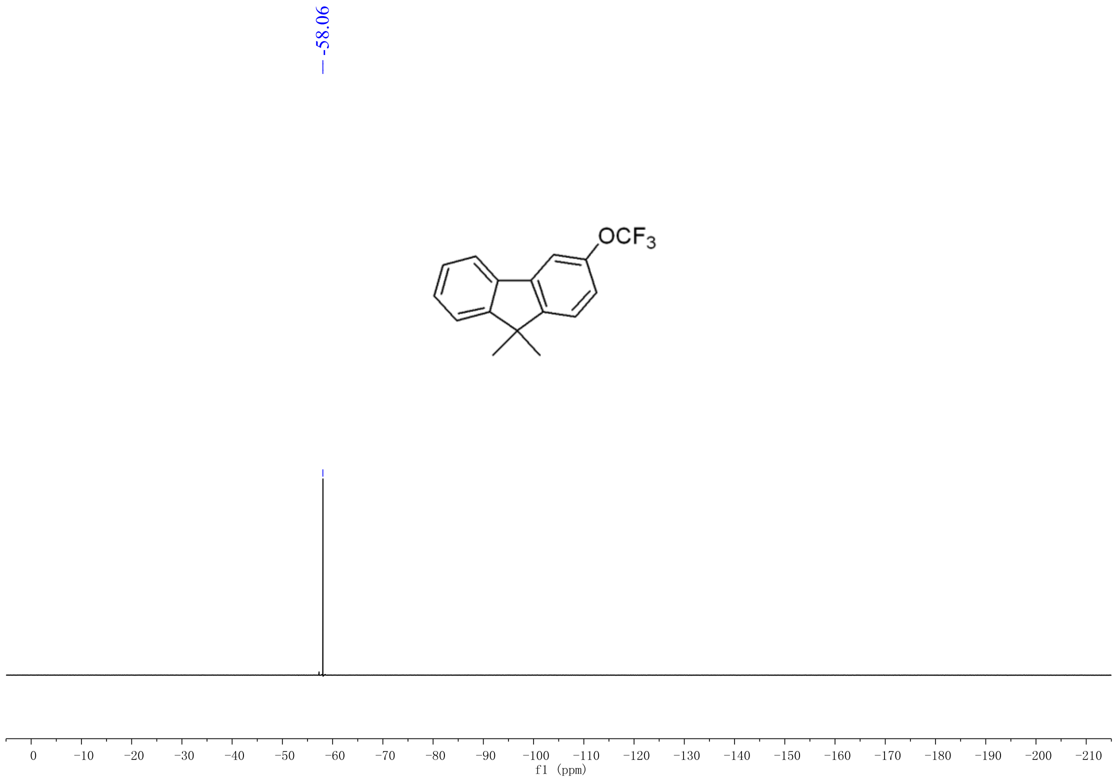

Supplementary Figure 114. ^{19}F NMR spectrum (376 MHz, $CDCl_3$) of **3cc**

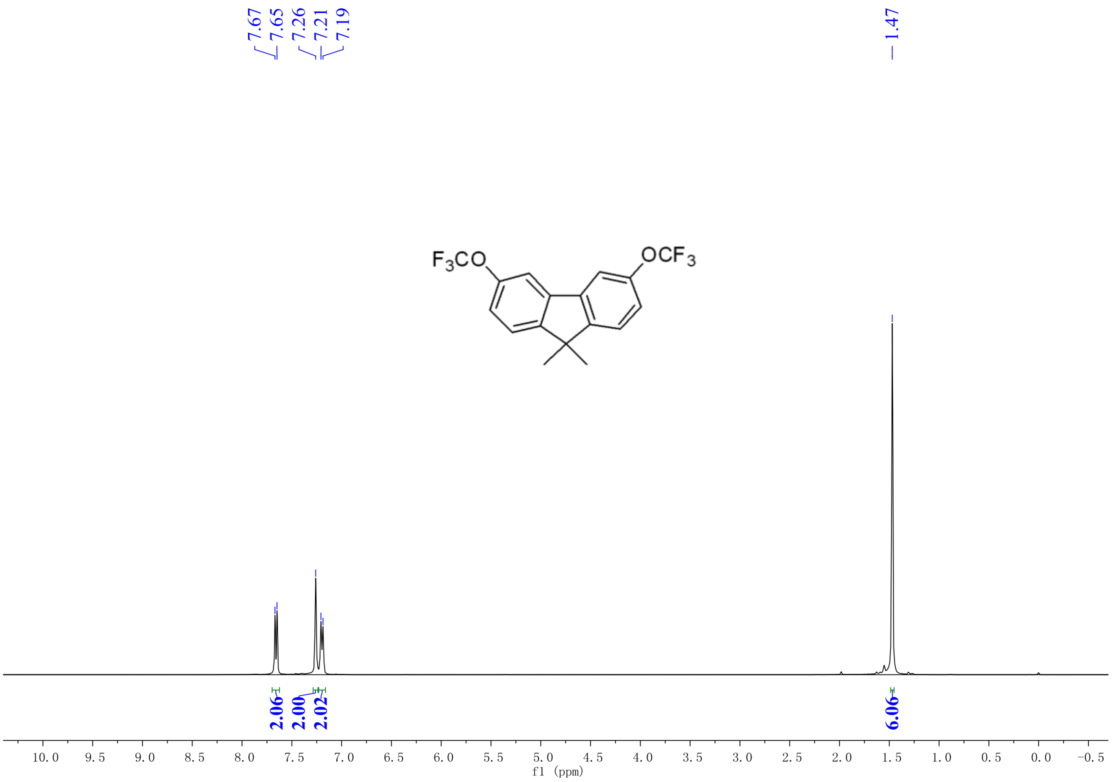

Supplementary Figure 115. ^{1}H NMR spectrum (400 MHz, $CDCl_3$) of **3cc'**

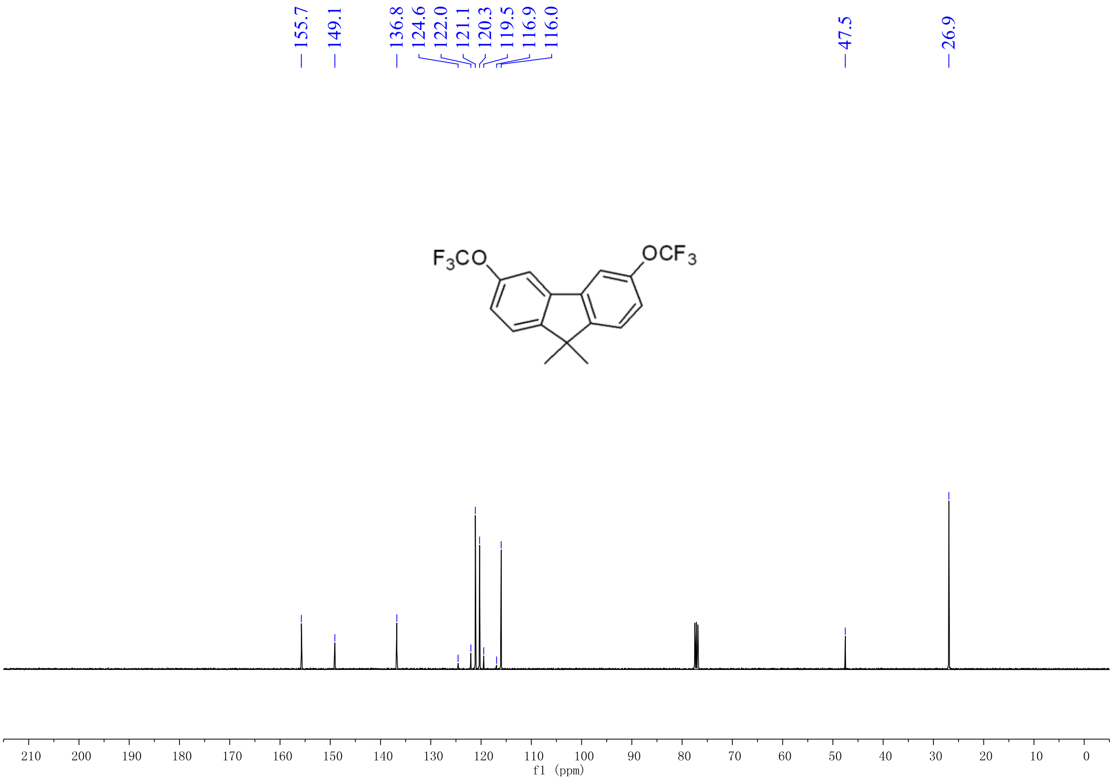

Supplementary Figure 116. ^{13}C NMR spectrum (101 MHz, $CDCl_3$) of **3cc'**

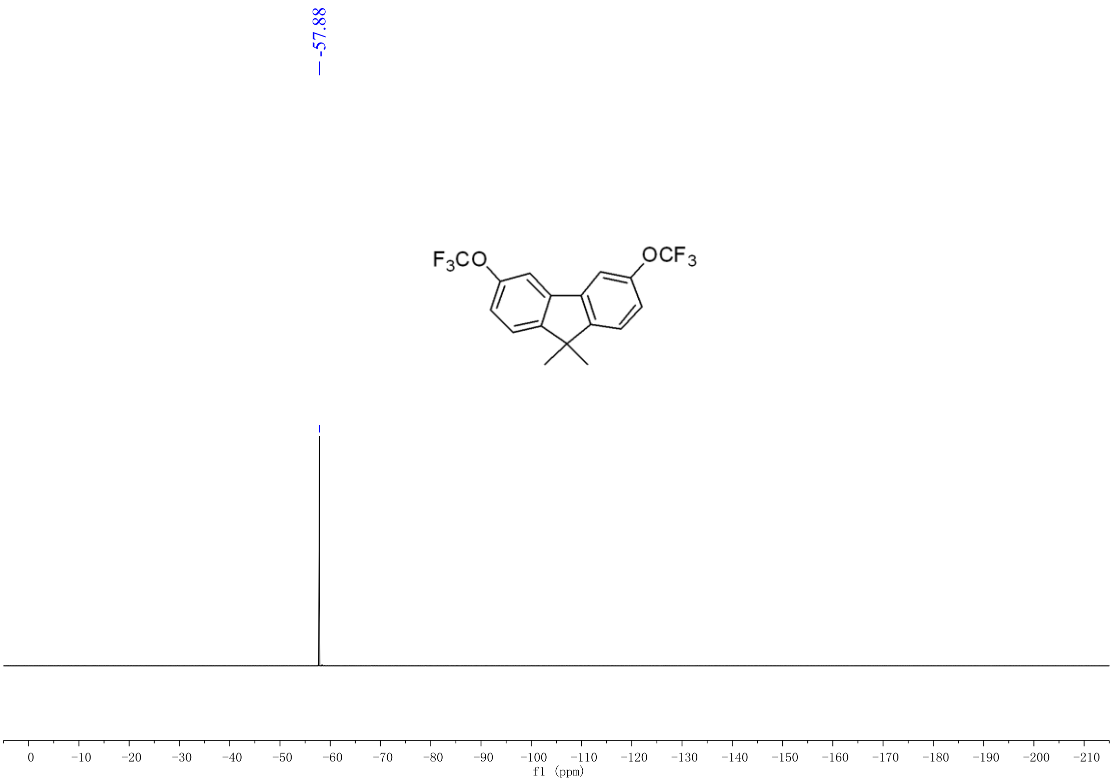

Supplementary Figure 117. ^{19}F NMR spectrum (376 MHz, $CDCl_3$) of **3cc'**

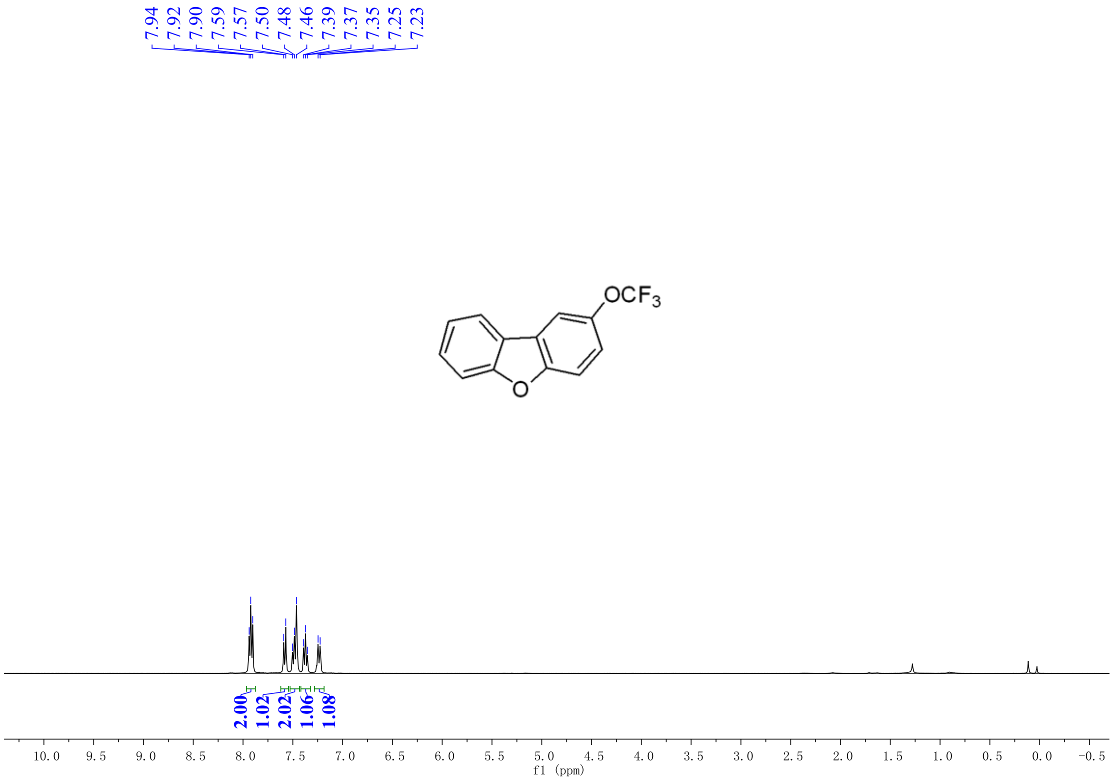

Supplementary Figure 118. ^{1}H NMR spectrum (400 MHz, $CDCl_3$) of **3dd**

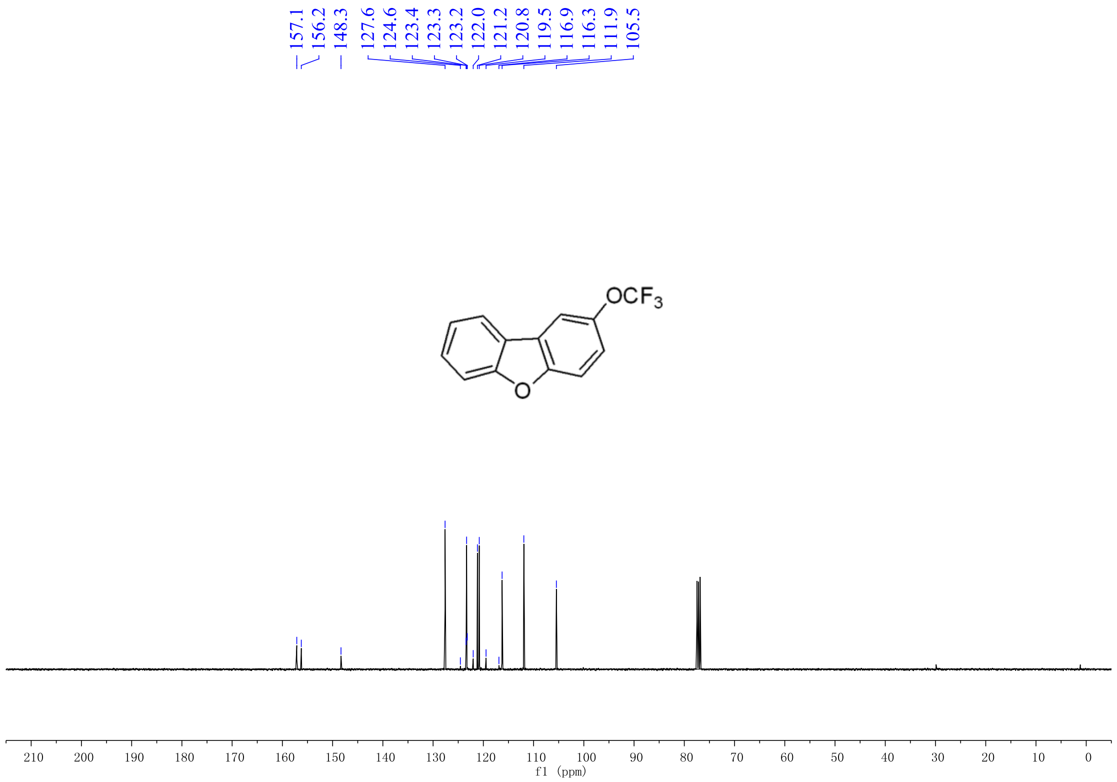

Supplementary Figure 119. ^{13}C NMR spectrum (101 MHz, $CDCl_3$) of **3dd**

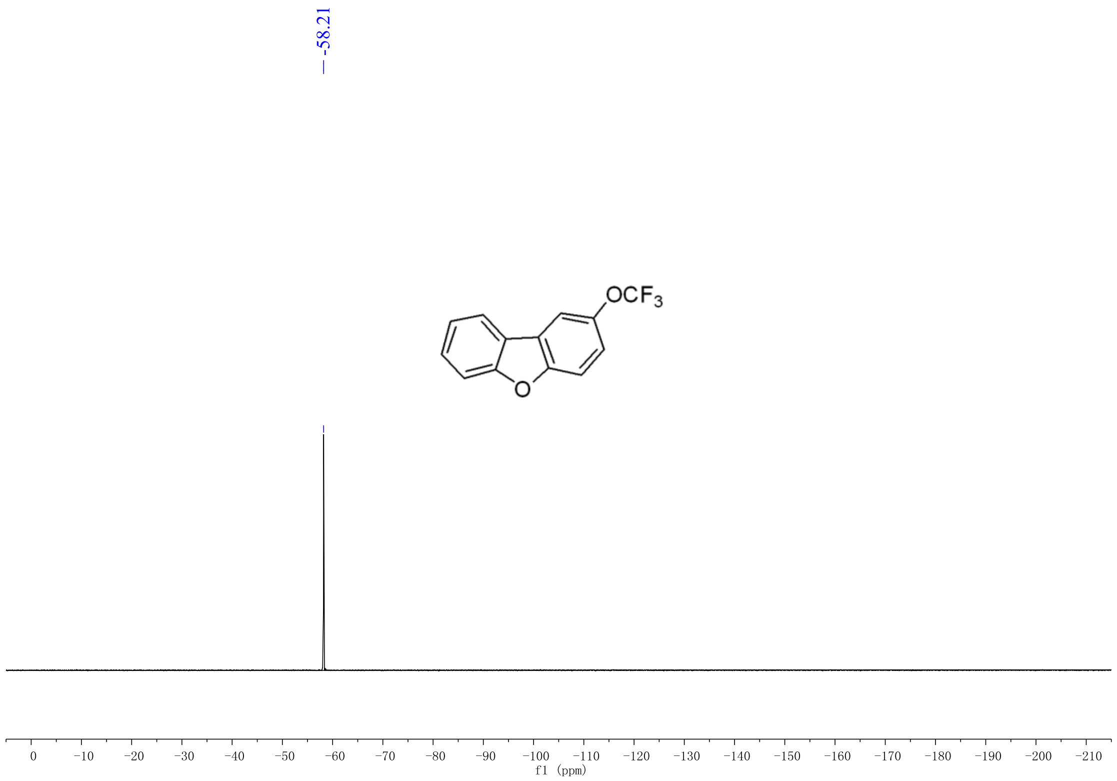

Supplementary Figure 120. ^{19}F NMR spectrum (376 MHz, $CDCl_3$) of **3dd**

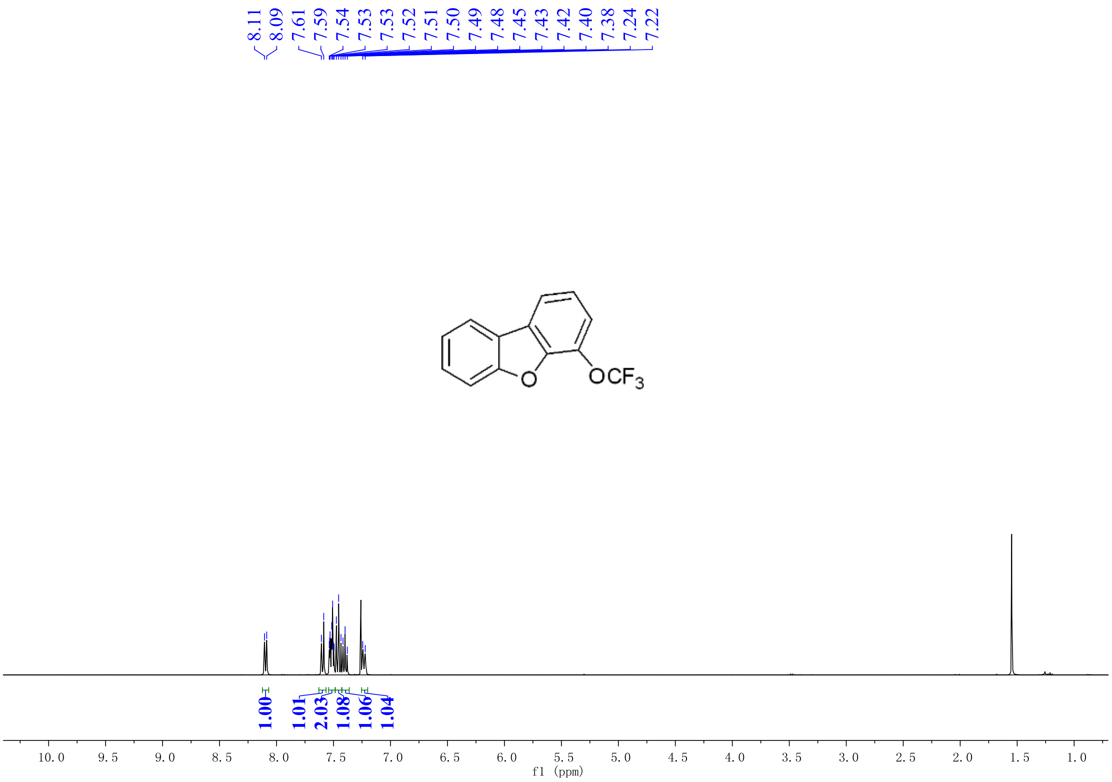

Supplementary Figure 121. ^{1}H NMR spectrum (400 MHz, $CDCl_3$) of ***iso*-3dd**

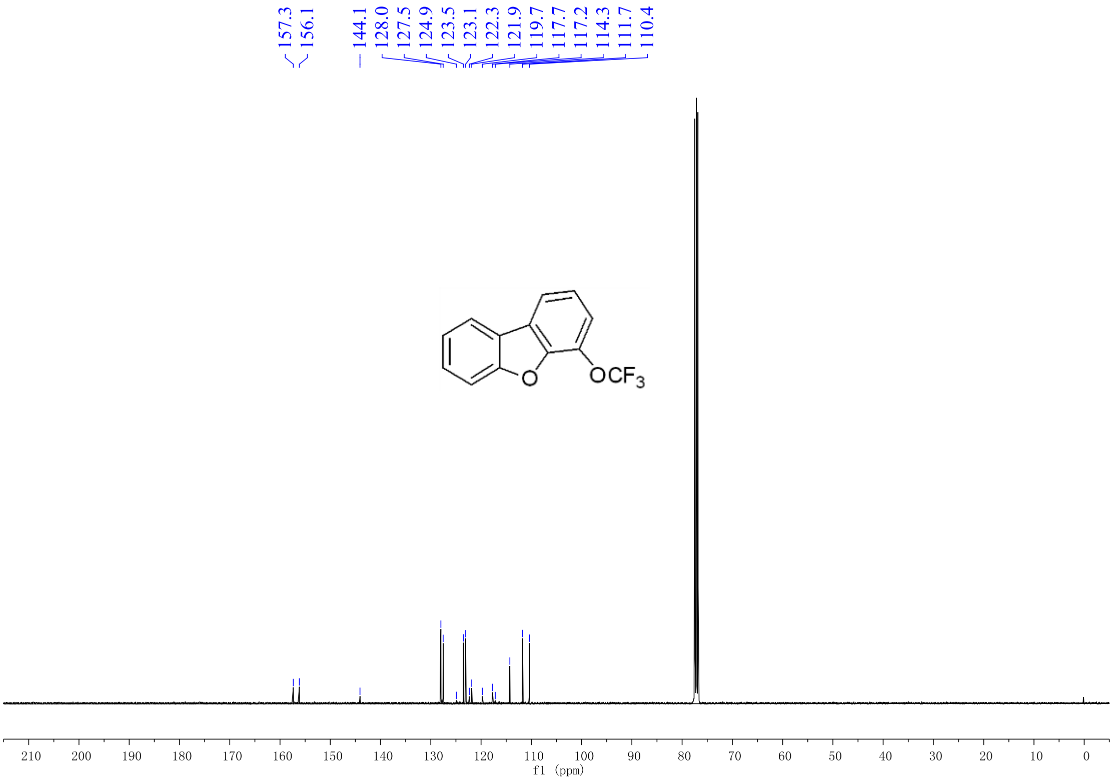

Supplementary Figure 122. ^{13}C NMR spectrum (101 MHz, $CDCl_3$) of ***iso*-3dd**

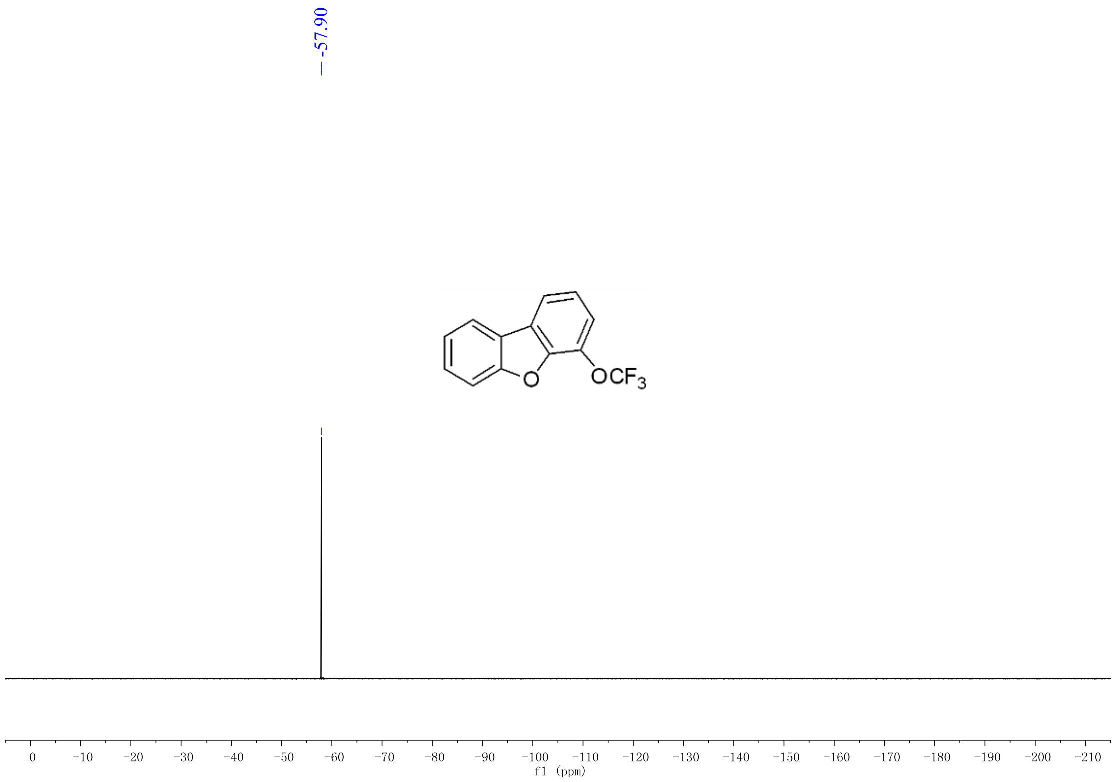

Supplementary Figure 123. ^{19}F NMR spectrum (376 MHz, $CDCl_3$) of ***iso*-3dd**

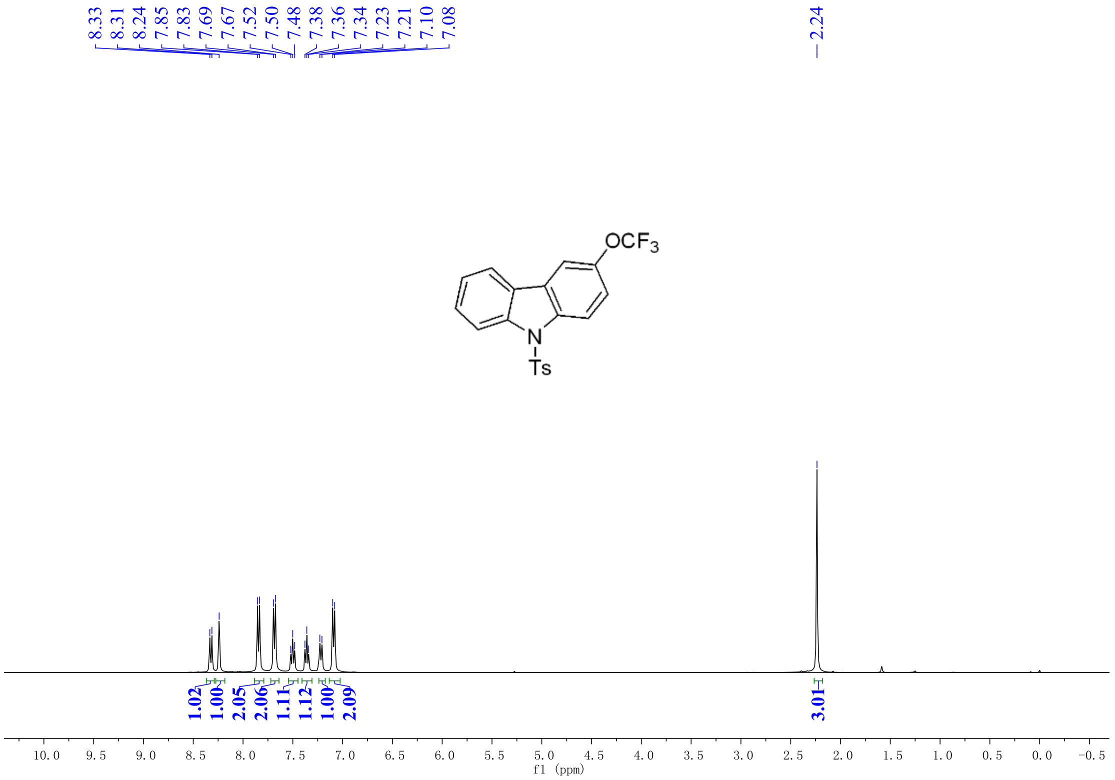

Supplementary Figure 124. ^{1}H NMR spectrum (400 MHz, $CDCl_3$) of **3ee**

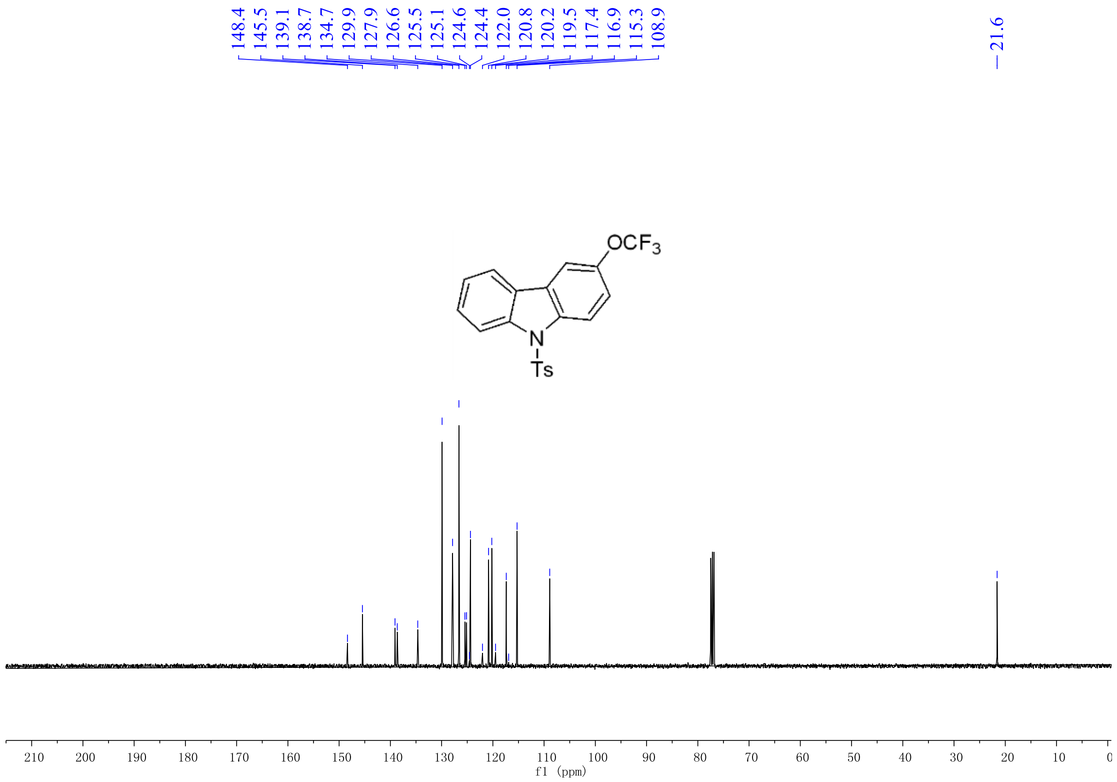

Supplementary Figure 125. ^{13}C NMR spectrum (101 MHz, $CDCl_3$) of **3ee**

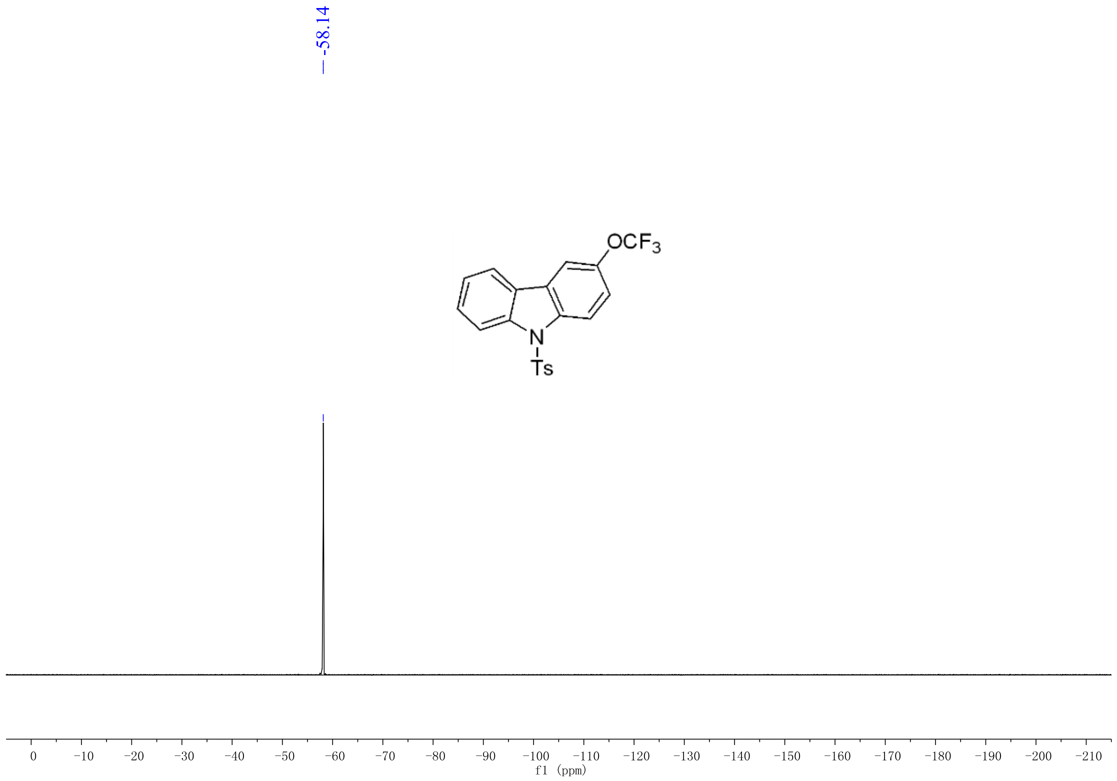

Supplementary Figure 126. ^{19}F NMR spectrum (376 MHz, $CDCl_3$) of **3ee**

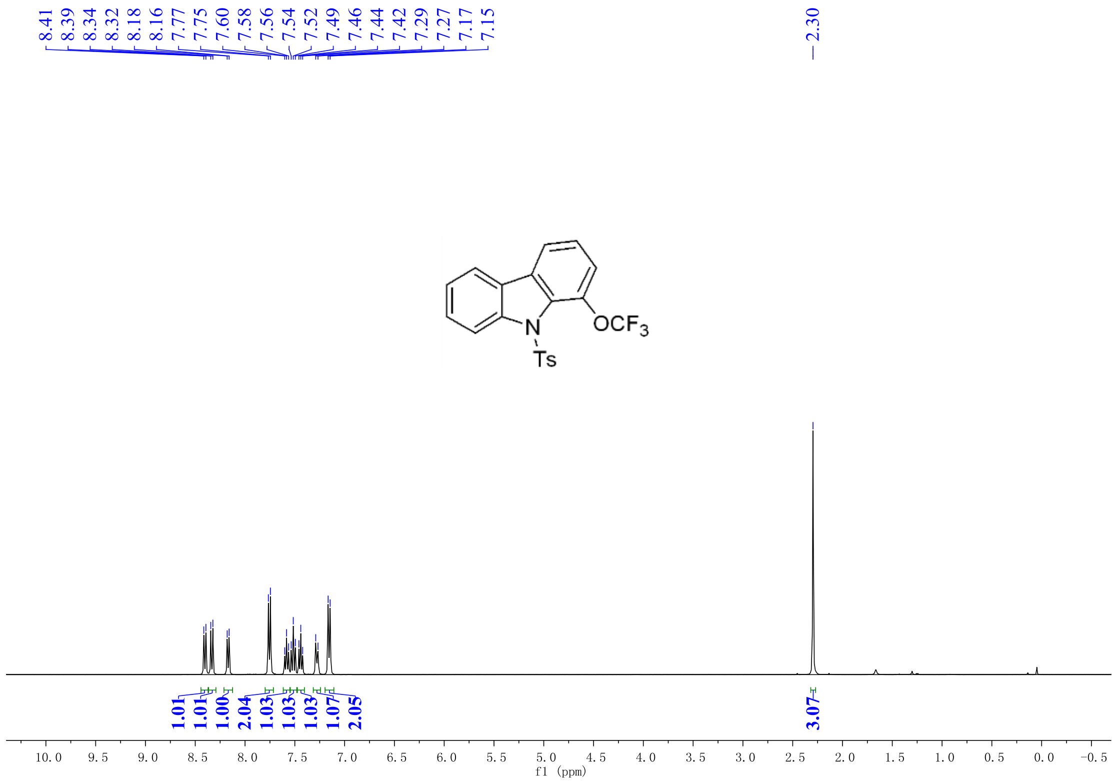

Supplementary Figure 127. ^{1}H NMR spectrum (400 MHz, $CDCl_3$) of ***iso*-3ee**

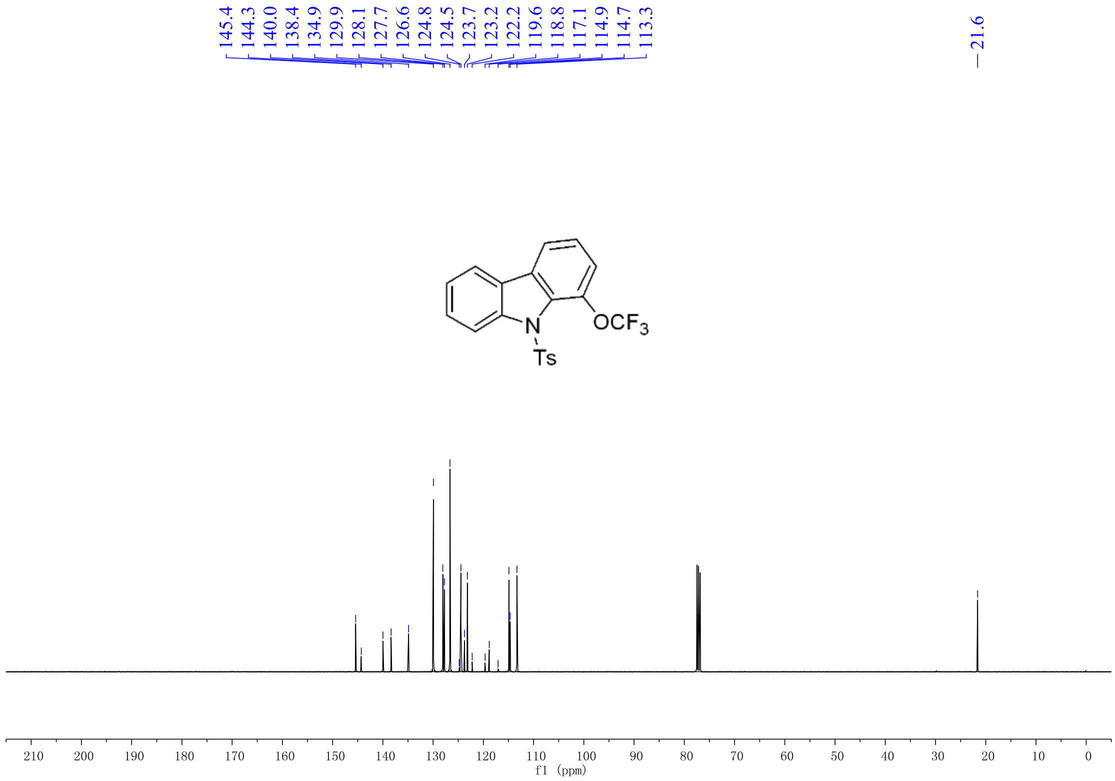

Supplementary Figure 128. ^{13}C NMR spectrum (101 MHz, $CDCl_3$) of ***iso*-3ee**

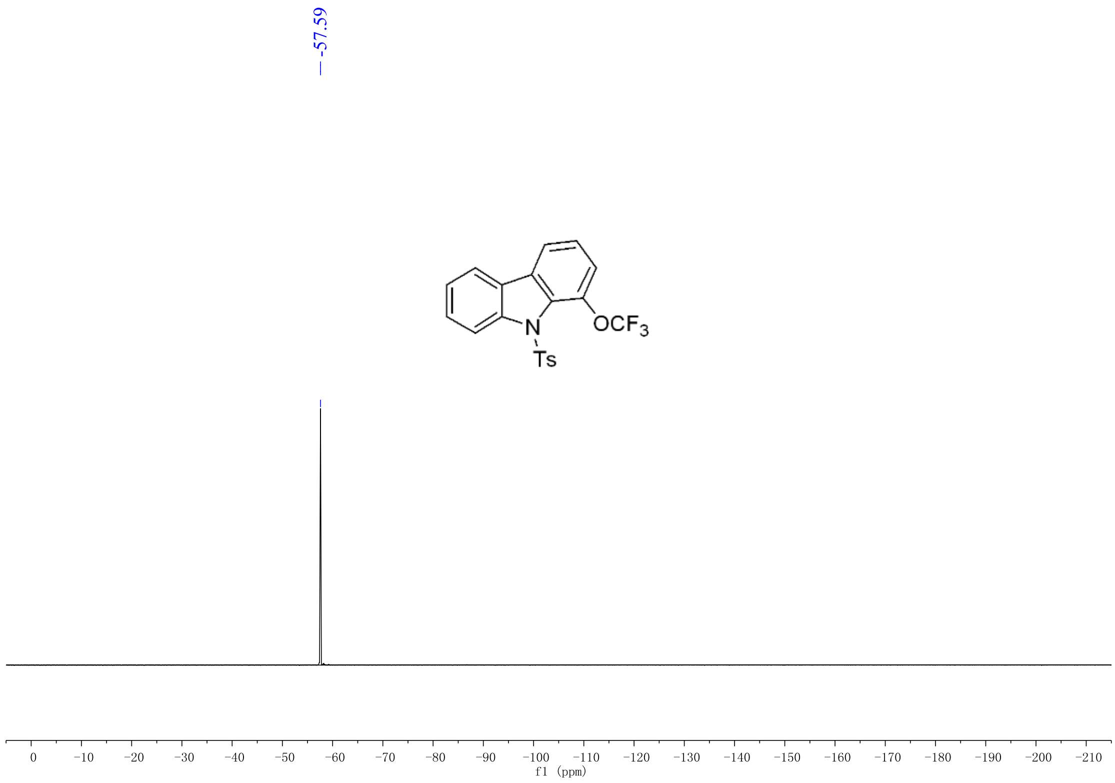

Supplementary Figure 129. ^{19}F NMR spectrum (376 MHz, $CDCl_3$) of ***iso*-3ee**

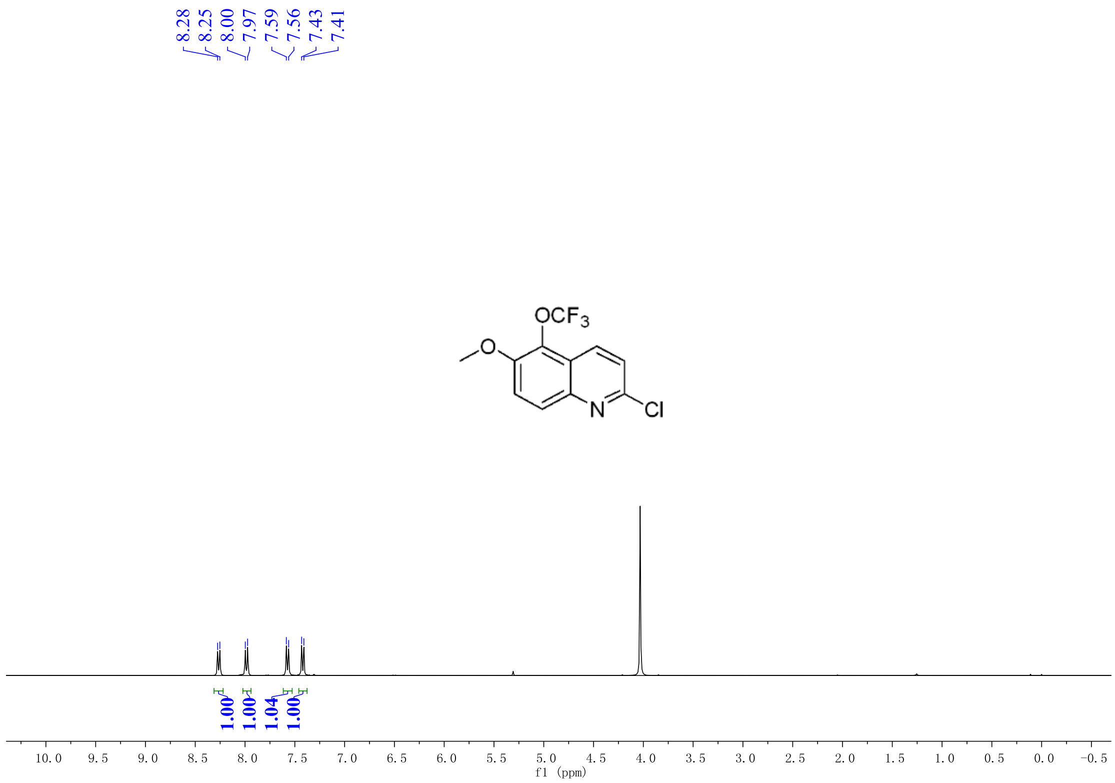

Supplementary Figure 130. ^{1}H NMR spectrum (400 MHz, $CDCl_3$) of **3ff**

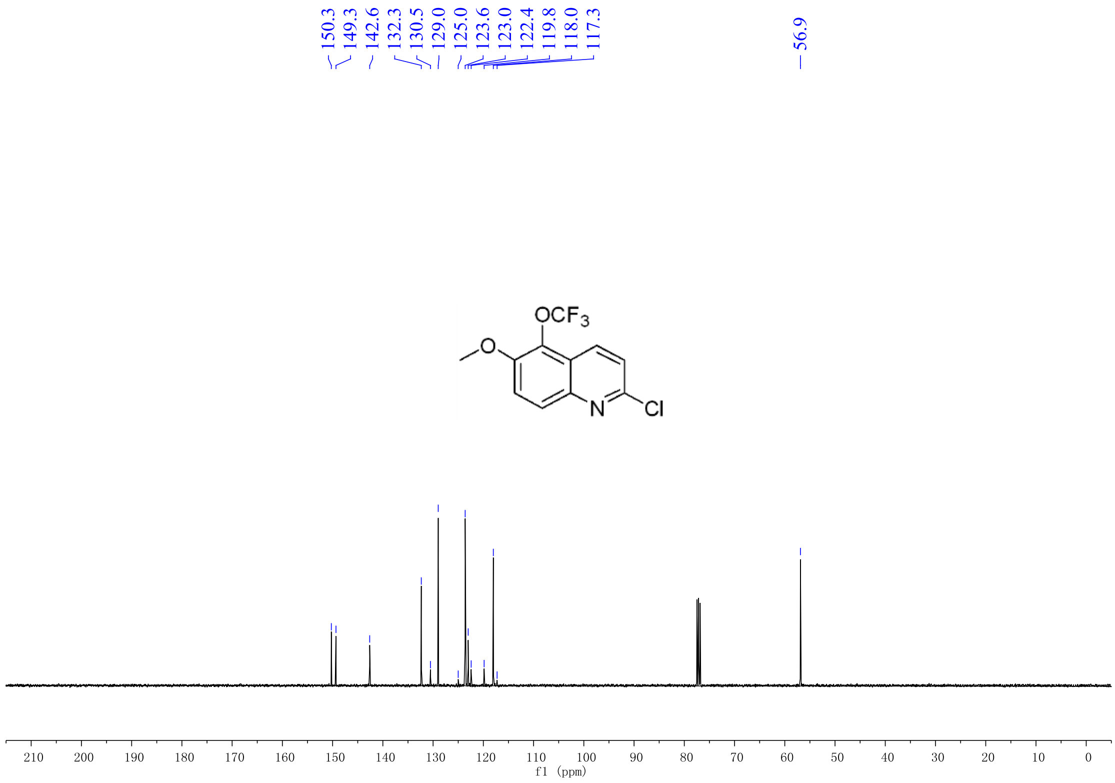

Supplementary Figure 131. ^{13}C NMR spectrum (101 MHz, $CDCl_3$) of **3ff**

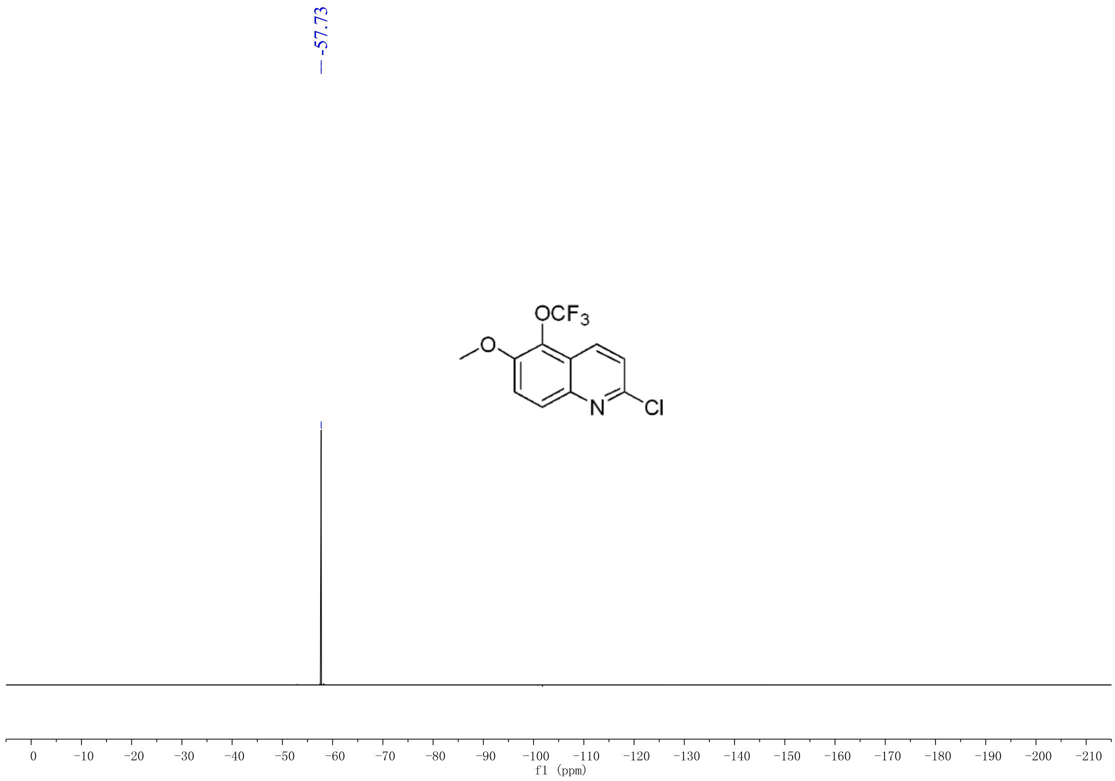

Supplementary Figure 132. ^{19}F NMR spectrum (376 MHz, $CDCl_3$) of **3ff**

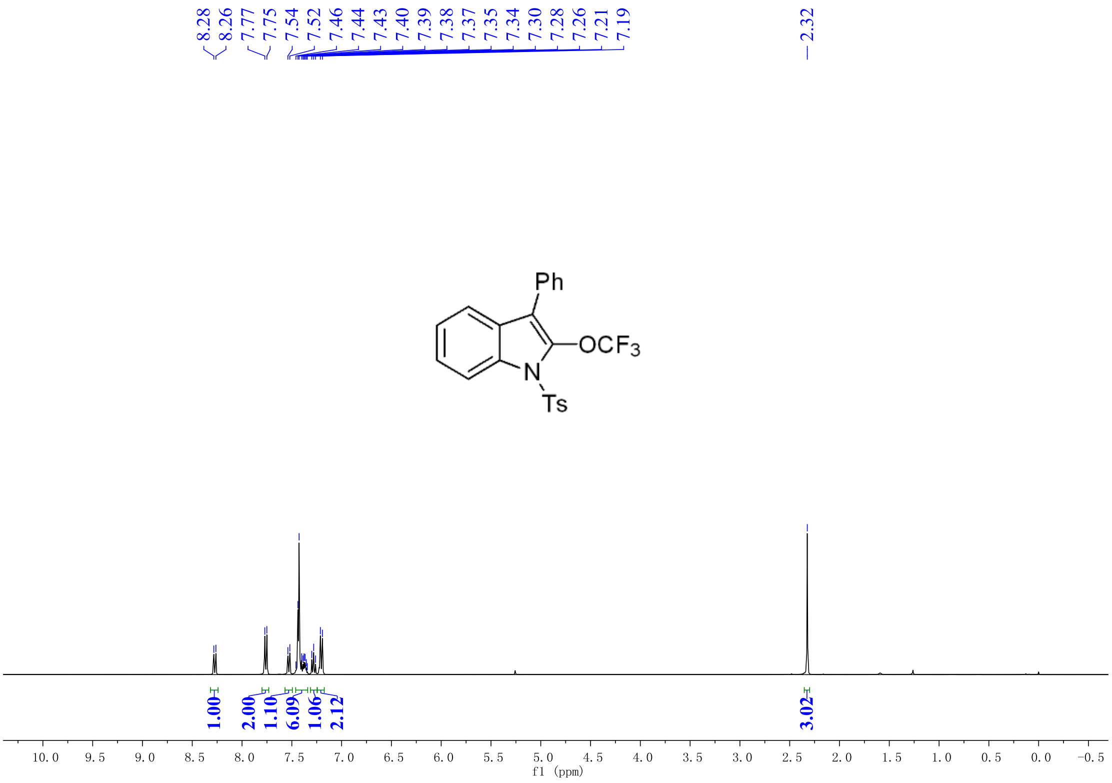

Supplementary Figure 133. ^{1}H NMR spectrum (400 MHz, $CDCl_3$) of **3gg**

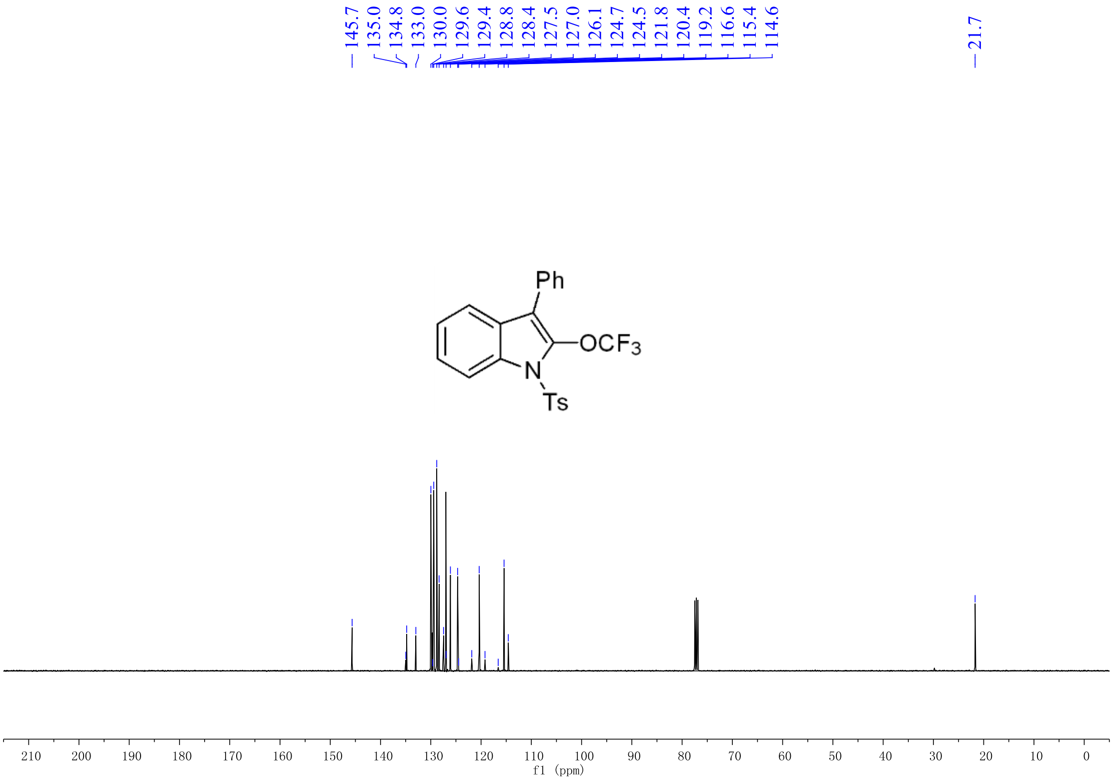

Supplementary Figure 134. ^{13}C NMR spectrum (101 MHz, $CDCl_3$) of **3gg**

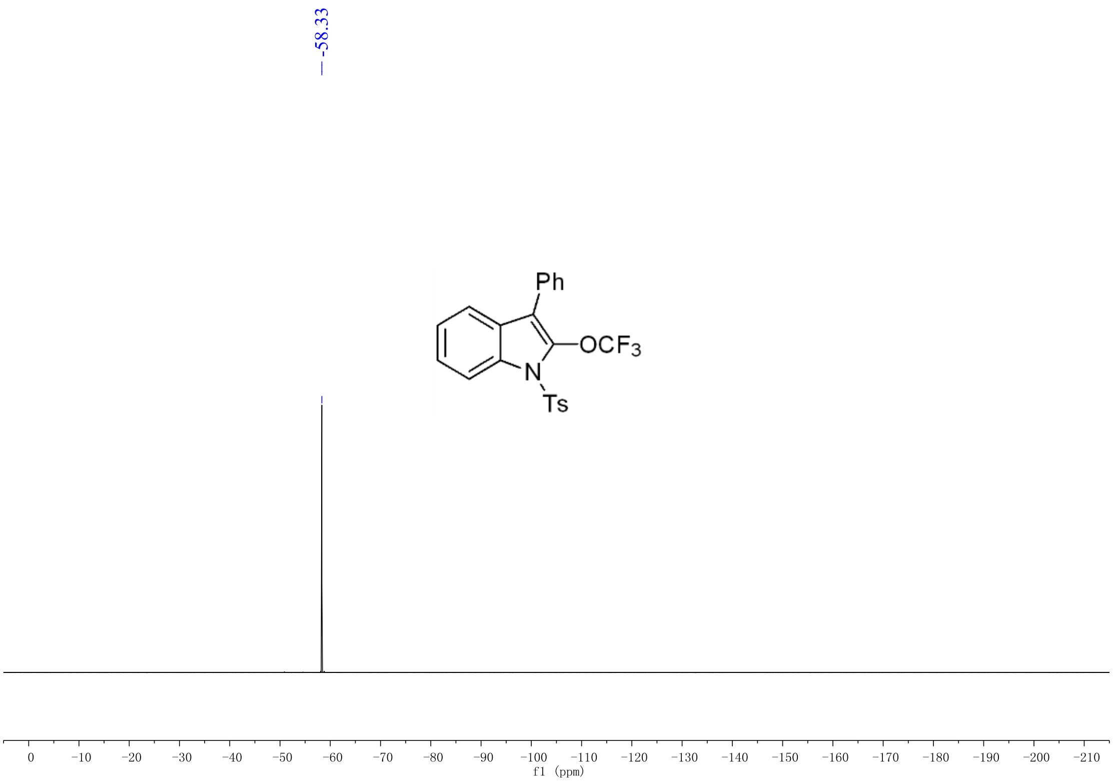

Supplementary Figure 135. ^{19}F NMR spectrum (376 MHz, $CDCl_3$) of **3gg**

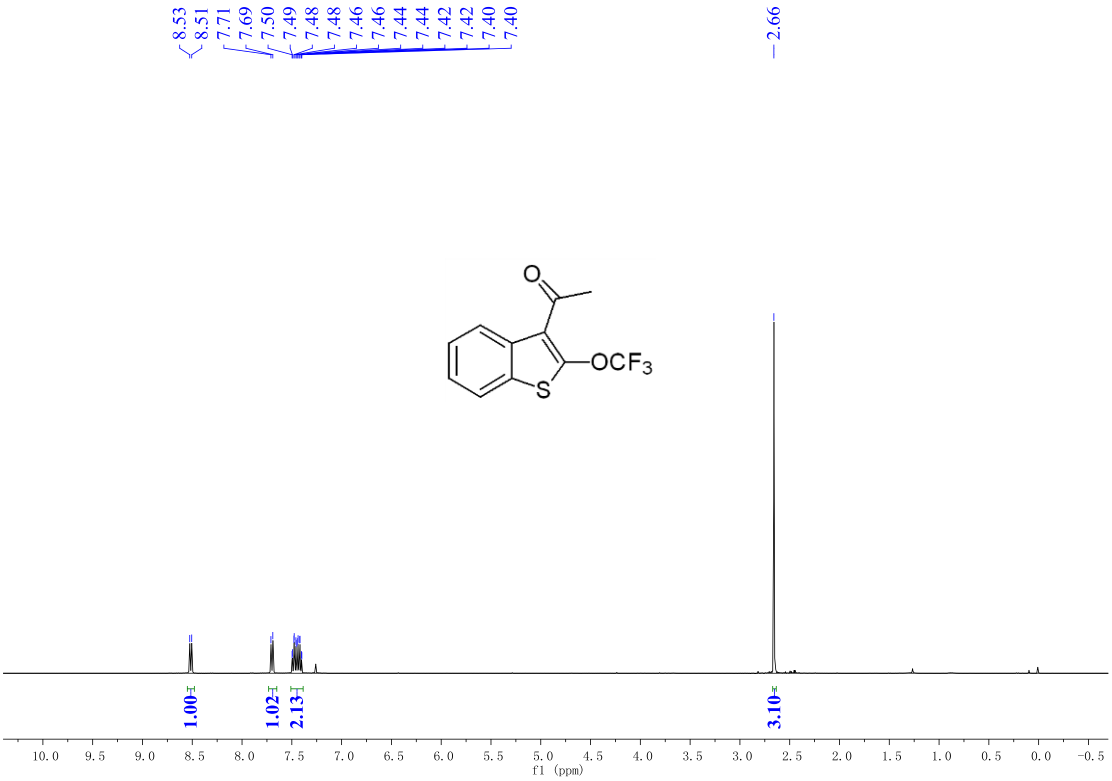

Supplementary Figure 136. ^{1}H NMR spectrum (400 MHz, $CDCl_3$) of **3hh**

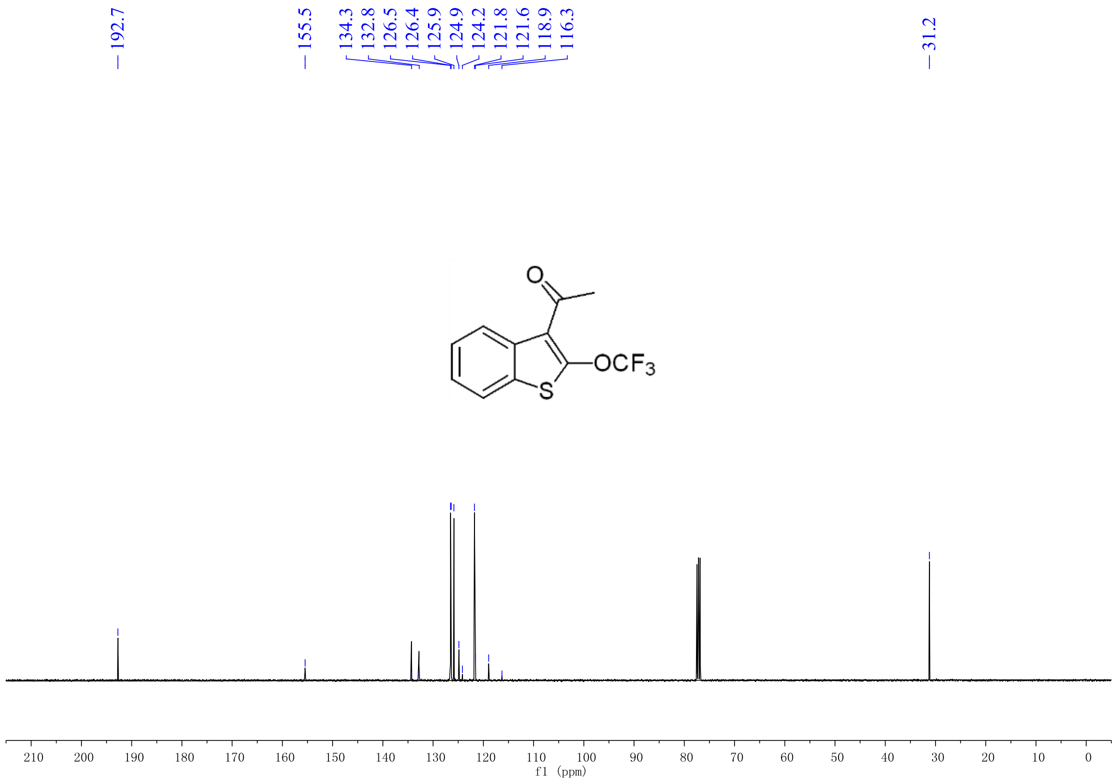

Supplementary Figure 137. ^{13}C NMR spectrum (101 MHz, $CDCl_3$) of **3hh**

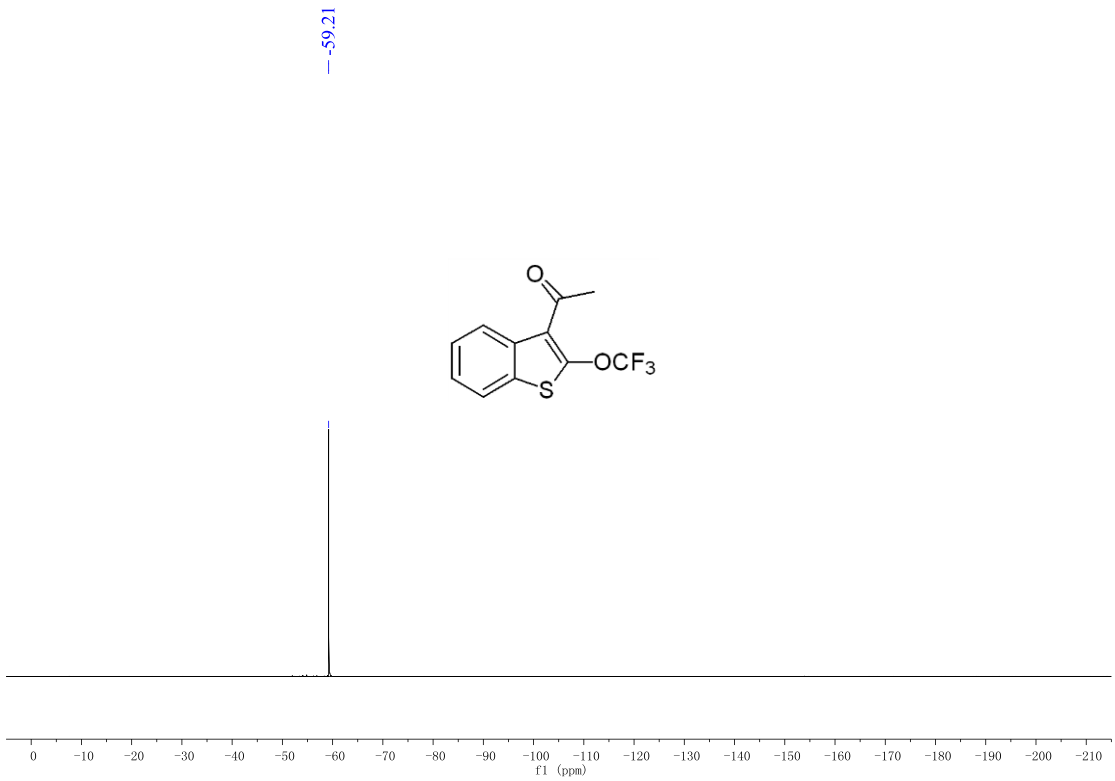

Supplementary Figure 138. ^{19}F NMR spectrum (376 MHz, $CDCl_3$) of **3hh**

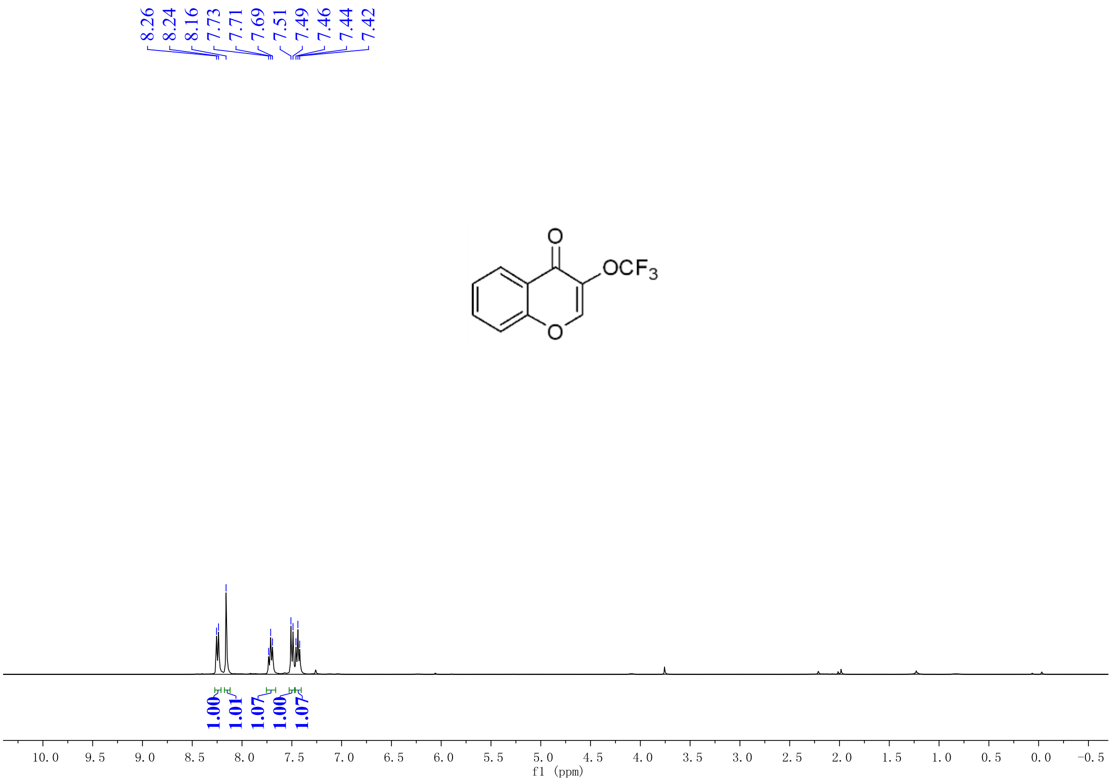

Supplementary Figure 139. ^{1}H NMR spectrum (400 MHz, $CDCl_3$) of **3ii**

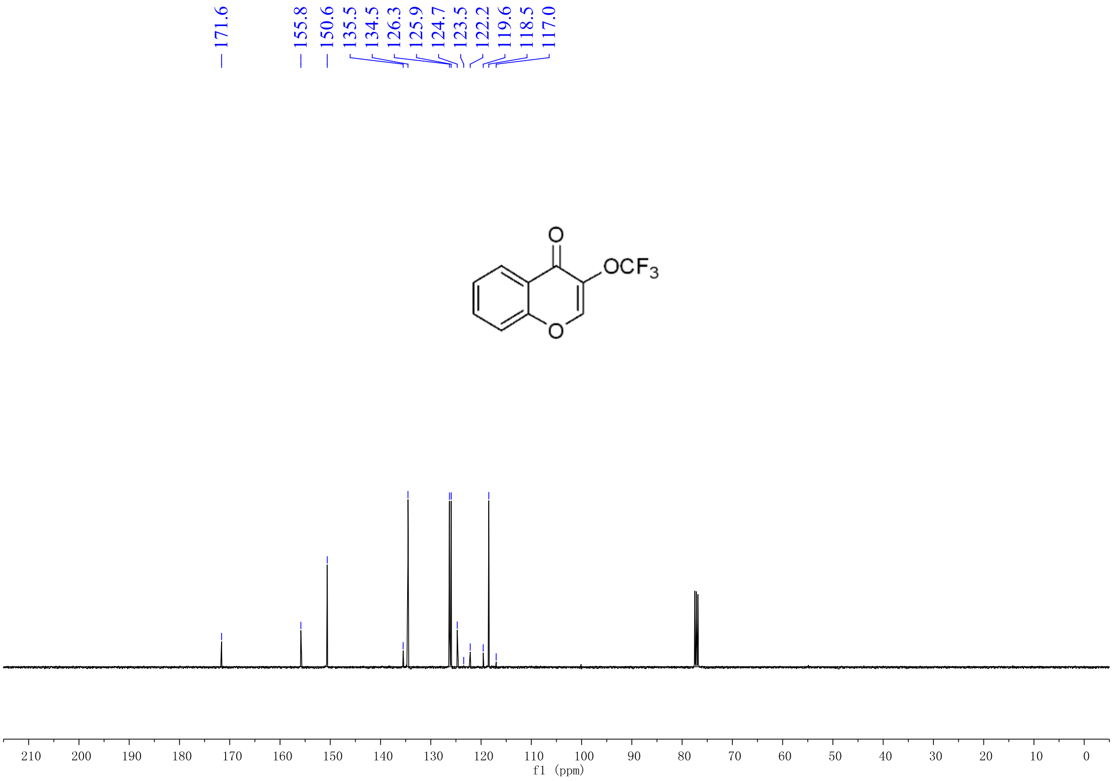

Supplementary Figure 140. ^{13}C NMR spectrum (101 MHz, $CDCl_3$) of **3ii**

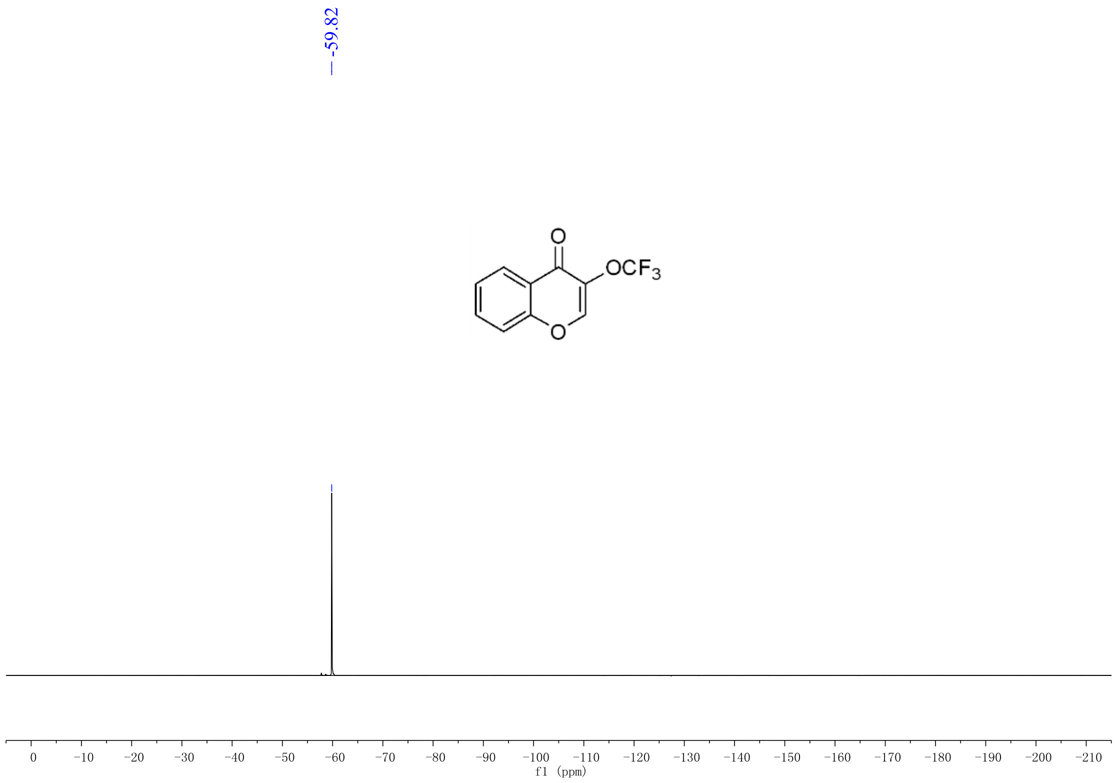

Supplementary Figure 141. ^{19}F NMR spectrum (376 MHz, $CDCl_3$) of **3ii**

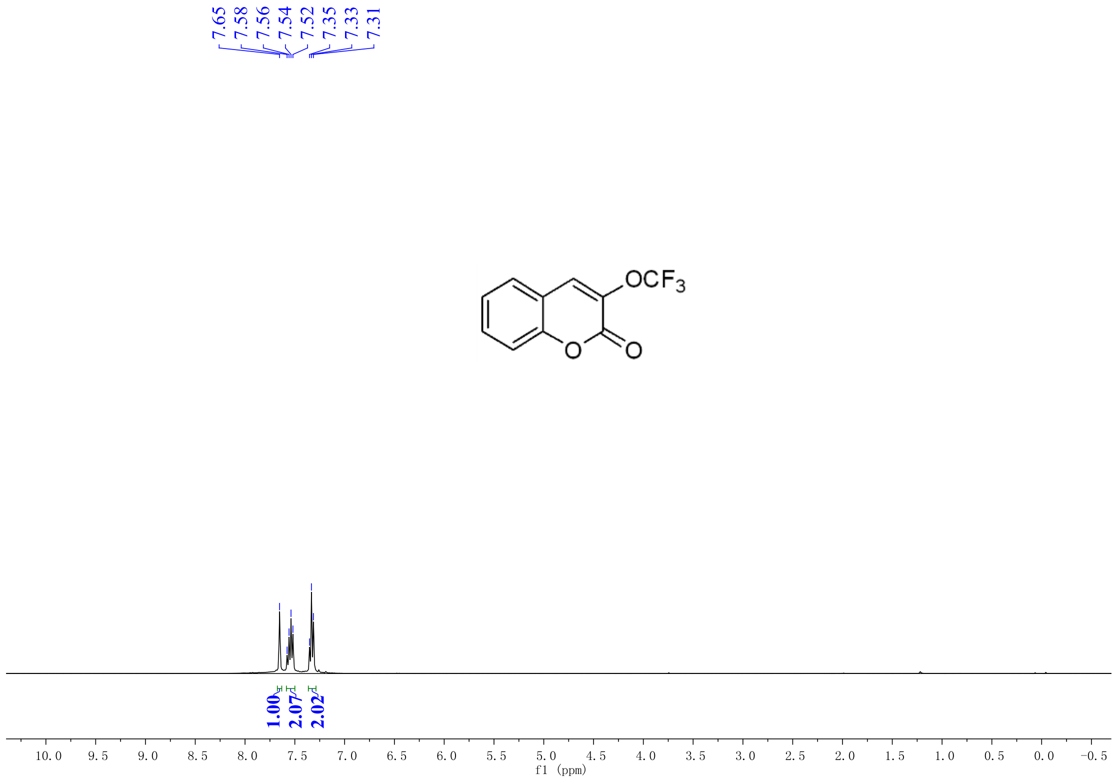

Supplementary Figure 142. ^{1}H NMR spectrum (400 MHz, $CDCl_3$) of **3jj**

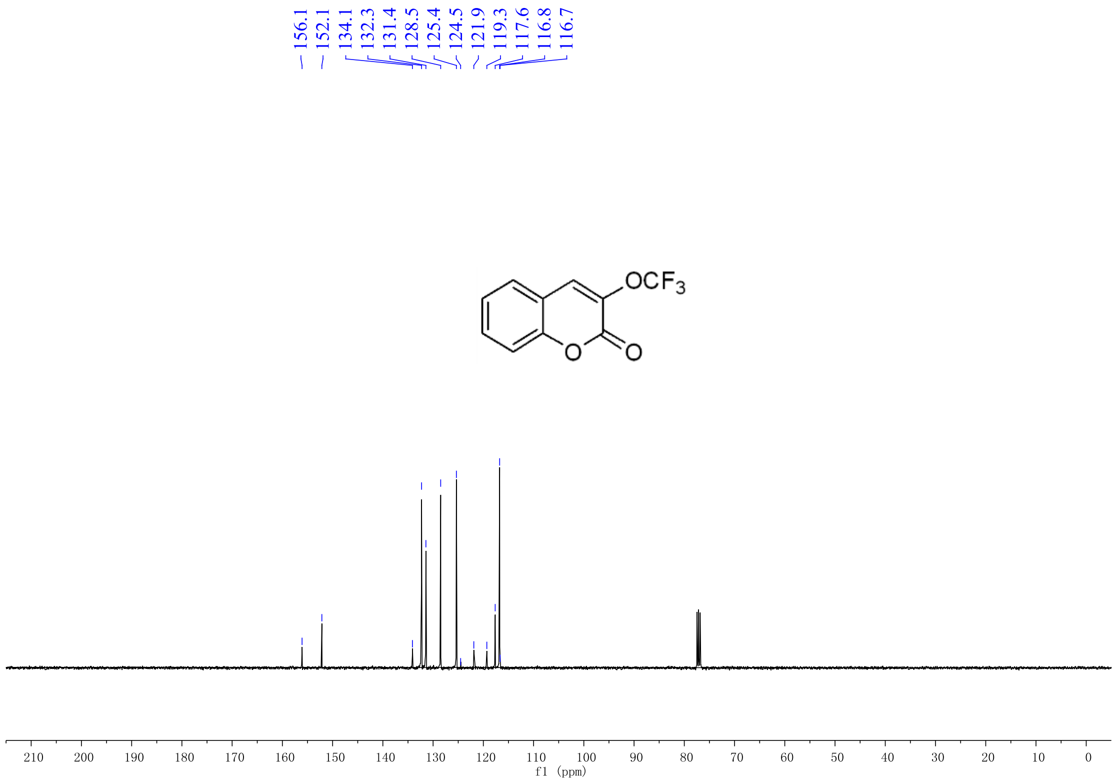

Supplementary Figure 143. ^{13}C NMR spectrum (101 MHz, $CDCl_3$) of **3jj**

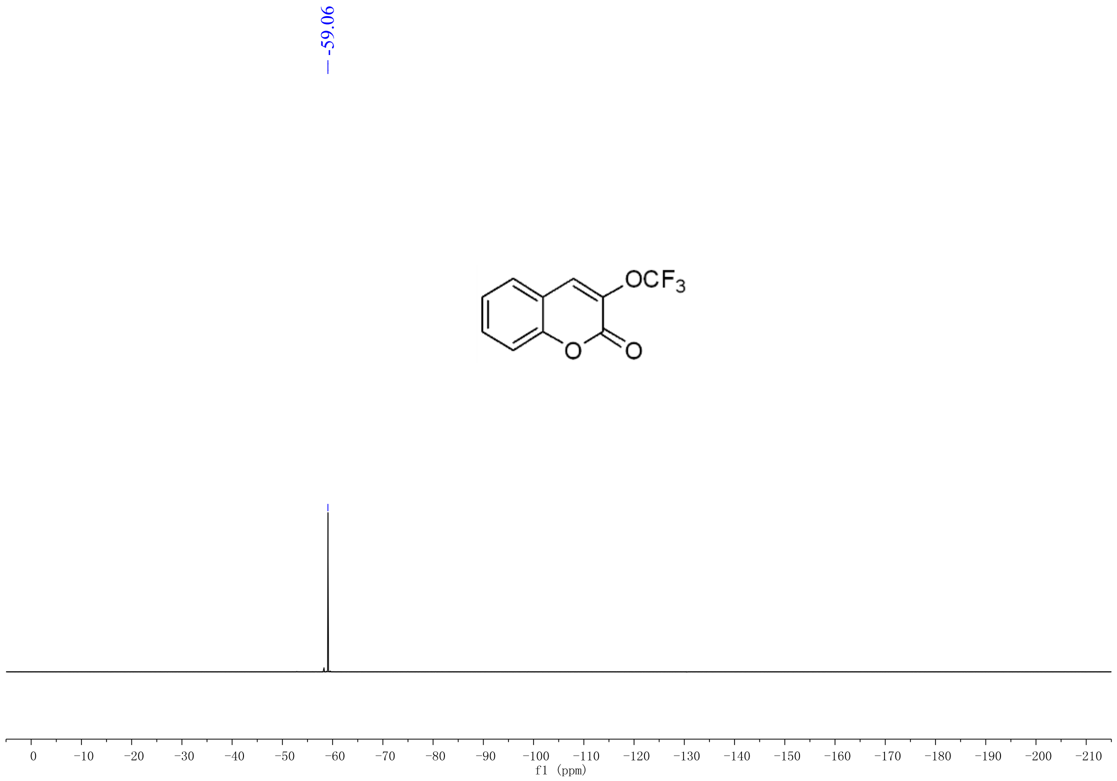

Supplementary Figure 144. ^{19}F NMR spectrum (376 MHz, $CDCl_3$) of **3jj**

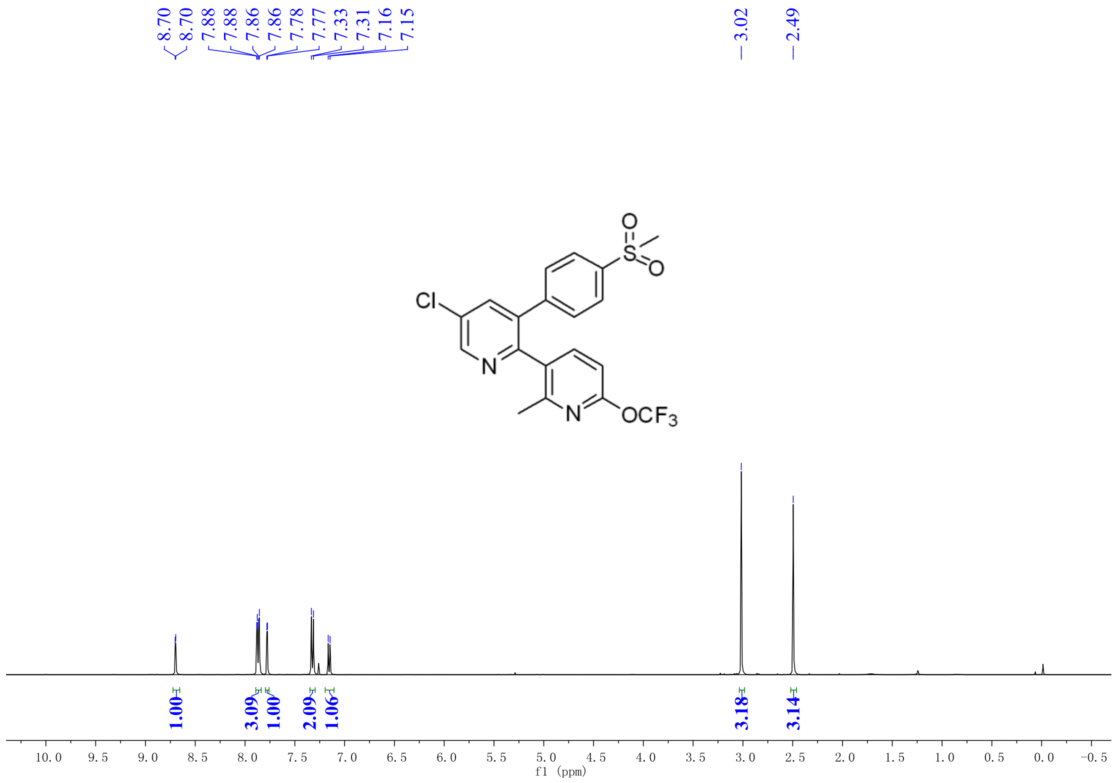

Supplementary Figure 145. ^{1}H NMR spectrum (400 MHz, $CDCl_3$) of **4kk**

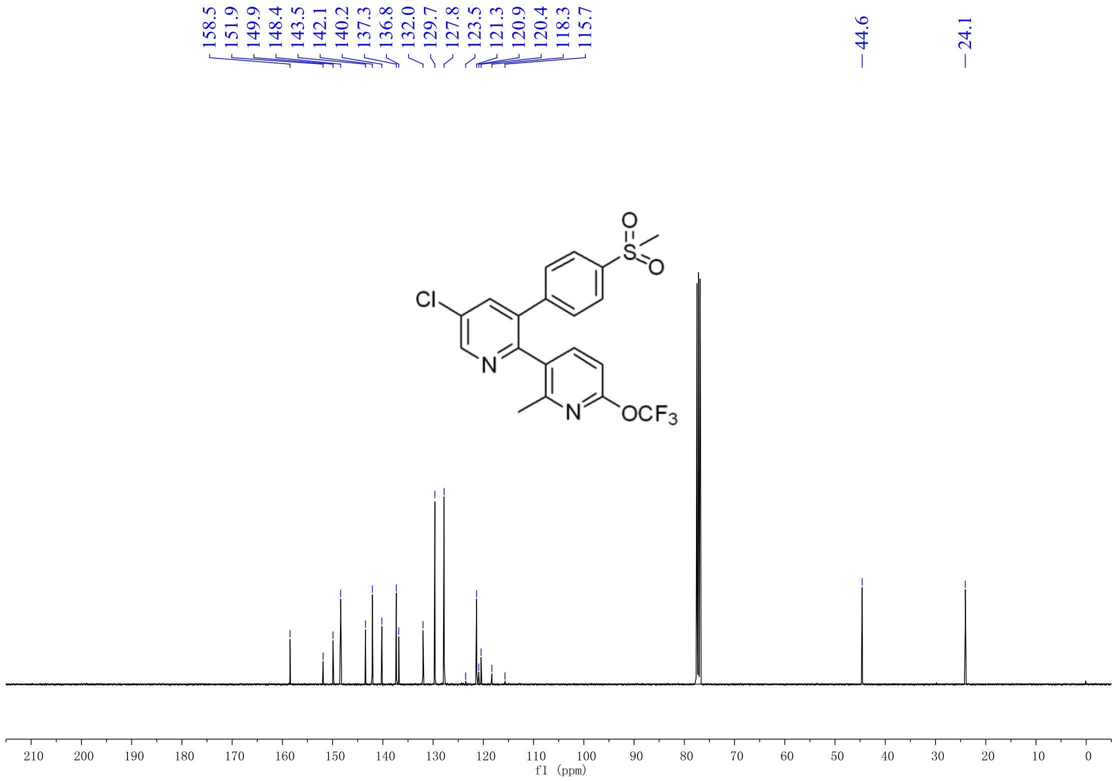

Supplementary Figure 146. ^{13}C NMR spectrum (101 MHz, $CDCl_3$) of **4kk**

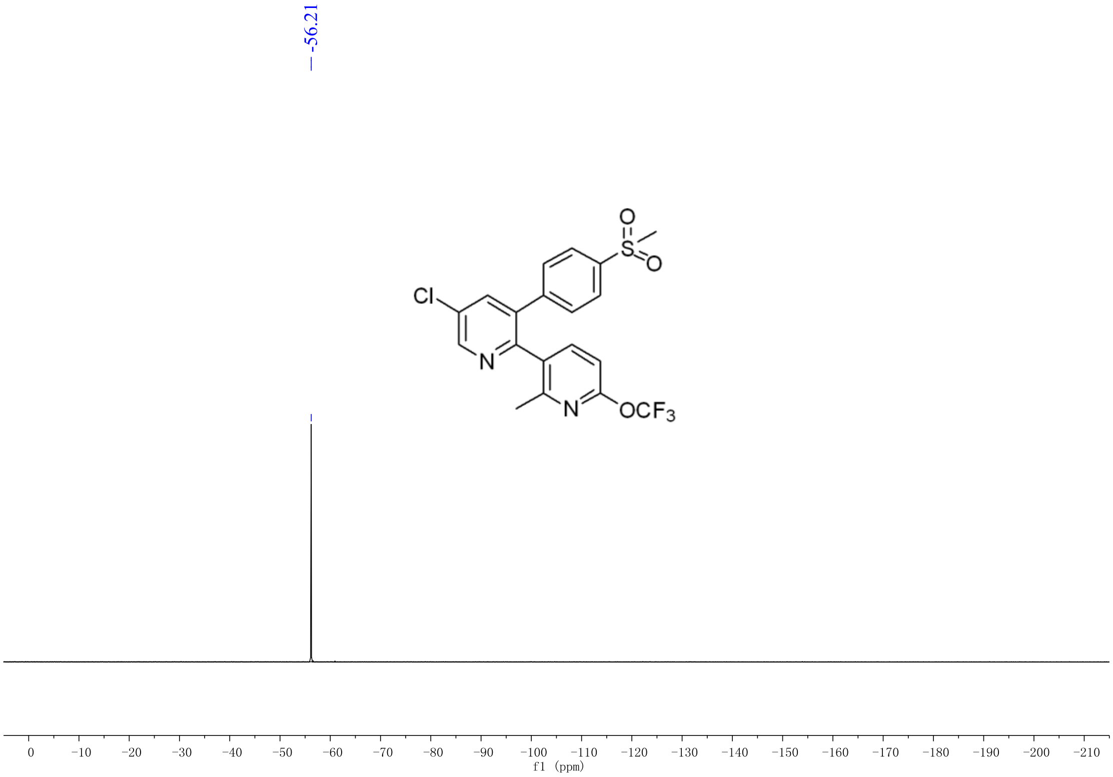

Supplementary Figure 147. ^{19}F NMR spectrum (376 MHz, $CDCl_3$) of **4kk**

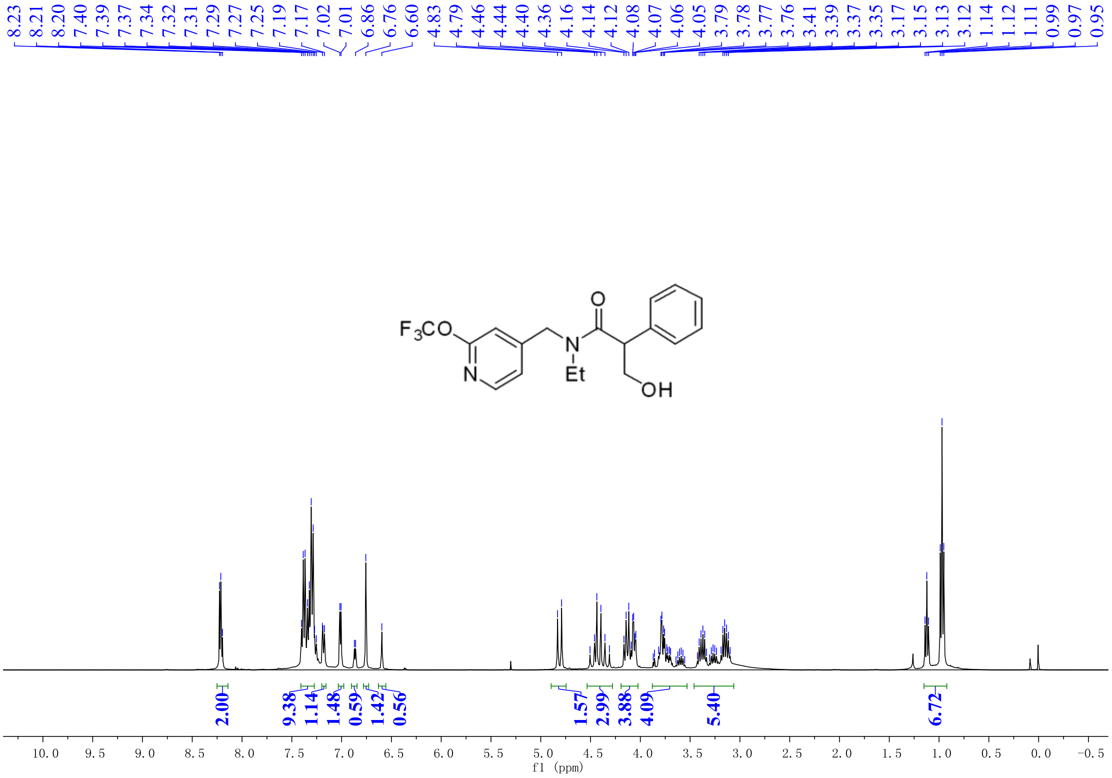

Supplementary Figure 148. ^{1}H NMR spectrum (400 MHz, $CDCl_3$) of **4ll**

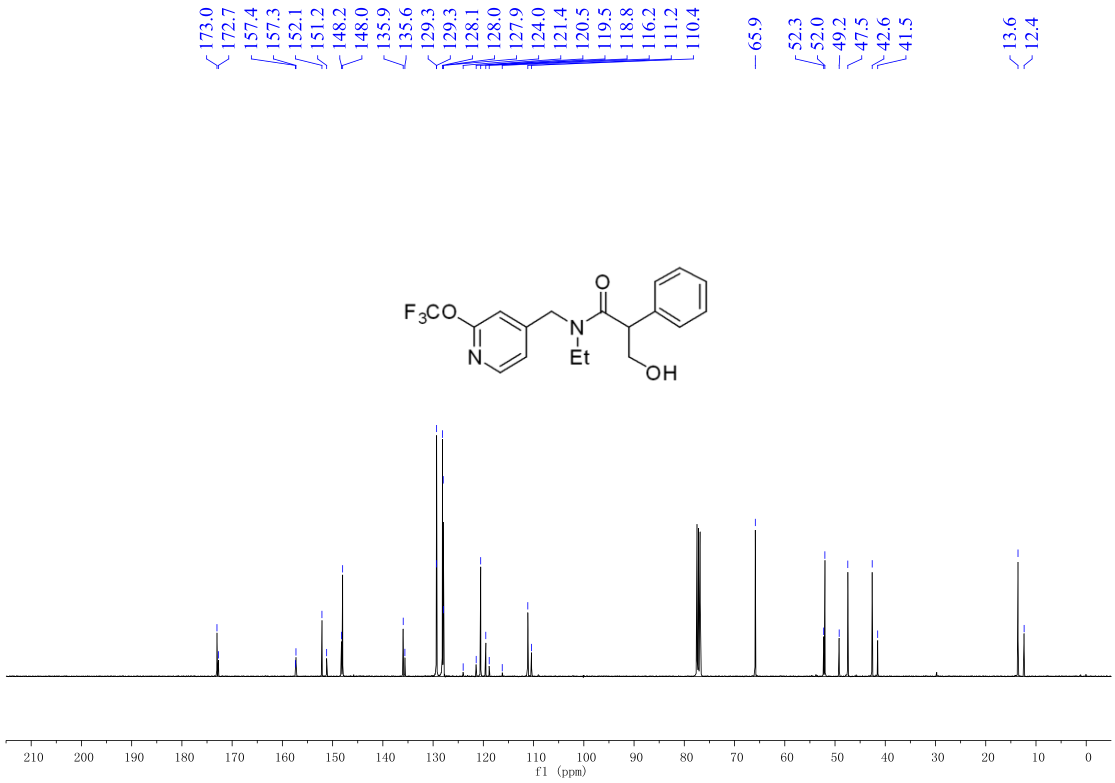

Supplementary Figure 149. ^{13}C NMR spectrum (101 MHz, $CDCl_3$) of **4ll**

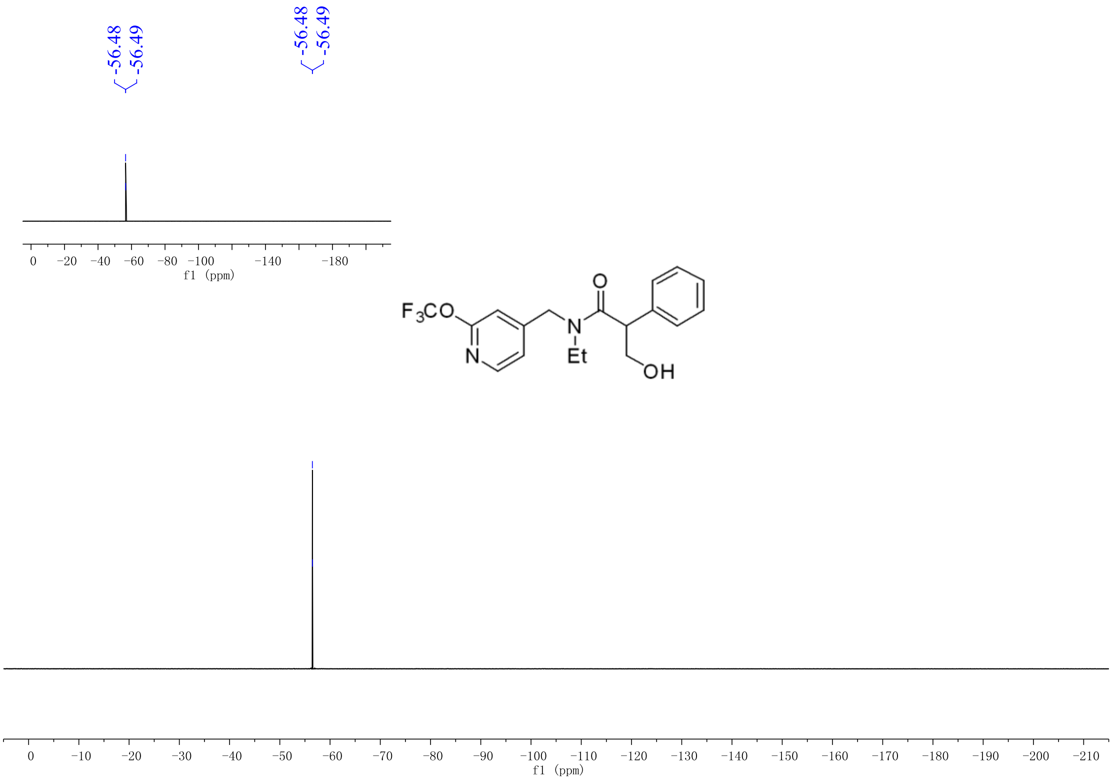

Supplementary Figure 150. ^{19}F NMR spectrum (376 MHz, $CDCl_3$) of **4ll**

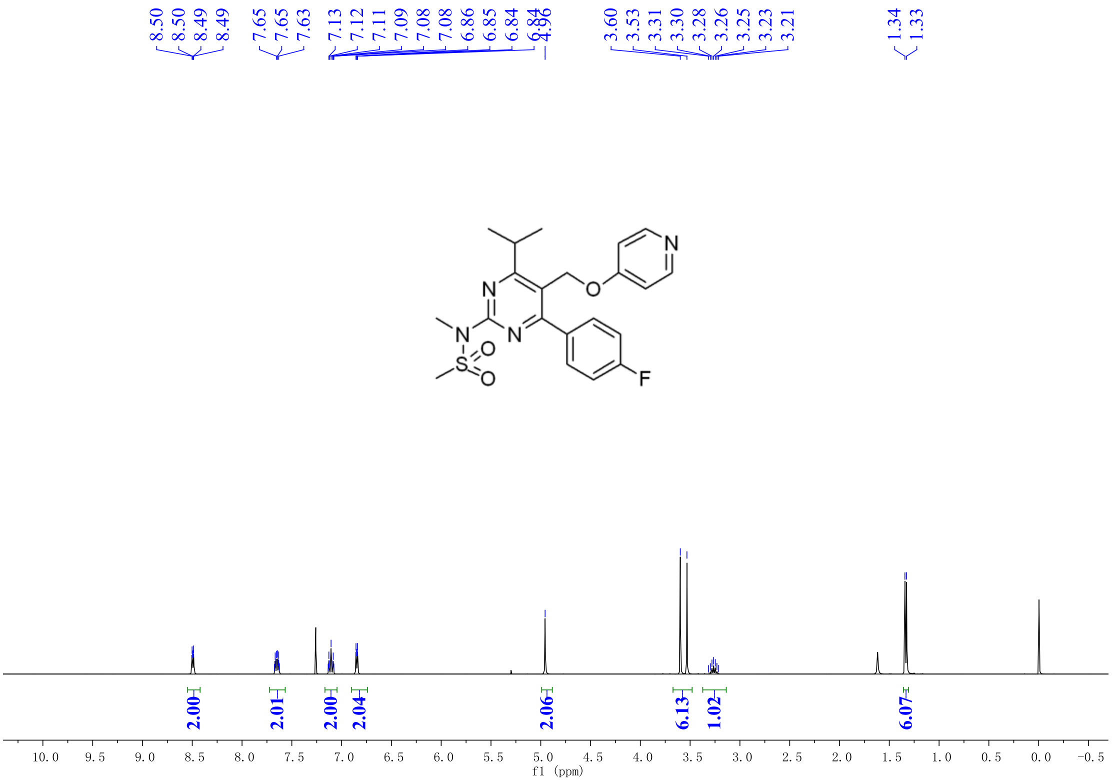

Supplementary Figure 151. ^{1}H NMR spectrum (400 MHz, $CDCl_3$) of **1mm**

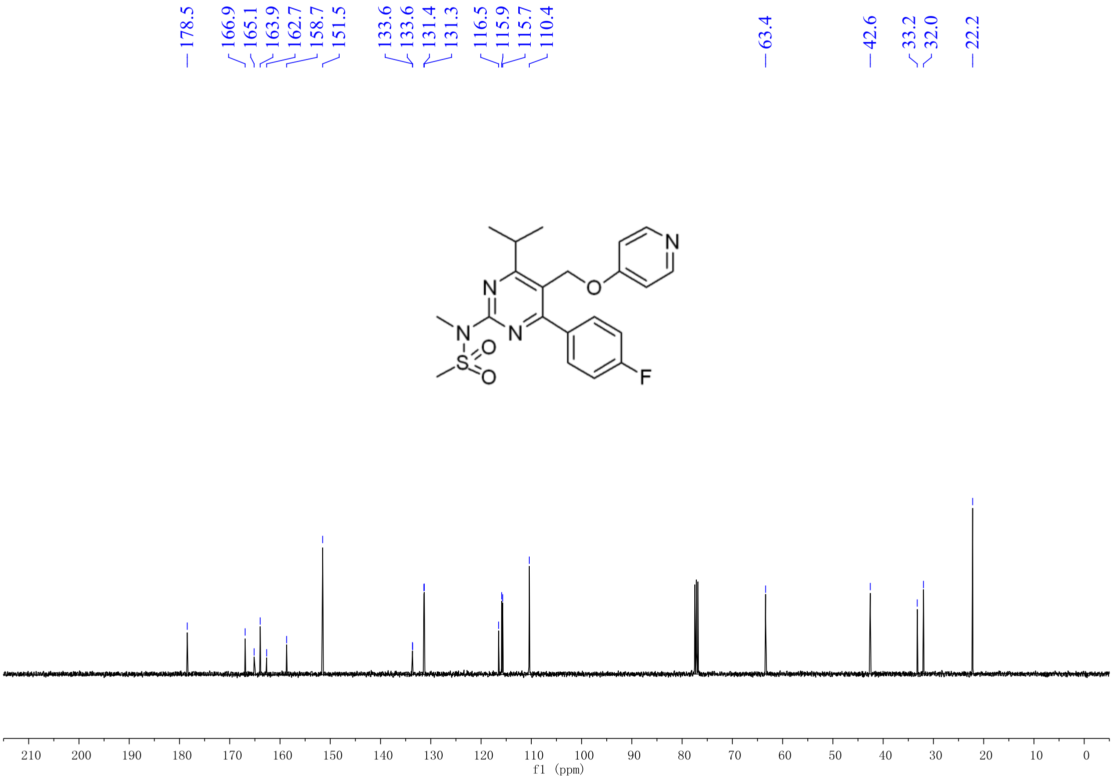

Supplementary Figure 152. ^{13}C NMR spectrum (101 MHz, $CDCl_3$) of **1mm**

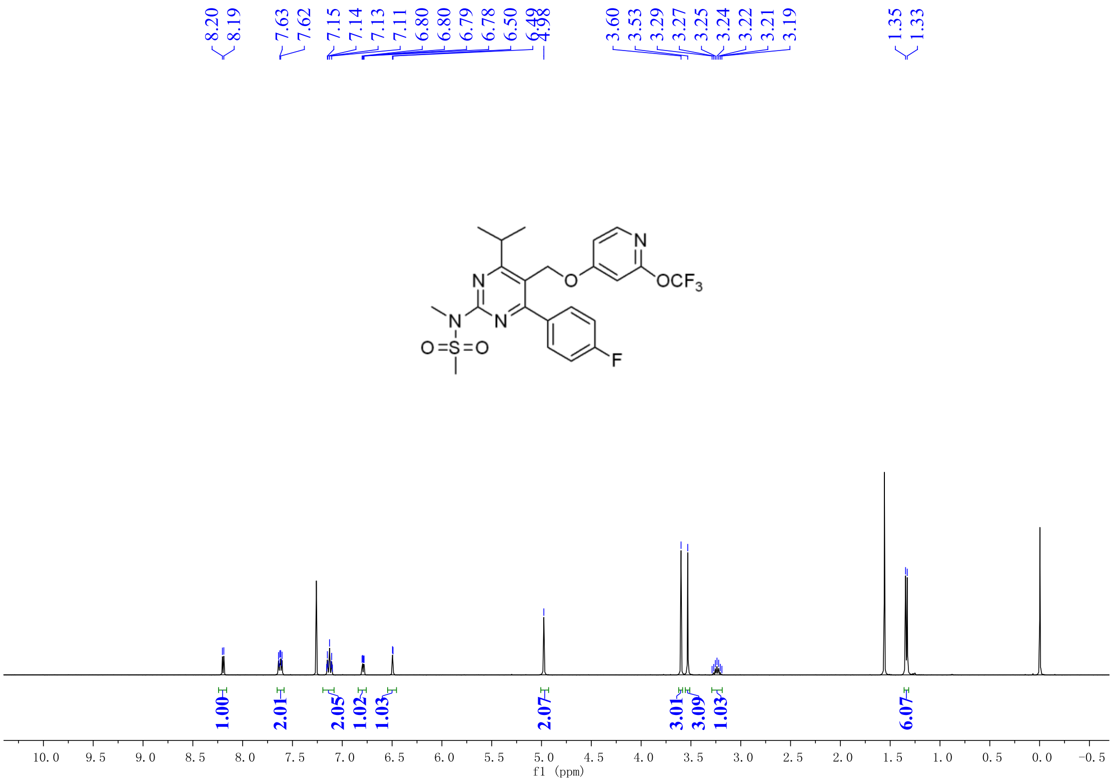

Supplementary Figure 153. ^{1}H NMR spectrum (400 MHz, $CDCl_3$) of **4mm**

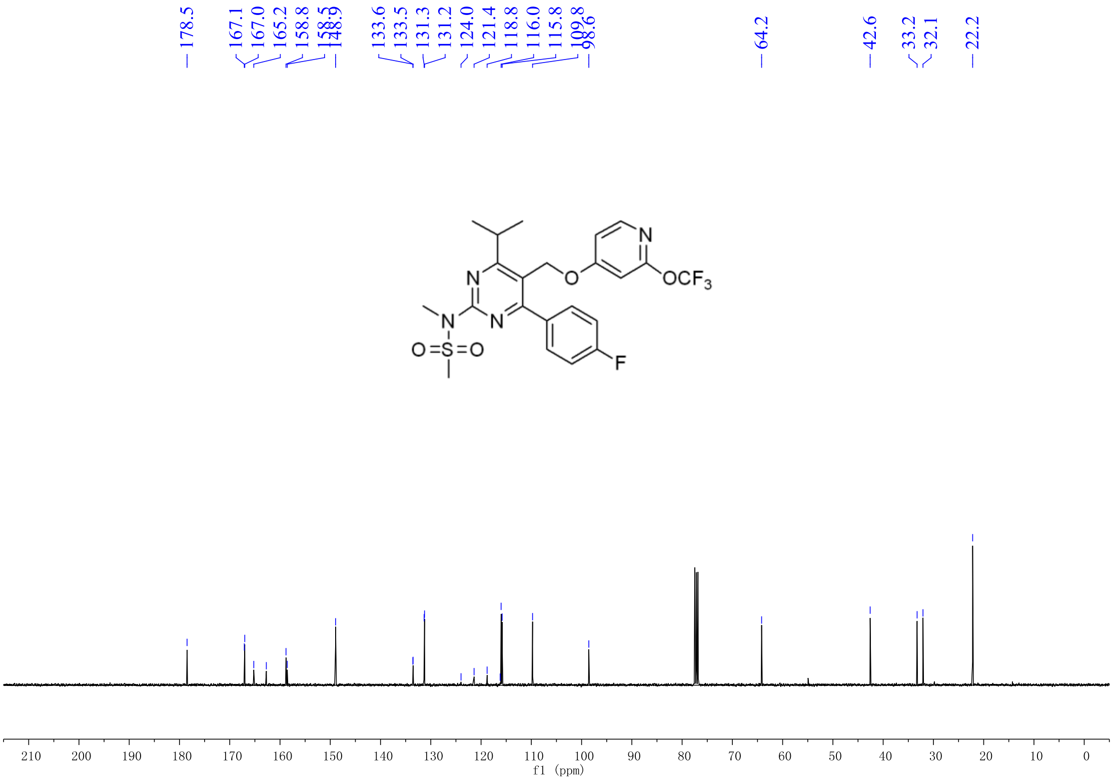

Supplementary Figure 154. ^{13}C NMR spectrum (101 MHz, $CDCl_3$) of **4mm**

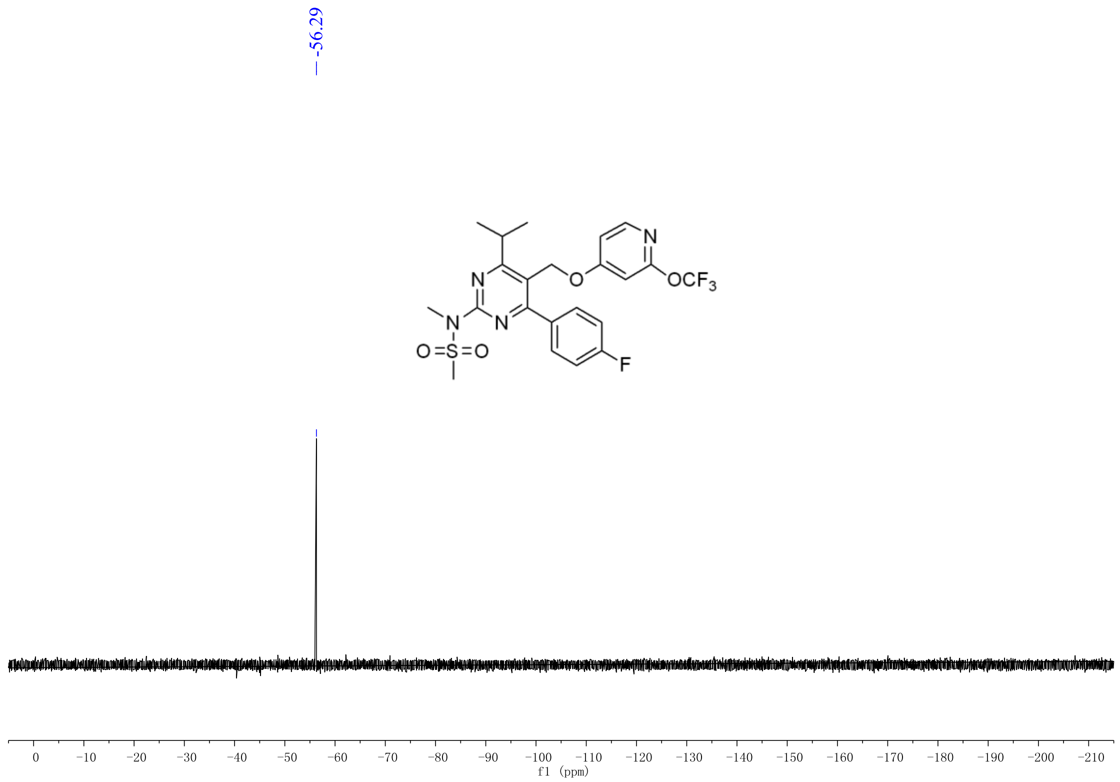

Supplementary Figure 155. ^{19}F NMR spectrum (376 MHz, $CDCl_3$) of **4mm**

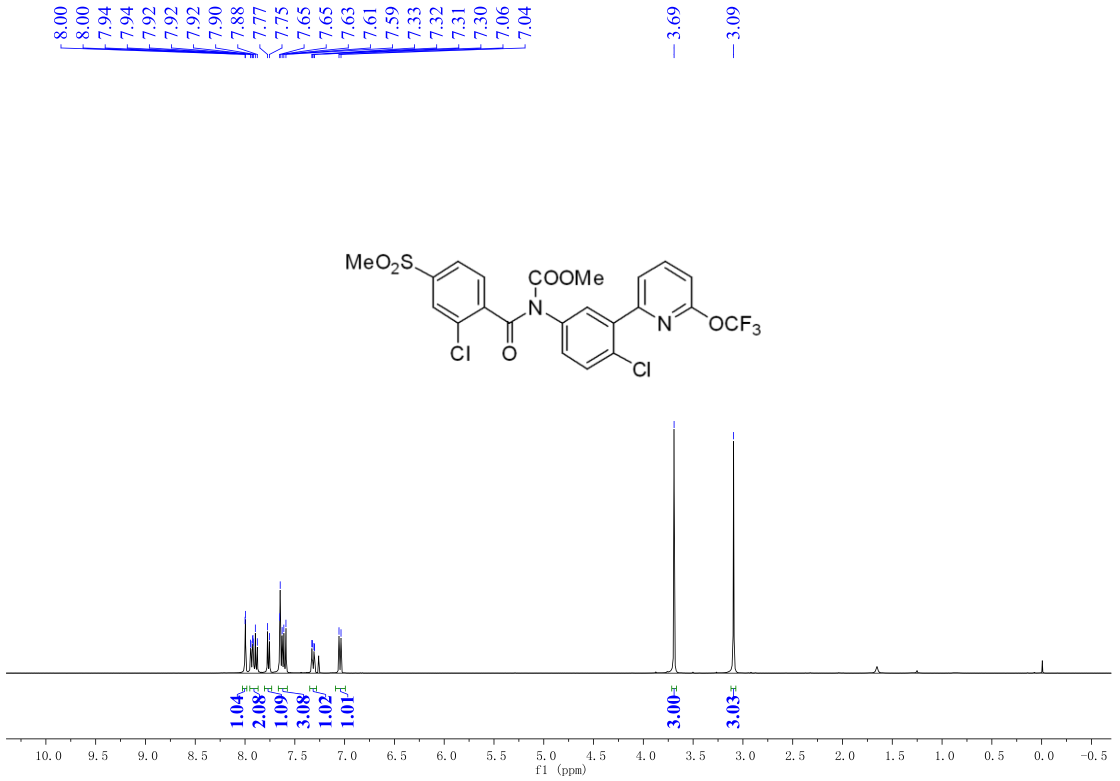

Supplementary Figure 156. ^{1}H NMR spectrum (400 MHz, $CDCl_3$) of **4nn**

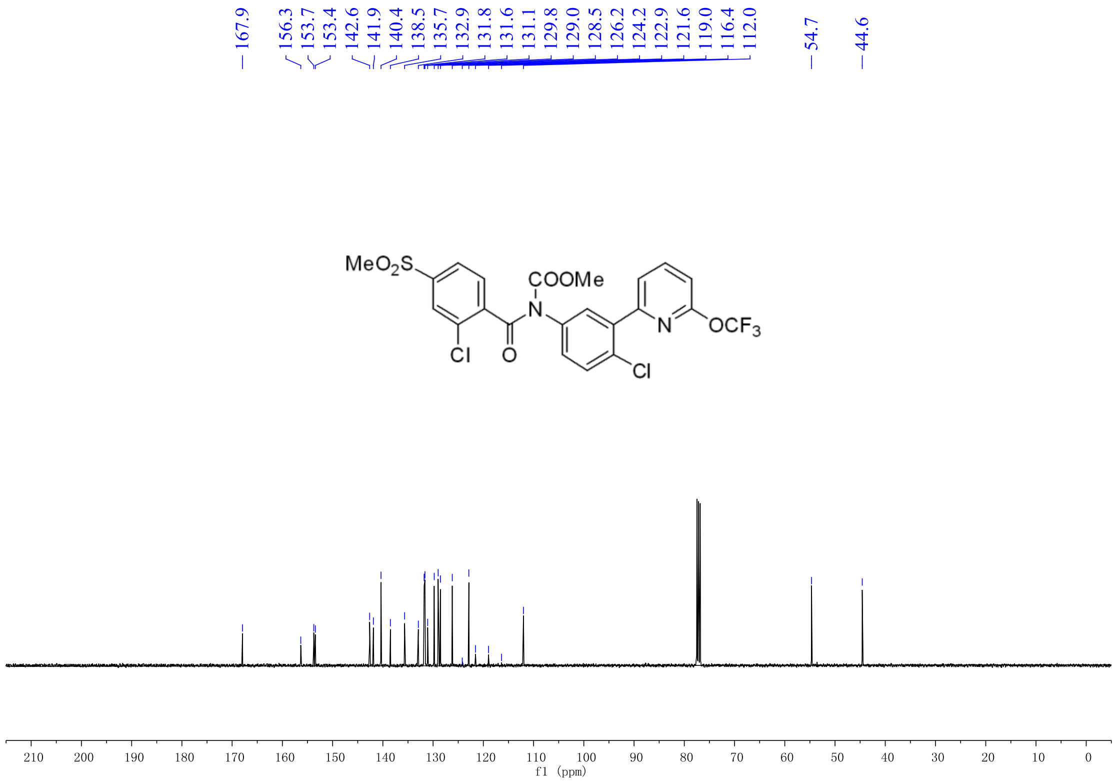

Supplementary Figure 157. ^{13}C NMR spectrum (101 MHz, $CDCl_3$) of **4nn**

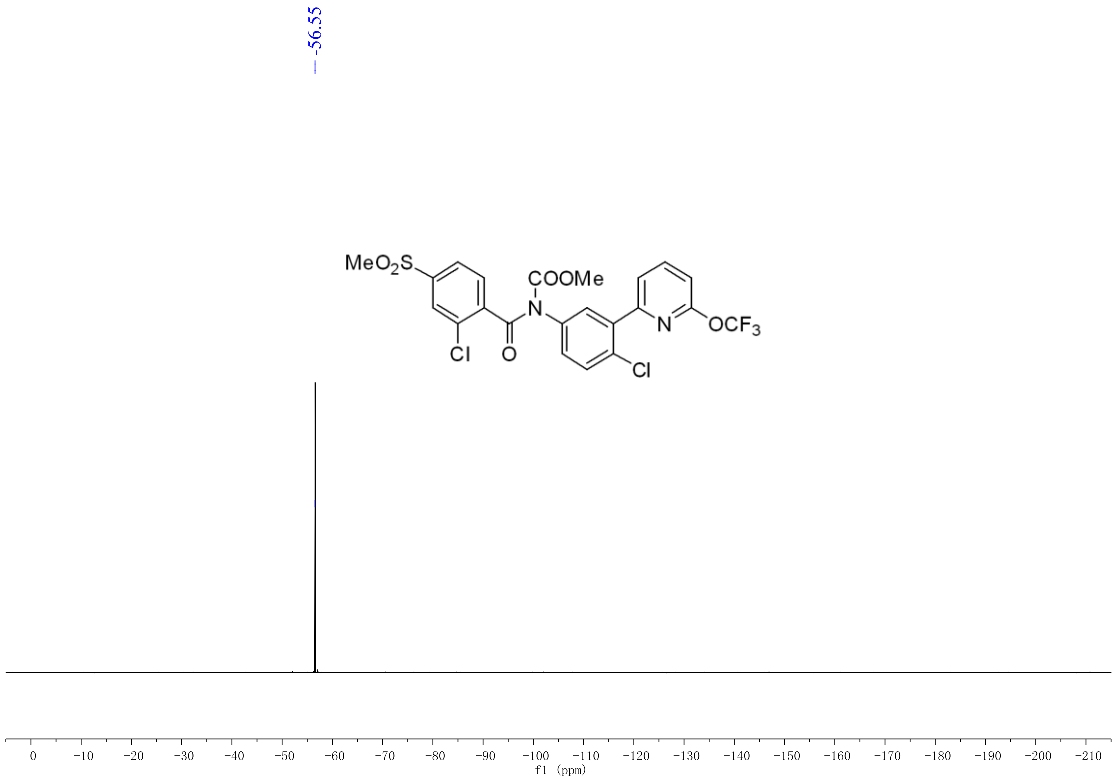

Supplementary Figure 158. ^{19}F NMR spectrum (376 MHz, $CDCl_3$) of **4nn**

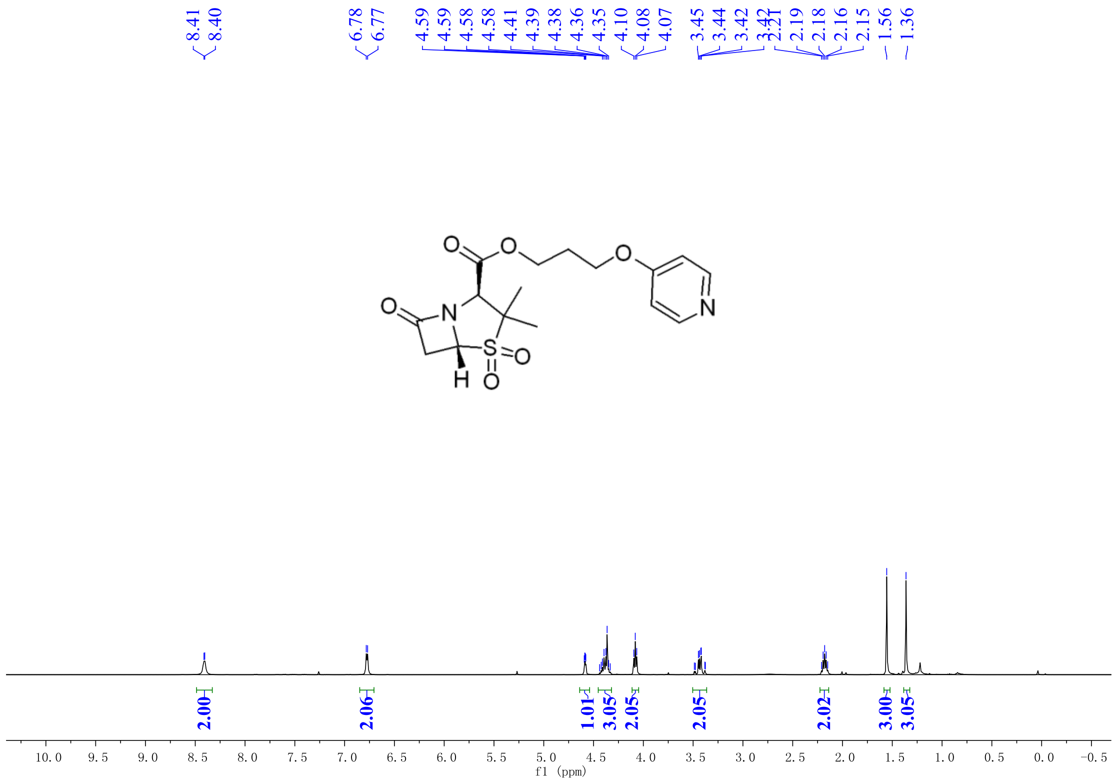

Supplementary Figure 159. ^{1}H NMR spectrum (400 MHz, $CDCl_3$) of **1oo**

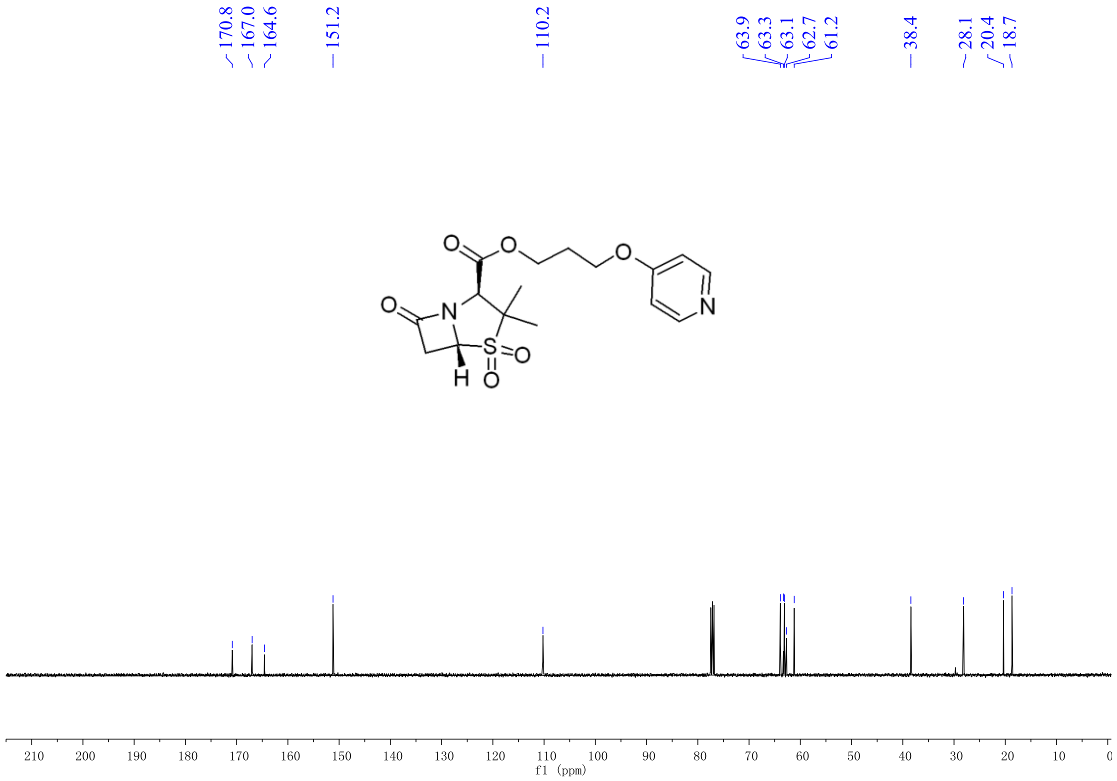

Supplementary Figure 160. ^{13}C NMR spectrum (101 MHz, $CDCl_3$) of **1oo**

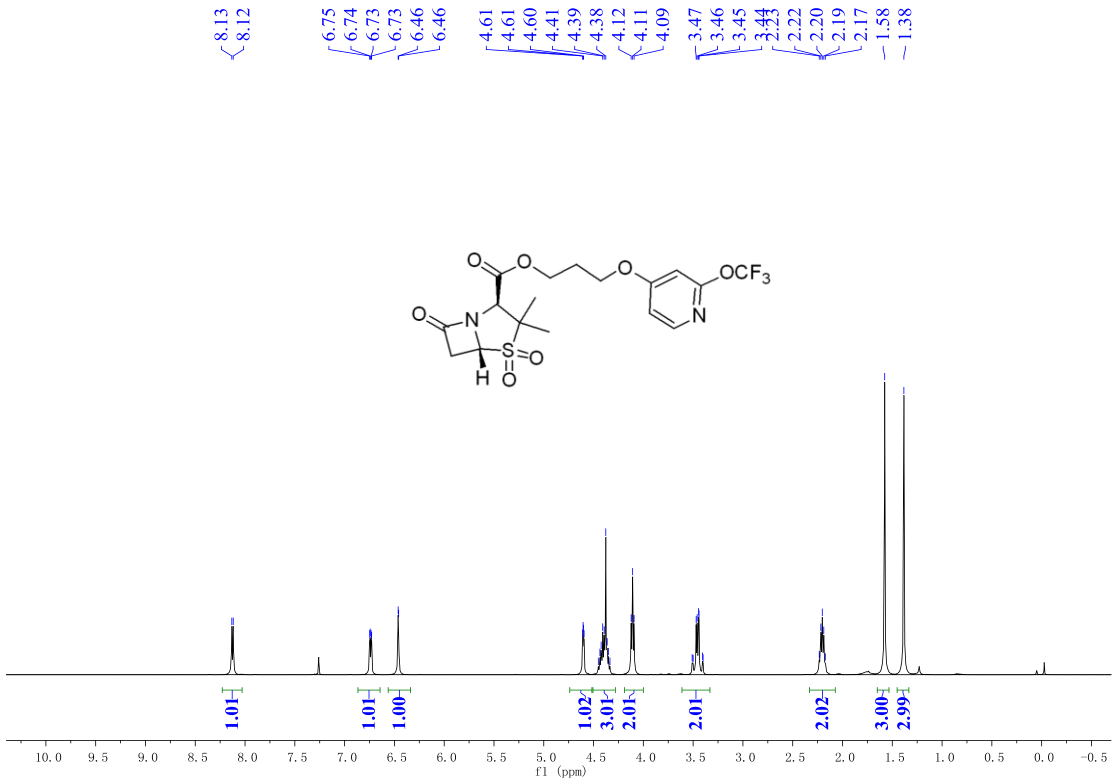

Supplementary Figure 161. ^{1}H NMR spectrum (400 MHz, $CDCl_3$) of **4oo**

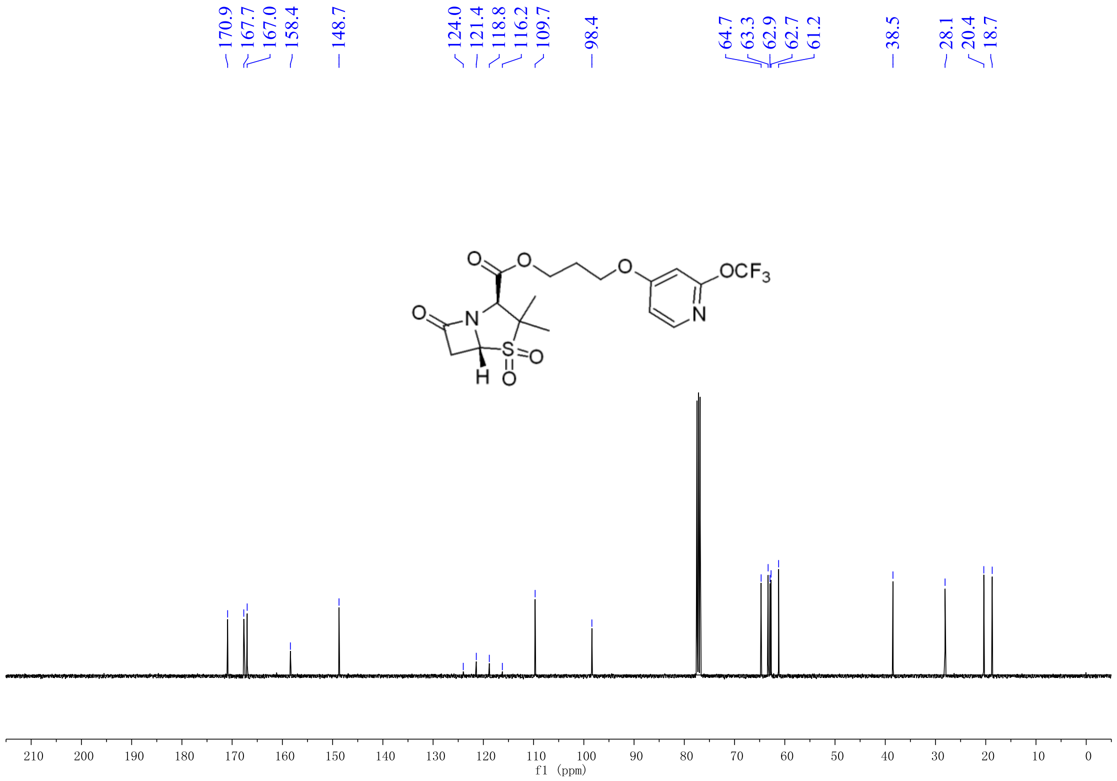

Supplementary Figure 162. ^{13}C NMR spectrum (101 MHz, $CDCl_3$) of **4oo**

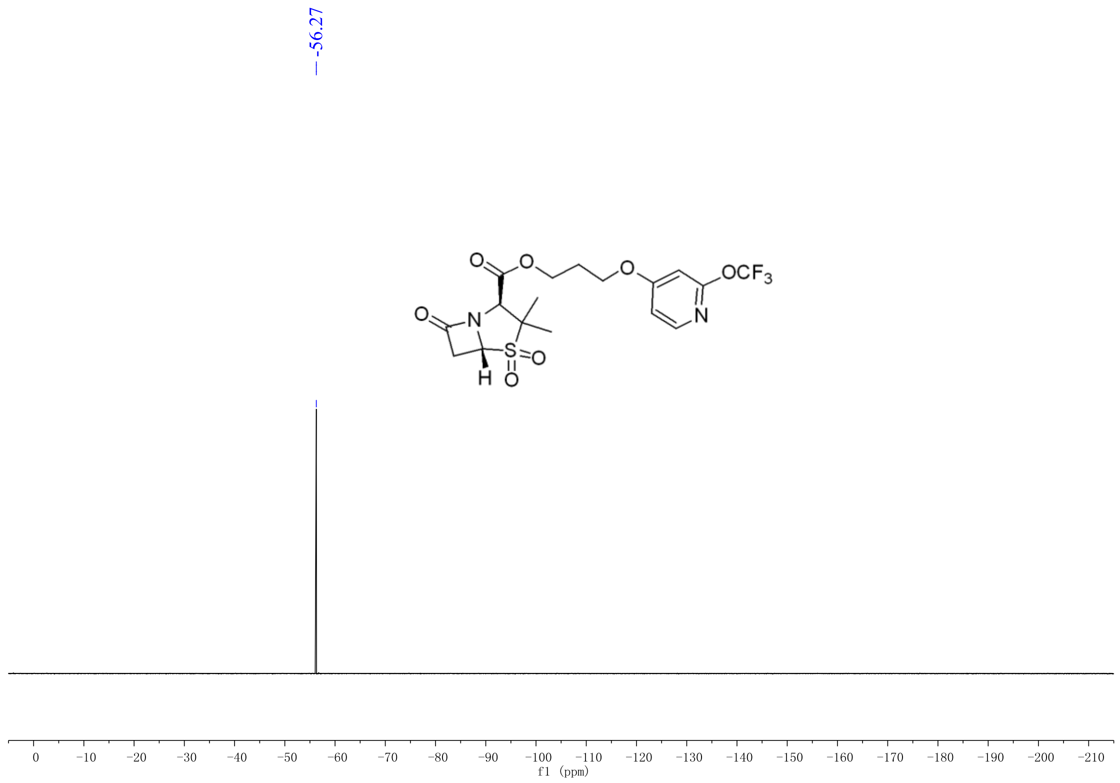

Supplementary Figure 163. ^{19}F NMR spectrum (376 MHz, $CDCl_3$) of **4oo**

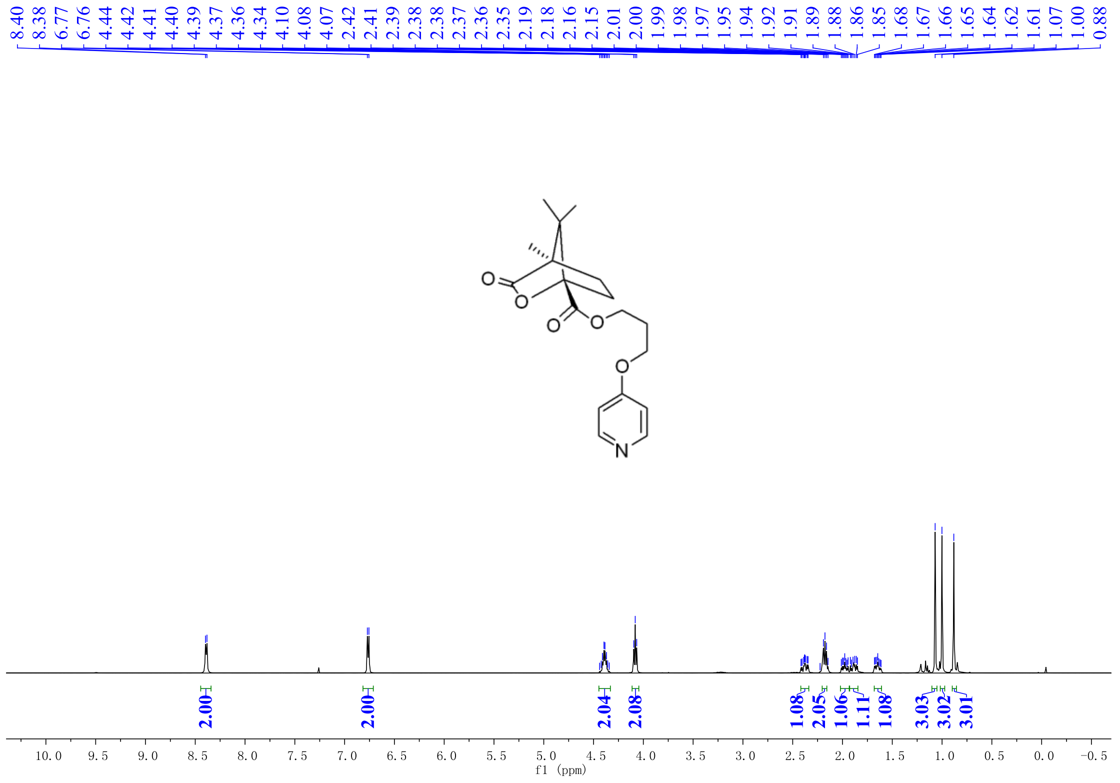

Supplementary Figure 164. ^{1}H NMR spectrum (400 MHz, $CDCl_3$) of **1pp**

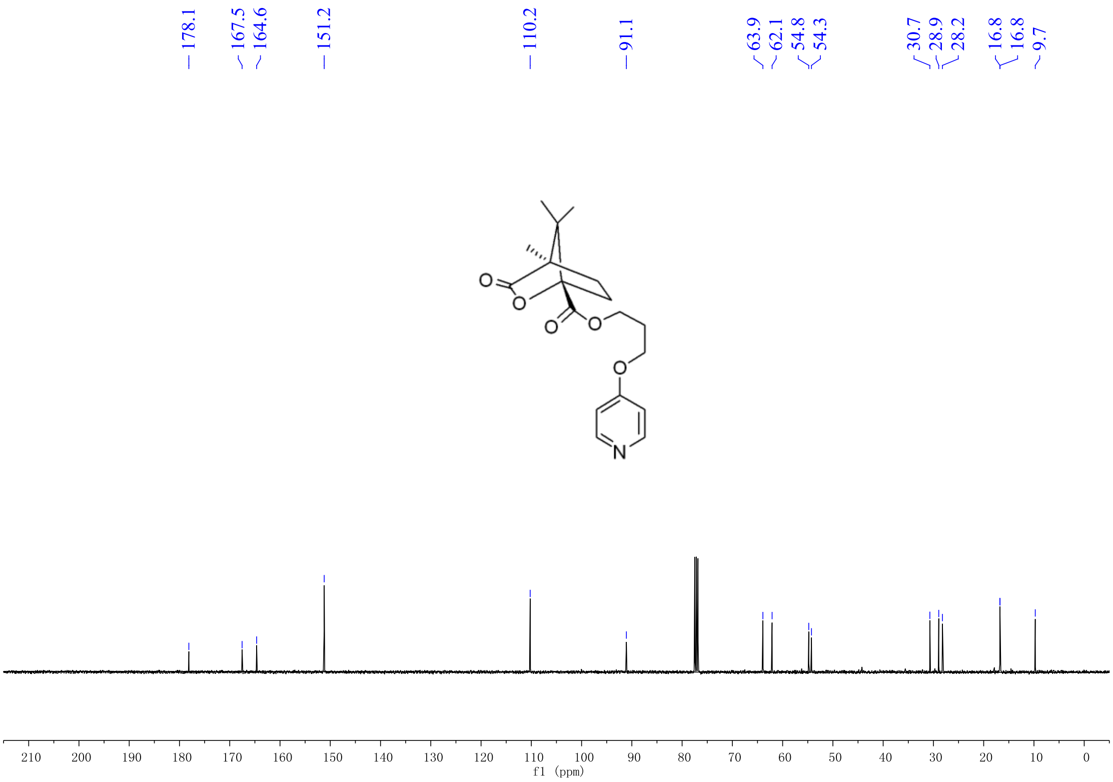

[165]**Supplementary Figure 165.** ^{13}C NMR spectrum (101 MHz, $CDCl_3$) of **1pp**

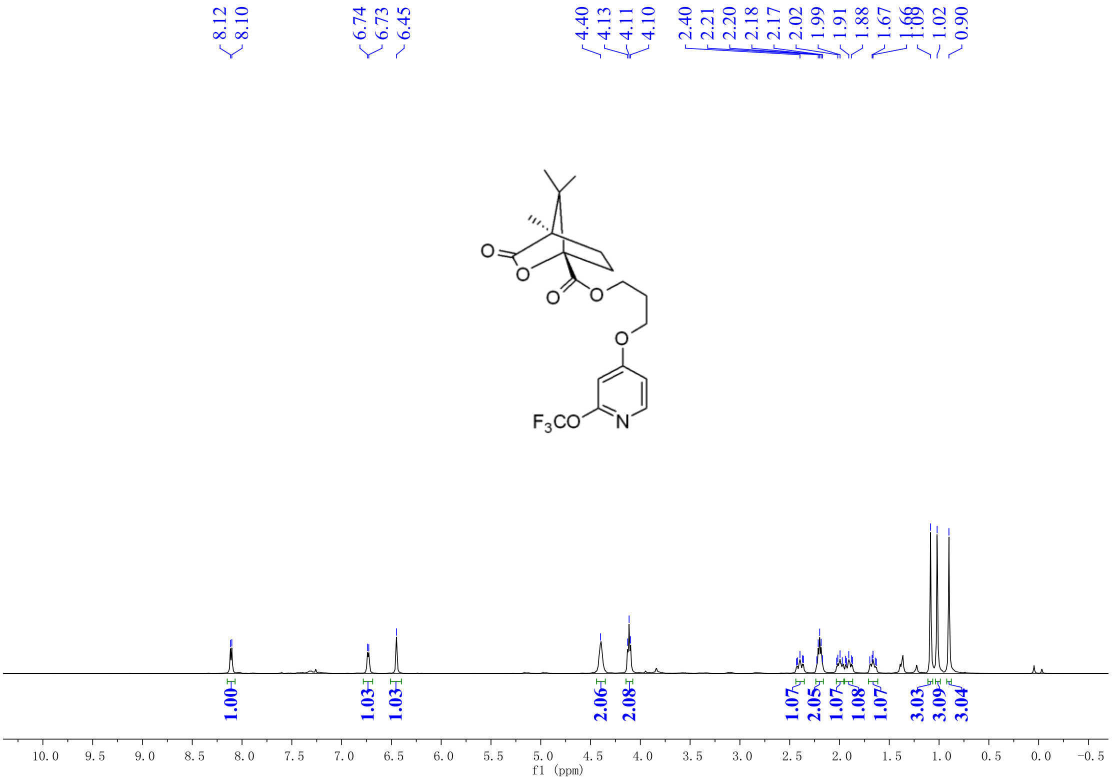

Supplementary Figure 166. ^{1}H NMR spectrum (400 MHz, $CDCl_3$) of **4pp**

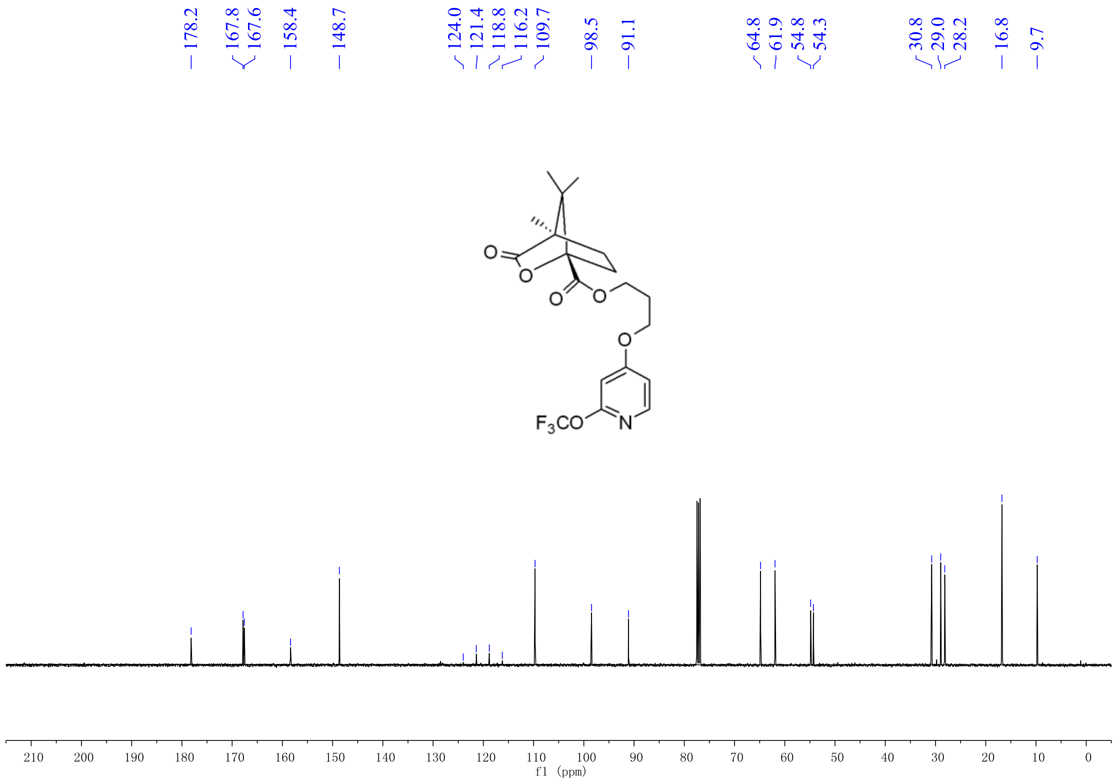

Supplementary Figure 167. ^{13}C NMR spectrum (101 MHz, $CDCl_3$) of **4pp**

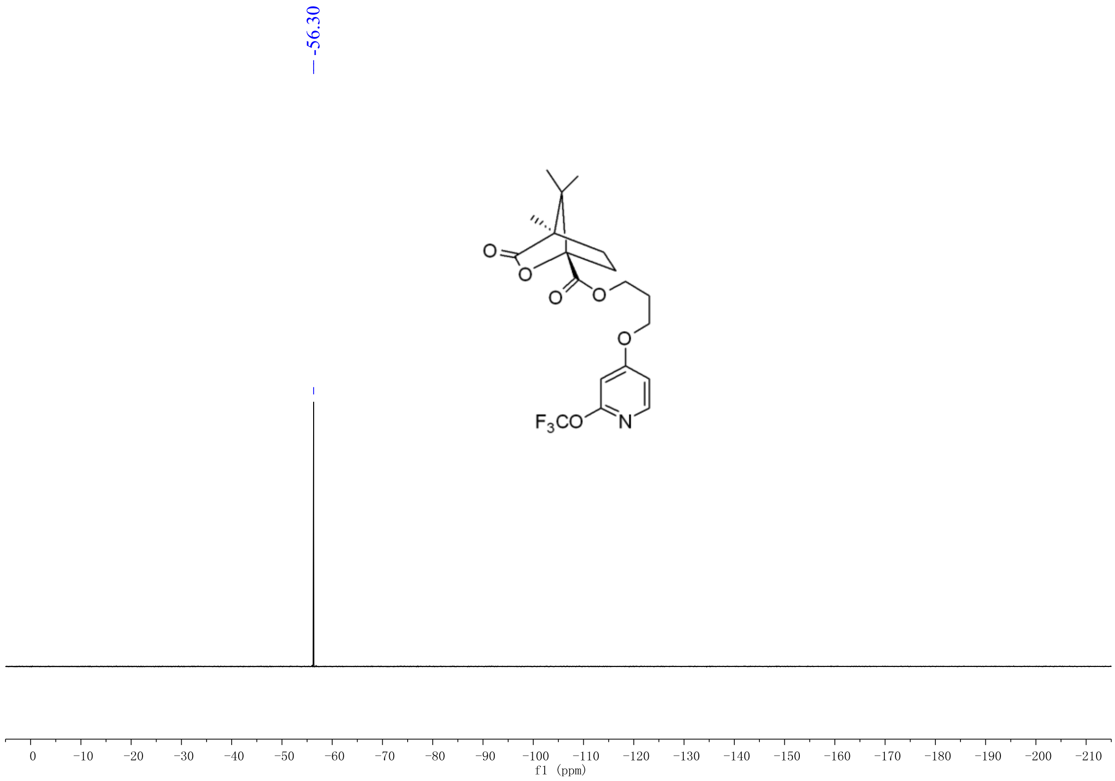

Supplementary Figure 168. ^{19}F NMR spectrum (376 MHz, $CDCl_3$) of **4pp**

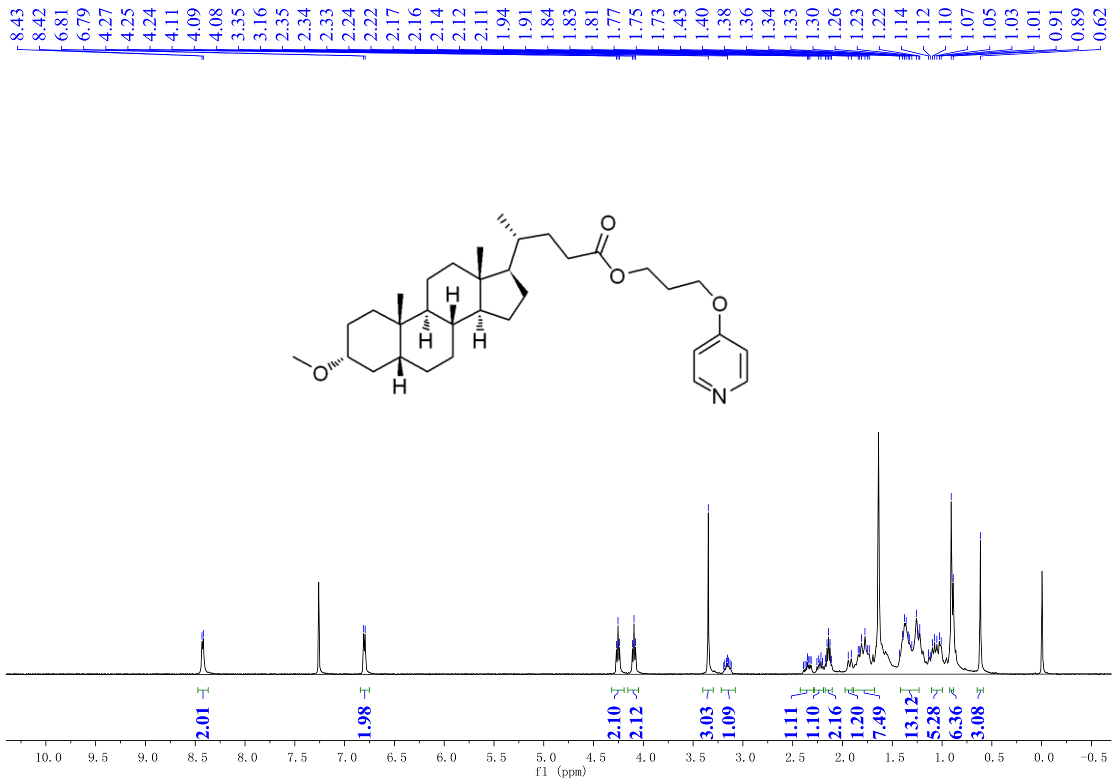

Supplementary Figure 169. ^{1}H NMR spectrum (400 MHz, $CDCl_3$) of **1qq**

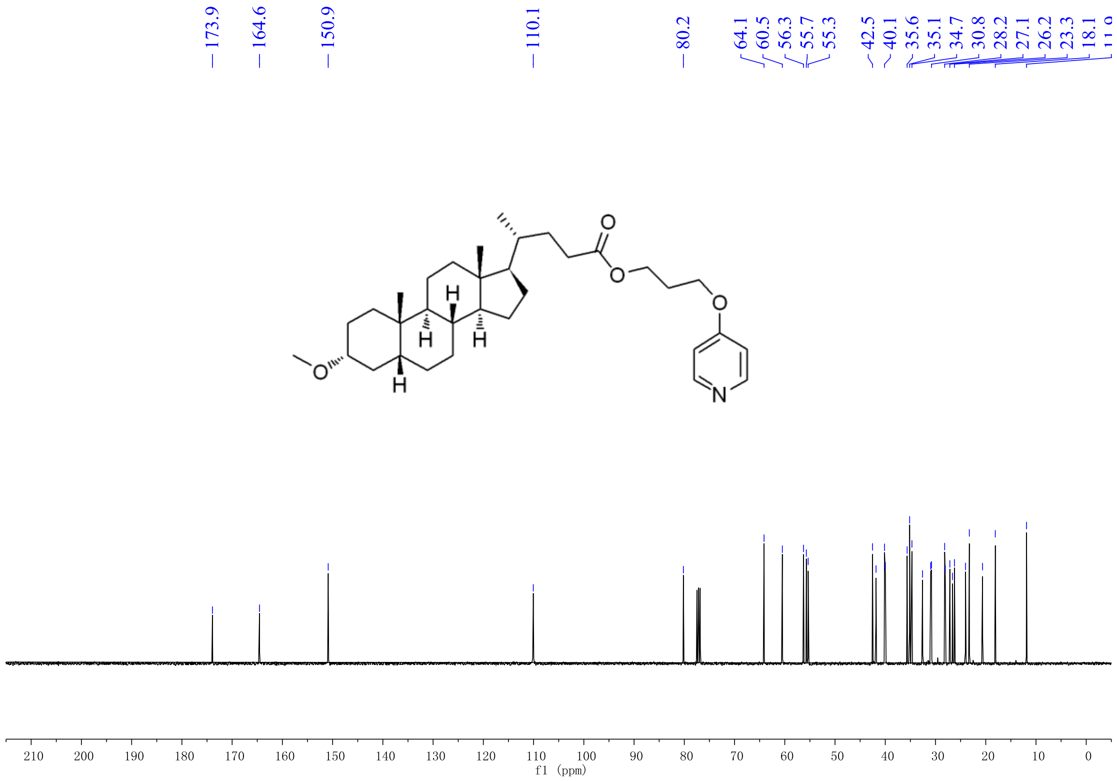

Supplementary Figure 170. ^{13}C NMR spectrum (101 MHz, $CDCl_3$) of **1qq**

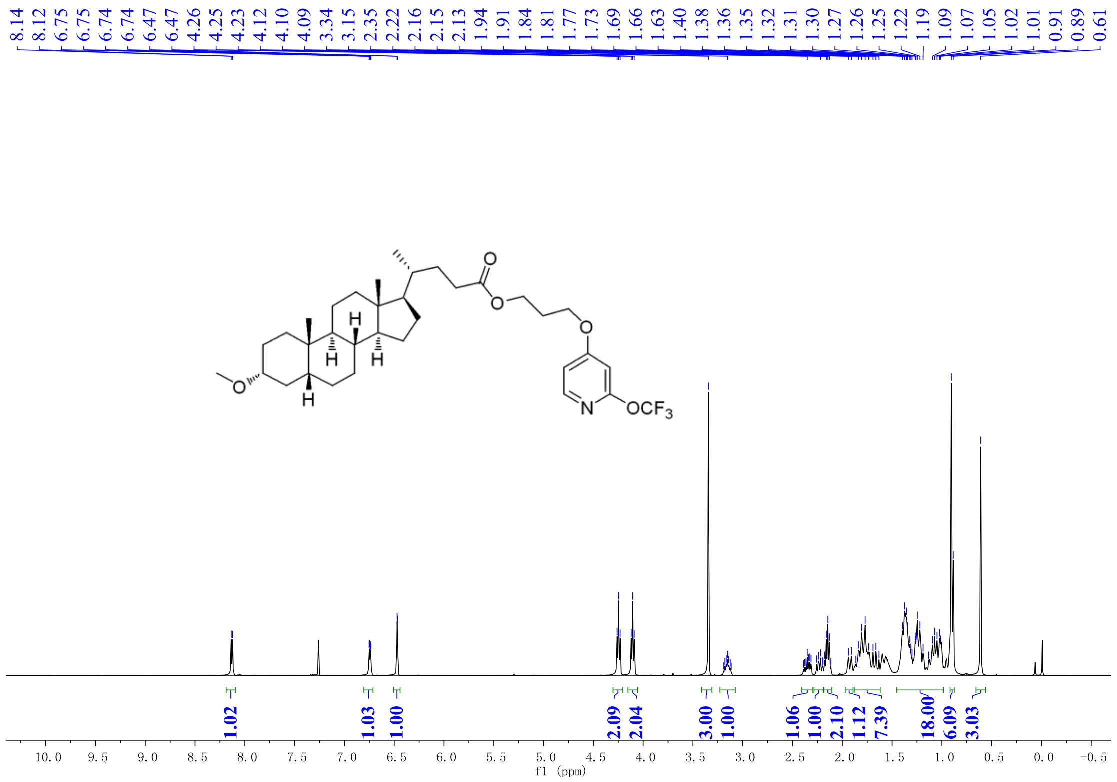

Supplementary Figure 171. ^{1}H NMR spectrum (400 MHz, $CDCl_3$) of **4qq**

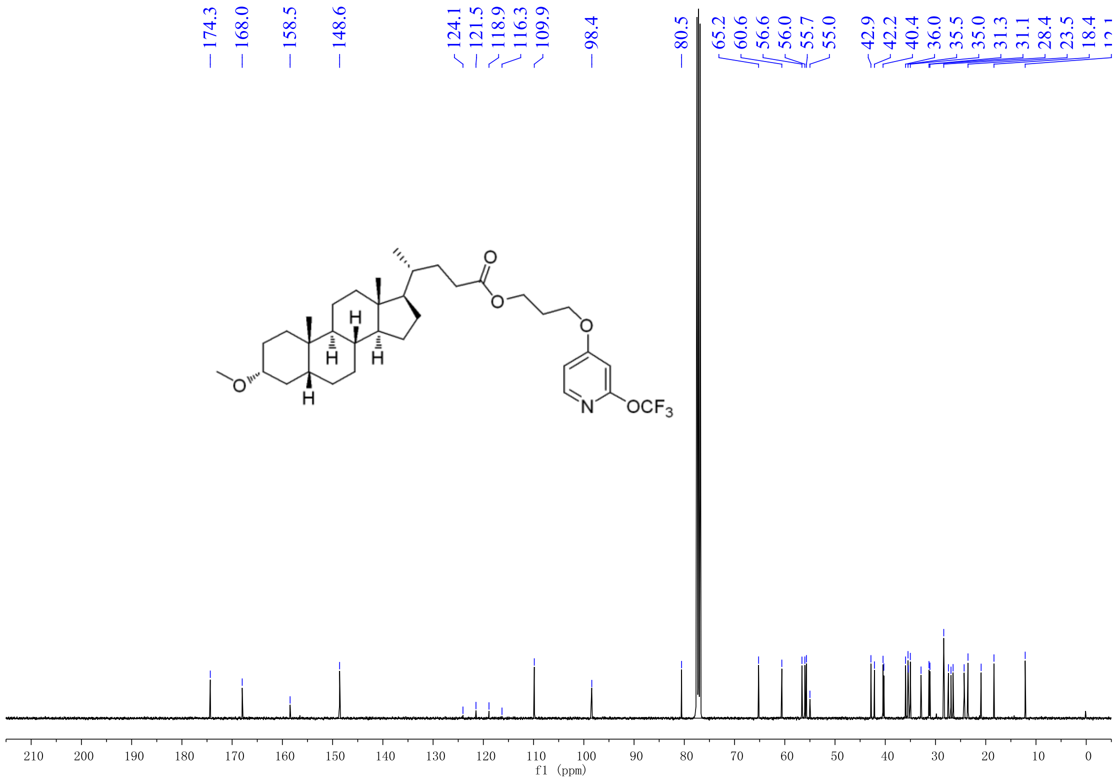

Supplementary Figure 172. ^{13}C NMR spectrum (101 MHz, $CDCl_3$) of **4qq**

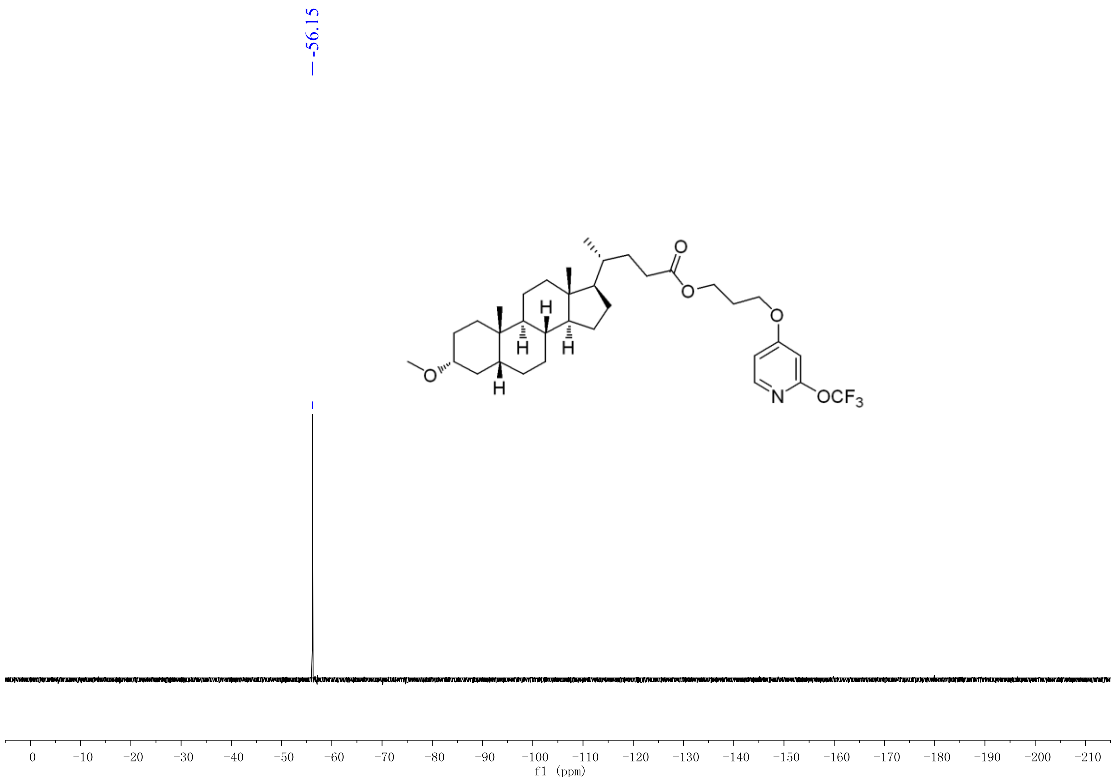

Supplementary Figure 173. ^{19}F NMR spectrum (376 MHz, $CDCl_3$) of **4qq**

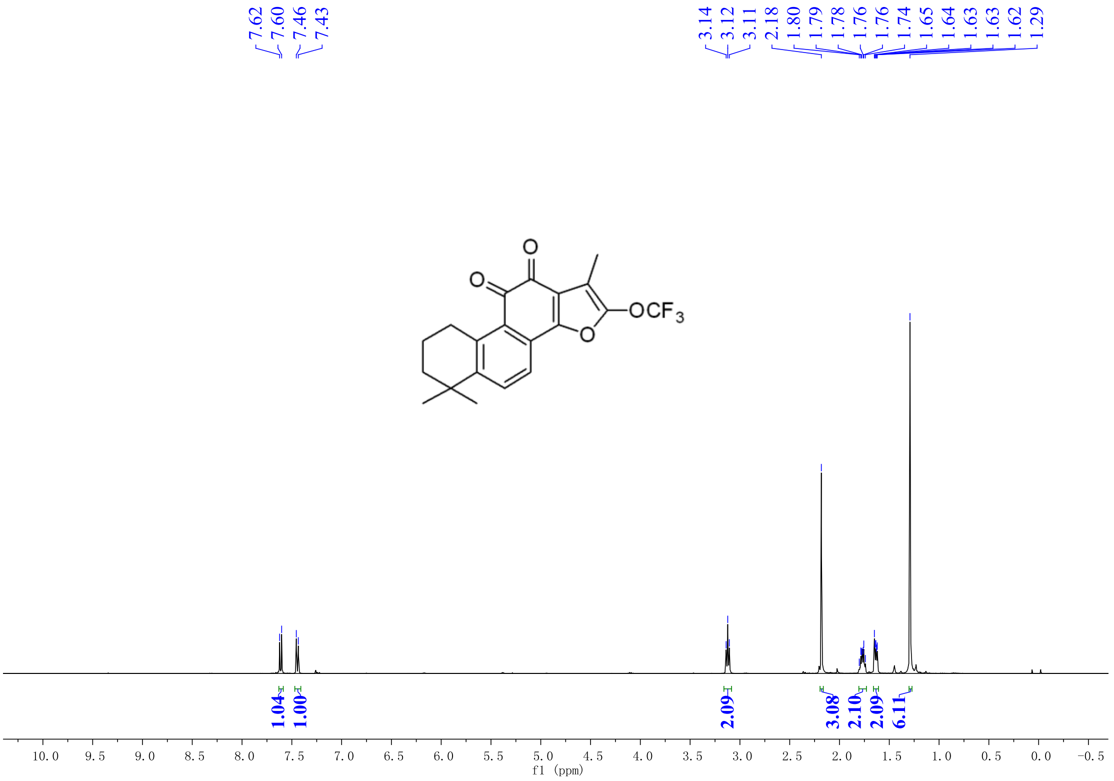

Supplementary Figure 174. ^{1}H NMR spectrum (400 MHz, $CDCl_3$) of **4rr**

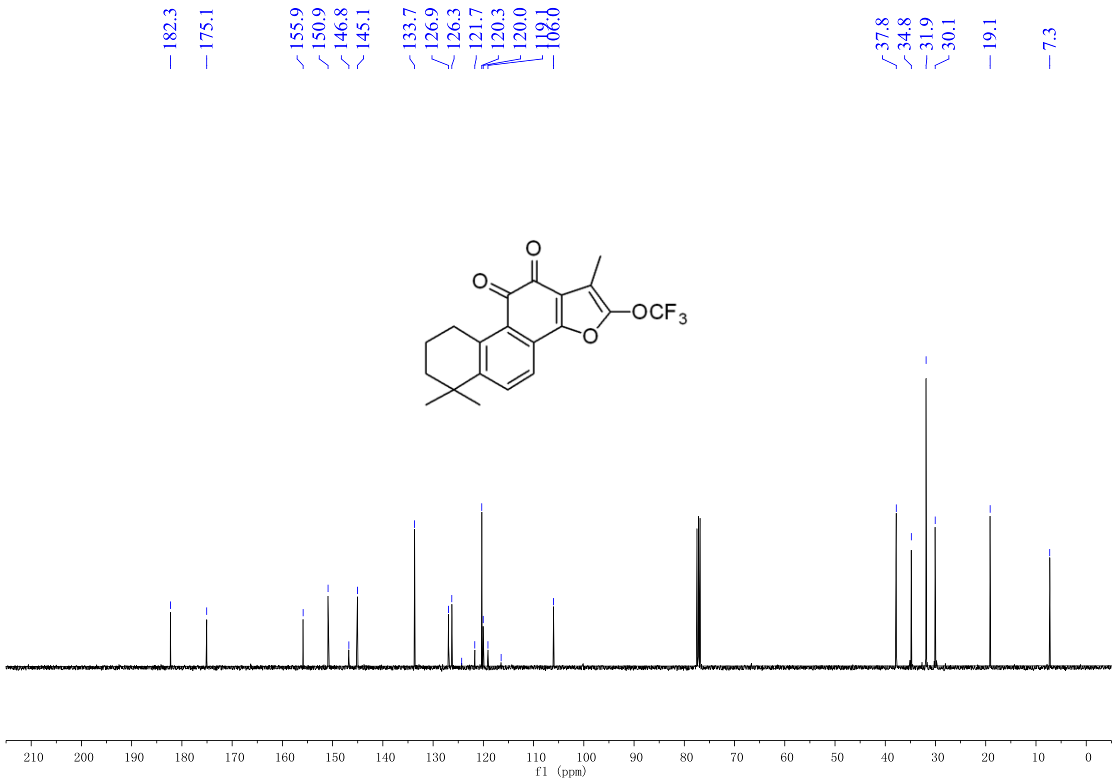

Supplementary Figure 175. ^{13}C NMR spectrum (101 MHz, $CDCl_3$) of **4rr**

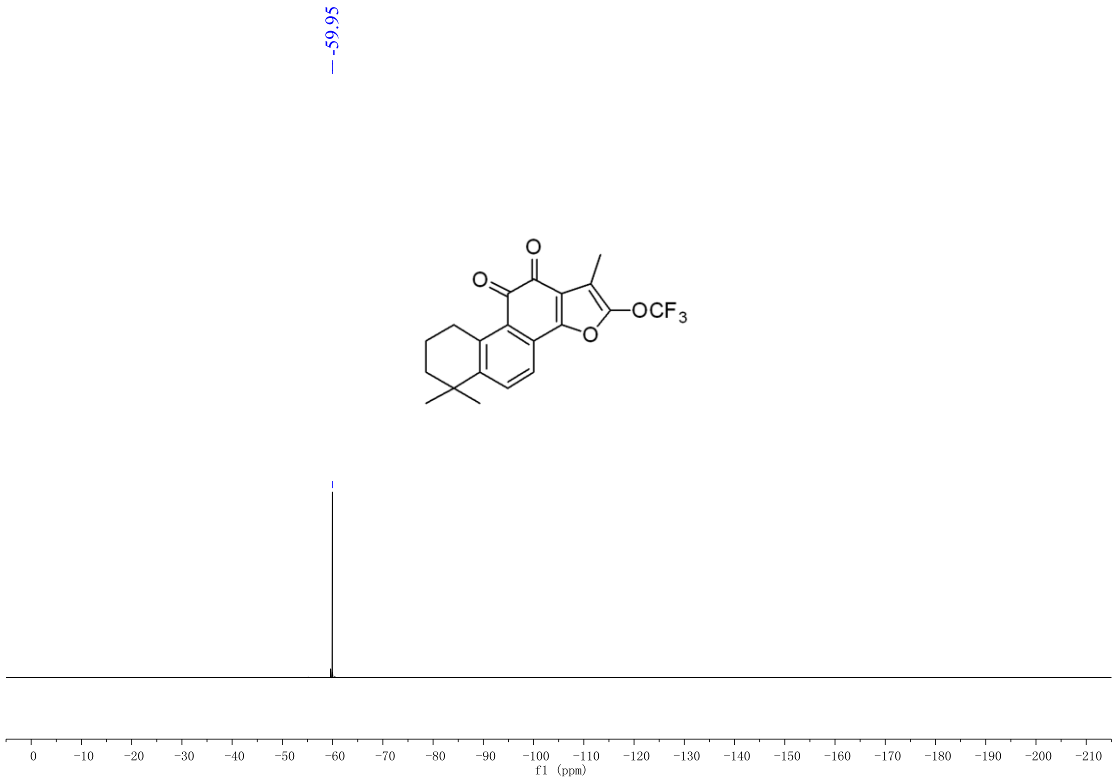

Supplementary Figure 176. ^{19}F NMR spectrum (376 MHz, $CDCl_3$) of **4rr**

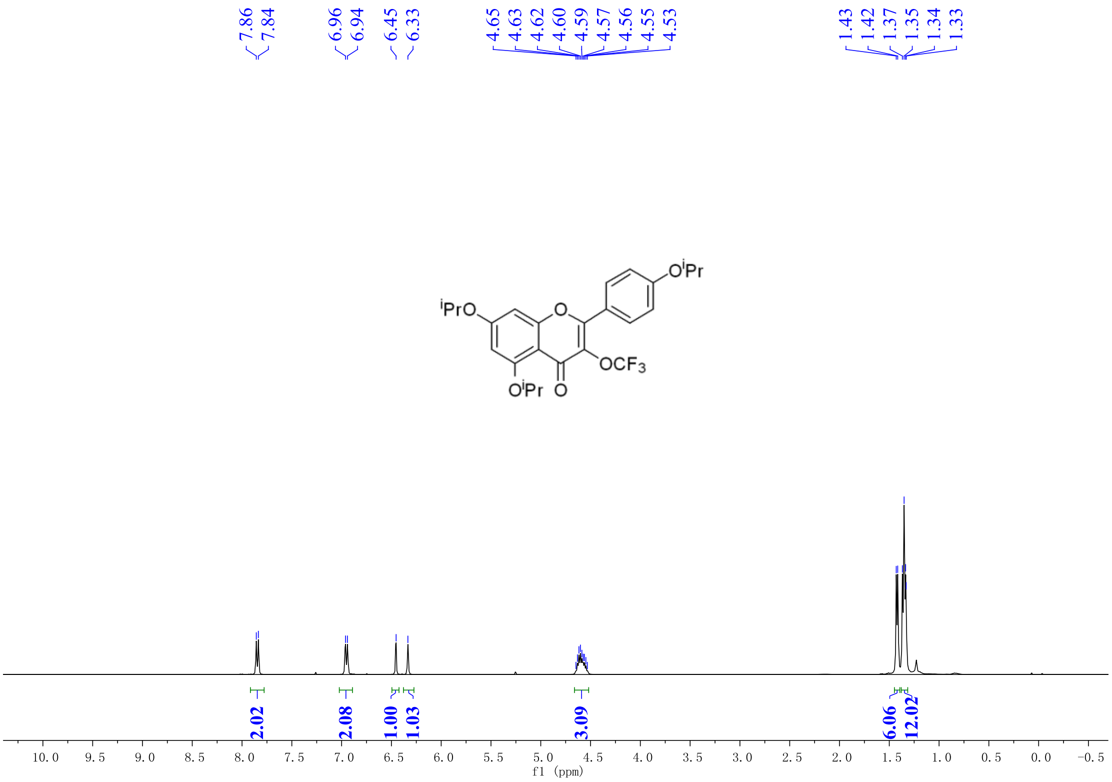

Supplementary Figure 177. ^{1}H NMR spectrum (400 MHz, $CDCl_3$) of **4ss**

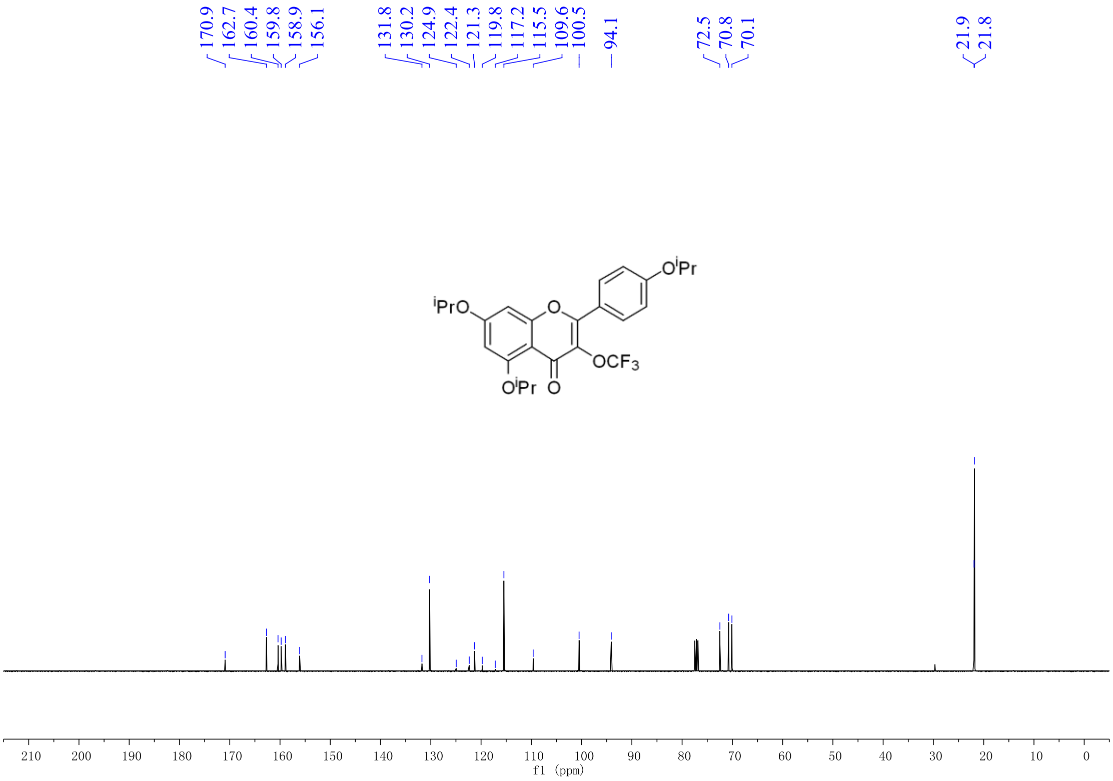

Supplementary Figure 178. ^{13}C NMR spectrum (101 MHz, $CDCl_3$) of **4ss**

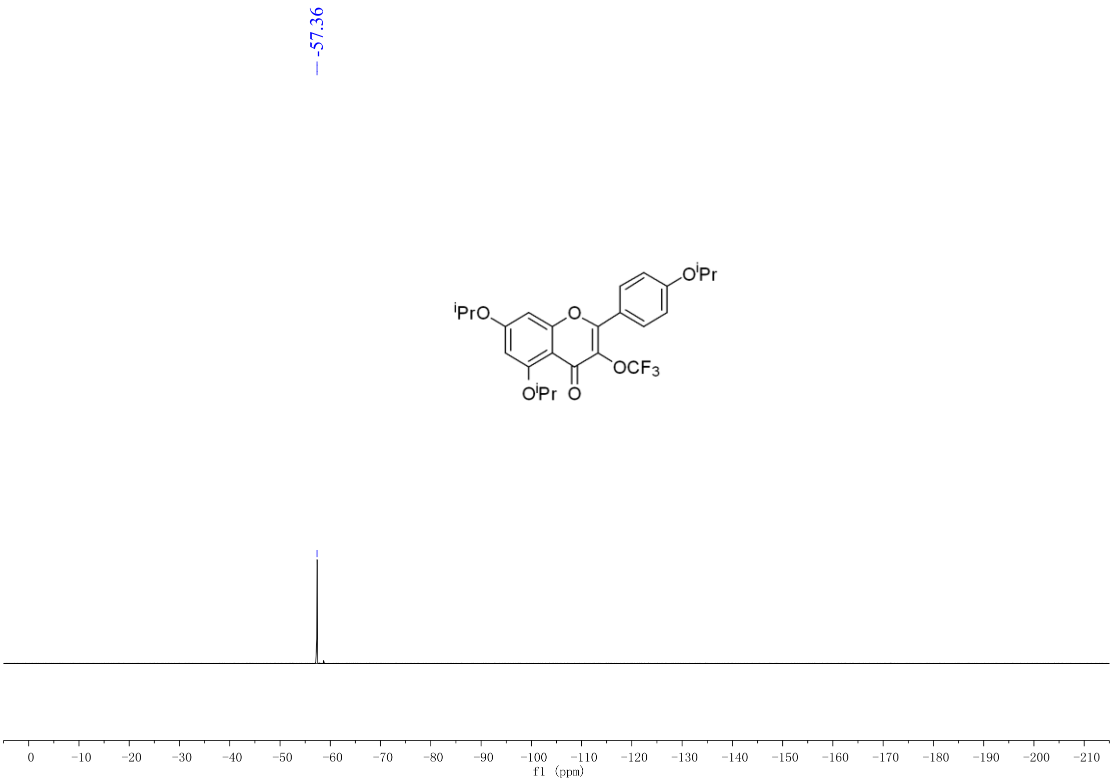

Supplementary Figure 179. ^{19}F NMR spectrum (376 MHz, $CDCl_3$) of **4ss**

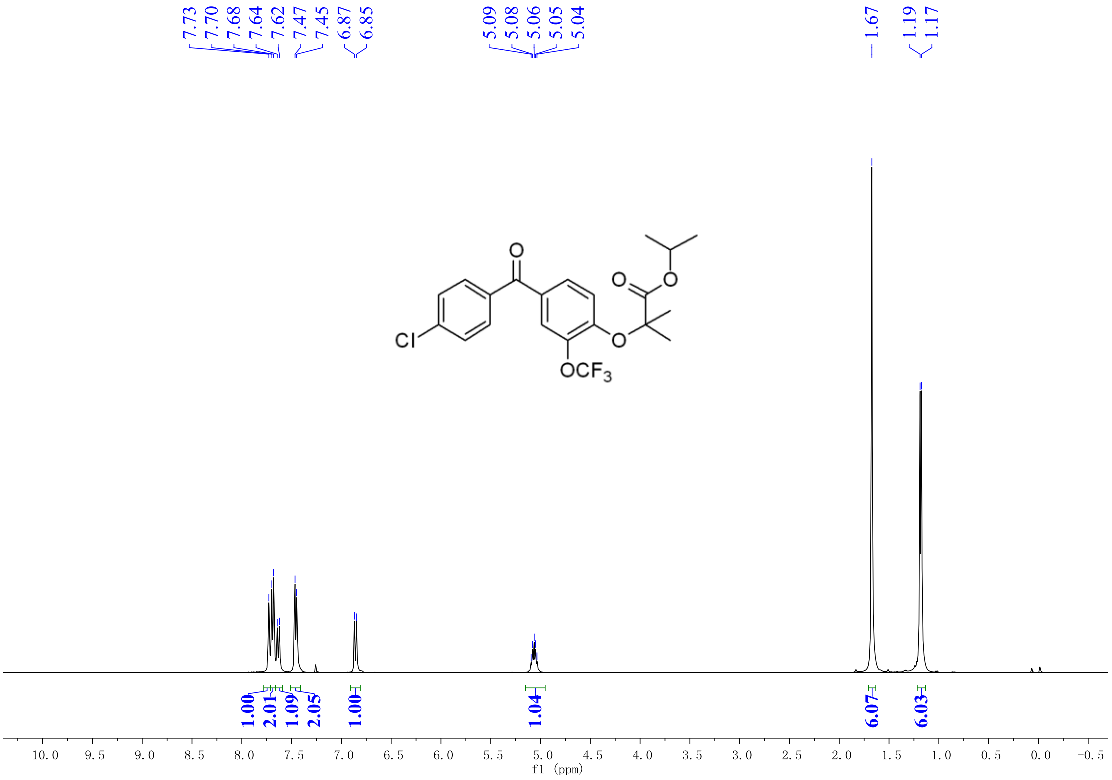

Supplementary Figure 180. ^{1}H NMR spectrum (400 MHz, $CDCl_3$) of **4tt**

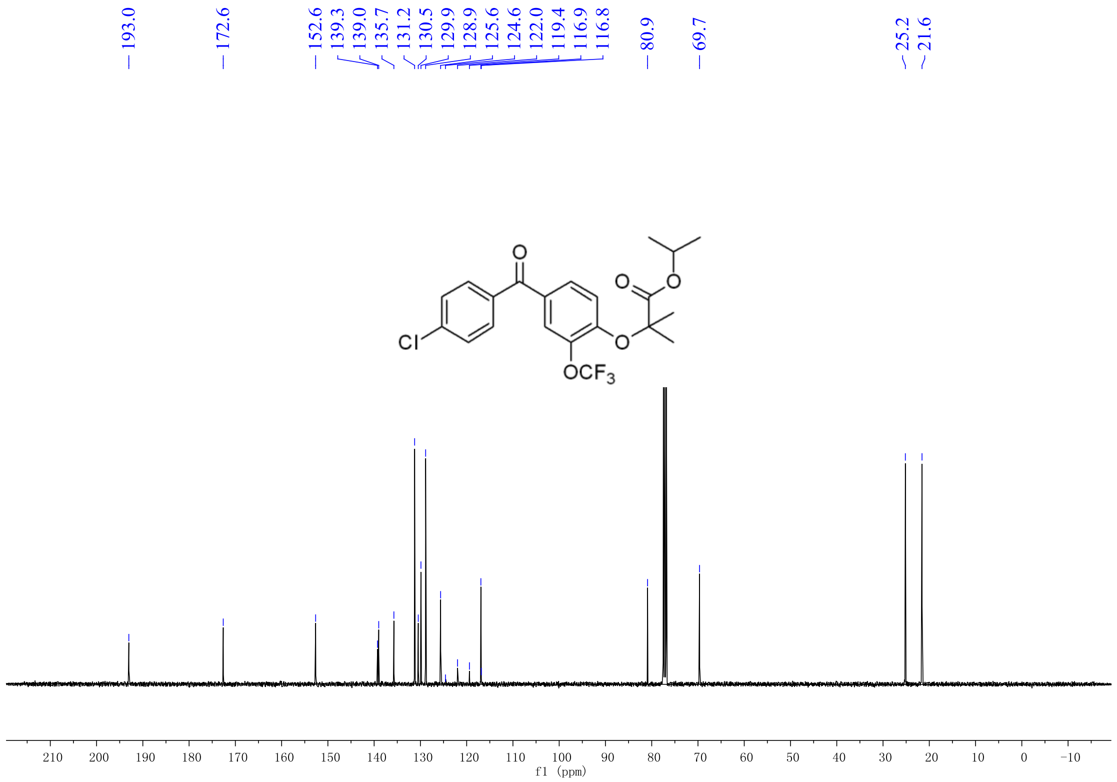

Supplementary Figure 181. ^{13}C NMR spectrum (101 MHz, $CDCl_3$) of **4tt**

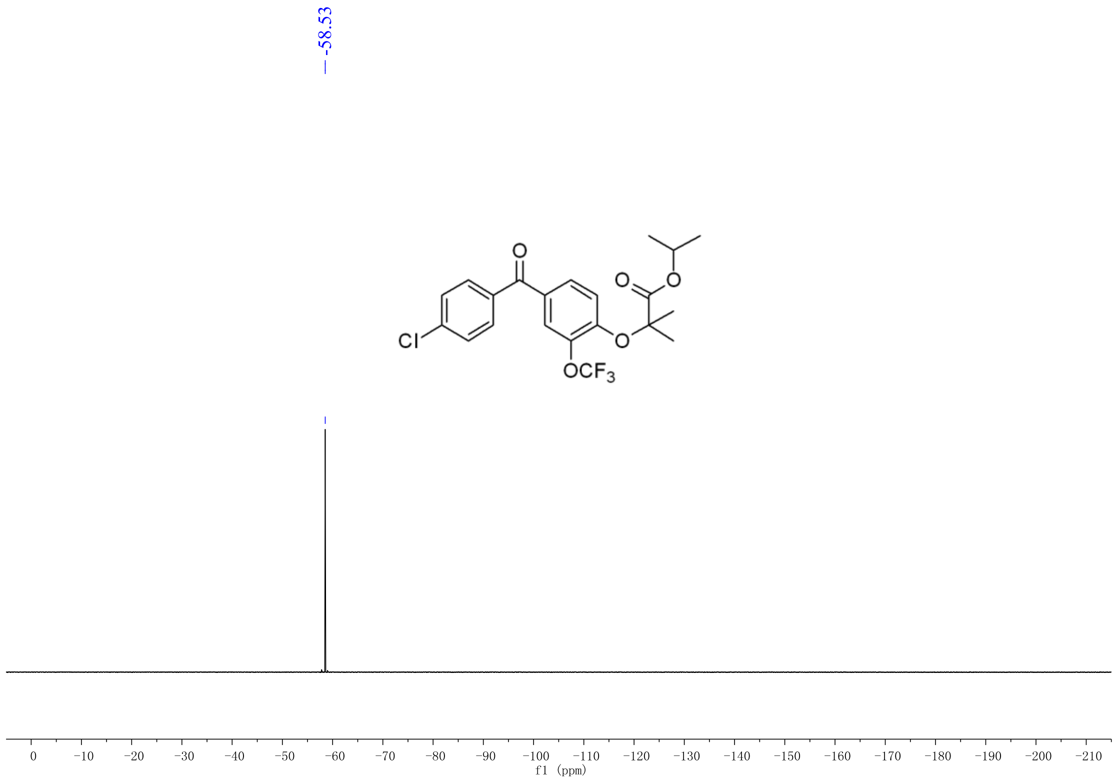

Supplementary Figure 182. ^{19}F NMR spectrum (376 MHz, $CDCl_3$) of **4tt**

Supplementary References

1. Guo, S.; Cong, F.; Guo, R.; Wang, L.; Tang, P. Asymmetric silver-catalysed intermolecular bromotrifluoromethoxylation of alkenes with a new trifluoromethoxylation reagent. *Nat. Chem*. **9**, 546-551 (2017).

2.Yuan, G.; Wang, F; Tang, P.; Liang, H. Metal-free ^{18}F-labeling of aryl-CF_2H via nucleophilic radiofluorination and oxidative C–H activation. *Chem. Commun*, **53**, 126-129 (2017).

3. Liang, A.; Han, S.; Wu, Y.; Wu, Y. S. Regioselective Synthesis of N‑Heteroaromatic Trifluoromethoxy Compounds by Direct O−CF3 Bond Formation. *Chem.Eur.J*. **22**, 5102-5106 (2016).

4. Engelhard, D. M. et al. Reversible Stabilization of Transition‑Metal‑Binding DNA G‑Quadruplexes. *Angew. Chem. Int. Ed*. **52**, 12843-12847 (2013).

5. Fortage, J. et al. Designing Multifunctional Expanded Pyridiniums: Properties of Branched and Fused Head-to-Tail Bipyridiniums. *J. Am. Chem. Soc*. **132**, 16700-16713 (2010).

6. Chatalova-Sazepin, C; Binayeva, M; Epifanov, M; Zhang, W; Foth, P; Amador, C; Jagdeo, M;Boswell, B. R; Sammis, G. M. Xenon difluoride mediated fluorodecarboxylations for the syntheses of di-and trifluoromethoxyarenes. *Org. Lett.* **18**, 4570-4573 (2016).

7. Khotavivattana, T.; Verhoog, S.; Tredwell, M.; Wheelhouse, K.; Lee Collier, T.; Gouverneur, V. ^{18}F‑Labeling of Aryl‑SCF_3, ‑OCF_3 and ‑$OCHF_2$ with [^{18}F]Fluoride. *Angew. Chem. Int. Ed.* **54**, 9991-9995 (2015).

8. Yang, S.; Chen, M.; Tang, P. Visible‑Light Photoredox‑Catalyzed and Copper‑Promoted Trifluoromethoxylation of Arenediazonium Tetrafluoroborates. *Angew. Chem. Int. Ed.* **58**, 7840-7844 (2019).

9. Hofmayer, M. S.; Lutter, F. H.; Grokenberger, L.; Hammann, J. M.; Knochel, P. Practical Ni-Catalyzed Cross-Coupling of Unsaturated Zinc Pivalates with Unsaturated Nonaflates and Triflates. *Org.Lett*. **21**, 36-39 (2019).

10. Cho, J.; Roh, G.; Cho, E. Visible-Light-Promoted Synthesis of Dibenzofuran Derivatives. *J. Org. Chem*. **83**, 805-811 (2018).

11. Fier, P. S.; Hartwig, J. F. Synthesis and Late-Stage Functionalization of Complex Molecules through C−H Fluorination and Nucleophilic Aromatic Substitution. *J. Am. Chem. Soc*. **136**, 10139-10147 (2014).

12. Pan, G.; Zhao, L.; Xiao, Na.; Yao, Q.; Lu, K.; Yu, P. Total synthesis of 8-(6″-umbelliferyl)-apigenin and its analogs as anti-diabetic reagents. *European Journal of Medicinal Chemistry*. ***122***, 674-683 (2016).

13. Jelier, B. J., Tripet, P. F., Pietrasiak, E., Franzoni, I., Jeschke, G. & Togni, A. Radical Trifluoromethoxylation of Arenes Triggered by a Visible-Light-Mediated N-O Bond Redox Fragmentation. *Angew. Chem. Int. Ed.* **57**, 13784-13789 (2018).
